# Author Correction: Global influence of mantle temperature and plate thickness on intraplate volcanism

**DOI:** 10.1038/s41467-022-29128-4

**Published:** 2022-03-18

**Authors:** P. W. Ball, N. J. White, J. Maclennan, S. N. Stephenson

**Affiliations:** 1 Bullard Laboratories, Department of Earth Sciences, University of Cambridge, Madingley Rise, Cambridge, UK; 2 Research School of Earth Sciences, Australian National University, Canberra, ACT Australia; 3 Department of Earth Sciences, University of Oxford, Oxford, UK

**Keywords:** Geochemistry, Geodynamics, Geophysics

Correction to: *Nature Communications* 10.1038/s41467-021-22323-9, published online 6 April 2021.

The original version of this Article contained an error in Equation 8. In the original version, we state that:

$${c}_{l}=\frac{{c}_{{{{\mathrm{s}}}}}(1-X)}{\bar{D}-\bar{P}{X}}.$$

However, the correct form of Equation 8 is:

$${c}_{l}=\frac{{c}_{{{{\mathrm{s}}}}}(1-X)}{\bar{D}({{1}-{{X}_{0}}})-\bar{P}({X}-{X_{0}})}.$$

This error arose since this equation is also incorrectly stated in Ref 41. It is crucial to note that this mistake is purely typographic. This equation is correctly included in our code and so this mistake does not affect our data, results, or conclusions.

