# Supplementary material for: Global influence of mantle temperature and plate thickness on intraplate volcanism
Source: Nat Commun. 2021 Apr 6;12:2045. doi: 10.1038/s41467-021-22323-9 (PMC8024351; doi:10.1038/s41467-021-22323-9)
Supplement: Supplementary file 1 — Supplementary Information [file 41467_2021_22323_MOESM1_ESM.pdf]

Supplementary Information: Global Influence of Mantle Temperature and Plate Thickness on Intraplate Volcanism

P. W. Ball[1,2*], N. J. White[1*], J. Maclennan[1] and S. N. Stephenson[1,3]

[1.] Bullard Laboratories, Department of Earth Sciences, University of Cambridge, Madingley Rise, Cambridge, CB3 0EZ, United Kingdom.
[2.] Research School of Earth Sciences, Australian National University, Canberra, ACT 2601, Australia.
[3.] Department of Earth Sciences, University of Oxford, Oxford, OX1 3AN, United Kingdom.
* corresponding authors: patrick.ball@anu.edu.au, njw10@cam.ac.uk

3rd March 2021

1 Supplementary Tables

Supplementary Table 1: References by region for Supplementary Database 1 of continental intraplate volcanism.

Province	References
Africa	
Afar	[25, 50, 63, 64, 77, 78, 109, 182, 232, 264, 291, 333, 358, 443, 423, 610, 761, 840, 845, 931]
Aïr	[688]
Angola	[146]
Atlas	[96, 97, 100, 122, 293, 314, 54]
Ethiopia	[49, 48, 50, 66, 89, 94, 99, 115, 133, 134, 183, 214, 265, 332, 358, 372, 382, 383, 388, 422, 443, 474, 485, 528, 690, 701, 730, 731, 739, 783, 790, 839, 843, 840, 841, 844, 846, 904, 930, 931, 932, 948, 1018, 1017, 1019, 1038, 1040]
Cameroon	[15, 62, 266, 267, 281, 338, 337, 344, 425, 426, 515, 517, 555, 657, 658, 659, 744, 745, 748, 746, 747, 751, 810, 914, 913, 984, 1039]
Dakkar	[735]
Darfur	[241, 346, 613, 614, 743]
Hoggar	[14, 30, 53, 234, 235, 236, 300, 514, 667, 1031]
Madagascar	[68, 139, 228, 229, 230, 531, 681, 682, 683, 684, 685]
Northern Kenya	[47, 110, 114, 135, 203, 208, 214, 357, 358, 424, 513, 548, 569, 631, 632, 835, 836, 842, 875, 883, 884, 904, 926]
Libya	[61, 69, 88, 623, 696]
Southern Kenya	[56, 214, 251, 250, 244, 512, 524, 569, 663, 632, 633, 644, 702, 703, 772, 773, 834, 836, 893, 894]
Tanganyika	[38, 55, 72, 95, 163, 213, 251, 243, 258, 254, 255, 355, 449, 457, 482, 518, 519, 520, 611, 612, 651, 723, 724, 727, 774, 793, 797, 837, 838, 847, 857, 909, 924]
Tibesti	[61, 397, 808]
Rungwe	[157, 354, 463, 651]
The Americas	
Anahim	[36, 102, 210, 285, 353, 554, 649]
Mexico	[10, 142, 155, 247, 248, 245, 246, 276, 391, 653, 678, 706, 766, 714, 788, 826, 828, 827, 879, 912, 961, 962, 963, 964, 965, 966, 967, 968, 982]
Northeast Brazil	[869, 343, 407, 533, 536, 608, 741, 831, 882]
Northern Cordillera	[4, 5, 306, 307, 308, 345, 483, 711, 850]
Patagonia	[137, 186, 282, 283, 323, 394, 393, 392, 809, 903]
Western USA	[532, 936, 1004]
Anatolia, Arabia and Iran	
Al Lith	[767]
As Sirat	[290, 211]
Central Anatolia	[8, 27, 29, 40, 51, 380, 379, 559, 755, 771, 794, 816, 1016]
Dead Sea Region	[2, 617, 551, 626, 625, 720, 866, 872, 945]
East Anatolia	[29, 40, 52, 312, 313, 273, 526, 527, 560, 575, 576, 577, 574, 622, 623, 737, 755, 762, 763, 764, 779]
Euphrates	[262, 549, 551, 573, 617, 872, 945]
Harrat Al Kishb	[13, 32, 101, 145, 978, 979]
Harrat Ash Shaam	[17, 18, 32, 65, 101, 136, 389, 486, 487, 489, 550, 573, 617, 720, 728, 871, 870, 873, 898, 899, 925, 945, 998, 995, 999, 996, 997]
Harrat Khaybar	[84, 101, 136, 144]
Harrat Lunayyir	[16, 32, 101, 136, 294]
Harrat Rahat	[32, 101, 136, 143, 289, 716, 717, 726]
Harrat Uwayrid	[32, 136]
Hutaymah	[32, 295]
South Dead Sea	[101, 315, 450, 899, 1036, 1037]
South Saudi Arabia	[32, 101, 59, 211, 977]
West Anatolia	[9, 22, 23, 24, 28, 319, 402, 775, 799, 825]
Yemen	[57, 58, 101, 147, 168, 184, 221, 224, 687, 731, 829, 867, 977]
Iran	[12, 31, 103, 240, 599, 578, 680, 699, 757, 768, 852, 853, 980]
Australasia and Antarctica	
Antarctica	[46, 217, 476, 494, 529, 580, 588, 589, 655, 758, 769, 789]
New Zealand	[216, 362, 473, 675, 676, 770, 865, 942]
North NSW	[1, 231, 385, 537, 759, 760, 907, 916, 970, 1049]
Northern Queensland	[44, 60, 324, 491, 537, 759, 760, 900, 901, 917, 916, 1007, 1052]
Seamounts	[309, 672]
South NSW	[231, 324, 537, 715, 738, 759, 760, 918, 1052]
Southern Queensland	[324, 537, 918]
Tasmania	[395, 396, 674, 729]
Victoria	[124, 261, 317, 324, 340, 350, 504, 537, 435, 669, 670, 738, 776, 803, 802, 959, 976, 1006]
Europe	
Bohemian Massif	[140, 618, 877, 955, 956, 957, 958]
Eifel	[7, 116, 296, 420, 507, 509, 508, 510, 511, 543, 689, 692, 666, 863, 991]
Massif Central	[132, 165, 212, 233, 237, 238, 287, 288, 582, 591, 596, 597, 598, 618, 791, 1013, 1015, 1014]
Mt. Etna	[42, 86, 104, 225, 219, 274, 335, 923, 943, 944, 947]
Pannonian Basin	[26, 279, 318, 431, 493, 868]
Sardinia	[271, 328, 390, 619, 616, 620, 621, 618, 624, 705, 851]
Sicily Channel	[45, 108, 141, 194, 195, 272, 322, 782, 848, 849, 949]
Spain	[87, 148, 158, 159, 160, 215, 292, 618, 609, 954]

Continued Overleaf...

Province	References
Eastern Asia	
Borneo	[640]
Cambodia	[76]
Changbaishan Region	[37, 82, 178, 325, 478, 479, 556, 602, 603, 605, 785, 1032, 1055, 1046, 1065]
Dariganga	[325, 409, 466, 1048]
Gobi Altai	[786]
Halaha	[176, 467, 686]
Hainan	[325, 464, 1033]
Hangai	[35, 79, 193, 484, 686, 858]
Lake Baikal	[192, 321, 398, 399, 439, 490, 492, 500, 601, 733, 734, 811, 813, 812, 885, 950, 1035]
Korea	[37, 127, 128, 129, 130, 185, 540, 796, 855, 892, 928]
Nushan	[187, 189, 284, 325, 465, 593, 594, 603, 605, 785, 915, 988, 1027, 1047, 1063, 1064]
Shandong	[178, 189, 325, 592, 603, 605, 785, 1030, 1044, 1045]
Shanxi	[82, 178, 280, 325, 326, 408, 409, 603, 605, 922, 1028, 1029, 1053, 1059]
South China Sea	[1032]
Taiwan and Fujian	[82, 171, 190, 191, 325, 465, 481, 506, 603, 915, 986, 1063]
Thailand	[76, 75, 1061]
Tibet	[43, 177, 180, 181, 218, 277, 363, 410, 411, 412, 413, 414, 430, 480, 604, 695, 700, 750, 941, 939, 940, 951, 987, 985, 989, 992, 1012, 1056, 1054, 1058, 1060]
Vietnam	[33, 76, 469, 470, 471]
Wudalianchi	[82, 178, 188, 325, 478, 558, 603, 686, 785, 814, 1050, 1051, 1057]

Supplementary Table 2: References by region for Supplementary Database 1 of oceanic intraplate volcanism.

Oceanic Islands	References
Atlantic Ocean	
Azores	[90, 91, 92, 93, 207, 242, 316, 331, 381, 460, 461, 564, 565, 586, 642, 643, 691, 698, 722, 805, 952, 1008, 1041, 1042]
Canary Islands	[3, 6, 11, 21, 138, 153, 154, 252, 253, 259, 260, 361, 417, 472, 499, 534, 535, 553, 606, 607, 615, 647, 650, 654, 740, 798, 862, 933, 934, 953, 960]
Cape Verde	[34, 70, 71, 242, 286, 297, 301, 311, 320, 384, 462, 477, 505, 634, 635, 641, 656, 668, 697, 718, 719, 993]
Foundation	[552]
Gough	[387, 1011]
Iceland	[107, 125, 126, 164, 256, 304, 305, 339, 356, 415, 416, 432, 433, 434, 455, 503, 525, 530, 541, 542, 547, 557, 566, 630, 637, 638, 639, 636, 645, 648, 664, 673, 704, 736, 742, 780, 781, 795, 801, 819, 861, 876, 880, 881, 886, 888, 889, 890, 911, 969, 983, 1043]
Jan Mayen	[256, 627, 946]
Madiera	[377, 378, 661, 660, 864]
Meteor	[1001]
Pagalu	[579]
St. Helena	[162, 400, 523, 756, 1011]
Trinidade	[120, 538, 652, 878, 990]
Tristan Da Cunha	[209, 387, 458, 571, 1011]
Indian Ocean	
Amsterdam Island	[501]
Christmas	[921]
Comoros	[202, 204, 205, 206, 263, 784, 895]
Crozet	[131, 693]
Mascarene	[19, 20, 85, 118, 275, 336, 348, 590, 707, 749, 777, 778, 787, 856, 860, 874, 891, 971, 972, 973, 974, 975]
Pacific Ocean	
Austral Cook	[39, 105, 121, 151, 166, 239, 298, 299, 310, 334, 428, 429, 447, 451, 456, 568, 516, 665, 765, 905, 920, 927, 935, 1023]
Caroline	[278, 498, 662]
Cobb	[161, 268]
Easter	[119, 201, 349, 418, 419, 552, 815]
Galapagos	[123, 374, 373, 375, 376, 386, 427, 437, 438, 561, 732, 806, 854, 887, 897, 929, 1005]
Gerlach	[421]
Guadalupe	[83, 247, 544]
Hawaii	[98, 117, 156, 173, 174, 175, 172, 170, 199, 197, 198, 196, 200, 223, 226, 227, 249, 303, 330, 329, 341, 342, 351, 352, 359, 360, 366, 367, 368, 365, 369, 370, 371, 405, 444, 448, 445, 453, 454, 495, 522, 562, 581, 600, 628, 629, 646, 712, 708, 713, 710, 709, 721, 752, 753, 754, 807, 817, 821, 823, 822, 824, 832, 833, 896, 891, 908, 906, 919, 938, 937, 994, 981, 1003, 1002, 1010, 1009, 1021, 1020, 1026, 1034]
Juan Fernandez	[270, 327, 563, 820]
Marquesas	[80, 150, 152, 167, 220, 269, 406, 488, 539, 570, 585, 584, 595]
Pitcairn	[67, 149, 220, 257, 300, 310, 364, 403, 404, 452, 1024]
Pratt	[222]
Samoa	[440, 441, 446, 496, 497, 502, 545, 546, 765, 818, 1025]
Society Islands	[106, 111, 112, 113, 169, 179, 208, 299, 436, 447, 451, 456, 459, 583]
Southern Ocean	
Balleny	[401]
Peter I Island	[442, 529, 800]
Heard	[73, 74, 587, 910]
Kerguelan	[1000]
Marion	[572]

Supplementary Table 3: Database 1 with number of samples for major geographical regions. Filter 1 = > 6 MgO wt%, <= 20 Ma. Filter 2 = 9 < MgO < 14.5 wt%, age < 10 Ma, < 400 km from locus of eruption, La/Sm > 0.

Area	Filter 1	Filter 2	Filtered to remove samples affected by subduction
Africa	2347	446	No filtering
Anatolia	488	143	Only included samples < 10 Ma [679].
Antarctica	165	51	No filtering
Arabia	1258	104	No filtering
Australia	2471	91	No filtering
Brazil	71	42	No filtering
Central America	270	9	Removed samples according to conclusions of study.
China and Korea	1321	269	Removed samples according to conclusions of study.
Iran	87	33	No filtering
Lake Baikal	528	11	No filtering
Mongolia	228	46	Removed samples according to conclusions of study.
New Zealand	140	79	Removed samples according to conclusions of study.
North America	177	109	Included samples deemed to be intraplate by ref. [532].
Oceanic Islands	10820	1678	No filtering
Patagonia	242	59	Removed samples according to conclusions of study.
SE Asia	107	32	Removed samples according to conclusions of study.
Tibet	239	6	No filtering.
Western Europe	1445	277	Removed samples according to conclusions of study.

Supplementary Table 4: Shear-wave velocity to temperature conversion scheme.

Model	Hoggard *et al.* (2020)
V_s Equations	Yamauchi & Takei, (2016)
Tomo. Model	SL2013sv
Plate Model	Richards *et al.* (2018)
Depths H_1 (km)	75 + 100 + 125
Depths H_2 (km)	250–400
Depths H_3 (km)	150–400
ν_{ref} (Pa s)	3×10^{20}
Depths H_4 (km)	225–400
w_1	10
w_2	1
w_3	2
w_4	2
w_5	0
μ_U^0 (GPa)	76.3
$\frac{\partial \mu_U}{\partial T}$ (MPa °C^{-1})	−17.7
$\frac{\partial \mu_U}{\partial P}$ (MPa °C^{-1})	2.53
ν_r (Pa s)	1.23×10^{21}
E_a (kJ mol^{-1})	202
V_a (cm^3 mol^{-1})	0.92
$\frac{\partial T_s}{\partial z}$ (°C km^{-1})	0.919

Supplementary Table 5: Element compositions for mantle source regions. Primitive mantle source = PM; depleted mantle source = DMM where values are given in ppm [677]. H_2O in the source is calculated assuming that that $X^{bulk}_{H_2O}/X^{bulk}_{Ce} = 200$ [Methods; 694].

Source	εNd	La	Ce	Pr	Nd	Sm	Eu	Gd	Tb	Dy
PM	0	0.55	1.4	0.22	1.08	0.35	0.13	0.457	0.084	0.57
DMM	10	0.206	0.722	0.143	0.815	0.299	0.115	0.419	0.077	0.525

Source	εNd	Ho	Er	Tm	Yb	Lu	Ba	Nb	K	Zr	H_2O
PM	0	0.13	0.372	0.058	0.372	0.057	6.5	0.54	200	8.51	280
DMM	10	0.12	0.347	0.054	0.347	0.057	0.65	0.39	20	7.19	144.4

Supplementary Table 6: Mineral proportions in source region. Proportion by weight of each mineral = F^0_n.

	Olivine	Orthopyroxene	Clinopyroxene	Plagioclase	Spinel	Garnet
Plagioclase peridotite	0.636	0.263	0.012	0.089	–	–
Spinel peridotite	0.578	0.27	0.199	–	0.033	–
Garnet peridotite	0.598	0.211	0.076	–	–	0.115

Supplementary Table 7: Mineral compositions in source region by weight %. Molecular weight of each oxide quoted in final row.

Mineral	SiO_2	TiO_2	Al_2O_3	Cr_2O_3	FeO	MnO	MgO	CaO	Na_2O	K_2O	NiO
Olivine	40.84	0.02	0.04	0.04	8.72	0.50	49.53	0.06	0.00	0.00	0.24
Orthopyroxene	57.32	0.11	0.89	0.30	5.35	0.12	35.03	0.69	0.17	0.00	0.01
Clinopyroxene	54.76	0.21	2.59	1.63	2.96	0.10	17.36	18.34	2.04	0.01	0.0
Plagioclase	44.79	0.00	34.81	0.00	0.27	0.0	0.03	19.47	0.64	0.00	0.0
Spinel	0.00	0.13	65.55	0.00	11.94	0.10	22.28	0.00	0.00	0.00	0.0
Garnet	41.74	0.43	20.21	3.99	7.68	0.37	20.40	5.11	0.05	0.00	0.0
Molecular wt.	60.09	79.90	102.0	151.99	71.85	70.94	40.30	56.08	61.98	94.20	74.70

Supplementary Table 8: Element properties for invariant minerals.

Element	La	Ce	Pr	Nd	Sm	Eu	Gd	Tb	Dy
Ionic Radius (Å)	1.160	1.143	1.126	1.109	1.079	1.066	1.053	1.040	1.027
Valency	3	3	3	3	3	3	3	3	3
D olivine	0.0004	0.0005	0.0008	0.001	0.0013	0.0016	0.0015	0.0015	0.0017
D orthopyroxene	0.002	0.003	0.0048	0.0068	0.01	0.013	0.016	0.019	0.022
D spinel	0.01	0.01	0.01	0.01	0.01	0.01	0.01	0.01	0.01

Element	Ho	Er	Tm	Yb	Lu	Ba	Nb	K	Zr
Ionic Radius (Å)	1.015	1.004	0.994	0.985	0.977	1.42	0.74	1.51	0.84
Valency	3	3	3	3	3	2	5	1	4
D olivine	0.0016	0.0015	0.0015	0.0015	0.0015	0.003	0.005	0.00018	0.01
D orthopyroxene	0.026	0.03	0.04	0.049	0.060	0.0001	0.005	0.001	0.03
D clinopyroxene	–	–	–	–	–	0.0005	0.02	0.002	0.1
D spinel	0.01	0.01	0.01	0.01	0.01	0.001	0	0.0001	0
D garnet	–	–	–	–	–	0.0005	0.07	0.001	0.32

Supplementary Table 9: Minerals with variable partition coefficients. Constants used to calculate partition coefficients for varying pressure, P, and temperature, T, using Equation 16 in main text [1022]. PYR = fraction of pyrope

	$r^{v+}_{0(M)}$ (Å)	E^{v+}_M (GPa)	$D^{v+}_{0(M)}$
Clinopyroxene (M2)	$(0.974 + 0.067x^{M2}_{Ca} - 0.051x^{M1}_{Al})$	$(318.6 + 6.9P - 0.036T)$	$\frac{x^l_{Mg} exp(a)}{x^s_{Mg}}$
Plagioclase	1.234	150	0.20
Garnet	$(0.993 - 0.0628PYR)$	580	$(2.33 + 2.97PYR)$

$a = \frac{(88750-65.644T+7050P-770P^2)}{RT}$

Supplementary Table 10: Geochemical Temperature and Lithospheric Thickness Estimates. Best-fitting potential temperature, T_p, and lithospheric thickness, a, results for each 1° bin.

Region	Province	Bin Location	εNd	rms	T_p (°C)	$+T_p$	$-T_p$	a (km)	$+a$	$-a$
Australasia and Antarctica	Antarctica	167°W, 78°S	5.12	0.31	1250	6	0	63	0	1
The Americas	Patagonia	70°E, 52°S	5.24	0.71	1276	30	26	60	4	3
Southern Ocean	Marion Island	38°W, 47°S	5.74	0.29	1310	6	10	57	1	1
Southern Ocean	Crozet Island	52°W, 46°S	4.04	0.18	1295	3	4	58	0	0
The Americas	Patagonia	70°E, 45°S	0.67	0.29	1289	14	17	58	1	1
Australasia and Antarctica	Southern NSW	141°W, 38°S	0	0.51	1328	25	15	56	1	2
Australasia and Antarctica	Southern NSW	142°W, 38°S	5	0.28	1335	36	17	58	1	2
Australasia and Antarctica	Southern NSW	144°W, 38°S	5	0.24	1258	19	8	63	2	2
Australasia and Antarctica	New Zealand	175°W, 38°S	6.48	0.25	1330	32	31	58	2	2
Australasia and Antarctica	New Zealand	175°W, 37°S	6.07	0.17	1263	7	8	63	2	1
Pacific Ocean	Easter Island	111°E, 27°S	8.42	0.25	1369	10	8	30	6	0
Pacific Ocean	Pitcairn Island	129°E, 25°S	0	0.42	1317	21	12	53	1	2
Indian Ocean	Mascarene Islands	57°W, 20°S	4.79	0.42	1366	26	28	50	4	5
Indian Ocean	Mascarene Islands	58°W, 20°S	5.2	0.39	1421	45	25	45	5	11
Australasia and Antarctica	Northern Queensland	144°W, 20°S	5	0.24	1319	18	9	59	0	1
Australasia and Antarctica	Northern Queensland	146°W, 20°S	5	0.86	1356	11	17	55	1	1
Pacific Ocean	Austral Cook Islands	160°E, 19°S	5	0.67	1250	29	0	64	3	4
Australasia and Antarctica	Northern Queensland	145°W, 18°S	5.3	0.24	1315	17	15	58	1	1
Pacific Ocean	Society Islands	151°E, 17°S	5	0.74	1283	22	20	60	2	1
Pacific Ocean	Samoa	172°E, 14°S	1.19	0.39	1300	18	21	58	1	1
Indian Ocean	Comoros	45°W, 13°S	3.88	0.15	1263	12	7	61	1	1
Africa	Madagascar	49°W, 12°S	4.14	0.27	1319	36	33	55	3	4
Indian Ocean	Comoros	43°W, 11°S	2.87	0.47	1311	47	32	56	3	6
Indian Ocean	Marquesas	139°E, 10°S	4.01	0.62	1280	23	26	58	3	2
Indian Ocean	Marquesas	140°E, 9°S	3.59	0.75	1300	17	25	58	2	1
Africa	Rungwe	34°W, 9°S	5	0.55	1254	30	4	62	1	4
The Americas	Northeast Brazi	32°E, 4°S	3.54	0.49	1250	15	0	63	0	2
Africa	Southern Kenya	38°W, 3°S	3.36	0.64	1250	51	0	62	5	6
Africa	Tanganyika	29°W, 2°S	0	0.34	1251	11	1	59	1	1
Pacific Ocean	Galapagos	91°E, 1°S	6.51	0.27	1372	16	15	45	4	5
Pacific Ocean	Galapagos	90°E, 1°S	7.21	0.5	1380	25	19	38	8	8
Africa	Tanganyika	30°W, 1°S	0	0.89	1250	9	0	60	0	1
Pacific Ocean	Galapagos	92°E, 0°N	6.21	0.31	1366	15	12	47	3	5
Pacific Ocean	Galapagos	91°E, 0°N	8.06	0.16	1378	7	7	33	5	3
Africa	Tanganyika	30°W, 0°N	0	0.92	1259	9	9	76	3	3
Africa	Northern Kenya	38°W, 2°N	5.61	0.25	1299	21	21	58	2	2
Africa	Northern Kenya	37°W, 3°N	5.25	0.32	1312	21	19	55	2	3
Africa	Cameroon Line	10°W, 6°N	4.21	0.28	1273	21	10	60	1	2
Africa	Cameroon Line	14°W, 7°N	5	0.35	1259	7	9	62	2	1
Eastern Asia	Vietnam	107°W, 11°N	4.34	0.93	1250	1	0	61	0	0
Africa	Afar	43°W, 12°N	5.86	0.44	1442	57	45	30	10	0
Eastern Asia	Vietnam	108°W, 13°N	4.59	0.49	1310	45	27	59	2	3
Eastern Asia	Vietnam	108°W, 14°N	3.26	0.56	1298	30	34	59	2	2
Atlantic Ocean	Cape Verde	25°E, 15°N	5	0.15	1299	21	10	61	0	1
Atlantic Ocean	Cape Verde	24°E, 15°N	3.39	0.52	1255	29	5	62	2	3
Atlantic Ocean	Cape Verde	23°E, 16°N	5	0.55	1250	28	0	64	1	4
Atlantic Ocean	Cape Verde	25°E, 17°N	5.15	0.35	1250	9	0	63	1	1
Atlantic Ocean	Cape Verde	24°E, 17°N	6.5	0.26	1276	10	7	60	1	1
Pacific Ocean	Hawaii	156°E, 19°N	5.95	0.35	1389	10	9	45	3	3
Pacific Ocean	Hawaii	155°E, 19°N	5.9	0.24	1366	11	16	51	2	2
Pacific Ocean	Hawaii	156°E, 20°N	6.13	0.66	1386	40	33	53	4	6
Pacific Ocean	Hawaii	155°E, 20°N	6.14	0.36	1352	15	18	52	2	3
Pacific Ocean	Hawaii	158°E, 21°N	5.41	0.24	1273	36	23	61	4	3
Pacific Ocean	Hawaii	157°E, 21°N	6.55	0.18	1361	14	18	51	2	3
Pacific Ocean	Hawaii	156°E, 21°N	7.47	0.45	1341	25	16	55	2	3
Pacific Ocean	Hawaii	155°E, 21°N	5	0.39	1357	13	16	50	2	3
Pacific Ocean	Hawaii	159°E, 22°N	6.48	0.25	1330	22	12	52	2	4
Pacific Ocean	Hawaii	158°E, 22°N	5.61	0.37	1267	36	15	63	4	3
Pacific Ocean	Hawaii	157°E, 22°N	6.5	0.36	1375	12	10	46	3	3

Continued Overleaf...

Region	Province	Bin Location	εNd	rms	T_p (°C)	$+T_p$	$-T_p$	a (km)	$+a$	$-a$
Africa	Hoggar	5°W, 23°N	5	0.68	1250	16	0	63	2	2
Anatolia, Arabia and Iran	Harrat Al Kishb	41°W, 23°N	5	0.12	1260	9	7	60	1	2
Africa	Libya	19°W, 24°N	5	0.55	1250	18	0	63	1	3
Anatolia, Arabia and Iran	Harrat Rahat	40°W, 24°N	6.96	0.59	1409	30	24	47	5	8
Africa	Libya	17°W, 26°N	5	0.26	1333	26	27	58	2	1
Eastern Asia	Taiwan	117°W, 26°N	5	0.5	1250	16	0	65	2	3
Africa	Libya	17°W, 27°N	5.26	0.61	1383	33	30	54	3	4
Atlantic Ocean	Canary Islands	18°E, 28°N	5.37	0.28	1262	18	12	62	3	2
Atlantic Ocean	Canary Islands	17°E, 28°N	5.2	0.33	1279	18	19	60	2	1
Atlantic Ocean	Canary Islands	14°E, 28°N	5	0.38	1269	15	18	62	4	1
Africa	Libya	17°W, 28°N	3.89	0.38	1396	23	19	51	2	4
Africa	Libya	18°W, 28°N	5.18	0.28	1367	24	28	56	2	2
Atlantic Ocean	Canary Islands	18°E, 29°N	5.26	0.26	1254	12	4	63	1	2
Atlantic Ocean	Canary Islands	14°E, 29°N	2.28	0.37	1274	29	24	59	3	2
Eastern Asia	Nushan	121°W, 29°N	6.1	0.67	1315	22	22	59	1	2
Eastern Asia	Nushan	121°W, 30°N	4.35	0.74	1283	31	22	60	2	2
Eastern Asia	Nushan	119°W, 31°N	6.13	0.33	1289	24	21	61	2	1
North America	Rio Grande Rift	107°E, 32°N	6.04	0.46	1289	18	10	57	1	2
Eastern Asia	Nushan	119°E, 32°N	5	0.36	1305	17	24	58	2	1
Atlantic Ocean	Madiera	17°E, 33°N	8.16	0.1	1264	6	5	62	1	1
Africa	Atlas	5°E, 33°N	4.02	0.4	1251	13	1	62	0	2
Anatolia, Arabia and Iran	Harrat Ash Shaam	36°W, 33°N	4.41	0.19	1313	16	8	59	0	1
Eastern Asia	Nushan	118°W, 33°N	6.77	0.72	1259	15	4	68	1	4
Eastern Asia	Korea	126°W, 33°N	2.43	0.49	1330	21	11	56	1	2
Eastern Asia	Korea	127°W, 33°N	3.16	0.44	1314	14	9	56	1	2
The Americas	Western USA	112°E, 35°N	5	0.45	1288	27	20	59	2	2
Anatolia, Arabia and Iran	Dead Sea Region	36°W, 35°N	5.22	0.15	1275	12	10	61	1	1
The Americas	Western USA	113°E, 36°N	5	0.23	1279	11	11	60	1	1
Anatolia, Arabia and Iran	Euphrates	40°W, 36°N	5.17	0.43	1314	22	15	61	0	1
The Americas	Western USA	113°E, 37°N	2.38	0.26	1276	9	11	59	1	0
Europe	Mt. Etna	15°W, 37°N	7.1	0.48	1251	6	1	64	0	2
Arabia	Karasu	36°W, 37°N	5	0.42	1289	15	10	59	1	1
Anatolia, Arabia and Iran	Euphrates	40°W, 37°N	3.75	0.32	1341	24	14	57	1	1
Atlantic Ocean	Azores	28°E, 38°N	4.76	0.37	1308	10	6	56	1	1
Atlantic Ocean	Azores	26°E, 38°N	5.11	0.3	1285	14	9	59	1	1
Atlantic Ocean	Azores	25°E, 38°N	3.93	0.32	1278	17	10	59	1	1
Anatolia, Arabia and Iran	Central Anatolia	34°W, 38°N	2.33	0.84	1360	22	30	47	5	7
Anatolia, Arabia and Iran	Karacadag	39°W, 38°N	3.96	0.62	1401	62	32	53	4	8
Anatolia, Arabia and Iran	Karacadag	40°W, 38°N	4.42	0.27	1319	21	9	59	0	2
Eastern Asia	Shandong	121°W, 38°N	5	0.97	1250	0	0	67	0	0
Eastern Asia	Korea	127°W, 38°N	0	0.72	1352	14	13	48	3	3
Atlantic Ocean	Azores	28°E, 39°N	5.55	0.3	1308	7	10	56	1	1
Atlantic Ocean	Azores	27°E, 39°N	5.09	0.35	1309	11	11	55	1	1
Anatolia, Arabia and Iran	Central Anatolia	4°E, 39°N	5	0.58	1250	19	0	63	1	3
Atlantic Ocean	Azores	31°E, 40°N	5.5	0.44	1329	35	29	56	3	4
Anatolia, Arabia and Iran	Western Anatolia	26°W, 40°N	6.09	0.48	1282	13	17	60	2	1
Anatolia, Arabia and Iran	Western Anatolia	27°W, 41°N	6.13	0.47	1287	19	20	60	2	1
The Americas	Western USA	112°E, 43°N	0	0.45	1372	15	12	33	7	3
Eastern Asia	Dariganga	116°W, 43°N	4.71	0.58	1251	23	1	63	1	3
Europe	Massif Central	3°W, 44°N	5.25	0.43	1253	12	3	63	1	2
Eastern Asia	Dariganga	115°W, 44°N	4.63	0.66	1269	34	19	60	4	3
Eastern Asia	Changbaishan	129°W, 44°N	3.31	0.23	1283	17	14	60	1	1
Europe	Massif Central	3°W, 45°N	4.98	0.3	1262	9	11	61	2	1
Europe	Pannonian Basin	16°W, 47°N	4.06	0.74	1265	16	11	61	2	2
Eastern Asia	Hangai	100°W, 47°N	0	0.43	1346	10	10	51	2	1
Eastern Asia	Halaha	121°W, 47°N	5.8	0.64	1328	31	17	57	1	3
Eastern Asia	Hangai	100°W, 48°N	2.3	0.53	1293	19	14	60	0	1
Eastern Asia	Halaha	121°W, 48°N	3.99	0.32	1339	22	19	55	2	2
Eastern Asia	Wudalianchi	123°W, 49°N	0	0.72	1275	23	18	60	1	1
Europe	Bohemian Massif	17°W, 50°N	4.55	0.36	1255	17	5	63	2	2
Europe	Bohemian Massif	18°W, 50°N	4.56	0.42	1253	14	3	63	1	2
Eastern Asia	Wudalianchi	123°W, 50°N	0	0.75	1262	27	12	61	2	1
The Americas	Northern Cordillra	134°E, 60°N	6.59	0.26	1329	39	20	60	1	2
Europe	Iceland	20°E, 63°N	7.43	0.58	1375	16	14	42	5	7
Europe	Iceland	22°E, 64°N	8.35	0.31	1453	34	35	30	8	0
Europe	Iceland	21°E, 64°N	7.74	0.21	1452	19	26	30	8	0
Europe	Iceland	24°E, 65°N	6.23	0.73	1313	20	17	57	1	3
Europe	Iceland	23°E, 65°N	5.79	0.64	1344	47	33	54	4	7
Europe	Iceland	22°E, 65°N	5.85	0.46	1374	27	30	46	6	10
Europe	Iceland	20°E, 65°N	7.63	0.4	1549	0	36	30	10	0
Europe	Iceland	17°E, 65°N	7.64	0.4	1460	39	38	30	8	0
Europe	Iceland	16°E, 65°N	7.35	0.32	1428	42	25	38	9	8
Europe	Iceland	17°E, 66°N	8.65	0.64	1549	0	99	30	13	0

2 Supplementary Figures

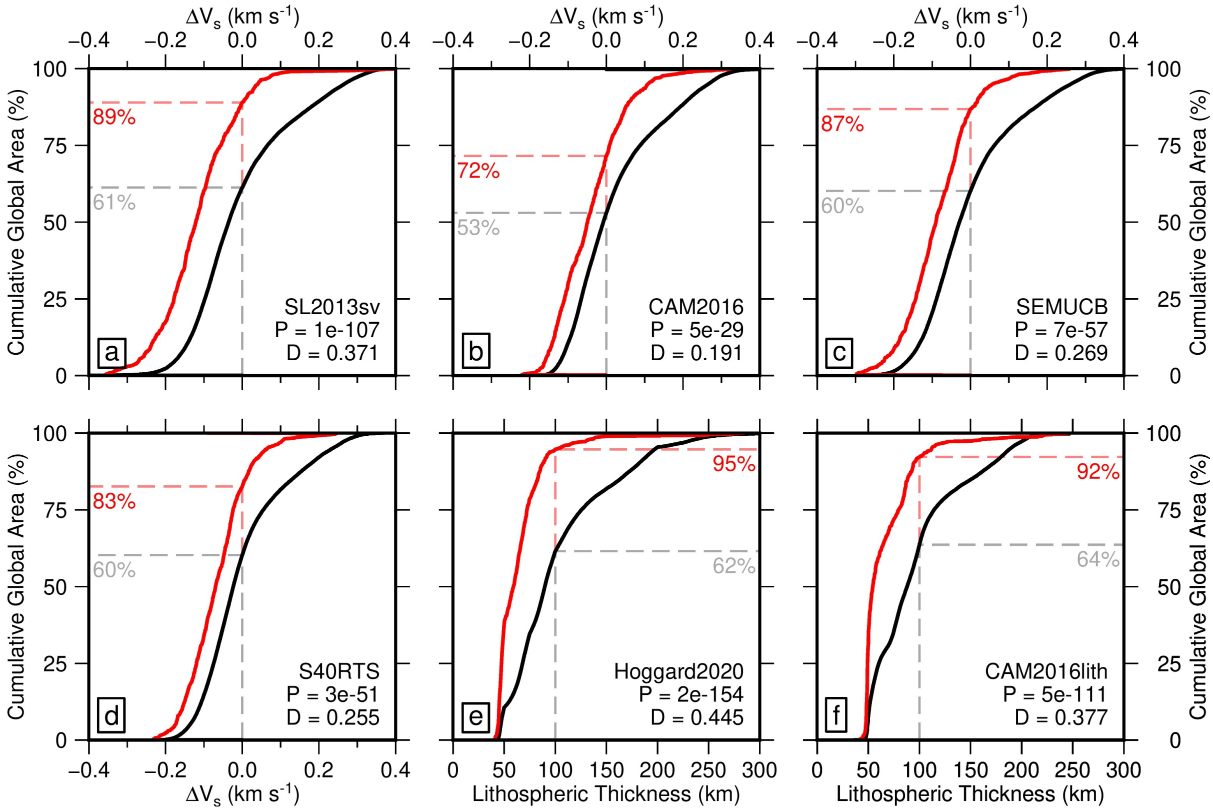

Supplementary Figure 1: Spatial correlation tests. (a) Black line = cumulative distribution of SL2013sv tomographic model averaged at depth of 150 ± 25 km (globe subdivided into 1° bins weighted by $\cos\phi$); red line = bins taken from global distribution that contain volcanic samples; dashed lines highlight percentage of Earth's surface where $\Delta V_s < 0$ km s^{-1} at depth of 150 ± 25 km; D = Kolmogorov Smirnov statistical measure; P = probability. (b) CAM2016-Vsv-200 model [468]. (c) SEMUCB-WM1 model [347]. (d) S40RTS model [830]. (e) Map showing lithospheric thickness variations [475]. Dashed lines highlight percentage of Earth's surface with lithosphere < 100 km thick. (f) Alternative map showing lithospheric thickness variations [804].

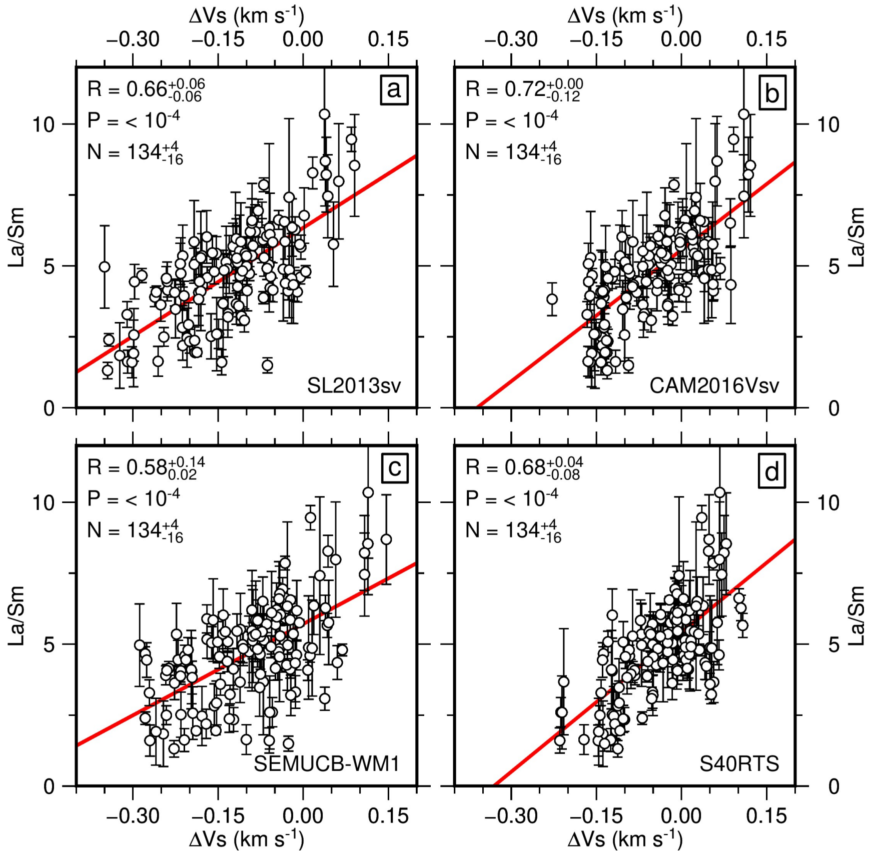

Supplementary Figure 2: Correlation between La/Sm and ΔV_s. (a) La/Sm plotted as function of average value of ΔV_s at depth of 150 ± 25 km for SL2013sv tomographic model. Circles and error bars = average value $\pm\sigma$ for each 1° bin weighted by $w = \cos\phi$; red line = best-fitting linear relationship with R value and its range; gray lines = suite of best-fitting relationships for 99 bin configurations spaced at intervals of 0.1°; N = number of bins used to calculate red line and to estimate range used for gray lines. Database 1 is filtered in accordance with $14.5 \geq$ MgO wt% ≥ 9, < 10 Ma, and number of samples in a given bin > 5. (b) CAM2016-Vsv-200 model. (c) SEMUCB-WM1 model. (d) S40RTS model.

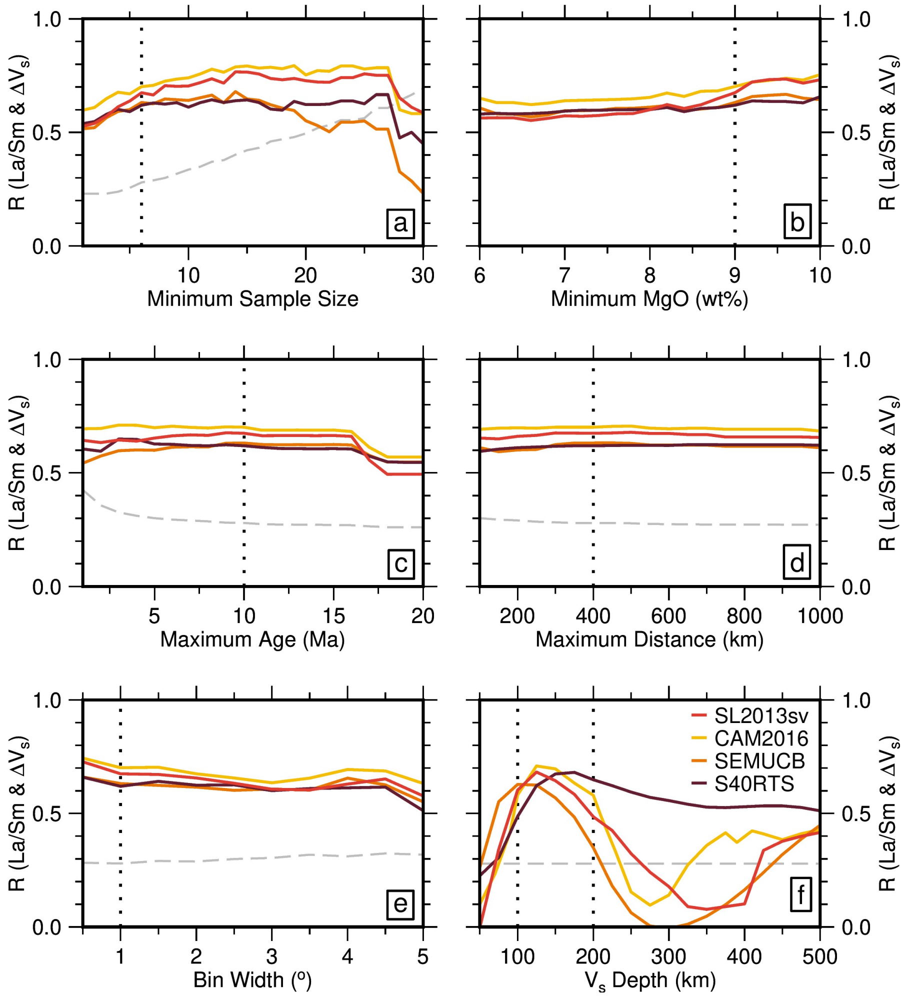

Supplementary Figure 3: Correlation between La/Sm and ΔV_s for different filtering parameters. (a) Value of R calculated between La/Sm and ΔV_s as function of minimum number of samples in 1° bins; colored lines = four different global tomographic models [SL2013sv, CAM2016Vsv, SEMUCB-WM1 and S40RTS; 859, 468, 347, 830]. Database 1 is subdivided into 1° bins weighted by $\cos\phi$; dotted black line = chosen value for correlation shown in Supplementary Figure 2; dashed gray line = value of R that can be resolved from zero at significance level = 0.001 given number of bins. (b) Value of R as function of minimum content of MgO (wt%) for each sample. (c) Value of R as function of maximum age of each sample. (d) Value of R as function of maximum distance from point of eruption based upon present-day plate speeds [41]. (e) Value of R as function of bin size. (f) value of R as function of depth.

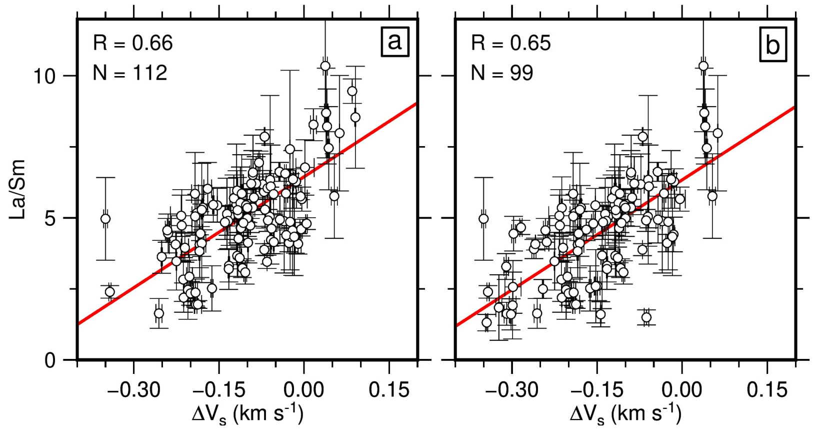

Supplementary Figure 4: Correlation that accounts for samples adjacent to rifting and subduction zones. (a) La/Sm plotted as function of ΔV_s. Circles and error bars = average values $\pm\sigma$ for samples in 1° bins; samples 9 > MgO > 14.5 wt%; number of samples in each bin > 5; samples with ages < 10 Ma excluding samples in Database 1 from Azores, Easter Island, Galapagos, Iceland and Jan Mayen; red line = best-fitting linear relationship where R = correlation coefficient and N = number of bins. (b) Database 1 excluding samples from Anatolia, America, Canada, China, Italy, Iran, Mexico, New Zealand, Patagonia and southern Spain.

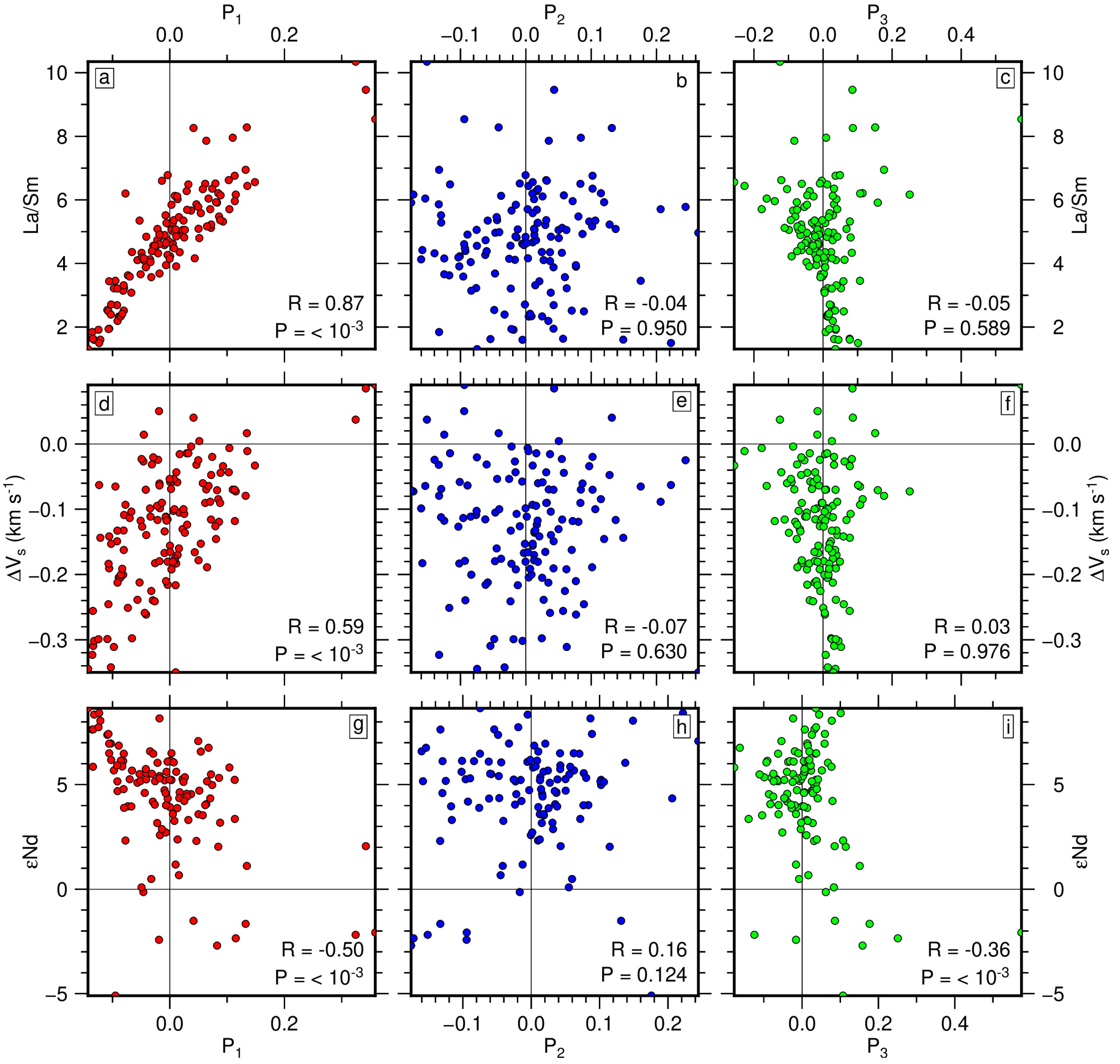

Supplementary Figure 5: Principal component analysis as function of geologic observations from Database 1. (a) La/Sm as function of first Principal Component, P_1, where correlation coefficient, R, is shown at bottom right-hand side. (b) La/Sm as function of P_2. (c) La/Sm as function of P_3. (d)–(f) ΔV_s as function of principal components. (g)–(i) ϵNd as function of principal components.

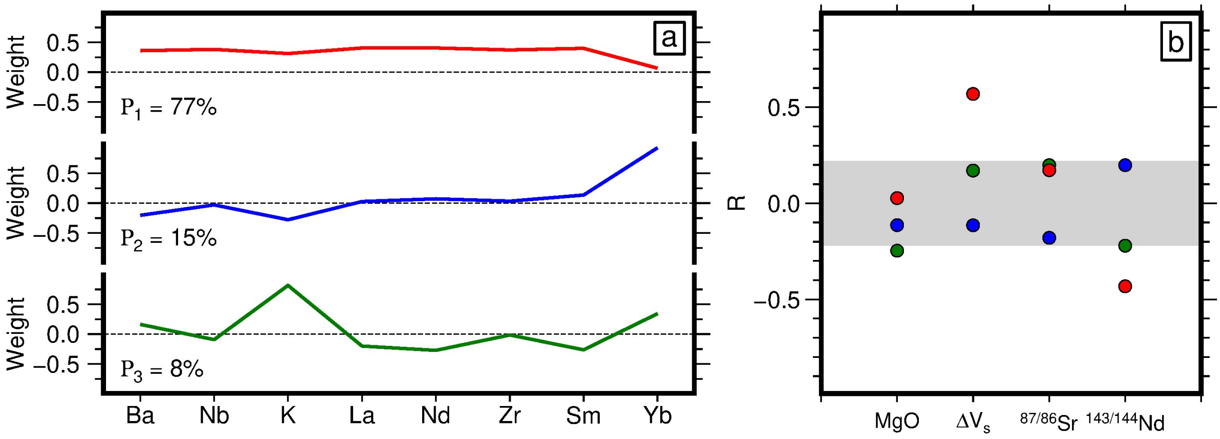

Supplementary Figure 6: Principal component analysis using un-binned data from Database 1. (a) Elemental weightings for each principal component. Prior to principal component analysis, Database 1 is filtered using 9 < MgO wt% < 14.5, < 10 Ma. Measurements are mean-centered and variance-scaled. Red/blue/green lines = proportion of variance for $P_1/P_2/P_3$. At bottom left-hand side, proportion of variance described by each component is given. (b) Correlation coefficient, R, calculated between each P and average value of ΔV_s between 100 and 200 km [859], MgO wt%, ^{87}Sr/^{86}Sr and ^{143}Nd/^{144}Nd.

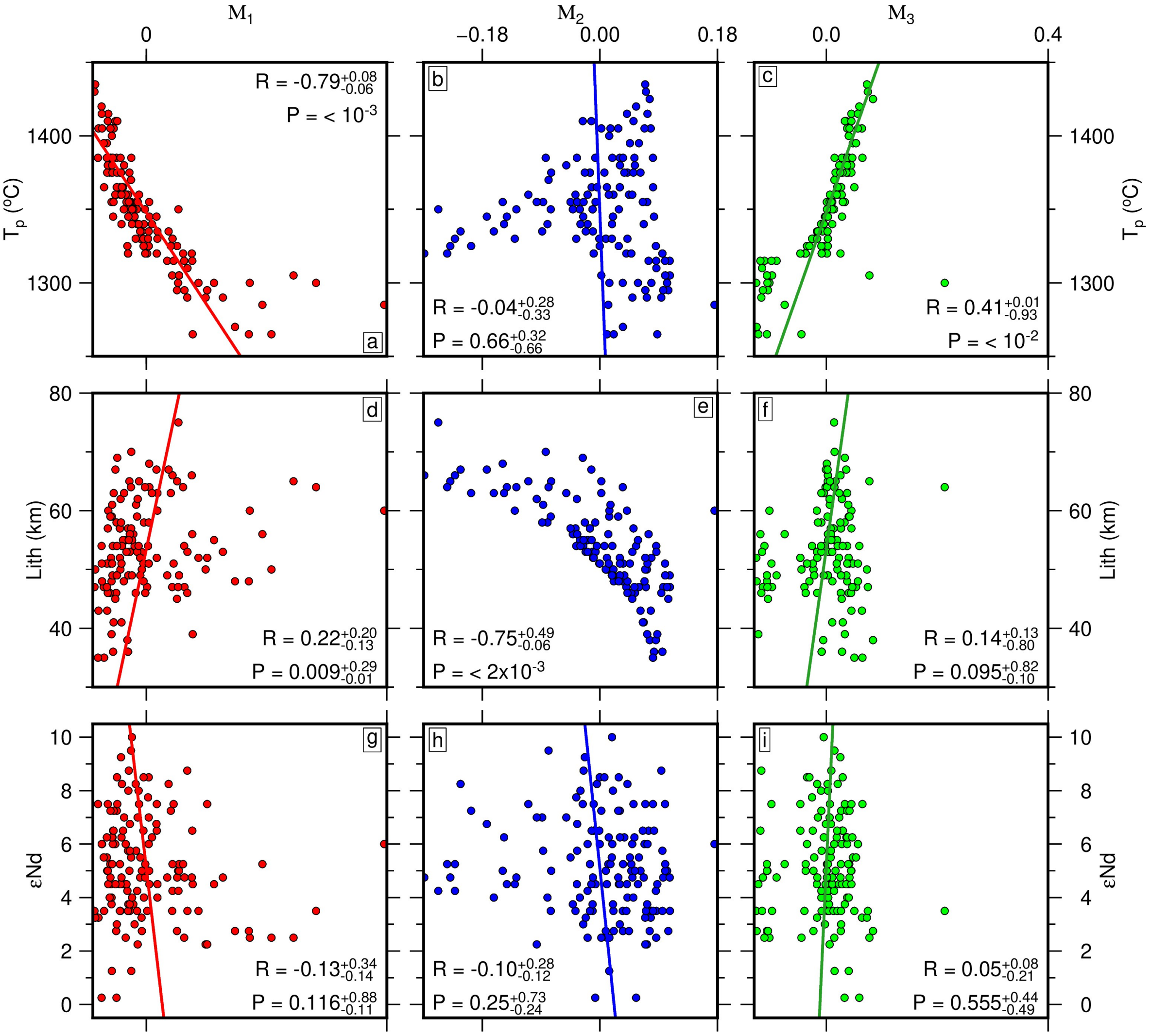

Supplementary Figure 7: Principal component analysis data generated using INVMEL model as function of model parameters. (a) T_p as function of first Principal Component, M_1, where correlation coefficient, R, is displayed at bottom right-hand side. Red line = best-fitting relationship. (b) T_p as function of M_2. (c) T_p as function of M_3. (d)–(f) Lithospheric thickness as function of principal components. (g)–(i) ϵNd as function of principal components.

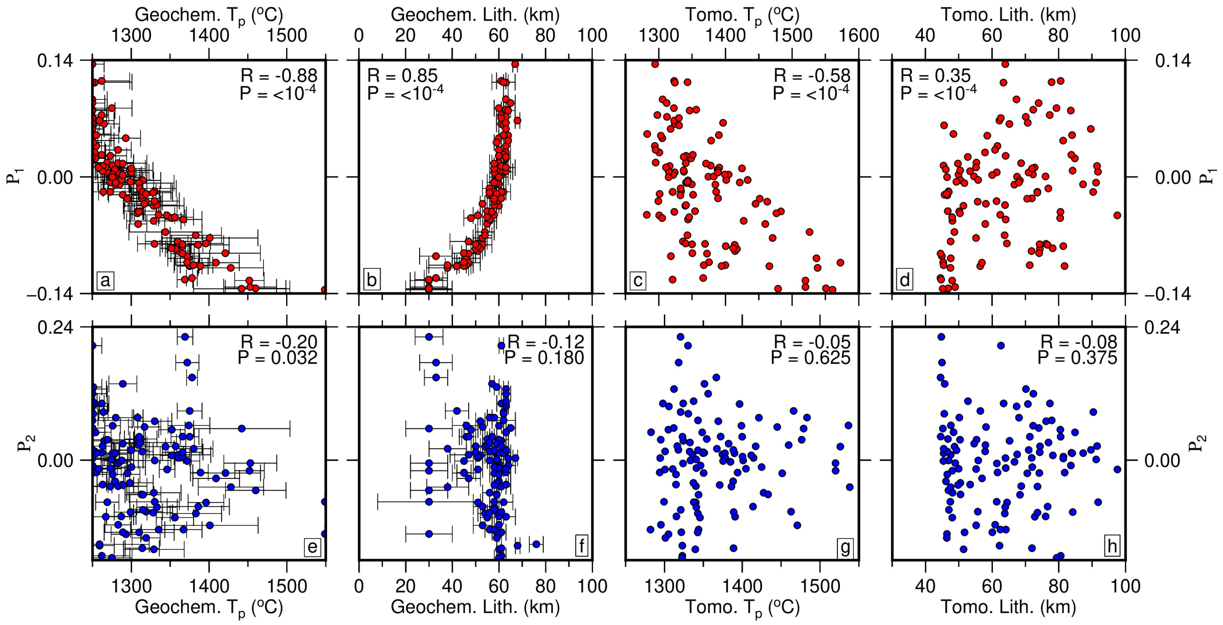

Supplementary Figure 8: Principal component analysis of Database 1 as function of temperature and lithospheric thickness estimates. (a) T_p estimated by geochemical inverse modeling of Database 1 as function of first Principal Component, P_1, where ratio of correlation and population correlation coefficients, R/P, are shown at top right-hand side. T_p error bars = $\pm 1.5\times$ minimum misfit. (b) Lithospheric thickness estimated using geochemical inversion scheme on Database 1 as function of P_1. (c) T_p estimated using V_s-to-T parameterization as function of First Principal Component, P_1. (d) Lithospheric thickness, calculated using V_s-to-T parameterization, as function of P_1. (e–h) Same for P_2.

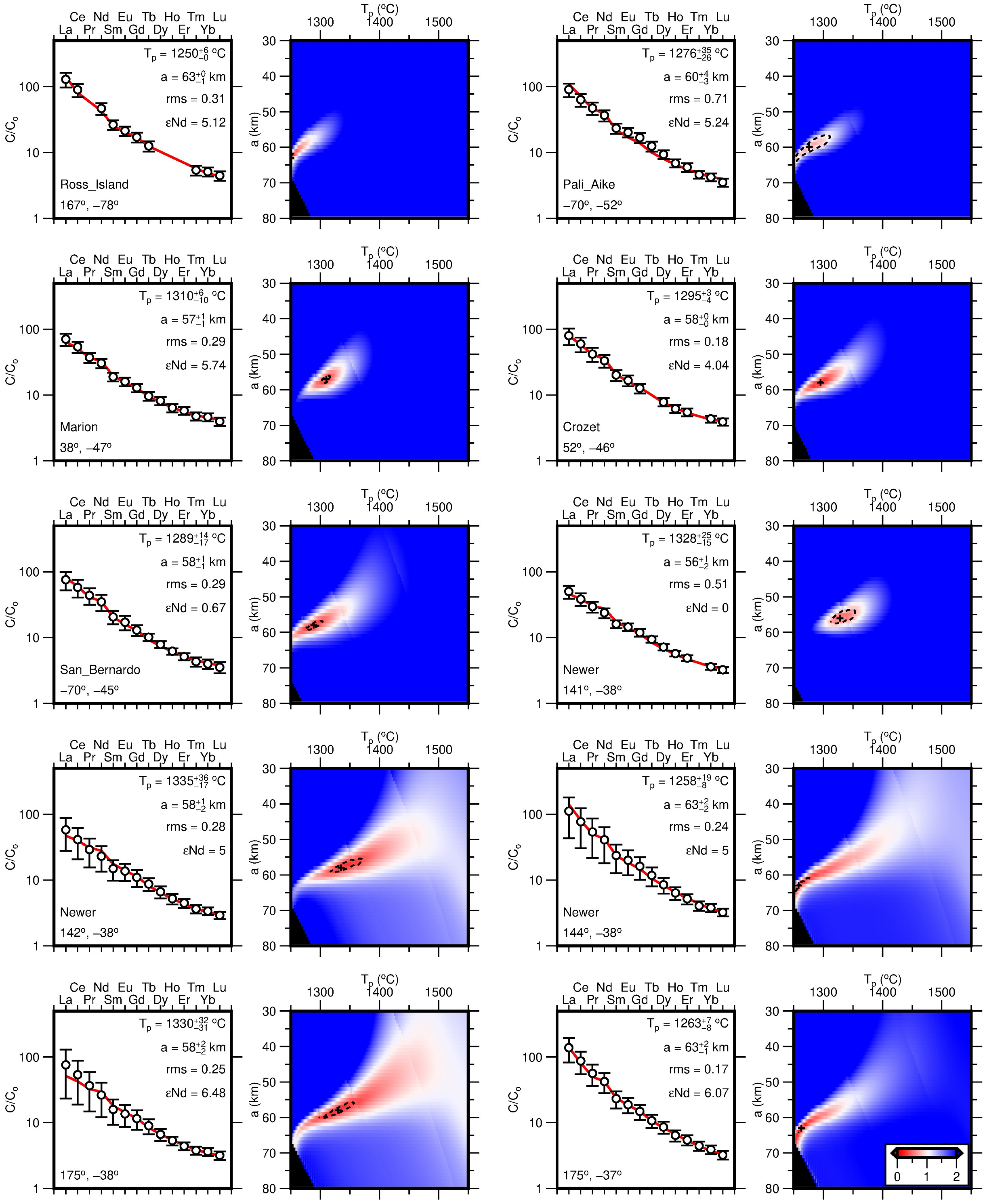

Supplementary Figure 9: Results of INVMEL modeling. Left-hand panel: REE concentrations, normalized with respect to source composition for 1° bins, shown at bottom left-hand side of panel. White circles with vertical bars = average concentrations $\pm\sigma$; red line = REE concentrations calculated for best-fitting model. Optimal values of potential temperature, T_p, $\pm1.25\times$ minimum misfit, lithospheric thickness, a, $\pm1.5\times$ minimum misfit; value of misfit at global minimum and value of ϵNd shown at top right-hand side of panel. Right-hand panel: RMS misfit function between observed and calculated REE concentrations plotted as function of T_p and a. Black cross = locus of global minimum; black dashed line = region where rms misfit $< 1.5\times$ misfit at global minimum..

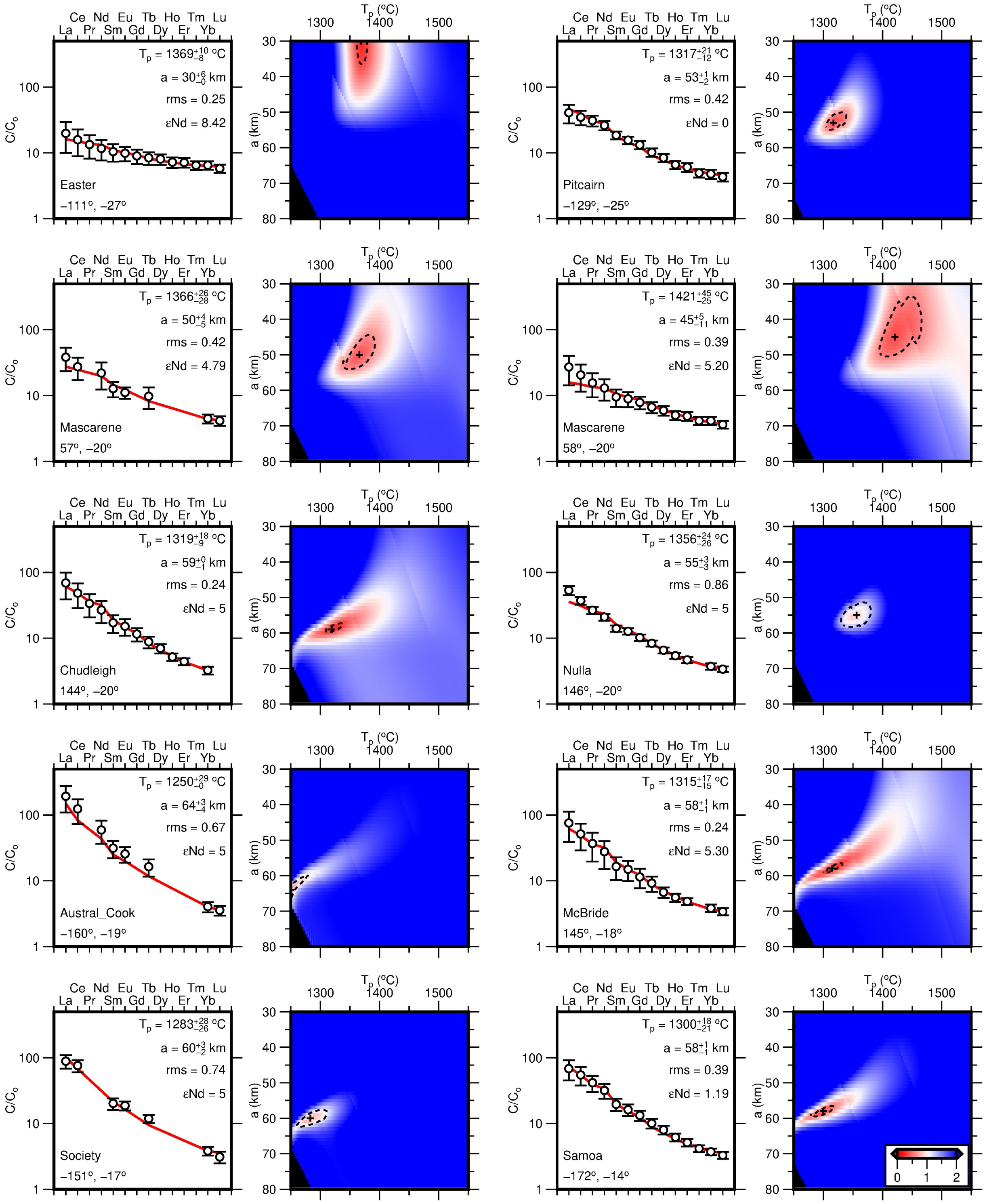

Ce Nd Eu Tb Ho Tm Lu
La Pr Sm Gd Dy Er Yb
C/Co
a (km)
Tp (ºC)
Tp = 1369+10−8 ºC
a = 30+6−0 km
rms = 0.25
εNd = 8.42
Easter
−111º, −27º
Tp = 1317+21−12 ºC
a = 53+1−2 km
rms = 0.42
εNd = 0
Pitcairn
−129º, −25º
Tp = 1366+26−28 ºC
a = 50+4−5 km
rms = 0.42
εNd = 4.79
Mascarene
57º, −20º
Tp = 1421+45−25 ºC
a = 45+5−11 km
rms = 0.39
εNd = 5.20
Mascarene
58º, −20º
Tp = 1319+18−9 ºC
a = 59+0−1 km
rms = 0.24
εNd = 5
Chudleigh
144º, −20º
Tp = 1356+24−26 ºC
a = 55+3−3 km
rms = 0.86
εNd = 5
Nulla
146º, −20º
Tp = 1250+29−0 ºC
a = 64+3−4 km
rms = 0.67
εNd = 5
Austral_Cook
−160º, −19º
Tp = 1315+17−15 ºC
a = 58+1−1 km
rms = 0.24
εNd = 5.30
McBride
145º, −18º
Tp = 1283+28−26 ºC
a = 60+3−2 km
rms = 0.74
εNd = 5
Society
−151º, −17º
Tp = 1300+18−21 ºC
a = 58+1−1 km
rms = 0.39
εNd = 1.19
Samoa
−172º, −14º

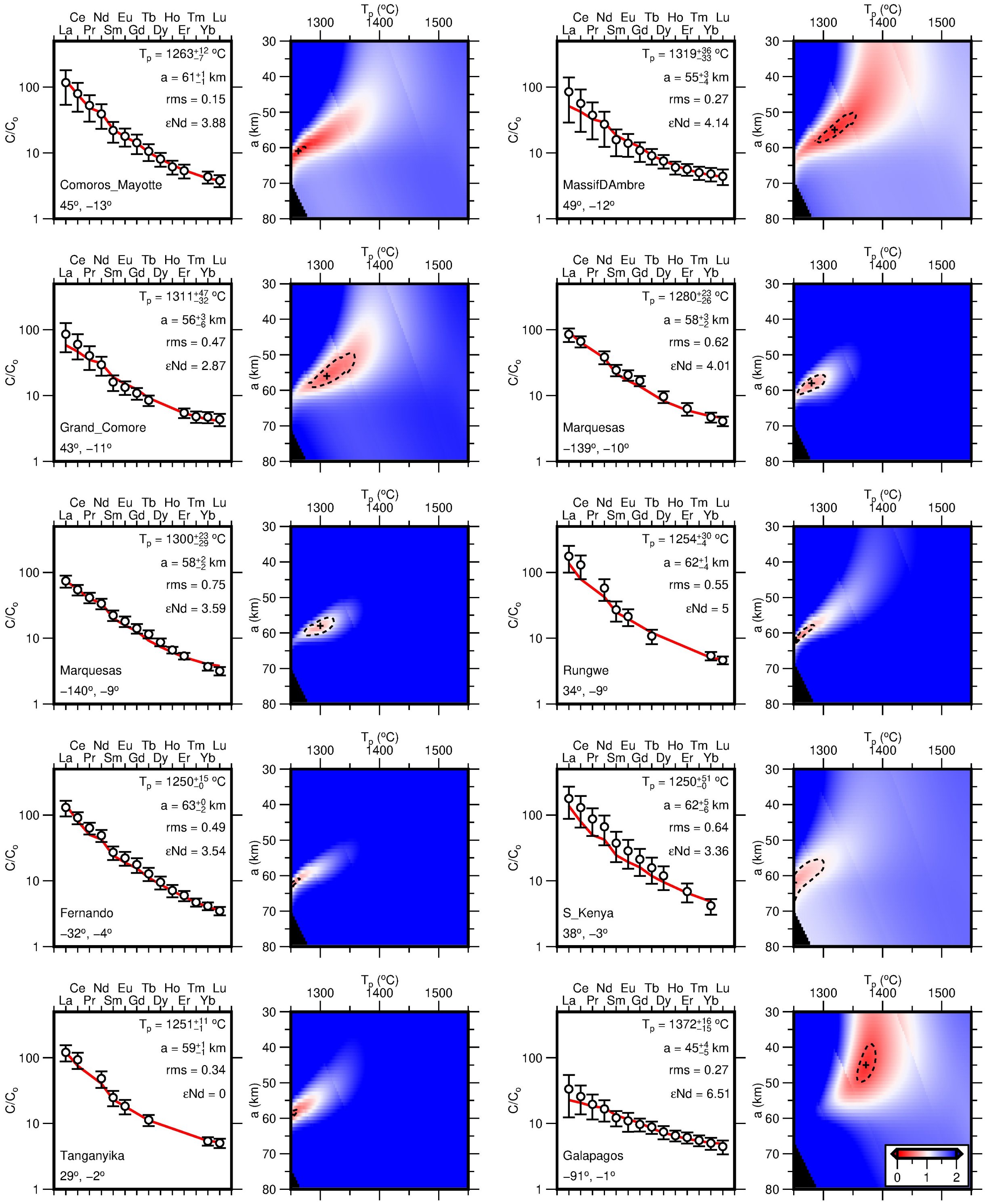
La Ce Pr Nd Sm Eu Gd Tb Dy Ho Er Tm Yb Lu
C/C$_o$
a (km)
T$_p$ (°C)
1300 1400 1500
T$_p$ = 1263$^{+12}_{-7}$ °C
a = 61$^{+1}_{-1}$ km
rms = 0.15
εNd = 3.88
Comoros_Mayotte
45°, −13°
T$_p$ = 1319$^{+36}_{-33}$ °C
a = 55$^{+3}_{-4}$ km
rms = 0.27
εNd = 4.14
MassifDAmbre
49°, −12°
T$_p$ = 1311$^{+47}_{-32}$ °C
a = 56$^{+3}_{-6}$ km
rms = 0.47
εNd = 2.87
Grand_Comore
43°, −11°
T$_p$ = 1280$^{+23}_{-26}$ °C
a = 58$^{+3}_{-2}$ km
rms = 0.62
εNd = 4.01
Marquesas
−139°, −10°
T$_p$ = 1300$^{+23}_{-29}$ °C
a = 58$^{+2}_{-2}$ km
rms = 0.75
εNd = 3.59
Marquesas
−140°, −9°
T$_p$ = 1254$^{+30}_{-4}$ °C
a = 62$^{+1}_{-4}$ km
rms = 0.55
εNd = 5
Rungwe
34°, −9°
T$_p$ = 1250$^{+15}_{-0}$ °C
a = 63$^{+0}_{-2}$ km
rms = 0.49
εNd = 3.54
Fernando
−32°, −4°
T$_p$ = 1250$^{+51}_{-0}$ °C
a = 62$^{+5}_{-6}$ km
rms = 0.64
εNd = 3.36
S_Kenya
38°, −3°
T$_p$ = 1251$^{+11}_{-1}$ °C
a = 59$^{+1}_{-1}$ km
rms = 0.34
εNd = 0
Tanganyika
29°, −2°
T$_p$ = 1372$^{+16}_{-15}$ °C
a = 45$^{+4}_{-5}$ km
rms = 0.27
εNd = 6.51
Galapagos
−91°, −1°
0 1 2

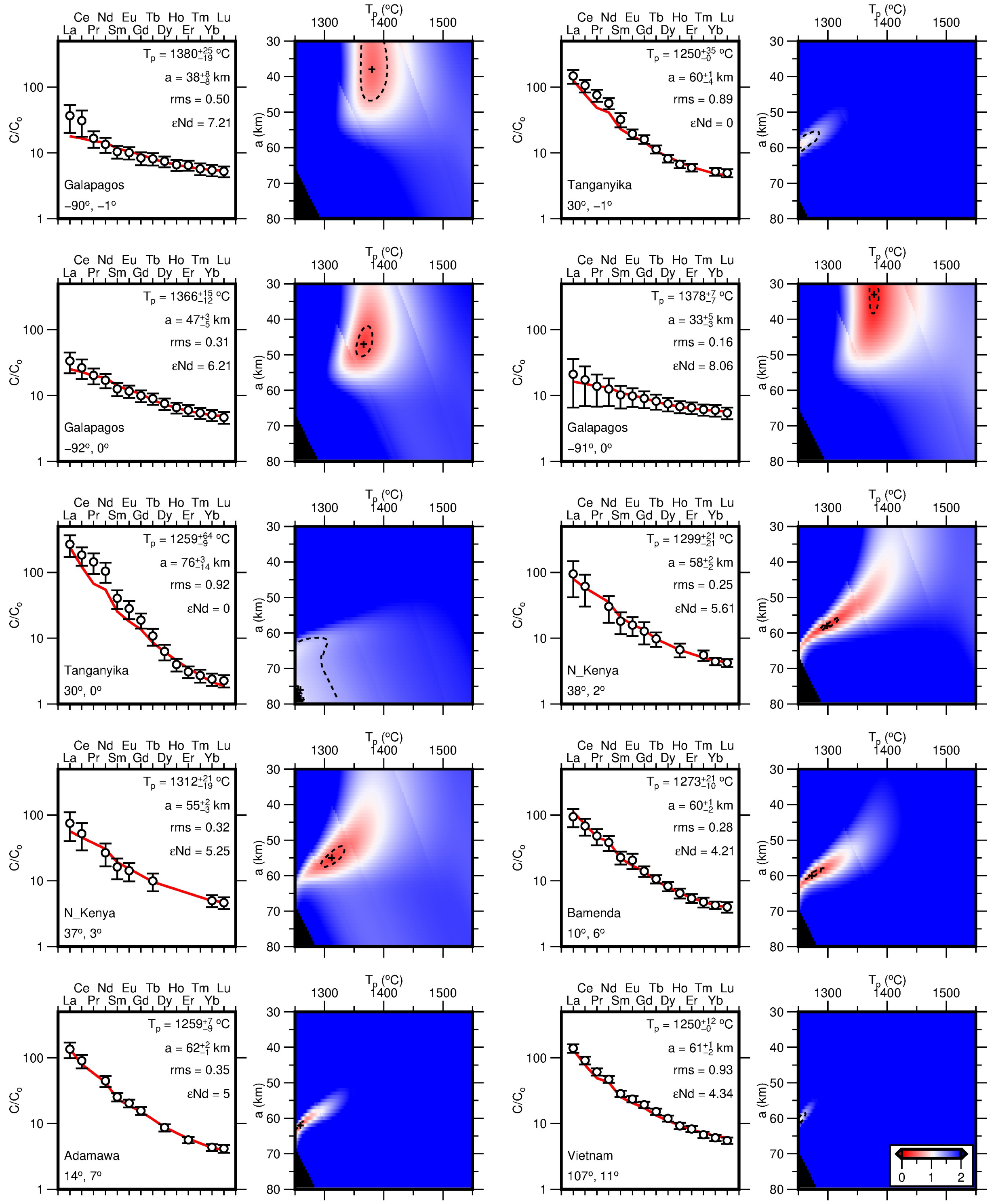
La Ce Pr Nd Sm Eu Gd Tb Dy Ho Er Tm Yb Lu
C/C$_o$
T_p (ºC)
a (km)
Galapagos
–90º, –1º
$T_p = 1380^{+25}_{-19}$ ºC
$a = 38^{+8}_{-8}$ km
rms = 0.50
εNd = 7.21
Tanganyika
30º, –1º
$T_p = 1250^{+35}_{-0}$ ºC
$a = 60^{+1}_{-4}$ km
rms = 0.89
εNd = 0
Galapagos
–92º, 0º
$T_p = 1366^{+15}_{-12}$ ºC
$a = 47^{+3}_{-5}$ km
rms = 0.31
εNd = 6.21
Galapagos
–91º, 0º
$T_p = 1378^{+7}_{-7}$ ºC
$a = 33^{+5}_{-3}$ km
rms = 0.16
εNd = 8.06
Tanganyika
30º, 0º
$T_p = 1259^{+64}_{-9}$ ºC
$a = 76^{+3}_{-14}$ km
rms = 0.92
εNd = 0
N_Kenya
38º, 2º
$T_p = 1299^{+21}_{-21}$ ºC
$a = 58^{+2}_{-2}$ km
rms = 0.25
εNd = 5.61
N_Kenya
37º, 3º
$T_p = 1312^{+21}_{-19}$ ºC
$a = 55^{+2}_{-3}$ km
rms = 0.32
εNd = 5.25
Bamenda
10º, 6º
$T_p = 1273^{+21}_{-10}$ ºC
$a = 60^{+1}_{-2}$ km
rms = 0.28
εNd = 4.21
Adamawa
14º, 7º
$T_p = 1259^{+7}_{-9}$ ºC
$a = 62^{+2}_{-1}$ km
rms = 0.35
εNd = 5
Vietnam
107º, 11º
$T_p = 1250^{+12}_{-0}$ ºC
$a = 61^{+1}_{-2}$ km
rms = 0.93
εNd = 4.34
0 1 2

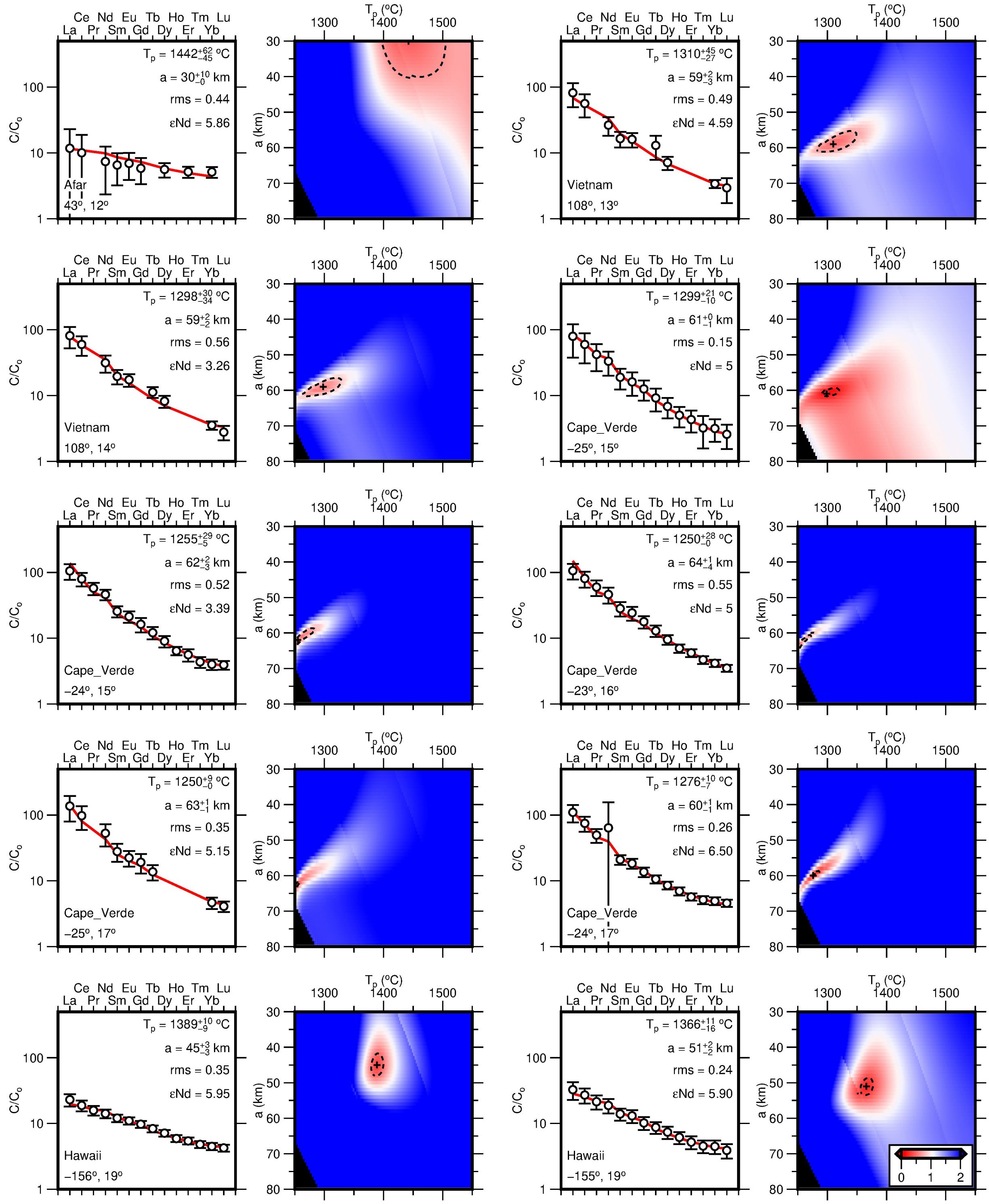
La Ce Pr Nd Sm Eu Gd Tb Dy Ho Er Tm Yb Lu
C/Co
a (km)
Tp (ºC)
Tp = 1442+62−45 ºC
a = 30+10−0 km
rms = 0.44
εNd = 5.86
Afar
43º, 12º
Tp = 1310+45−27 ºC
a = 59+2−3 km
rms = 0.49
εNd = 4.59
Vietnam
108º, 13º
Tp = 1298+30−34 ºC
a = 59+2−2 km
rms = 0.56
εNd = 3.26
Vietnam
108º, 14º
Tp = 1299+21−10 ºC
a = 61+0−1 km
rms = 0.15
εNd = 5
Cape_Verde
−25º, 15º
Tp = 1255+29−5 ºC
a = 62+2−3 km
rms = 0.52
εNd = 3.39
Cape_Verde
−24º, 15º
Tp = 1250+28−0 ºC
a = 64+1−4 km
rms = 0.55
εNd = 5
Cape_Verde
−23º, 16º
Tp = 1250+9−0 ºC
a = 63+1−1 km
rms = 0.35
εNd = 5.15
Cape_Verde
−25º, 17º
Tp = 1276+10−7 ºC
a = 60+1−1 km
rms = 0.26
εNd = 6.50
Cape_Verde
−24º, 17º
Tp = 1389+10−9 ºC
a = 45+3−3 km
rms = 0.35
εNd = 5.95
Hawaii
−156º, 19º
Tp = 1366+11−16 ºC
a = 51+2−2 km
rms = 0.24
εNd = 5.90
Hawaii
−155º, 19º

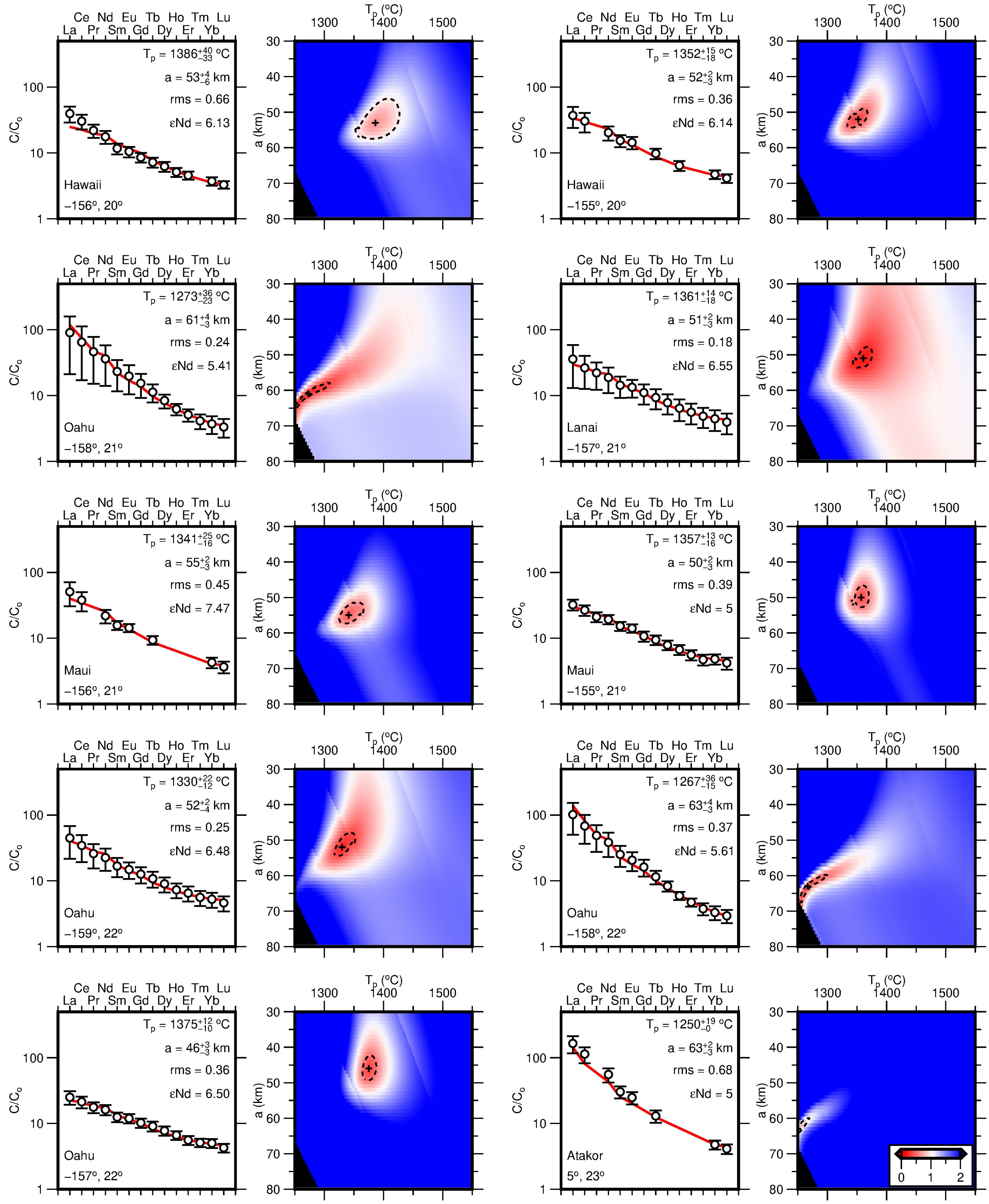

La Ce Pr Nd Sm Eu Gd Tb Dy Ho Er Tm Yb Lu
C/C$_o$
T_p (°C)
a (km)
$T_p = 1386^{+40}_{-33}$ °C
$a = 53^{+4}_{-6}$ km
rms = 0.66
εNd = 6.13
Hawaii
−156°, 20°
$T_p = 1352^{+15}_{-18}$ °C
$a = 52^{+2}_{-3}$ km
rms = 0.36
εNd = 6.14
Hawaii
−155°, 20°
$T_p = 1273^{+36}_{-23}$ °C
$a = 61^{+4}_{-3}$ km
rms = 0.24
εNd = 5.41
Oahu
−158°, 21°
$T_p = 1361^{+14}_{-18}$ °C
$a = 51^{+2}_{-3}$ km
rms = 0.18
εNd = 6.55
Lanai
−157°, 21°
$T_p = 1341^{+25}_{-16}$ °C
$a = 55^{+2}_{-3}$ km
rms = 0.45
εNd = 7.47
Maui
−156°, 21°
$T_p = 1357^{+13}_{-16}$ °C
$a = 50^{+2}_{-3}$ km
rms = 0.39
εNd = 5
Maui
−155°, 21°
$T_p = 1330^{+22}_{-12}$ °C
$a = 52^{+2}_{-4}$ km
rms = 0.25
εNd = 6.48
Oahu
−159°, 22°
$T_p = 1267^{+36}_{-15}$ °C
$a = 63^{+4}_{-3}$ km
rms = 0.37
εNd = 5.61
Oahu
−158°, 22°
$T_p = 1375^{+12}_{-10}$ °C
$a = 46^{+3}_{-3}$ km
rms = 0.36
εNd = 6.50
Oahu
−157°, 22°
$T_p = 1250^{+19}_{-0}$ °C
$a = 63^{+2}_{-3}$ km
rms = 0.68
εNd = 5
Atakor
5°, 23°

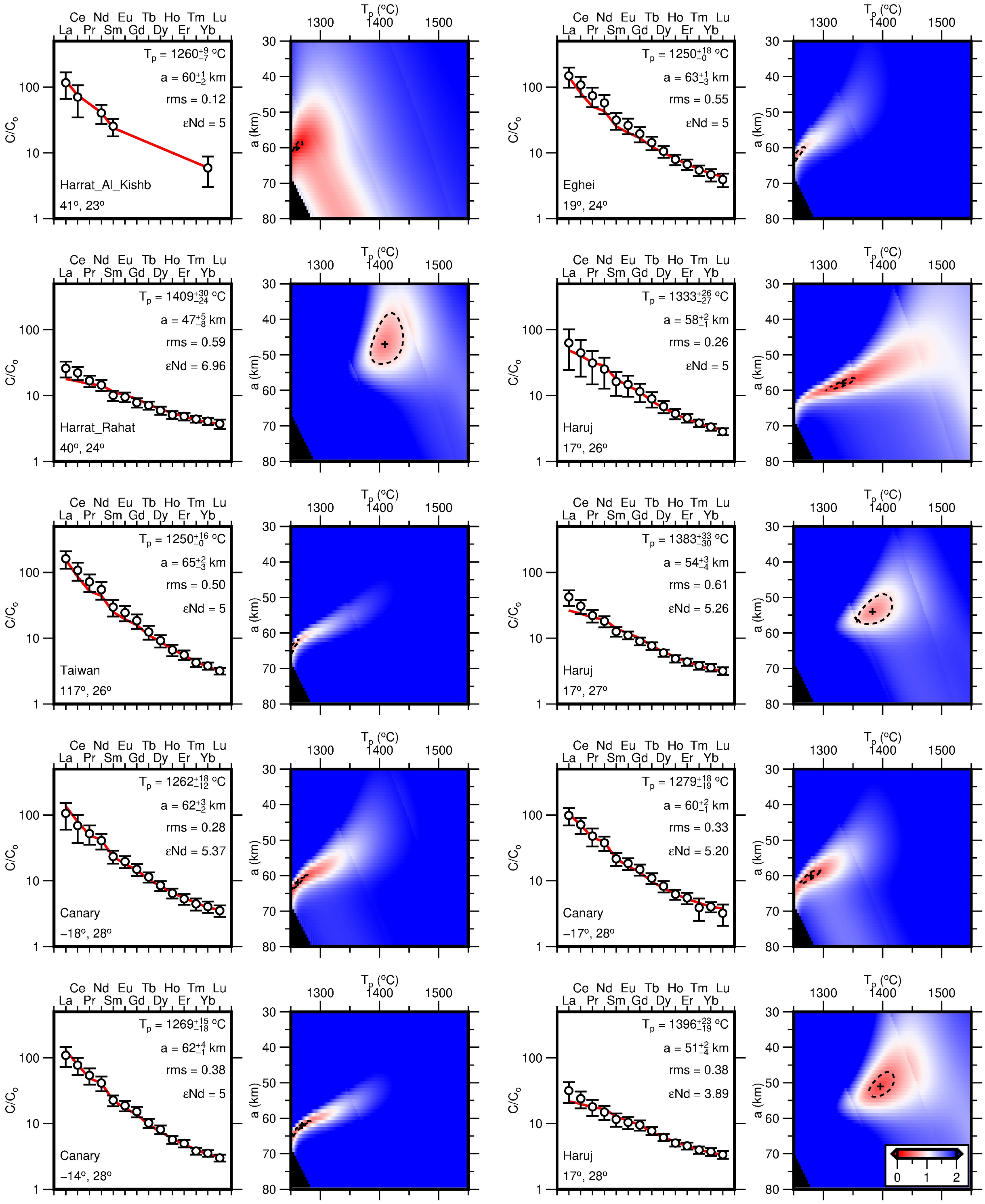

La Ce Pr Nd Sm Eu Gd Tb Dy Ho Er Tm Yb Lu
C/C$_o$
T_p (°C)
a (km)
$T_p = 1260^{+9}_{-7}$ °C
a = 60^{+1}_{-2} km
rms = 0.12
εNd = 5
Harrat_Al_Kishb
41°, 23°
$T_p = 1250^{+18}_{-0}$ °C
a = 63^{+1}_{-3} km
rms = 0.55
εNd = 5
Eghei
19°, 24°
$T_p = 1409^{+30}_{-24}$ °C
a = 47^{+5}_{-8} km
rms = 0.59
εNd = 6.96
Harrat_Rahat
40°, 24°
$T_p = 1333^{+26}_{-27}$ °C
a = 58^{+2}_{-1} km
rms = 0.26
εNd = 5
Haruj
17°, 26°
$T_p = 1250^{+16}_{-0}$ °C
a = 65^{+2}_{-3} km
rms = 0.50
εNd = 5
Taiwan
117°, 26°
$T_p = 1383^{+33}_{-30}$ °C
a = 54^{+3}_{-4} km
rms = 0.61
εNd = 5.26
Haruj
17°, 27°
$T_p = 1262^{+18}_{-12}$ °C
a = 62^{+3}_{-2} km
rms = 0.28
εNd = 5.37
Canary
−18°, 28°
$T_p = 1279^{+18}_{-19}$ °C
a = 60^{+2}_{-1} km
rms = 0.33
εNd = 5.20
Canary
−17°, 28°
$T_p = 1269^{+15}_{-18}$ °C
a = 62^{+4}_{-1} km
rms = 0.38
εNd = 5
Canary
−14°, 28°
$T_p = 1396^{+23}_{-19}$ °C
a = 51^{+2}_{-4} km
rms = 0.38
εNd = 3.89
Haruj
17°, 28°

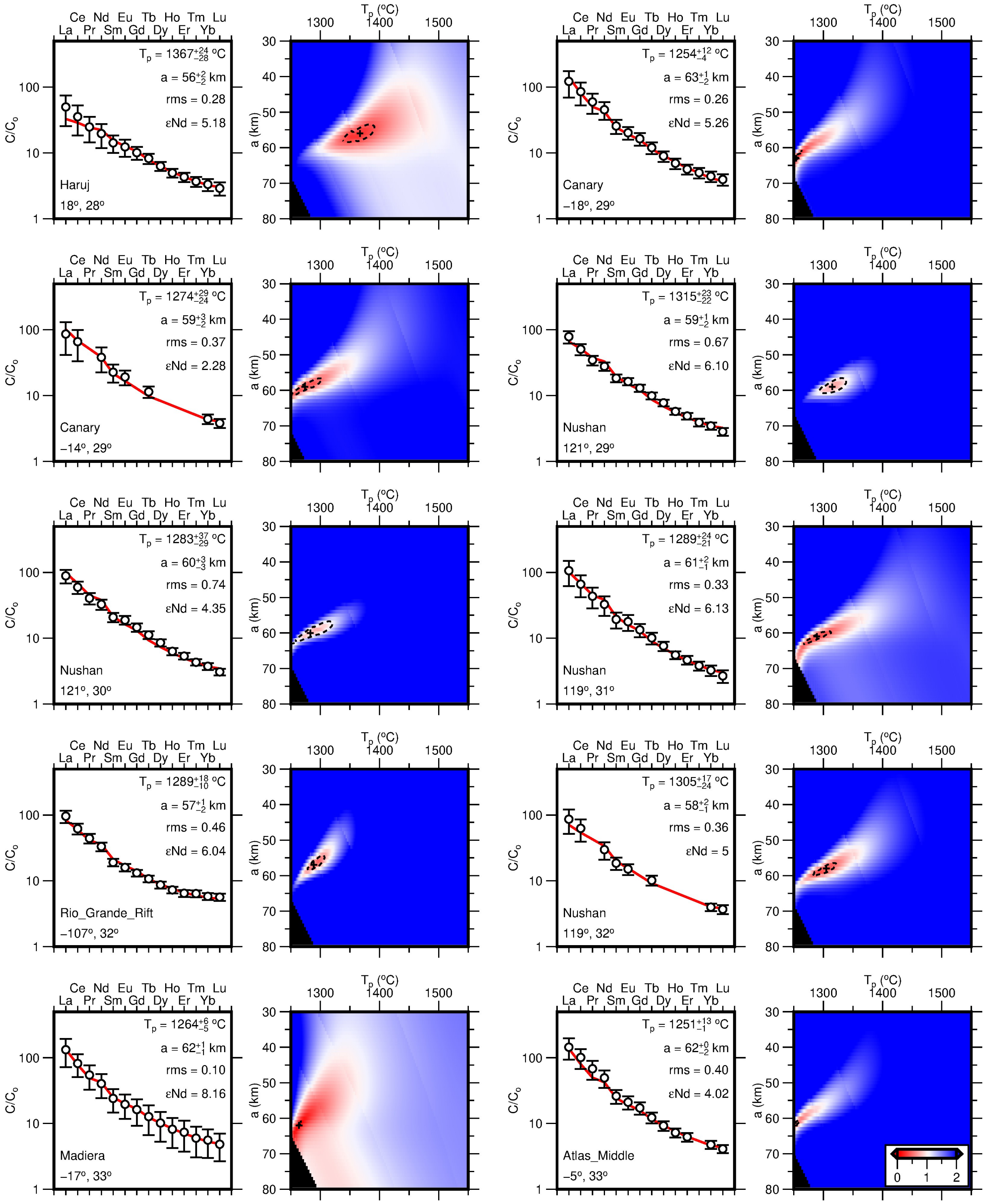

Tp = 1367 +24 −28 °C
a = 56 +2 −2 km
rms = 0.28
εNd = 5.18
Haruj
18°, 28°
Tp = 1254 +12 −4 °C
a = 63 +1 −2 km
rms = 0.26
εNd = 5.26
Canary
−18°, 29°
Tp = 1274 +29 −24 °C
a = 59 +3 −2 km
rms = 0.37
εNd = 2.28
Canary
−14°, 29°
Tp = 1315 +23 −22 °C
a = 59 +1 −2 km
rms = 0.67
εNd = 6.10
Nushan
121°, 29°
Tp = 1283 +37 −29 °C
a = 60 +3 −3 km
rms = 0.74
εNd = 4.35
Nushan
121°, 30°
Tp = 1289 +24 −21 °C
a = 61 +2 −1 km
rms = 0.33
εNd = 6.13
Nushan
119°, 31°
Tp = 1289 +18 −10 °C
a = 57 +1 −2 km
rms = 0.46
εNd = 6.04
Rio_Grande_Rift
−107°, 32°
Tp = 1305 +17 −24 °C
a = 58 +2 −1 km
rms = 0.36
εNd = 5
Nushan
119°, 32°
Tp = 1264 +6 −5 °C
a = 62 +1 −1 km
rms = 0.10
εNd = 8.16
Madiera
−17°, 33°
Tp = 1251 +13 −1 °C
a = 62 +0 −2 km
rms = 0.40
εNd = 4.02
Atlas_Middle
−5°, 33°
La Ce Pr Nd Sm Eu Gd Tb Dy Ho Er Tm Yb Lu
C/Co
a (km)
Tp (°C)
1300 1400 1500
0 1 2

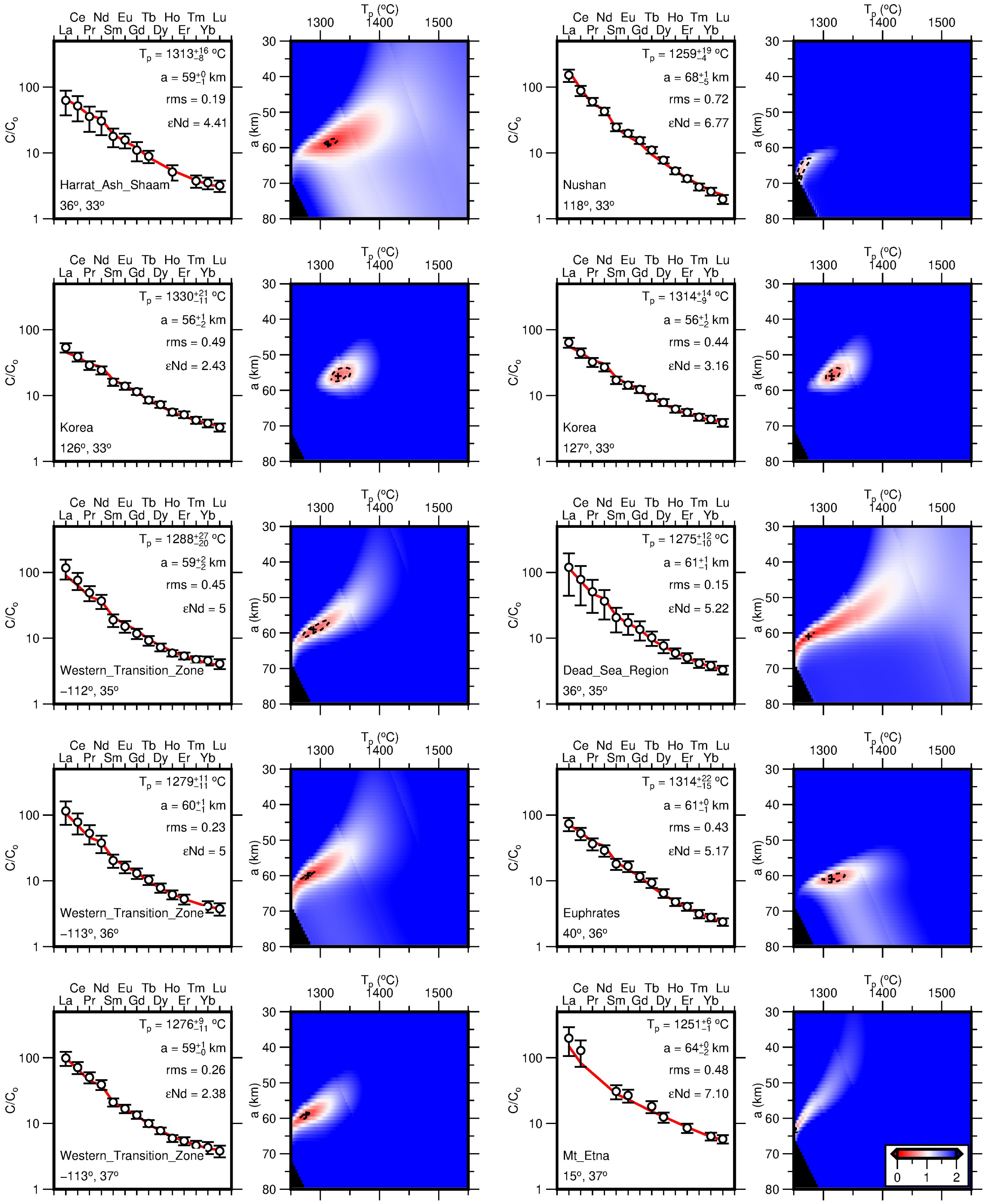

Ce Nd Eu Tb Ho Tm Lu
La Pr Sm Gd Dy Er Yb
C/C$_o$
a (km)
T_p (°C)
$T_p = 1313^{+16}_{-8}$ °C
$a = 59^{+0}_{-1}$ km
rms = 0.19
εNd = 4.41
Harrat_Ash_Shaam
36°, 33°
$T_p = 1259^{+19}_{-4}$ °C
$a = 68^{+1}_{-5}$ km
rms = 0.72
εNd = 6.77
Nushan
118°, 33°
$T_p = 1330^{+21}_{-11}$ °C
$a = 56^{+1}_{-2}$ km
rms = 0.49
εNd = 2.43
Korea
126°, 33°
$T_p = 1314^{+14}_{-9}$ °C
$a = 56^{+1}_{-2}$ km
rms = 0.44
εNd = 3.16
Korea
127°, 33°
$T_p = 1288^{+27}_{-20}$ °C
$a = 59^{+2}_{-2}$ km
rms = 0.45
εNd = 5
Western_Transition_Zone
–112°, 35°
$T_p = 1275^{+12}_{-10}$ °C
$a = 61^{+1}_{-1}$ km
rms = 0.15
εNd = 5.22
Dead_Sea_Region
36°, 35°
$T_p = 1279^{+11}_{-11}$ °C
$a = 60^{+1}_{-1}$ km
rms = 0.23
εNd = 5
Western_Transition_Zone
–113°, 36°
$T_p = 1314^{+22}_{-15}$ °C
$a = 61^{+0}_{-1}$ km
rms = 0.43
εNd = 5.17
Euphrates
40°, 36°
$T_p = 1276^{+9}_{-11}$ °C
$a = 59^{+1}_{-0}$ km
rms = 0.26
εNd = 2.38
Western_Transition_Zone
–113°, 37°
$T_p = 1251^{+6}_{-1}$ °C
$a = 64^{+0}_{-2}$ km
rms = 0.48
εNd = 7.10
Mt_Etna
15°, 37°

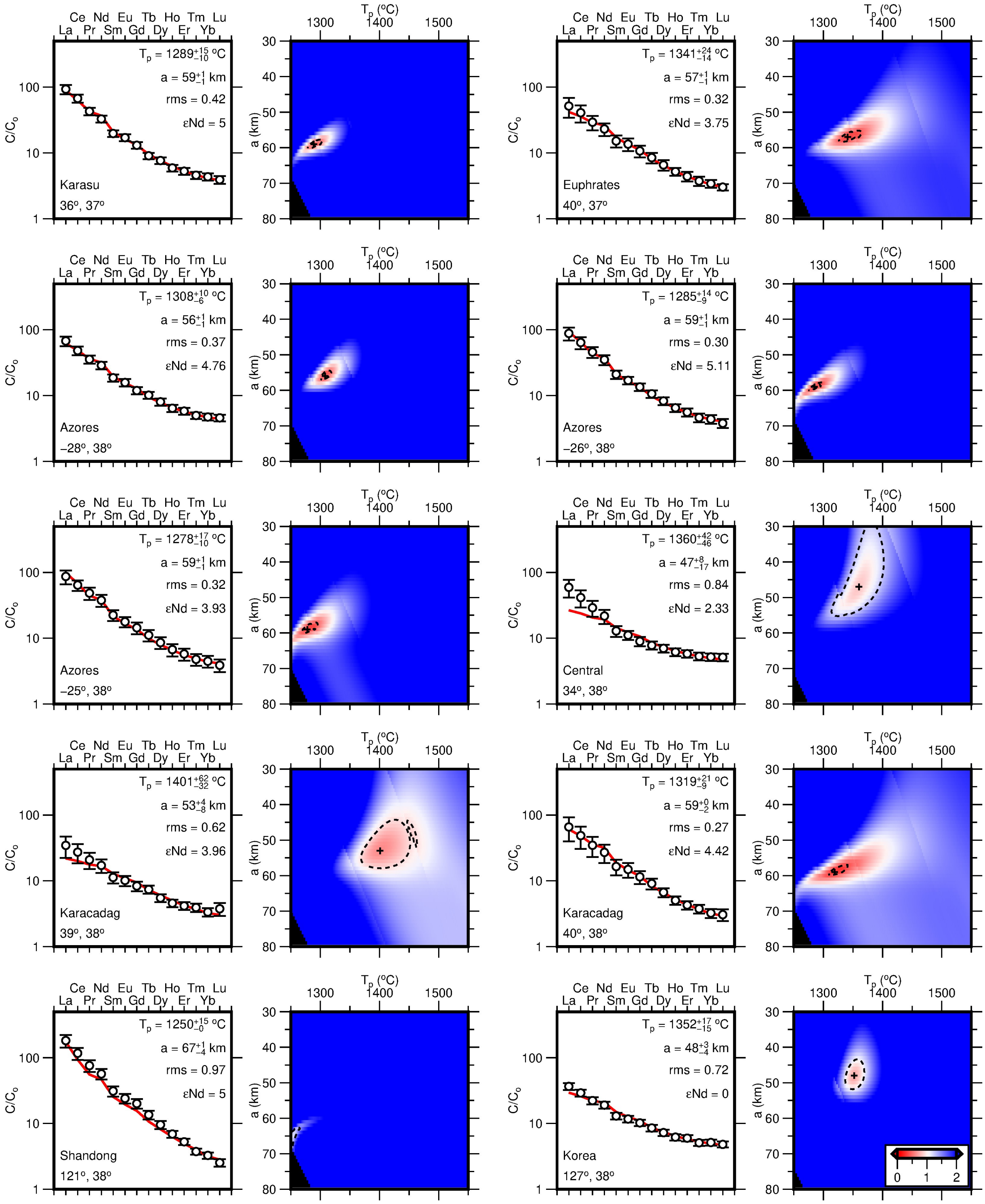
La Ce Pr Nd Sm Eu Gd Tb Dy Ho Er Tm Yb Lu
C/C$_o$
a (km)
T_p (°C)
1300 1400 1500
$T_p = 1289^{+15}_{-10}$ °C
$a = 59^{+1}_{-1}$ km
rms = 0.42
εNd = 5
Karasu
36°, 37°
$T_p = 1341^{+24}_{-14}$ °C
$a = 57^{+1}_{-1}$ km
rms = 0.32
εNd = 3.75
Euphrates
40°, 37°
$T_p = 1308^{+10}_{-6}$ °C
$a = 56^{+1}_{-1}$ km
rms = 0.37
εNd = 4.76
Azores
−28°, 38°
$T_p = 1285^{+14}_{-9}$ °C
$a = 59^{+1}_{-1}$ km
rms = 0.30
εNd = 5.11
Azores
−26°, 38°
$T_p = 1278^{+17}_{-10}$ °C
$a = 59^{+1}_{-1}$ km
rms = 0.32
εNd = 3.93
Azores
−25°, 38°
$T_p = 1360^{+42}_{-46}$ °C
$a = 47^{+8}_{-17}$ km
rms = 0.84
εNd = 2.33
Central
34°, 38°
$T_p = 1401^{+62}_{-32}$ °C
$a = 53^{+4}_{-8}$ km
rms = 0.62
εNd = 3.96
Karacadag
39°, 38°
$T_p = 1319^{+21}_{-9}$ °C
$a = 59^{+0}_{-2}$ km
rms = 0.27
εNd = 4.42
Karacadag
40°, 38°
$T_p = 1250^{+15}_{-0}$ °C
$a = 67^{+1}_{-4}$ km
rms = 0.97
εNd = 5
Shandong
121°, 38°
$T_p = 1352^{+17}_{-15}$ °C
$a = 48^{+3}_{-4}$ km
rms = 0.72
εNd = 0
Korea
127°, 38°
0 1 2

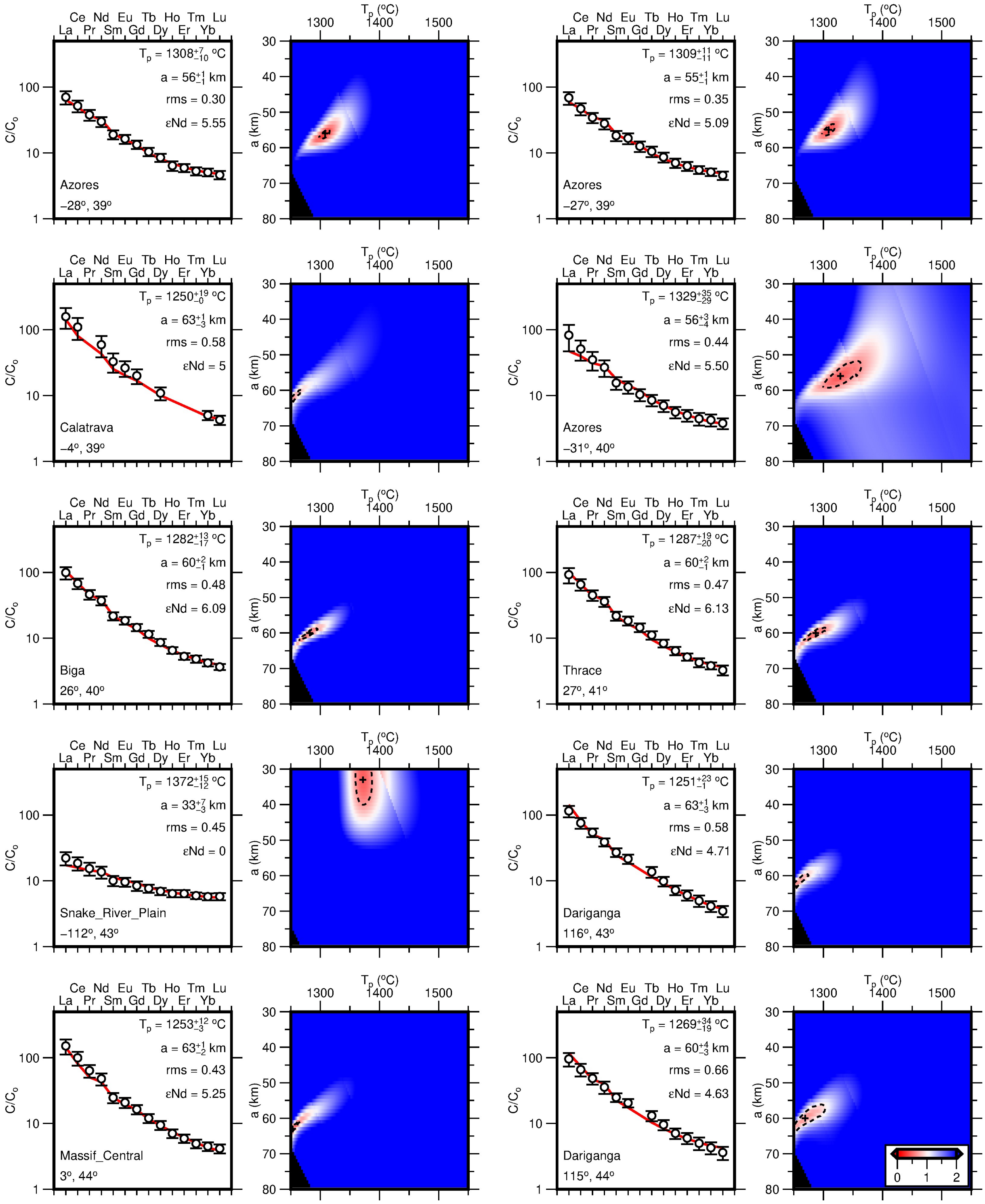

La Ce Pr Nd Sm Eu Gd Tb Dy Ho Er Tm Yb Lu
C/C$_o$
a (km)
T$_p$ (°C)
T$_p$ = 1308$^{+7}_{-10}$ °C
a = 56$^{+1}_{-1}$ km
rms = 0.30
εNd = 5.55
Azores
−28°, 39°
T$_p$ = 1309$^{+11}_{-11}$ °C
a = 55$^{+1}_{-1}$ km
rms = 0.35
εNd = 5.09
Azores
−27°, 39°
T$_p$ = 1250$^{+19}_{-0}$ °C
a = 63$^{+1}_{-3}$ km
rms = 0.58
εNd = 5
Calatrava
−4°, 39°
T$_p$ = 1329$^{+35}_{-29}$ °C
a = 56$^{+3}_{-4}$ km
rms = 0.44
εNd = 5.50
Azores
−31°, 40°
T$_p$ = 1282$^{+13}_{-17}$ °C
a = 60$^{+2}_{-1}$ km
rms = 0.48
εNd = 6.09
Biga
26°, 40°
T$_p$ = 1287$^{+19}_{-20}$ °C
a = 60$^{+2}_{-1}$ km
rms = 0.47
εNd = 6.13
Thrace
27°, 41°
T$_p$ = 1372$^{+15}_{-12}$ °C
a = 33$^{+7}_{-3}$ km
rms = 0.45
εNd = 0
Snake_River_Plain
−112°, 43°
T$_p$ = 1251$^{+23}_{-1}$ °C
a = 63$^{+1}_{-3}$ km
rms = 0.58
εNd = 4.71
Dariganga
116°, 43°
T$_p$ = 1253$^{+12}_{-3}$ °C
a = 63$^{+1}_{-2}$ km
rms = 0.43
εNd = 5.25
Massif_Central
3°, 44°
T$_p$ = 1269$^{+34}_{-19}$ °C
a = 60$^{+4}_{-3}$ km
rms = 0.66
εNd = 4.63
Dariganga
115°, 44°
0 1 2

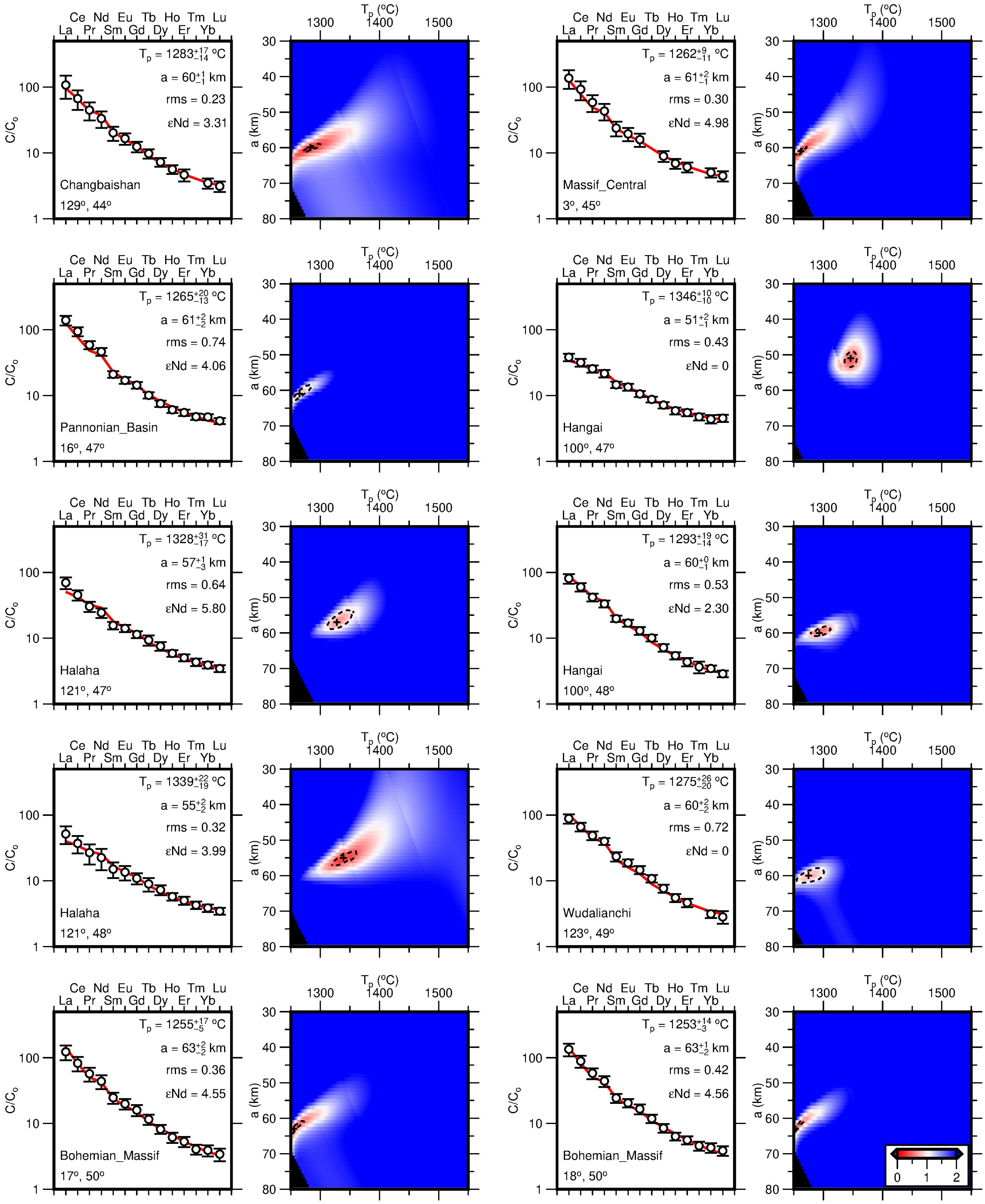

Changbaishan
129°, 44°
$T_p = 1283^{+17}_{-14}$ °C
$a = 60^{+1}_{-1}$ km
rms = 0.23
εNd = 3.31
Massif_Central
3°, 45°
$T_p = 1262^{+9}_{-11}$ °C
$a = 61^{+2}_{-1}$ km
rms = 0.30
εNd = 4.98
Pannonian_Basin
16°, 47°
$T_p = 1265^{+20}_{-13}$ °C
$a = 61^{+2}_{-2}$ km
rms = 0.74
εNd = 4.06
Hangai
100°, 47°
$T_p = 1346^{+10}_{-10}$ °C
$a = 51^{+2}_{-1}$ km
rms = 0.43
εNd = 0
Halaha
121°, 47°
$T_p = 1328^{+31}_{-17}$ °C
$a = 57^{+1}_{-3}$ km
rms = 0.64
εNd = 5.80
Hangai
100°, 48°
$T_p = 1293^{+19}_{-14}$ °C
$a = 60^{+0}_{-1}$ km
rms = 0.53
εNd = 2.30
Halaha
121°, 48°
$T_p = 1339^{+22}_{-19}$ °C
$a = 55^{+2}_{-2}$ km
rms = 0.32
εNd = 3.99
Wudalianchi
123°, 49°
$T_p = 1275^{+26}_{-20}$ °C
$a = 60^{+2}_{-2}$ km
rms = 0.72
εNd = 0
Bohemian_Massif
17°, 50°
$T_p = 1255^{+17}_{-5}$ °C
$a = 63^{+2}_{-2}$ km
rms = 0.36
εNd = 4.55
Bohemian_Massif
18°, 50°
$T_p = 1253^{+14}_{-3}$ °C
$a = 63^{+1}_{-2}$ km
rms = 0.42
εNd = 4.56
La Ce Pr Nd Sm Eu Gd Tb Dy Ho Er Tm Yb Lu
C/C_o
T_p (°C)
a (km)

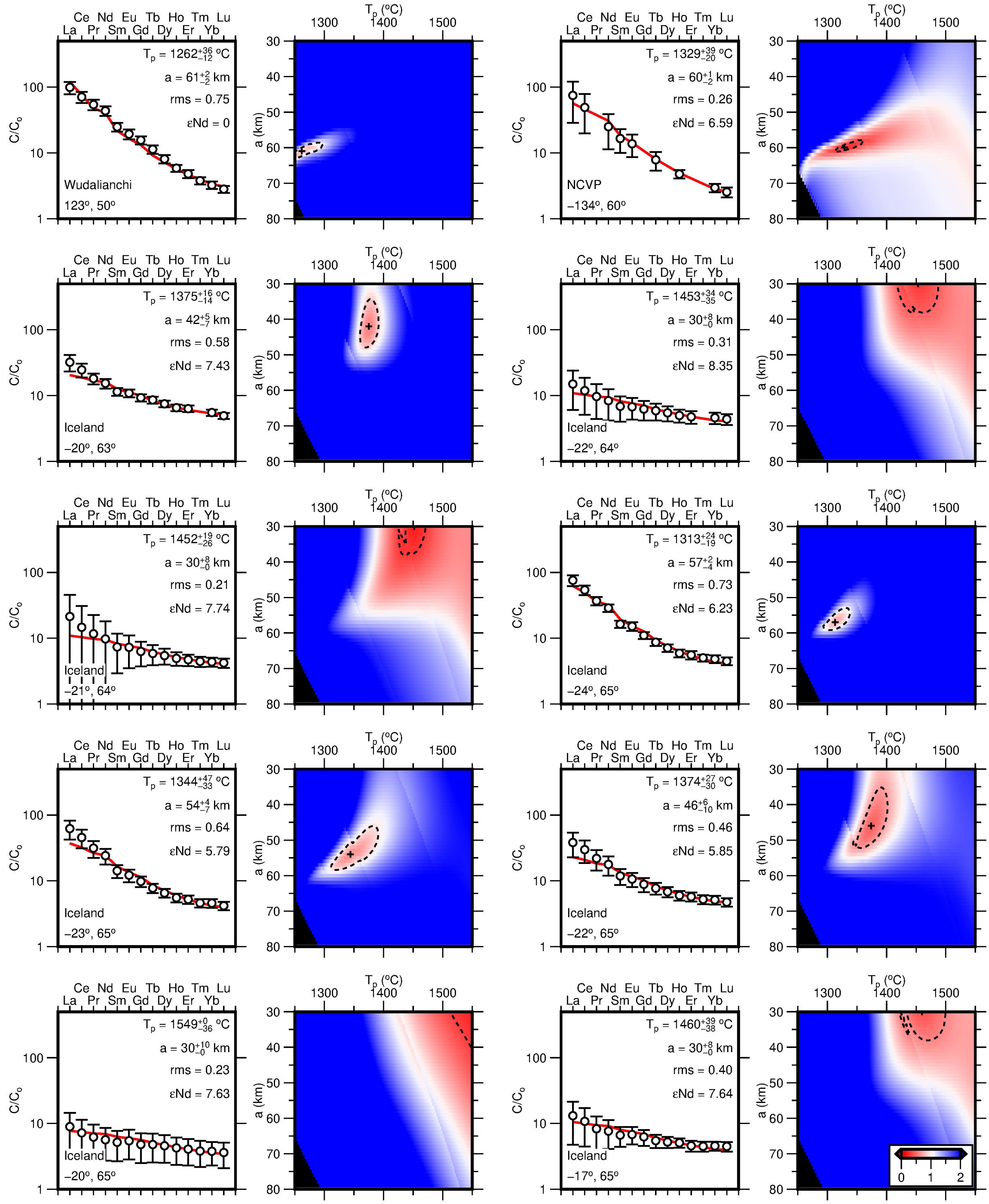
La Ce Pr Nd Sm Eu Gd Tb Dy Ho Er Tm Yb Lu
C/C$_o$
a (km)
T$_p$ (°C)
$T_p = 1262^{+36}_{-12}$ °C
$a = 61^{+2}_{-2}$ km
rms = 0.75
εNd = 0
Wudalianchi
123°, 50°
$T_p = 1329^{+39}_{-20}$ °C
$a = 60^{+1}_{-2}$ km
rms = 0.26
εNd = 6.59
NCVP
−134°, 60°
$T_p = 1375^{+16}_{-14}$ °C
$a = 42^{+5}_{-7}$ km
rms = 0.58
εNd = 7.43
Iceland
−20°, 63°
$T_p = 1453^{+34}_{-35}$ °C
$a = 30^{+8}_{-0}$ km
rms = 0.31
εNd = 8.35
Iceland
−22°, 64°
$T_p = 1452^{+19}_{-26}$ °C
$a = 30^{+8}_{-0}$ km
rms = 0.21
εNd = 7.74
Iceland
−21°, 64°
$T_p = 1313^{+24}_{-19}$ °C
$a = 57^{+2}_{-4}$ km
rms = 0.73
εNd = 6.23
Iceland
−24°, 65°
$T_p = 1344^{+47}_{-33}$ °C
$a = 54^{+4}_{-7}$ km
rms = 0.64
εNd = 5.79
Iceland
−23°, 65°
$T_p = 1374^{+27}_{-30}$ °C
$a = 46^{+6}_{-10}$ km
rms = 0.46
εNd = 5.85
Iceland
−22°, 65°
$T_p = 1549^{+0}_{-36}$ °C
$a = 30^{+10}_{-0}$ km
rms = 0.23
εNd = 7.63
Iceland
−20°, 65°
$T_p = 1460^{+39}_{-38}$ °C
$a = 30^{+8}_{-0}$ km
rms = 0.40
εNd = 7.64
Iceland
−17°, 65°
0 1 2

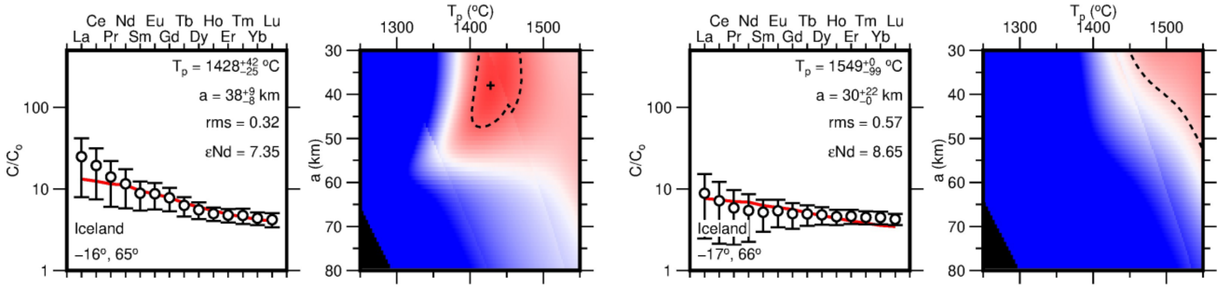

Ce Nd Eu Tb Ho Tm Lu
La Pr Sm Gd Dy Er Yb
C/C$_o$
Iceland
−16°, 65°
$T_p = 1428^{+42}_{-25}$ °C
$a = 38^{+9}_{-8}$ km
rms = 0.32
εNd = 7.35
T_p (°C)
a (km)
Iceland
−17°, 66°
$T_p = 1549^{+0}_{-99}$ °C
$a = 30^{+22}_{-0}$ km
rms = 0.57
εNd = 8.65

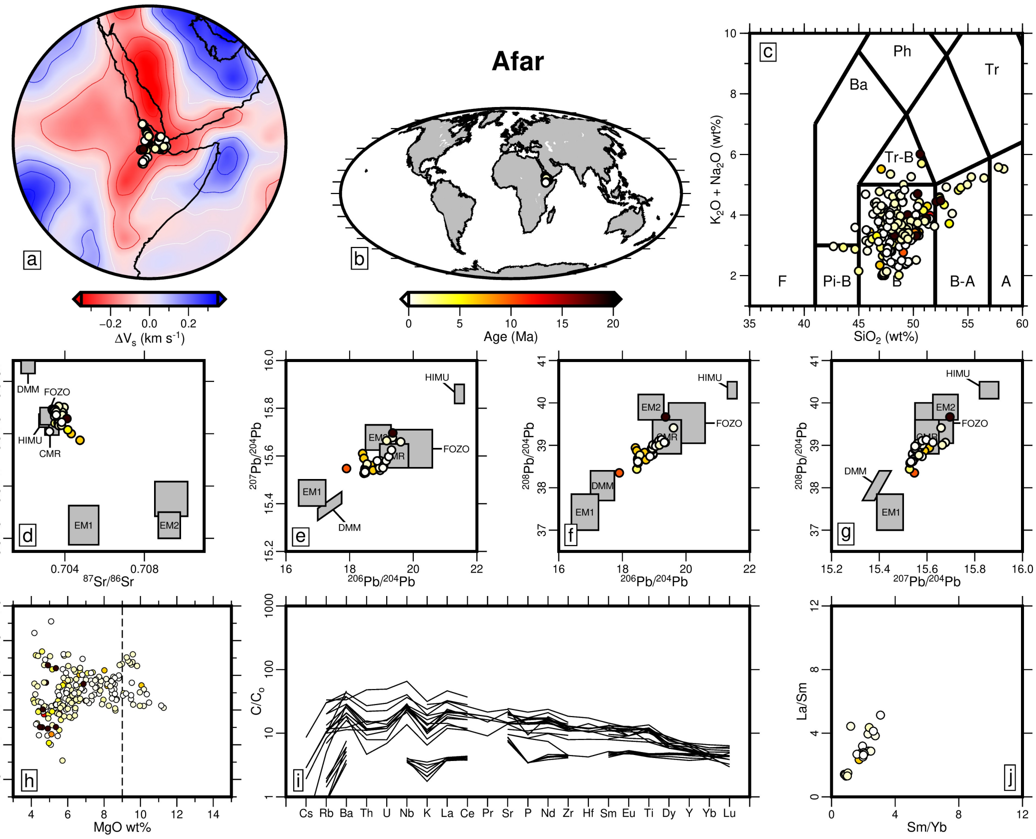

Supplementary Figure 10: Regional geochemical analyses. Geochemical information for Afar region (see Table 1). (a) SL2013sv model averaged between 100 and 200 km [859]. Red/white/blue contours = positive/zero/negative values of shear wave anomalies relative to Shaeffer & Lebedev's adapted version of Preliminary Reference Earth Model (PREM) plotted at intervals of 0.1 km s^{-1}. Circles = samples colored according to age. (b) Global map showing location of region. (c) Total alkalis (i.e. $K_2O + Na_2O$) plotted as function of SiO_2 for volcanic samples whose locations are shown in panel *a*. Sub-division and nomenclature follows standard scheme of Le Maitre (2002). F = Foidite, Pi-B = Picro-basalt, B = Basalt, B-A = Basaltic-Andesite, A = Andesite, Ba = Basanite, Tr-B = Trachy-Basalt, Tr = Trachyite. (d) $^{143}Nd/^{144}Nd$ ratios plotted as function of $^{87}Sr/^{86}Sr$ for samples where MgO < 6wt%. Labeled gray polygons = mantle reservoirs where DMM is depleted MORB mantle, FOZO is focal zone reservoir, HIMU is high-μ mantle, CMR is common mantle reservoir [618], and EM1/EM2 is enriched mantle 1/2 [1062]. (e) $^{207}Pb/^{204}Pb$ plotted as function of $^{206}Pb/^{204}Pb$. (f) $^{208}Pb/^{204}Pb$ plotted as function of $^{206}Pb/^{204}Pb$. (g) $^{208}Pb/^{204}Pb$ plotted as function of $^{207}Pb/^{204}Pb$. (h) Al_2O_3 plotted as function of MgO where vertical line indicates MgO > 9 wt%. (i) Trace element distribution of samples MgO > 9 wt%. Compositions are normalized with respect to primitive mantle [671]. (j) La/Sm plotted as function of Sm/Yb for samples with MgO > 9 wt%.

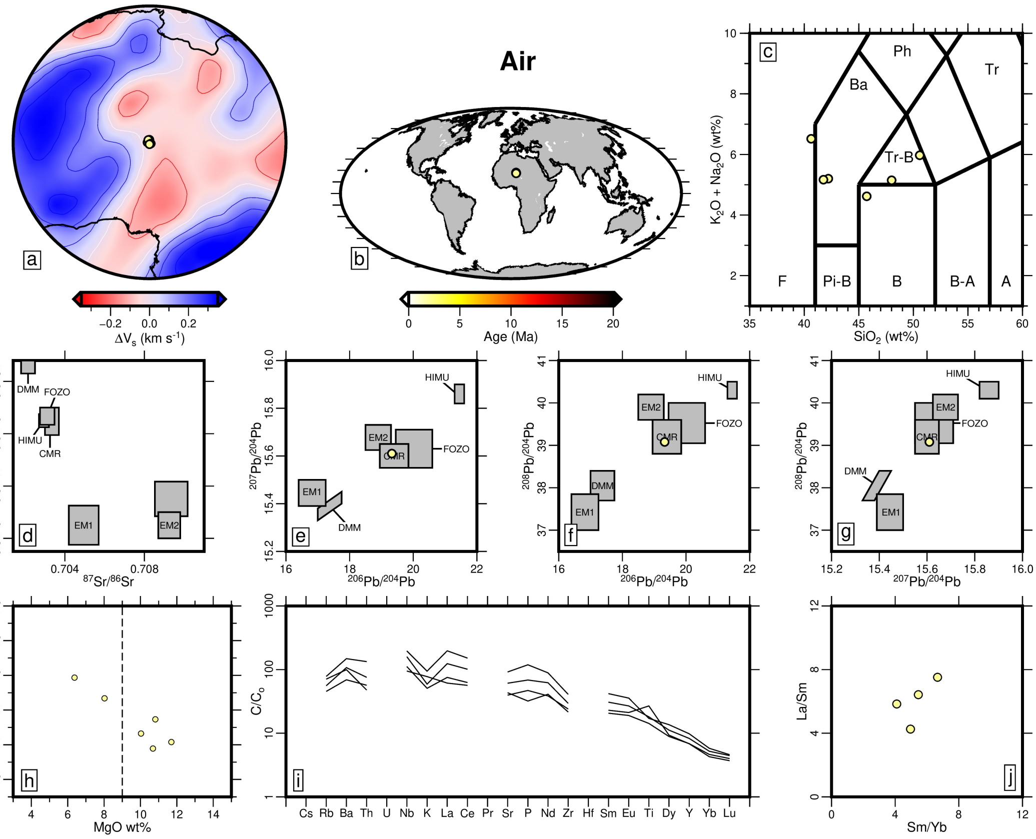
Air
a
b
c
ΔVs (km s-1)
Age (Ma)
SiO2 (wt%)
K2O + Na2O (wt%)
Ph
Tr
Ba
Tr-B
F
Pi-B
B
B-A
A
d
e
f
g
DMM
FOZO
HIMU
CMR
EM1
EM2
143Nd/144Nd
87Sr/86Sr
207Pb/204Pb
206Pb/204Pb
208Pb/204Pb
h
i
j
Al2O3 wt%
MgO wt%
C/Co
Cs Rb Ba Th U Nb K La Ce Pr Sr P Nd Zr Hf Sm Eu Ti Dy Y Yb Lu
La/Sm
Sm/Yb

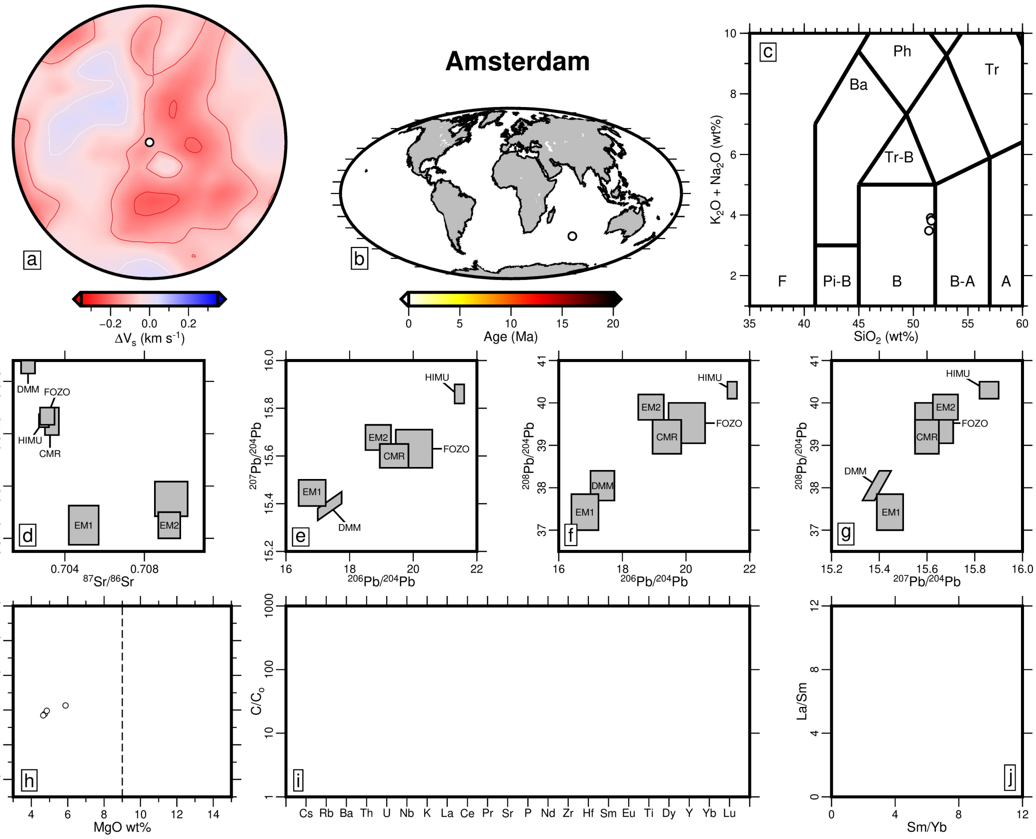
Amsterdam
a
b
c
ΔVs (km s-1)
Age (Ma)
SiO2 (wt%)
K2O + Na2O (wt%)
Ph
Tr
Ba
Tr-B
F
Pi-B
B
B-A
A
d
e
f
g
DMM
FOZO
HIMU
CMR
EM1
EM2
143Nd/144Nd
87Sr/86Sr
207Pb/204Pb
206Pb/204Pb
208Pb/204Pb
h
i
j
Al2O3 wt%
MgO wt%
C/Co
Cs Rb Ba Th U Nb K La Ce Pr Sr P Nd Zr Hf Sm Eu Ti Dy Y Yb Lu
La/Sm
Sm/Yb

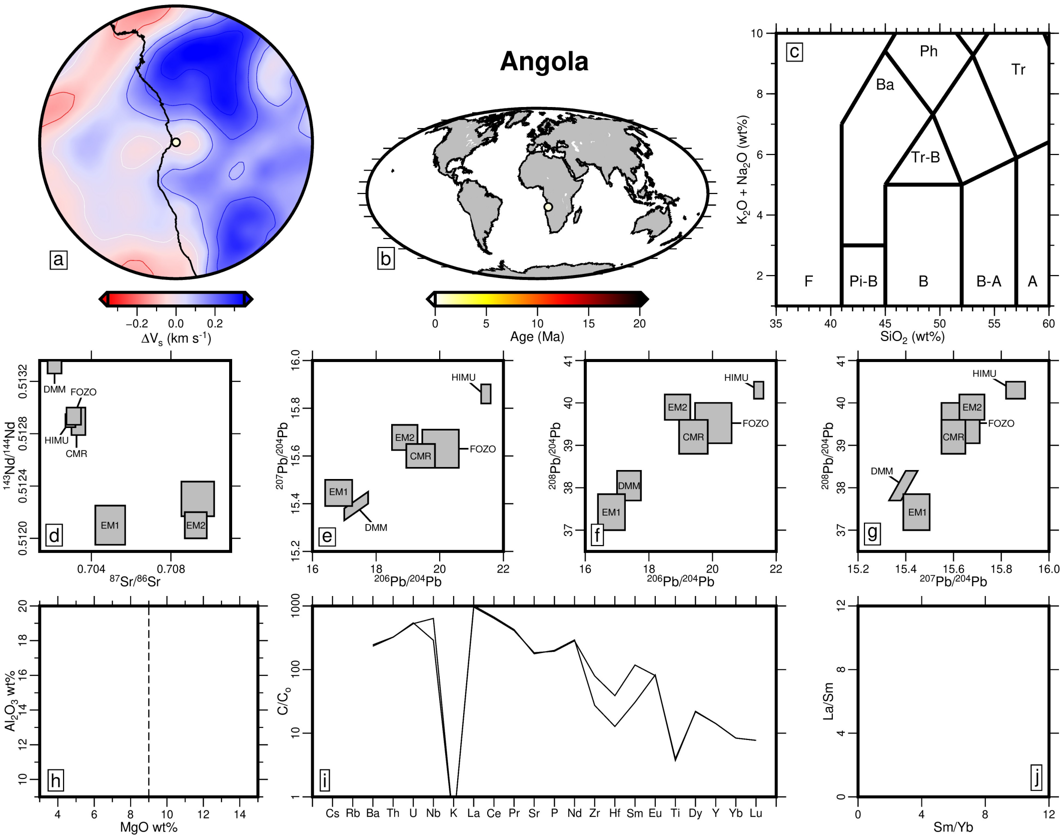
Angola
a
b
c
d
e
f
g
h
i
j
ΔVs (km s-1)
Age (Ma)
K2O + Na2O (wt%)
SiO2 (wt%)
Ph
Tr
Ba
Tr-B
F
Pi-B
B
B-A
A
143Nd/144Nd
87Sr/86Sr
207Pb/204Pb
206Pb/204Pb
208Pb/204Pb
DMM
FOZO
HIMU
CMR
EM1
EM2
Al2O3 wt%
MgO wt%
C/Co
Cs Rb Ba Th U Nb K La Ce Pr Sr P Nd Zr Hf Sm Eu Ti Dy Y Yb Lu
La/Sm
Sm/Yb

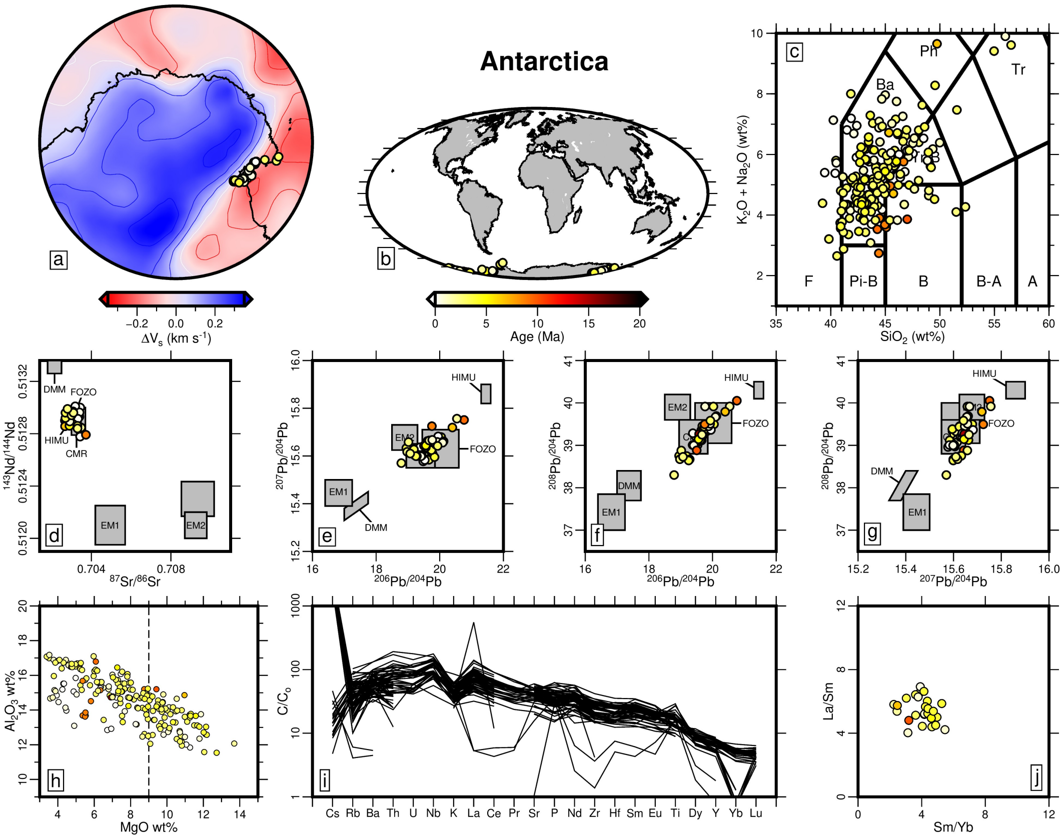
Antarctica
a
b
c
d
e
f
g
h
i
j
ΔVs (km s-1)
Age (Ma)
K2O + Na2O (wt%)
SiO2 (wt%)
Ph
Tr
Ba
F
Pi-B
B
B-A
A
143Nd/144Nd
87Sr/86Sr
207Pb/204Pb
206Pb/204Pb
208Pb/204Pb
DMM
FOZO
HIMU
CMR
EM1
EM2
Al2O3 wt%
MgO wt%
C/Co
Cs Rb Ba Th U Nb K La Ce Pr Sr P Nd Zr Hf Sm Eu Ti Dy Y Yb Lu
La/Sm
Sm/Yb

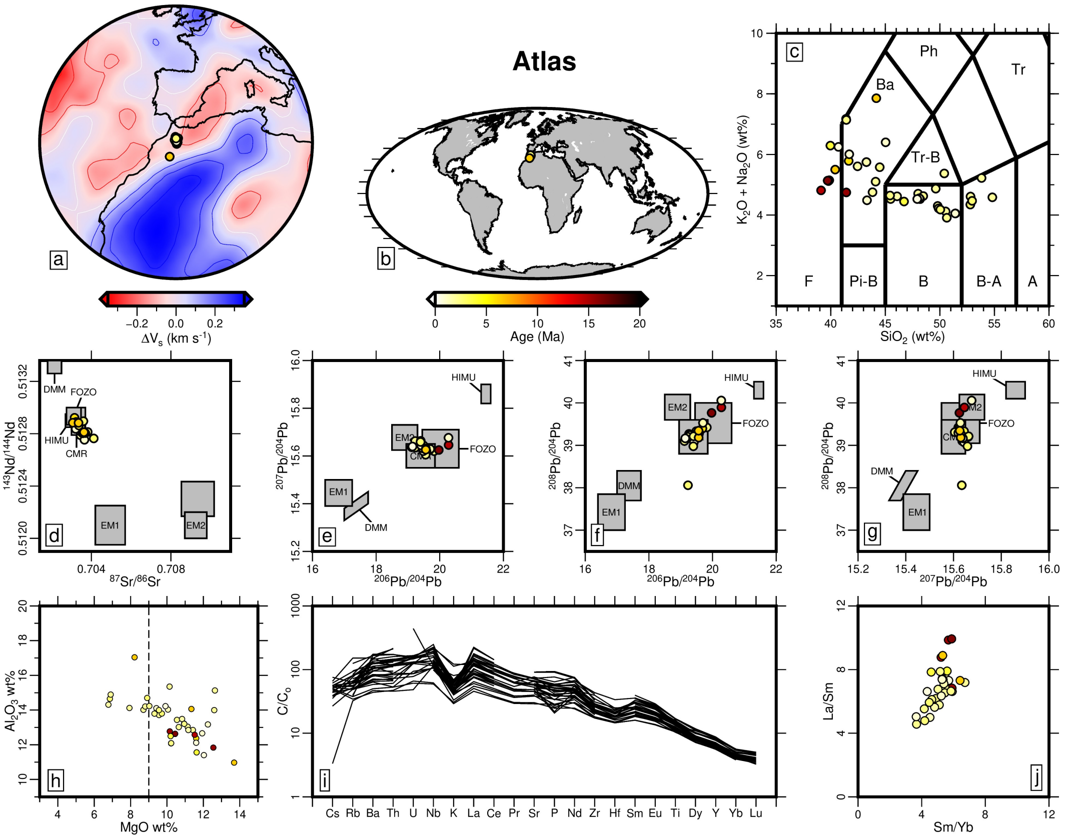
Atlas
a
b
c
−0.2 0.0 0.2
ΔVs (km s-1)
0 5 10 15 20
Age (Ma)
K2O + Na2O (wt%)
SiO2 (wt%)
F
Pi-B
B
B-A
A
Ba
Tr-B
Ph
Tr
d
e
f
g
143Nd/144Nd
87Sr/86Sr
207Pb/204Pb
206Pb/204Pb
208Pb/204Pb
207Pb/204Pb
DMM
FOZO
HIMU
CMR
EM1
EM2
h
i
j
Al2O3 wt%
MgO wt%
C/Co
Cs Rb Ba Th U Nb K La Ce Pr Sr P Nd Zr Hf Sm Eu Ti Dy Y Yb Lu
La/Sm
Sm/Yb

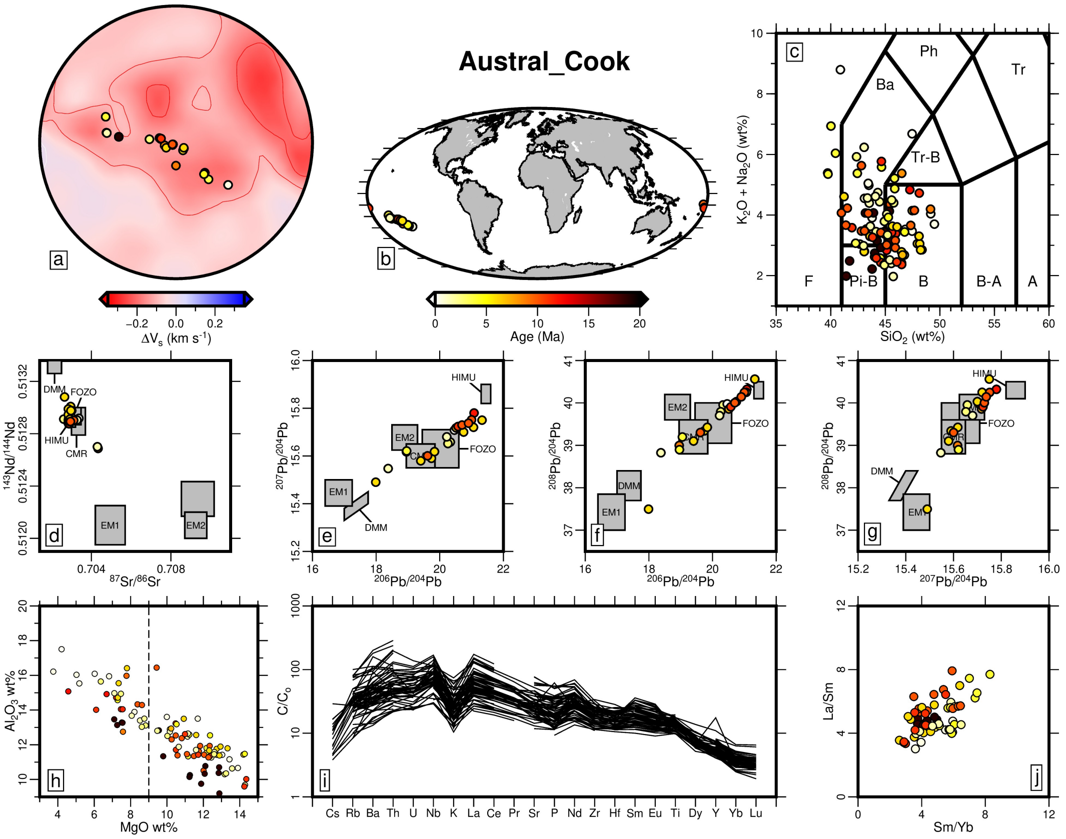
Austral_Cook
a
b
c
−0.2 0.0 0.2
ΔVs (km s-1)
0 5 10 15 20
Age (Ma)
K2O + Na2O (wt%)
SiO2 (wt%)
F
Pi-B
B
B-A
A
Ba
Tr-B
Ph
Tr
d
e
f
g
143Nd/144Nd
87Sr/86Sr
207Pb/204Pb
206Pb/204Pb
208Pb/204Pb
207Pb/204Pb
DMM
FOZO
HIMU
CMR
EM1
EM2
h
i
j
Al2O3 wt%
MgO wt%
C/Co
Cs Rb Ba Th U Nb K La Ce Pr Sr P Nd Zr Hf Sm Eu Ti Dy Y Yb Lu
La/Sm
Sm/Yb

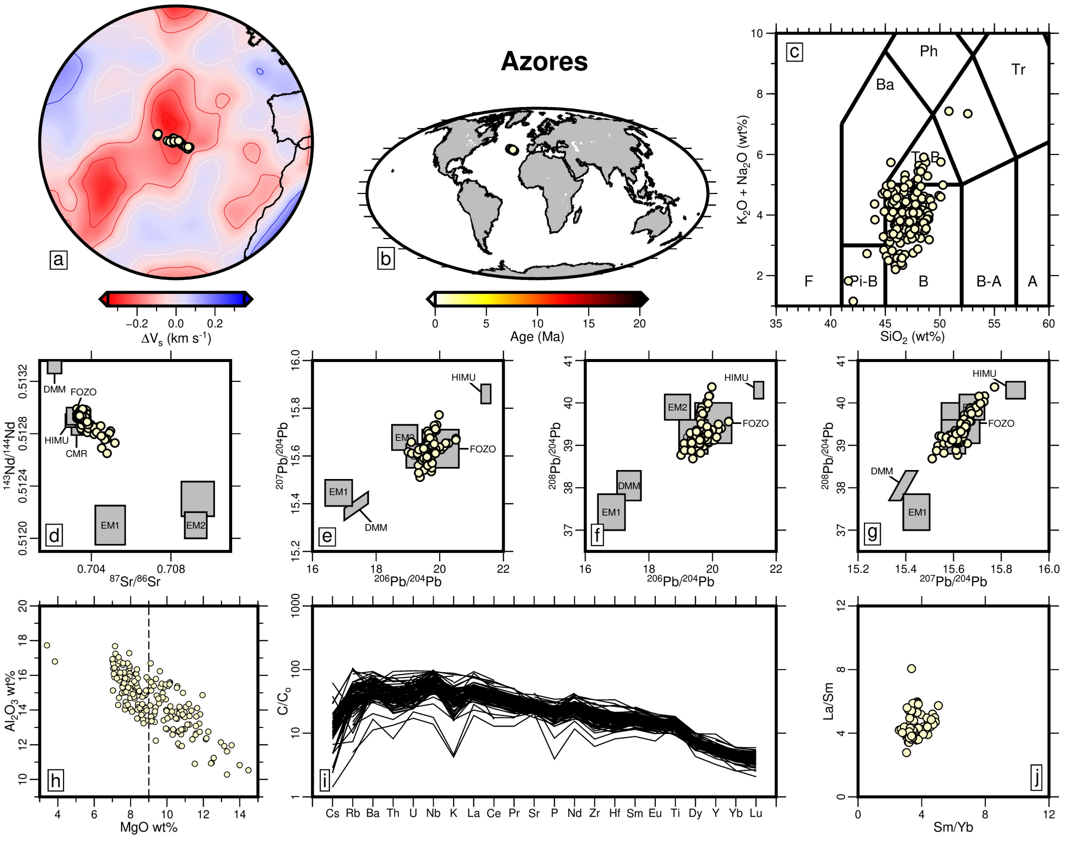
Azores
a
b
c
ΔV_s (km s^-1)
Age (Ma)
K_2O + Na_2O (wt%)
SiO_2 (wt%)
Ph
Tr
Ba
TeB
F
Pi-B
B
B-A
A
d
e
f
g
143Nd/144Nd
87Sr/86Sr
207Pb/204Pb
206Pb/204Pb
208Pb/204Pb
DMM
FOZO
HIMU
CMR
EM1
EM2
h
i
j
Al_2O_3 wt%
MgO wt%
C/C_0
Cs Rb Ba Th U Nb K La Ce Pr Sr P Nd Zr Hf Sm Eu Ti Dy Y Yb Lu
La/Sm
Sm/Yb

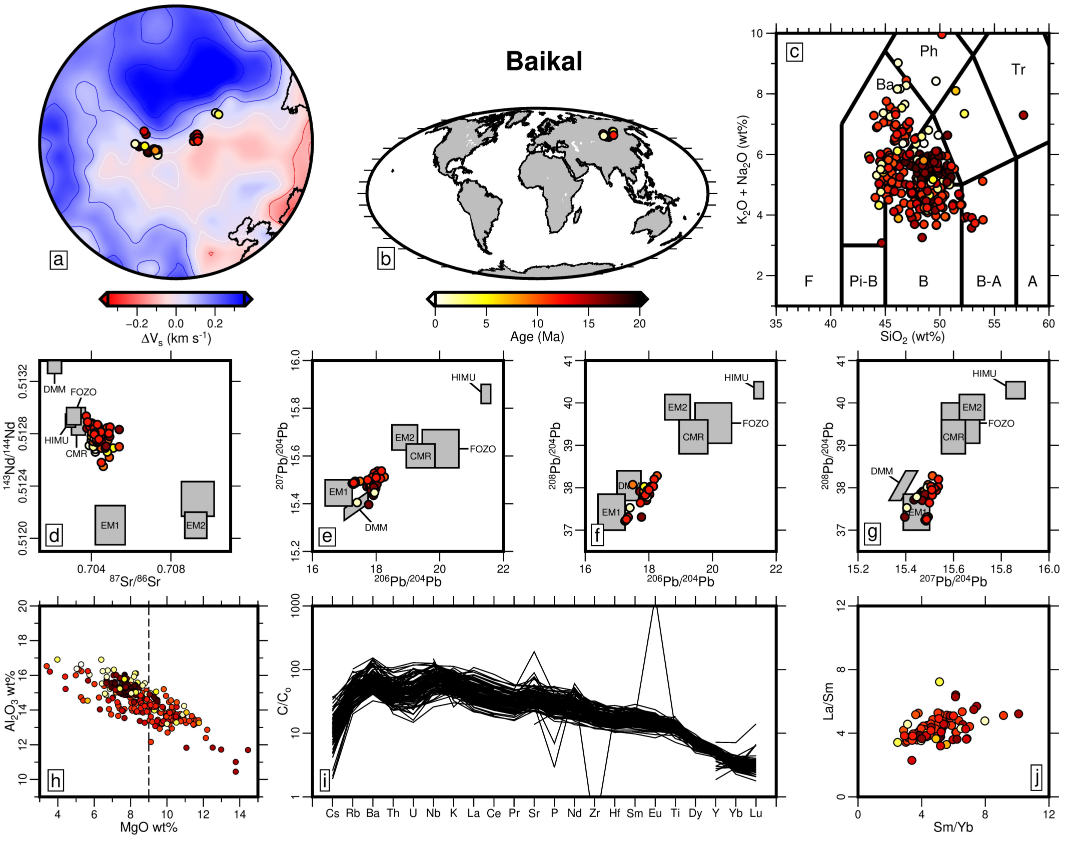
Baikal
a
b
c
ΔV_s (km s^-1)
Age (Ma)
K_2O + Na_2O (wt%)
SiO_2 (wt%)
Ph
Tr
Ba
F
Pi-B
B
B-A
A
d
e
f
g
143Nd/144Nd
87Sr/86Sr
207Pb/204Pb
206Pb/204Pb
208Pb/204Pb
DMM
FOZO
HIMU
CMR
EM1
EM2
h
i
j
Al_2O_3 wt%
MgO wt%
C/C_0
Cs Rb Ba Th U Nb K La Ce Pr Sr P Nd Zr Hf Sm Eu Ti Dy Y Yb Lu
La/Sm
Sm/Yb

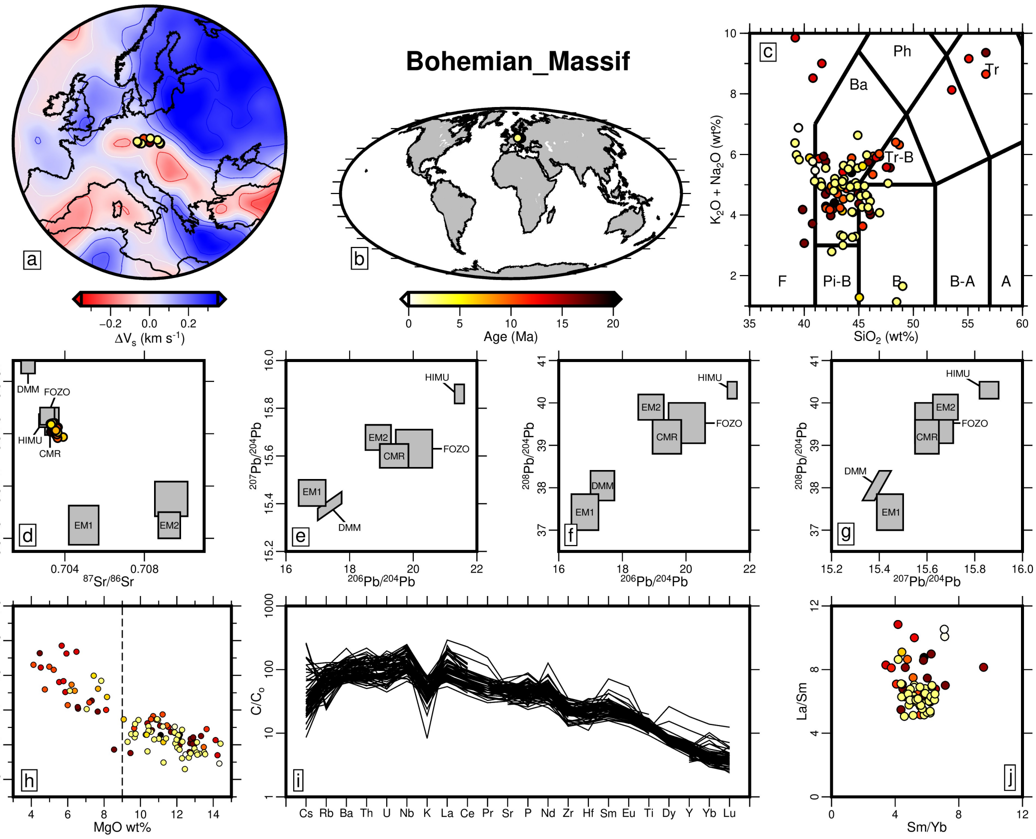
Bohemian_Massif
a
b
c
d
e
f
g
h
i
j
ΔVs (km s-1)
Age (Ma)
SiO2 (wt%)
K2O + Na2O (wt%)
Ph
Ba
Tr
Tr-B
F
Pi-B
B
B-A
A
143Nd/144Nd
87Sr/86Sr
207Pb/204Pb
206Pb/204Pb
208Pb/204Pb
DMM
FOZO
HIMU
CMR
EM1
EM2
Al2O3 wt%
MgO wt%
C/Co
Cs Rb Ba Th U Nb K La Ce Pr Sr P Nd Zr Hf Sm Eu Ti Dy Y Yb Lu
La/Sm
Sm/Yb

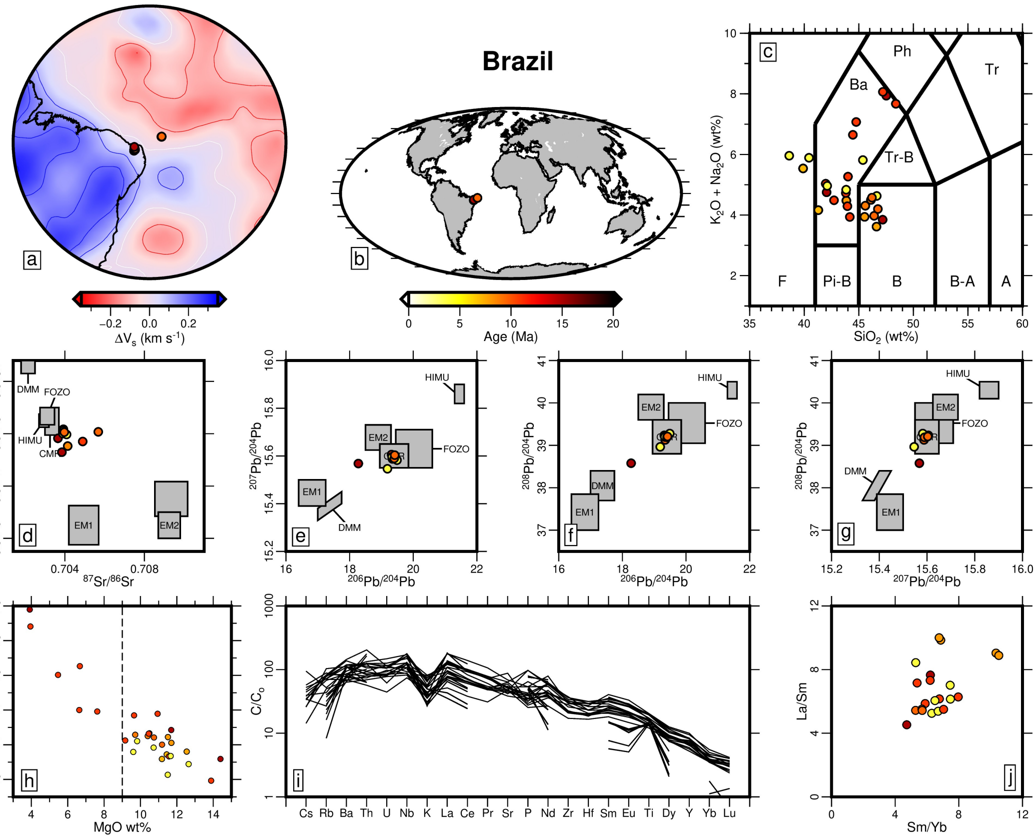
Brazil
a
b
c
d
e
f
g
h
i
j
ΔVs (km s-1)
Age (Ma)
SiO2 (wt%)
K2O + Na2O (wt%)
Ph
Ba
Tr
Tr-B
F
Pi-B
B
B-A
A
143Nd/144Nd
87Sr/86Sr
207Pb/204Pb
206Pb/204Pb
208Pb/204Pb
DMM
FOZO
HIMU
CMR
EM1
EM2
Al2O3 wt%
MgO wt%
C/Co
Cs Rb Ba Th U Nb K La Ce Pr Sr P Nd Zr Hf Sm Eu Ti Dy Y Yb Lu
La/Sm
Sm/Yb

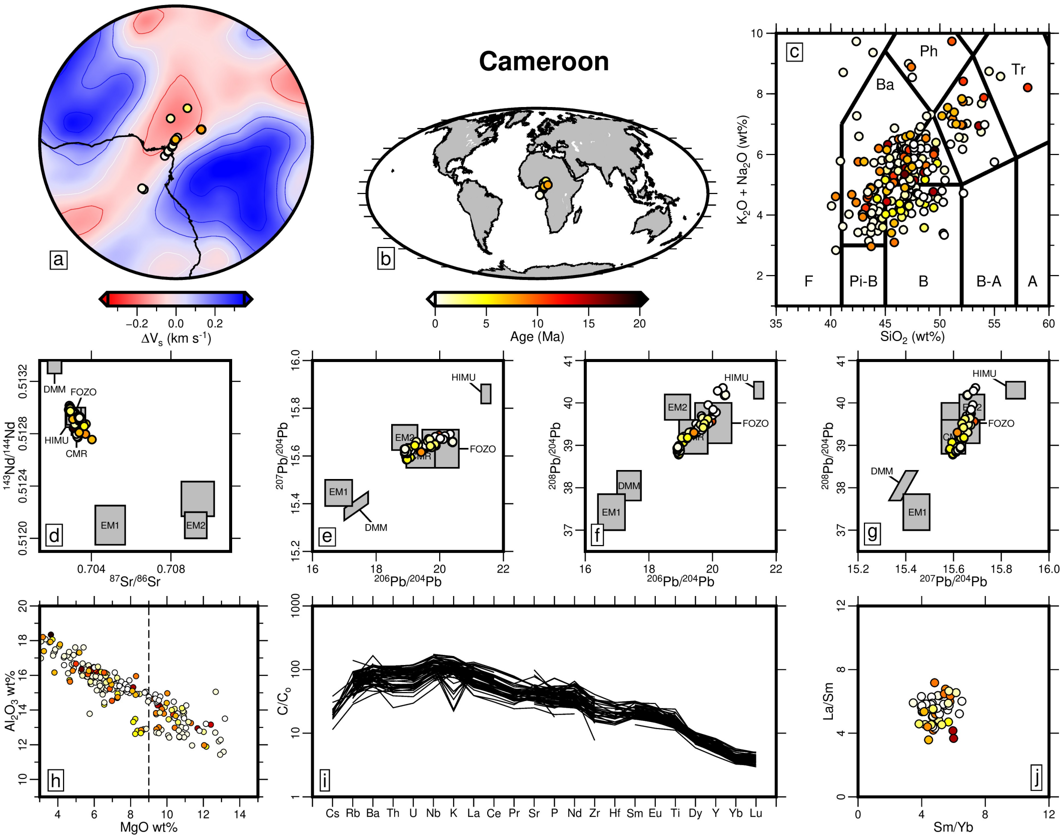
Cameroon
a
ΔVs (km s-1)
b
Age (Ma)
c
K2O + Na2O (wt%)
SiO2 (wt%)
F
Pi-B
B
B-A
A
Ba
Ph
Tr
d
143Nd/144Nd
87Sr/86Sr
DMM
FOZO
HIMU
CMR
EM1
EM2
e
207Pb/204Pb
206Pb/204Pb
f
208Pb/204Pb
206Pb/204Pb
g
208Pb/204Pb
207Pb/204Pb
h
Al2O3 wt%
MgO wt%
i
C/C0
Cs Rb Ba Th U Nb K La Ce Pr Sr P Nd Zr Hf Sm Eu Ti Dy Y Yb Lu
j
La/Sm
Sm/Yb

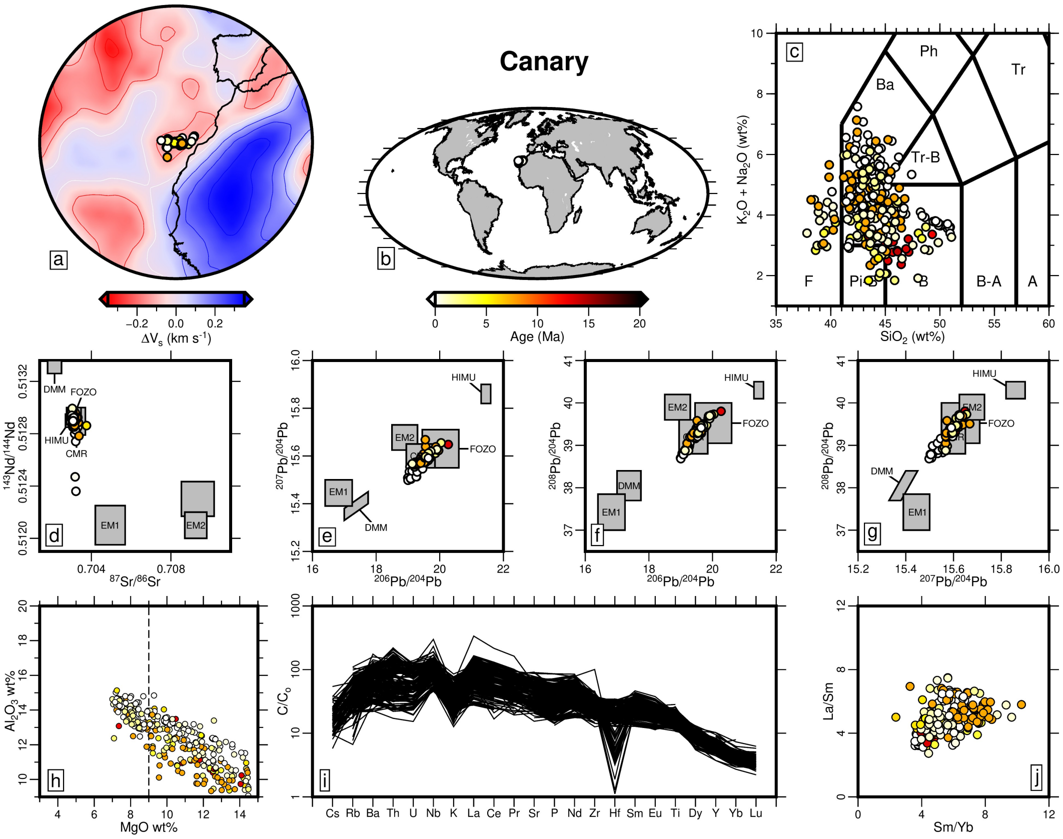
Canary
a
ΔVs (km s-1)
b
Age (Ma)
c
K2O + Na2O (wt%)
SiO2 (wt%)
F
Pi
B
B-A
A
Ba
Ph
Tr
Tr-B
d
143Nd/144Nd
87Sr/86Sr
DMM
FOZO
HIMU
CMR
EM1
EM2
e
207Pb/204Pb
206Pb/204Pb
f
208Pb/204Pb
206Pb/204Pb
g
208Pb/204Pb
207Pb/204Pb
h
Al2O3 wt%
MgO wt%
i
C/C0
Cs Rb Ba Th U Nb K La Ce Pr Sr P Nd Zr Hf Sm Eu Ti Dy Y Yb Lu
j
La/Sm
Sm/Yb

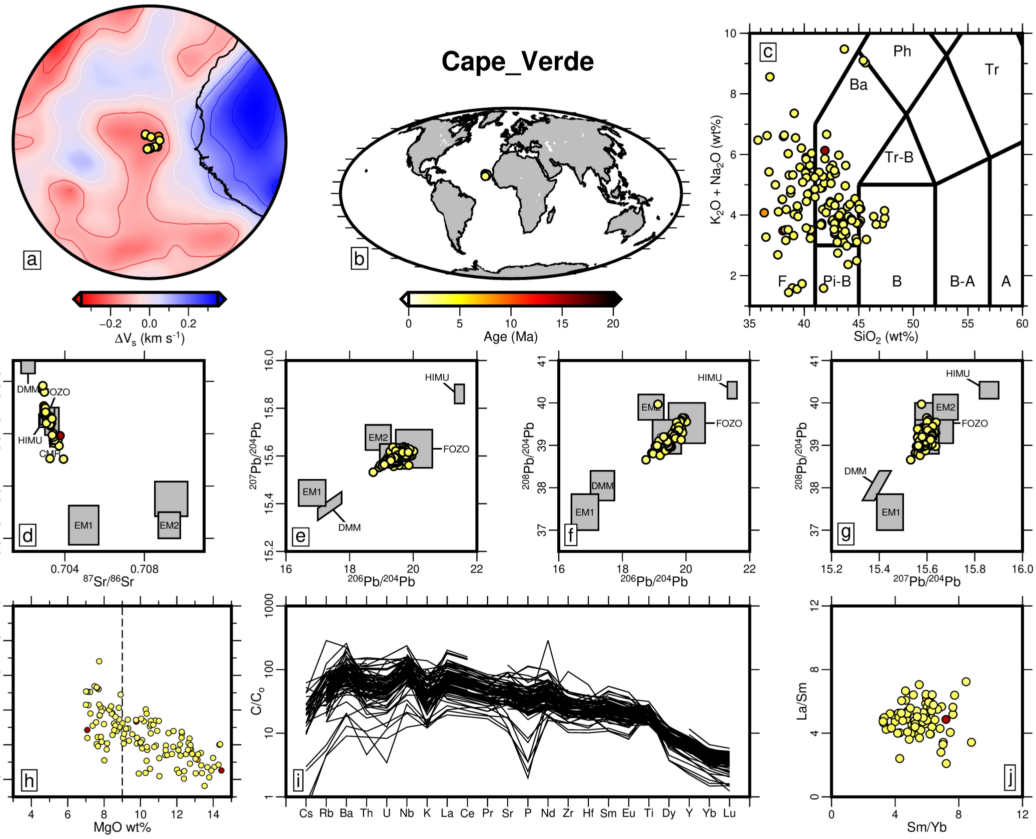
Cape_Verde
a
−0.2 0.0 0.2
ΔVs (km s-1)
b
0 5 10 15 20
Age (Ma)
c
Ph
Tr
Ba
Tr-B
F
Pi-B
B
B-A
A
K2O + Na2O (wt%)
SiO2 (wt%)
d
DMM
FOZO
HIMU
CMR
EM1
EM2
143Nd/144Nd
87Sr/86Sr
e
HIMU
EM2
FOZO
EM1
DMM
207Pb/204Pb
206Pb/204Pb
f
HIMU
EM2
FOZO
DMM
EM1
208Pb/204Pb
206Pb/204Pb
g
HIMU
EM2
FOZO
DMM
EM1
208Pb/204Pb
207Pb/204Pb
h
Al2O3 wt%
MgO wt%
i
C/Co
Cs Rb Ba Th U Nb K La Ce Pr Sr P Nd Zr Hf Sm Eu Ti Dy Y Yb Lu
j
La/Sm
Sm/Yb

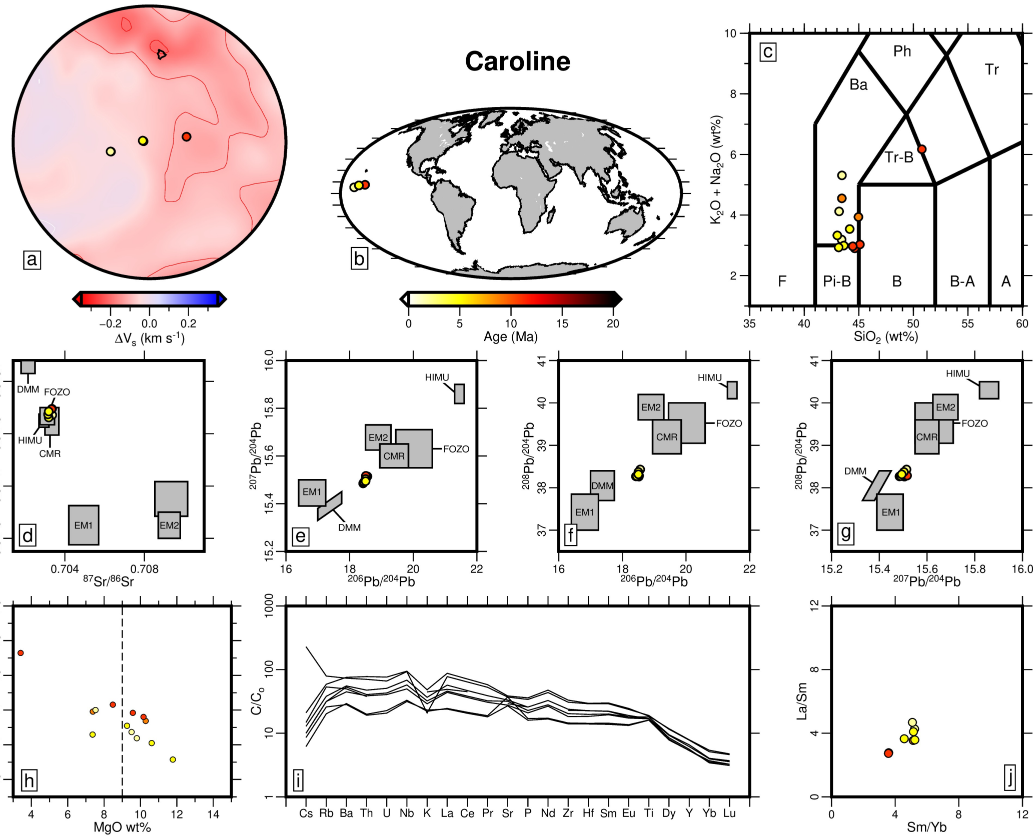
Caroline
a
−0.2 0.0 0.2
ΔVs (km s-1)
b
0 5 10 15 20
Age (Ma)
c
Ph
Tr
Ba
Tr-B
F
Pi-B
B
B-A
A
K2O + Na2O (wt%)
SiO2 (wt%)
d
DMM
FOZO
HIMU
CMR
EM1
EM2
143Nd/144Nd
87Sr/86Sr
e
HIMU
EM2
CMR
FOZO
EM1
DMM
207Pb/204Pb
206Pb/204Pb
f
HIMU
EM2
CMR
FOZO
DMM
EM1
208Pb/204Pb
206Pb/204Pb
g
HIMU
EM2
CMR
FOZO
DMM
EM1
208Pb/204Pb
207Pb/204Pb
h
Al2O3 wt%
MgO wt%
i
C/Co
Cs Rb Ba Th U Nb K La Ce Pr Sr P Nd Zr Hf Sm Eu Ti Dy Y Yb Lu
j
La/Sm
Sm/Yb

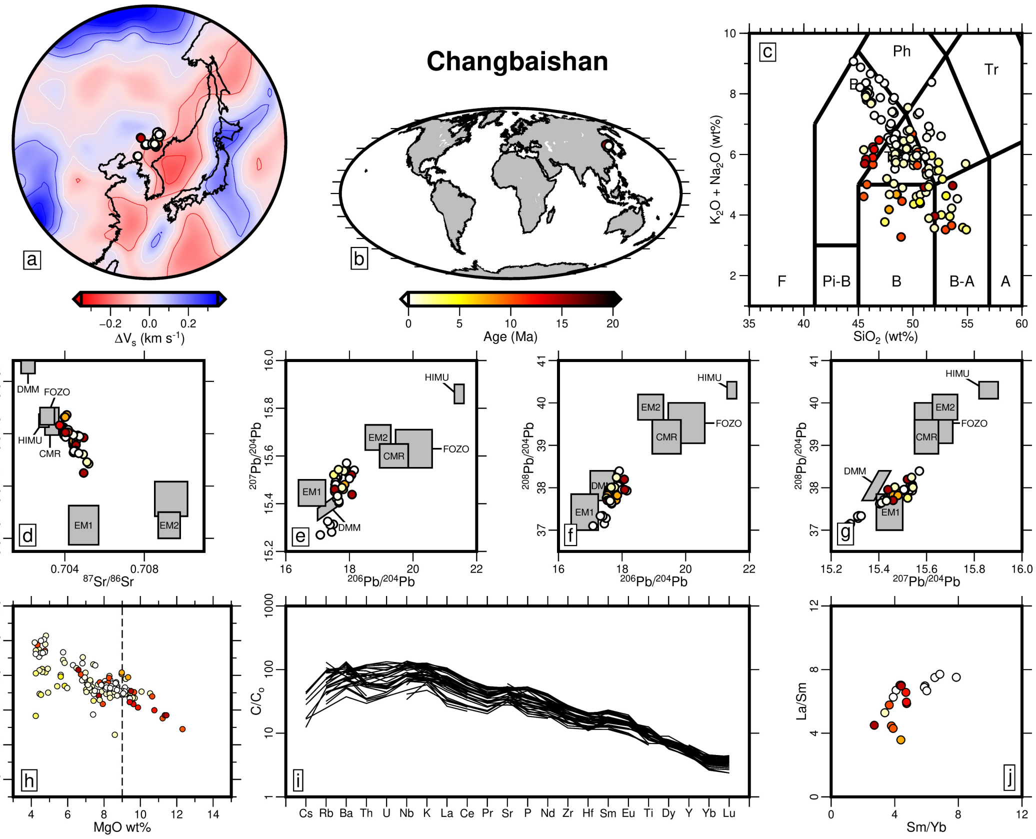
Changbaishan
a
ΔVs (km s-1)
b
Age (Ma)
c
Ph
Tr
F
Pi-B
B
B-A
A
K2O + Na2O (wt%)
SiO2 (wt%)
d
DMM
FOZO
HIMU
CMR
EM1
EM2
143Nd/144Nd
87Sr/86Sr
e
207Pb/204Pb
206Pb/204Pb
f
208Pb/204Pb
g
h
Al2O3 wt%
MgO wt%
i
C/Co
Cs Rb Ba Th U Nb K La Ce Pr Sr P Nd Zr Hf Sm Eu Ti Dy Y Yb Lu
j
La/Sm
Sm/Yb

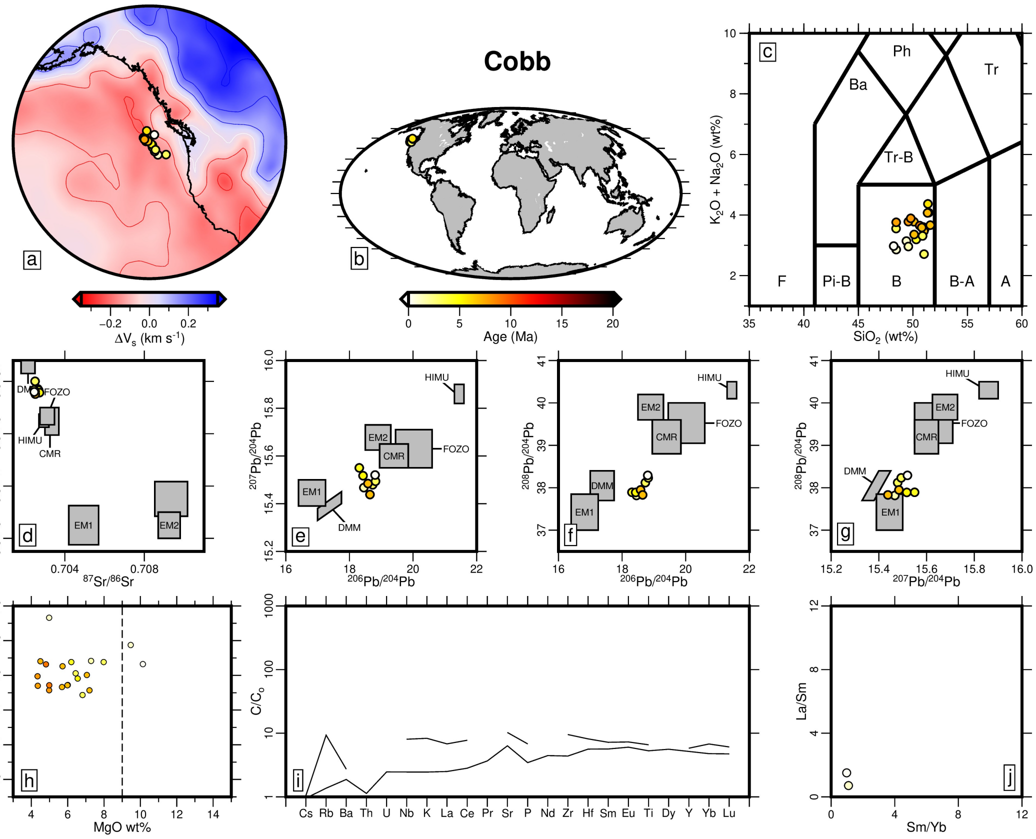
Cobb
a
ΔVs (km s-1)
b
Age (Ma)
c
Ph
Tr
Ba
Tr-B
F
Pi-B
B
B-A
A
K2O + Na2O (wt%)
SiO2 (wt%)
d
FOZO
HIMU
CMR
EM1
EM2
143Nd/144Nd
87Sr/86Sr
e
DMM
207Pb/204Pb
206Pb/204Pb
f
208Pb/204Pb
g
h
Al2O3 wt%
MgO wt%
i
C/Co
Cs Rb Ba Th U Nb K La Ce Pr Sr P Nd Zr Hf Sm Eu Ti Dy Y Yb Lu
j
La/Sm
Sm/Yb

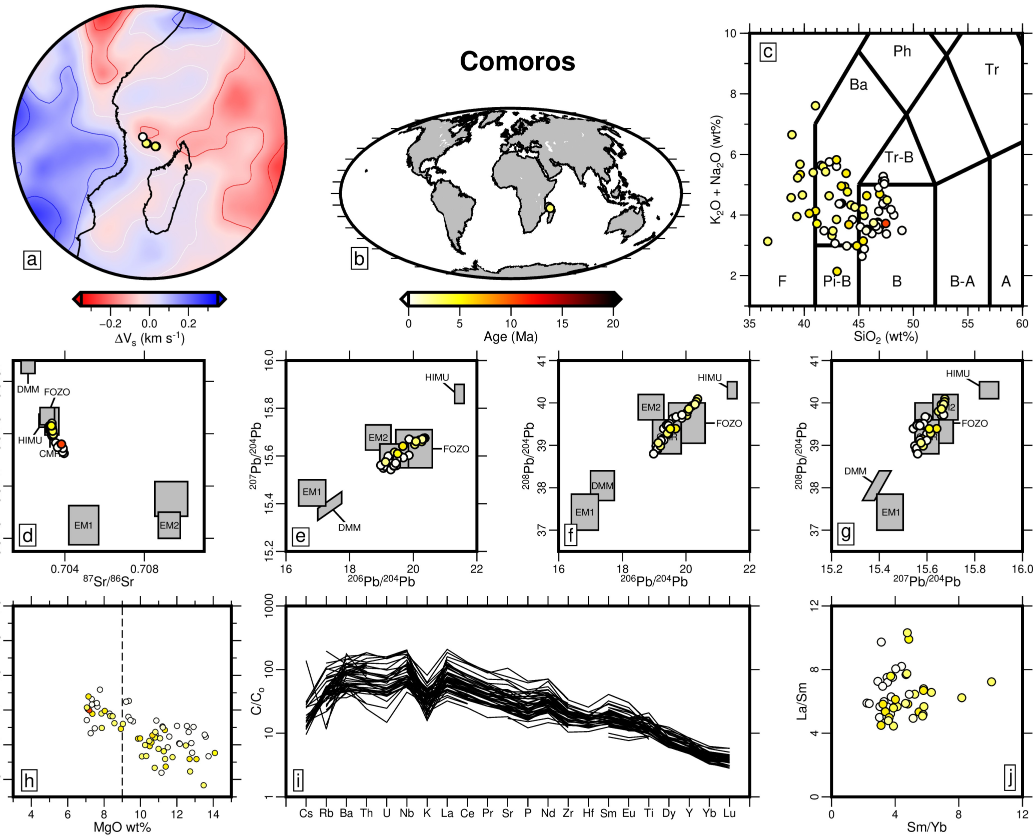
Comoros
a
ΔVs (km s-1)
−0.2 0.0 0.2
b
Age (Ma)
0 5 10 15 20
c
K2O + Na2O (wt%)
SiO2 (wt%)
Ph
Tr
Ba
Tr-B
F
Pi-B
B
B-A
A
d
143Nd/144Nd
87Sr/86Sr
DMM
FOZO
HIMU
CMR
EM1
EM2
e
207Pb/204Pb
206Pb/204Pb
f
208Pb/204Pb
206Pb/204Pb
g
208Pb/204Pb
207Pb/204Pb
h
Al2O3 wt%
MgO wt%
i
C/Co
Cs Rb Ba Th U Nb K La Ce Pr Sr P Nd Zr Hf Sm Eu Ti Dy Y Yb Lu
j
La/Sm
Sm/Yb

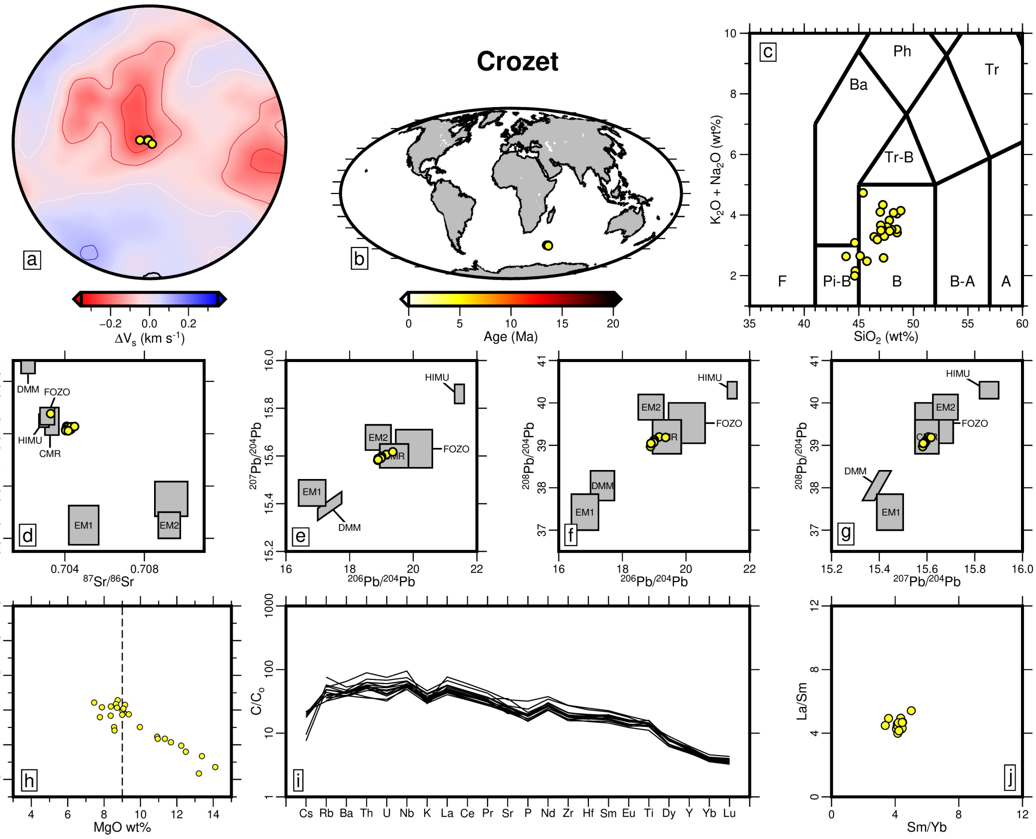
Crozet
a
ΔVs (km s-1)
−0.2 0.0 0.2
b
Age (Ma)
0 5 10 15 20
c
K2O + Na2O (wt%)
SiO2 (wt%)
Ph
Tr
Ba
Tr-B
F
Pi-B
B
B-A
A
d
143Nd/144Nd
87Sr/86Sr
DMM
FOZO
HIMU
CMR
EM1
EM2
e
207Pb/204Pb
206Pb/204Pb
f
208Pb/204Pb
206Pb/204Pb
g
208Pb/204Pb
207Pb/204Pb
h
Al2O3 wt%
MgO wt%
i
C/Co
Cs Rb Ba Th U Nb K La Ce Pr Sr P Nd Zr Hf Sm Eu Ti Dy Y Yb Lu
j
La/Sm
Sm/Yb

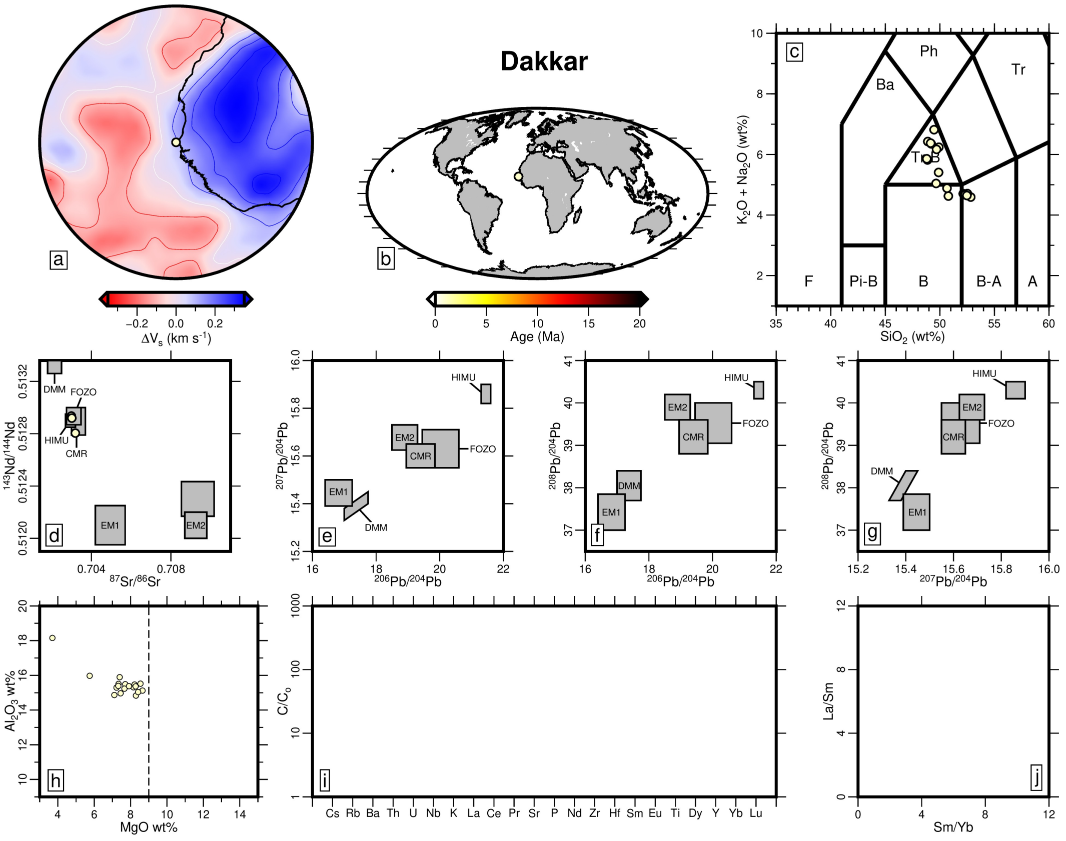
Dakkar
a
ΔVs (km s-1)
b
Age (Ma)
c
Ph
Ba
Tr
TrB
F
Pi-B
B
B-A
A
K2O + Na2O (wt%)
SiO2 (wt%)
d
143Nd/144Nd
87Sr/86Sr
DMM
FOZO
HIMU
CMR
EM1
EM2
e
207Pb/204Pb
206Pb/204Pb
f
208Pb/204Pb
206Pb/204Pb
g
208Pb/204Pb
207Pb/204Pb
h
Al2O3 wt%
MgO wt%
i
C/Co
Cs Rb Ba Th U Nb K La Ce Pr Sr P Nd Zr Hf Sm Eu Ti Dy Y Yb Lu
j
La/Sm
Sm/Yb

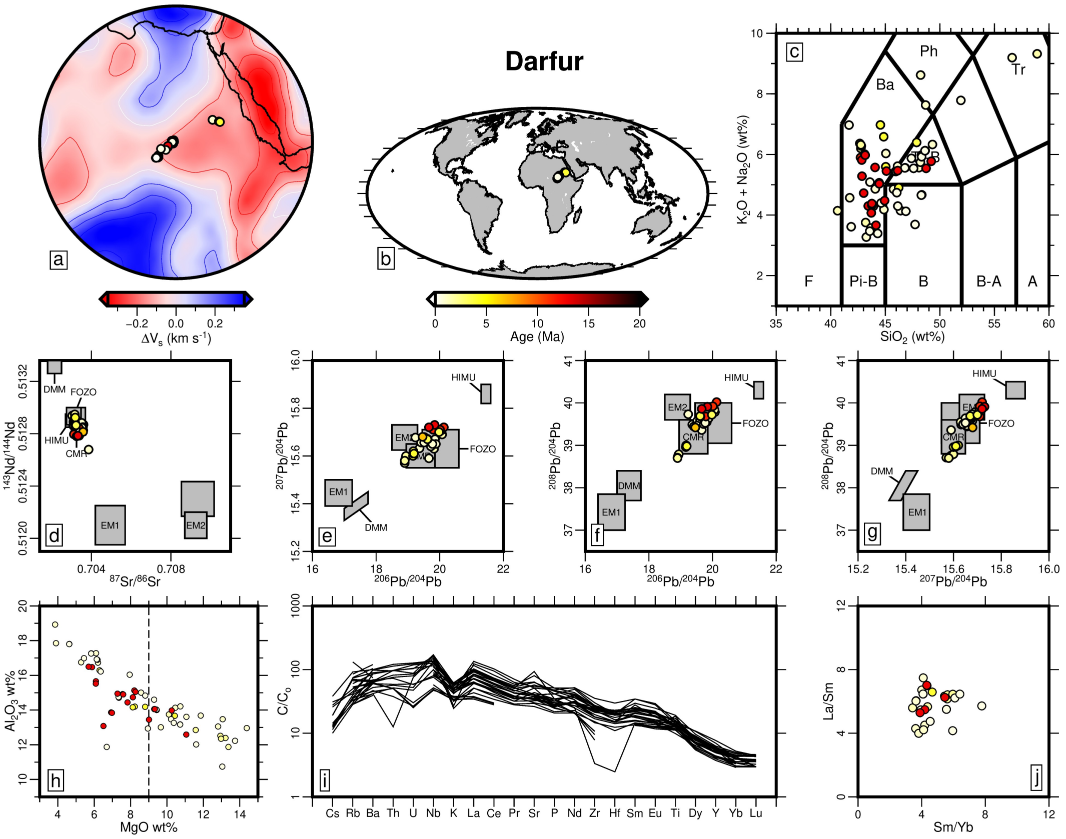
Darfur
a
ΔVs (km s-1)
b
Age (Ma)
c
Ph
Ba
Tr
F
Pi-B
B
B-A
A
K2O + Na2O (wt%)
SiO2 (wt%)
d
143Nd/144Nd
87Sr/86Sr
DMM
FOZO
HIMU
CMR
EM1
EM2
e
207Pb/204Pb
206Pb/204Pb
f
208Pb/204Pb
206Pb/204Pb
g
208Pb/204Pb
207Pb/204Pb
h
Al2O3 wt%
MgO wt%
i
C/Co
Cs Rb Ba Th U Nb K La Ce Pr Sr P Nd Zr Hf Sm Eu Ti Dy Y Yb Lu
j
La/Sm
Sm/Yb

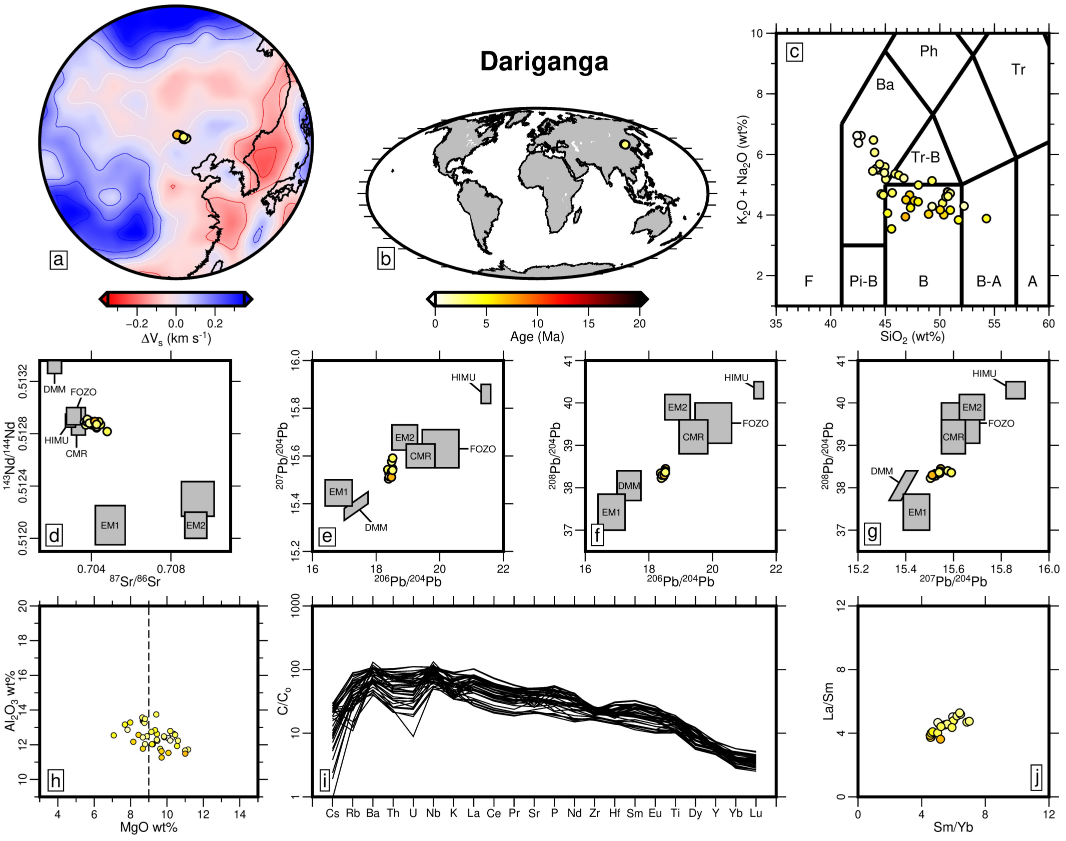
Dariganga
a
b
c
d
e
f
g
h
i
j
ΔVs (km s-1)
Age (Ma)
SiO2 (wt%)
K2O + Na2O (wt%)
Ph
Tr
Ba
Tr-B
F
Pi-B
B
B-A
A
87Sr/86Sr
143Nd/144Nd
206Pb/204Pb
207Pb/204Pb
208Pb/204Pb
DMM
FOZO
HIMU
CMR
EM1
EM2
MgO wt%
Al2O3 wt%
C/Co
Cs Rb Ba Th U Nb K La Ce Pr Sr P Nd Zr Hf Sm Eu Ti Dy Y Yb Lu
Sm/Yb
La/Sm

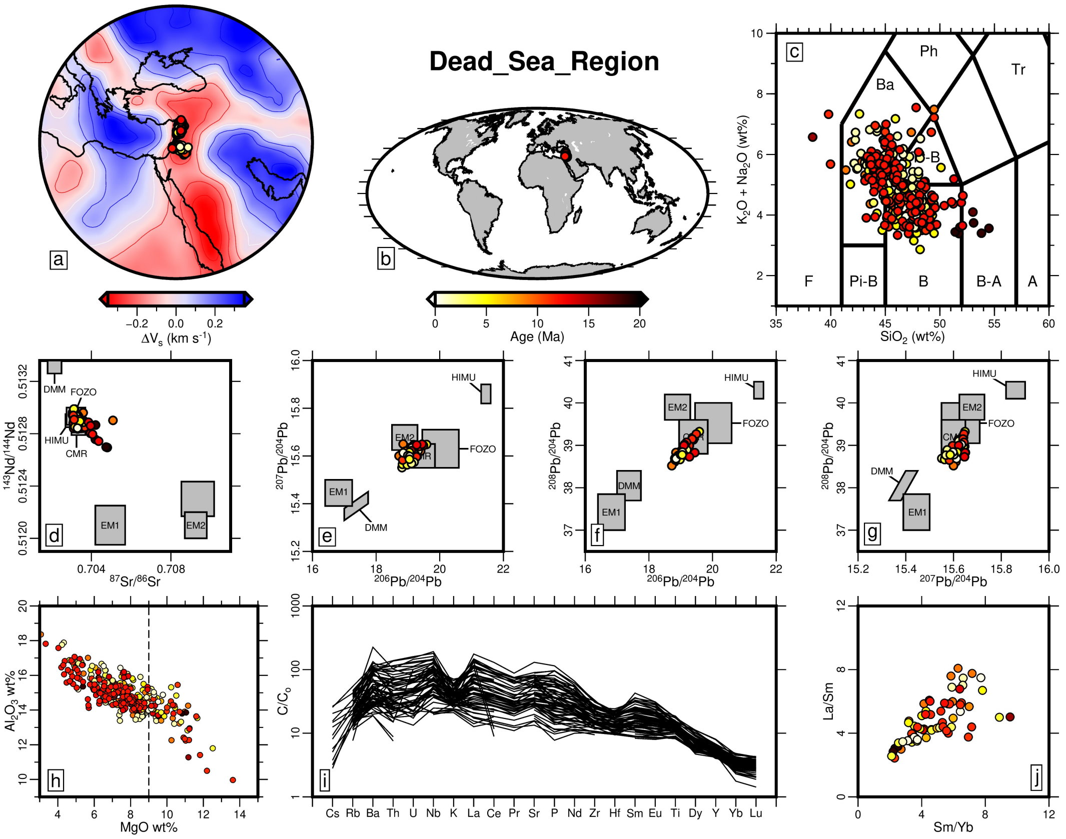
Dead_Sea_Region
a
b
c
d
e
f
g
h
i
j
ΔVs (km s-1)
Age (Ma)
SiO2 (wt%)
K2O + Na2O (wt%)
Ph
Tr
Ba
F
Pi-B
B
B-A
A
87Sr/86Sr
143Nd/144Nd
206Pb/204Pb
207Pb/204Pb
208Pb/204Pb
DMM
FOZO
HIMU
CMR
EM1
EM2
MgO wt%
Al2O3 wt%
C/Co
Cs Rb Ba Th U Nb K La Ce Pr Sr P Nd Zr Hf Sm Eu Ti Dy Y Yb Lu
Sm/Yb
La/Sm

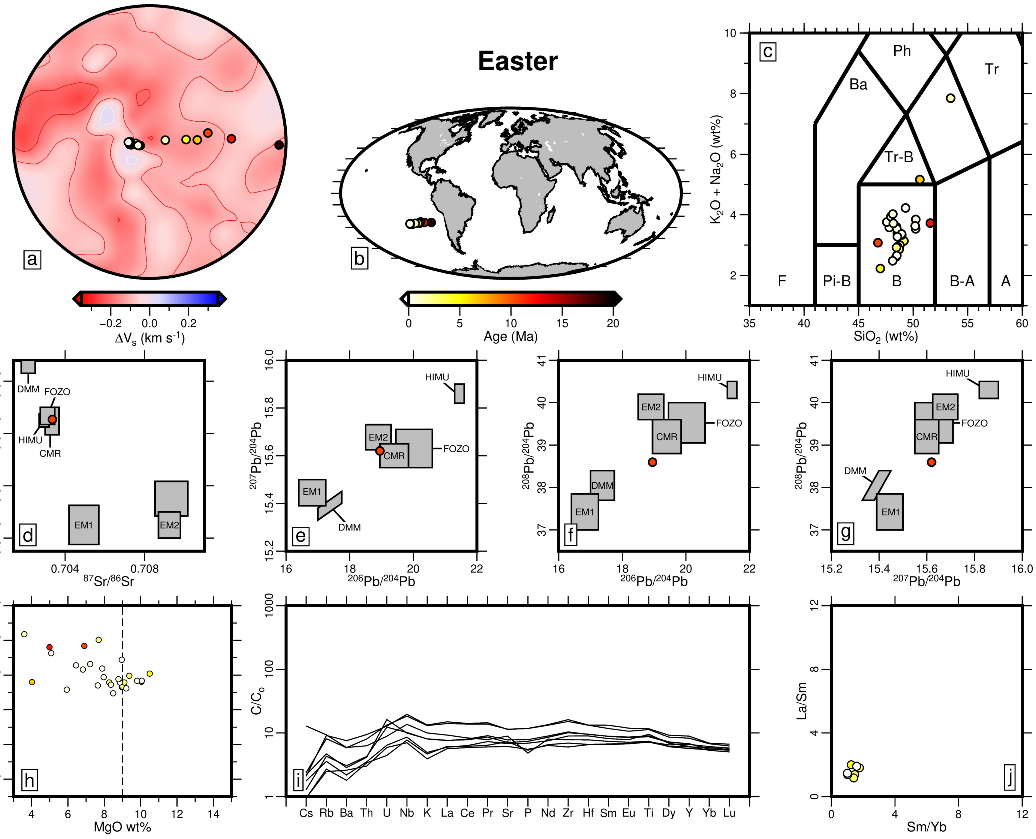
Easter
a
ΔVs (km s-1)
−0.2 0.0 0.2
b
Age (Ma)
0 5 10 15 20
c
Ph
Tr
Ba
Tr-B
F
Pi-B
B
B-A
A
K2O + Na2O (wt%)
SiO2 (wt%)
d
DMM
FOZO
HIMU
CMR
EM1
EM2
143Nd/144Nd
87Sr/86Sr
e
207Pb/204Pb
206Pb/204Pb
f
208Pb/204Pb
206Pb/204Pb
g
208Pb/204Pb
207Pb/204Pb
h
Al2O3 wt%
MgO wt%
i
C/Co
Cs Rb Ba Th U Nb K La Ce Pr Sr P Nd Zr Hf Sm Eu Ti Dy Y Yb Lu
j
La/Sm
Sm/Yb

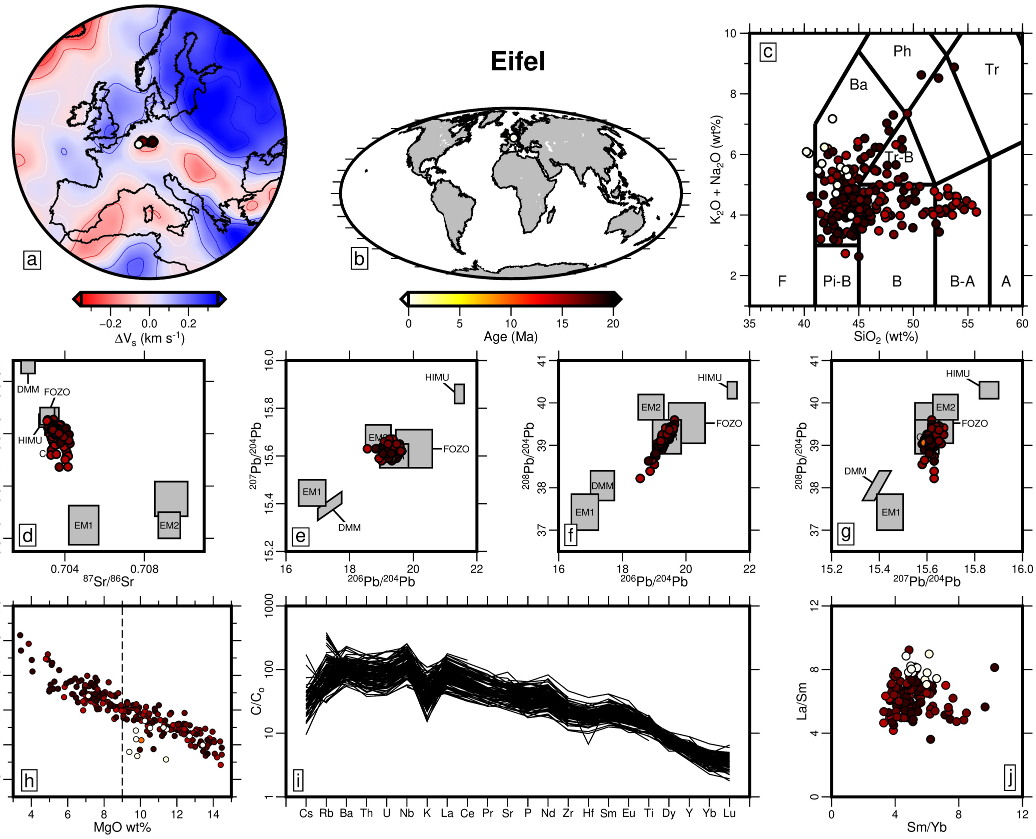
Eifel
a
ΔVs (km s-1)
−0.2 0.0 0.2
b
Age (Ma)
0 5 10 15 20
c
Ph
Tr
Ba
Tr-B
F
Pi-B
B
B-A
A
K2O + Na2O (wt%)
SiO2 (wt%)
d
DMM
FOZO
HIMU
EM1
EM2
143Nd/144Nd
87Sr/86Sr
e
207Pb/204Pb
206Pb/204Pb
f
208Pb/204Pb
206Pb/204Pb
g
208Pb/204Pb
207Pb/204Pb
h
Al2O3 wt%
MgO wt%
i
C/Co
Cs Rb Ba Th U Nb K La Ce Pr Sr P Nd Zr Hf Sm Eu Ti Dy Y Yb Lu
j
La/Sm
Sm/Yb

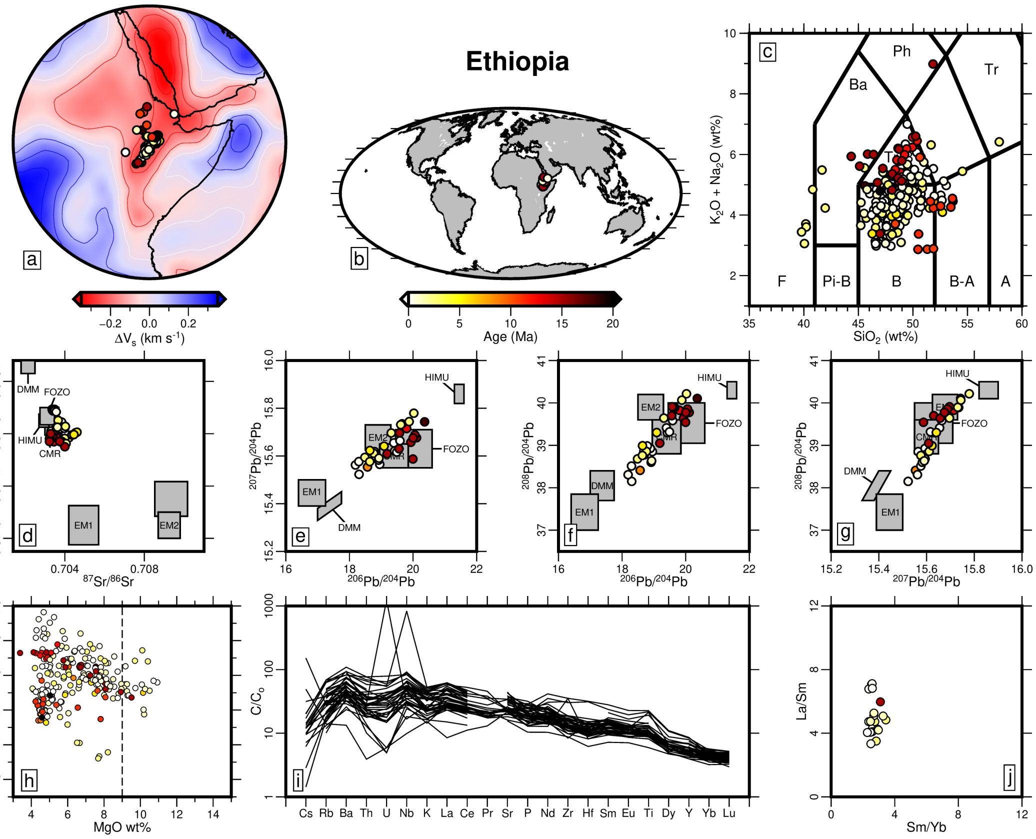
Ethiopia
a
b
c
d
e
f
g
h
i
j
ΔVs (km s-1)
Age (Ma)
SiO2 (wt%)
K2O + Na2O (wt%)
F
Pi-B
B
B-A
A
Ba
Ph
Tr
DMM
FOZO
HIMU
CMR
EM1
EM2
87Sr/86Sr
143Nd/144Nd
206Pb/204Pb
207Pb/204Pb
208Pb/204Pb
Al2O3 wt%
MgO wt%
C/Co
Cs Rb Ba Th U Nb K La Ce Pr Sr P Nd Zr Hf Sm Eu Ti Dy Y Yb Lu
La/Sm
Sm/Yb

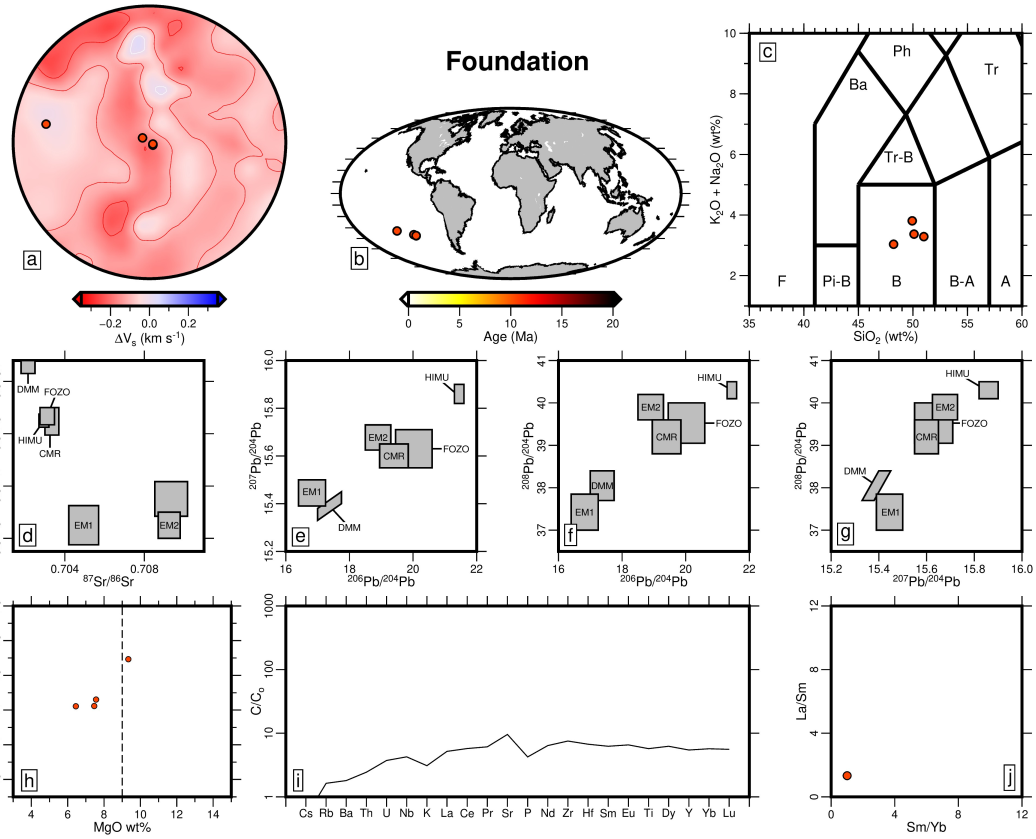
Foundation
a
b
c
d
e
f
g
h
i
j
ΔVs (km s-1)
Age (Ma)
SiO2 (wt%)
K2O + Na2O (wt%)
F
Pi-B
B
B-A
A
Ba
Ph
Tr
Tr-B
DMM
FOZO
HIMU
CMR
EM1
EM2
87Sr/86Sr
143Nd/144Nd
206Pb/204Pb
207Pb/204Pb
208Pb/204Pb
Al2O3 wt%
MgO wt%
C/Co
Cs Rb Ba Th U Nb K La Ce Pr Sr P Nd Zr Hf Sm Eu Ti Dy Y Yb Lu
La/Sm
Sm/Yb

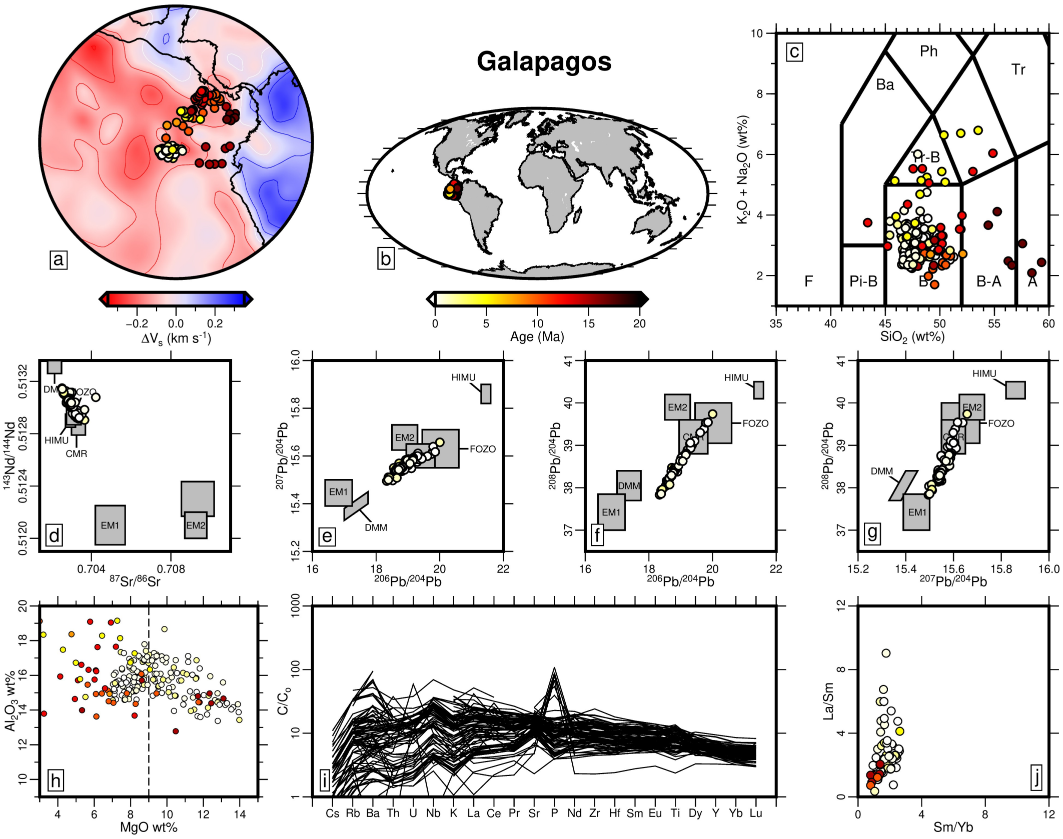
Galapagos
a
b
c
d
e
f
g
h
i
j
ΔVs (km s-1)
Age (Ma)
Ph
Tr
Ba
Tr-B
F
Pi-B
B
B-A
A
K2O + Na2O (wt%)
SiO2 (wt%)
143Nd/144Nd
87Sr/86Sr
207Pb/204Pb
206Pb/204Pb
208Pb/204Pb
207Pb/204Pb
HIMU
EM1
EM2
DMM
FOZO
CMR
Al2O3 wt%
MgO wt%
C/Co
Cs Rb Ba Th U Nb K La Ce Pr Sr P Nd Zr Hf Sm Eu Ti Dy Y Yb Lu
La/Sm
Sm/Yb

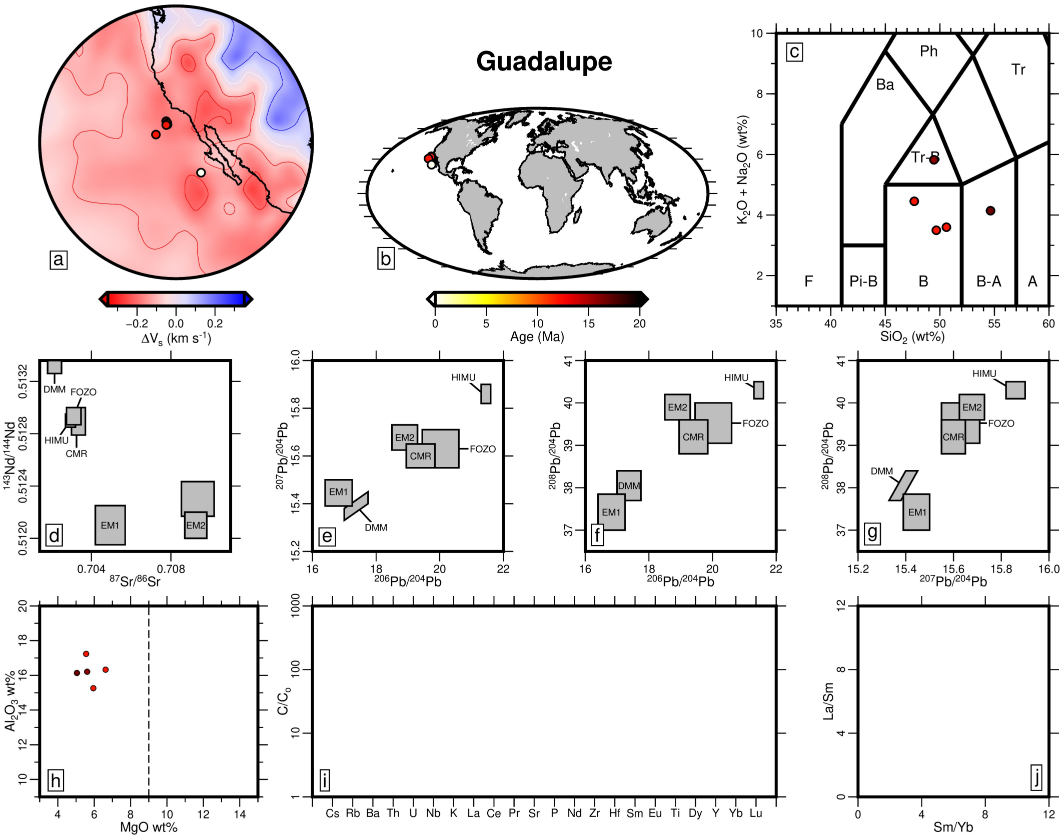
Guadalupe
a
b
c
d
e
f
g
h
i
j
ΔVs (km s-1)
Age (Ma)
Ph
Tr
Ba
Tr-B
F
Pi-B
B
B-A
A
K2O + Na2O (wt%)
SiO2 (wt%)
143Nd/144Nd
87Sr/86Sr
207Pb/204Pb
206Pb/204Pb
208Pb/204Pb
207Pb/204Pb
HIMU
EM1
EM2
DMM
FOZO
CMR
Al2O3 wt%
MgO wt%
C/Co
Cs Rb Ba Th U Nb K La Ce Pr Sr P Nd Zr Hf Sm Eu Ti Dy Y Yb Lu
La/Sm
Sm/Yb

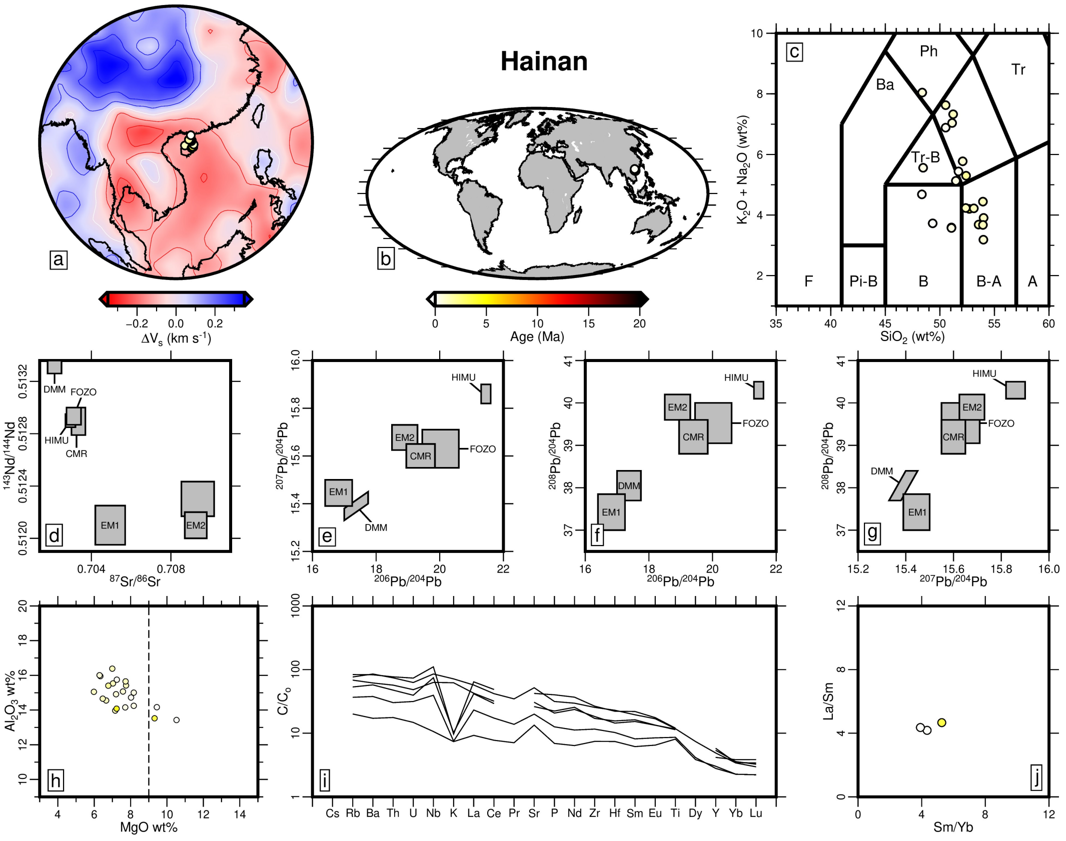
Hainan
a
b
c
d
e
f
g
h
i
j
ΔVs (km s-1)
Age (Ma)
K2O + Na2O (wt%)
SiO2 (wt%)
F
Pi-B
B
B-A
A
Ba
Ph
Tr
Tr-B
143Nd/144Nd
87Sr/86Sr
207Pb/204Pb
206Pb/204Pb
208Pb/204Pb
DMM
FOZO
HIMU
CMR
EM1
EM2
Al2O3 wt%
MgO wt%
C/Co
Cs Rb Ba Th U Nb K La Ce Pr Sr P Nd Zr Hf Sm Eu Ti Dy Y Yb Lu
La/Sm
Sm/Yb

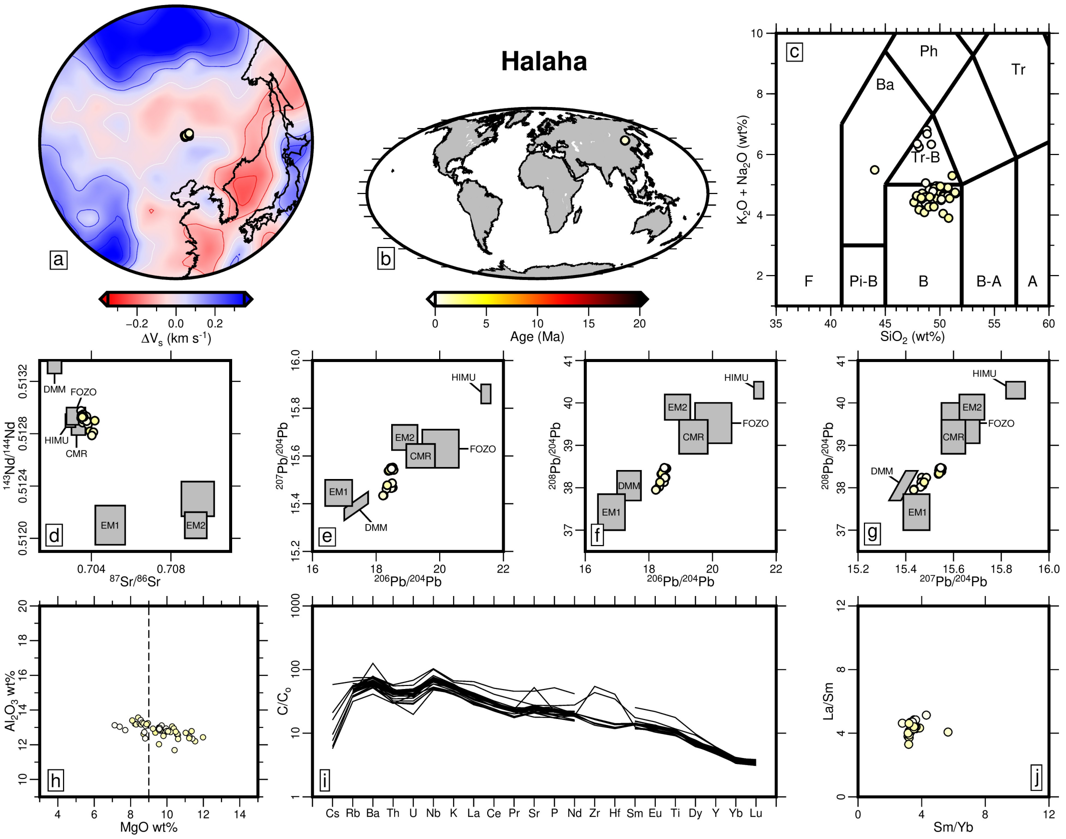
Halaha
a
b
c
d
e
f
g
h
i
j
ΔVs (km s-1)
Age (Ma)
K2O + Na2O (wt%)
SiO2 (wt%)
F
Pi-B
B
B-A
A
Ba
Ph
Tr
Tr-B
143Nd/144Nd
87Sr/86Sr
207Pb/204Pb
206Pb/204Pb
208Pb/204Pb
DMM
FOZO
HIMU
CMR
EM1
EM2
Al2O3 wt%
MgO wt%
C/Co
Cs Rb Ba Th U Nb K La Ce Pr Sr P Nd Zr Hf Sm Eu Ti Dy Y Yb Lu
La/Sm
Sm/Yb

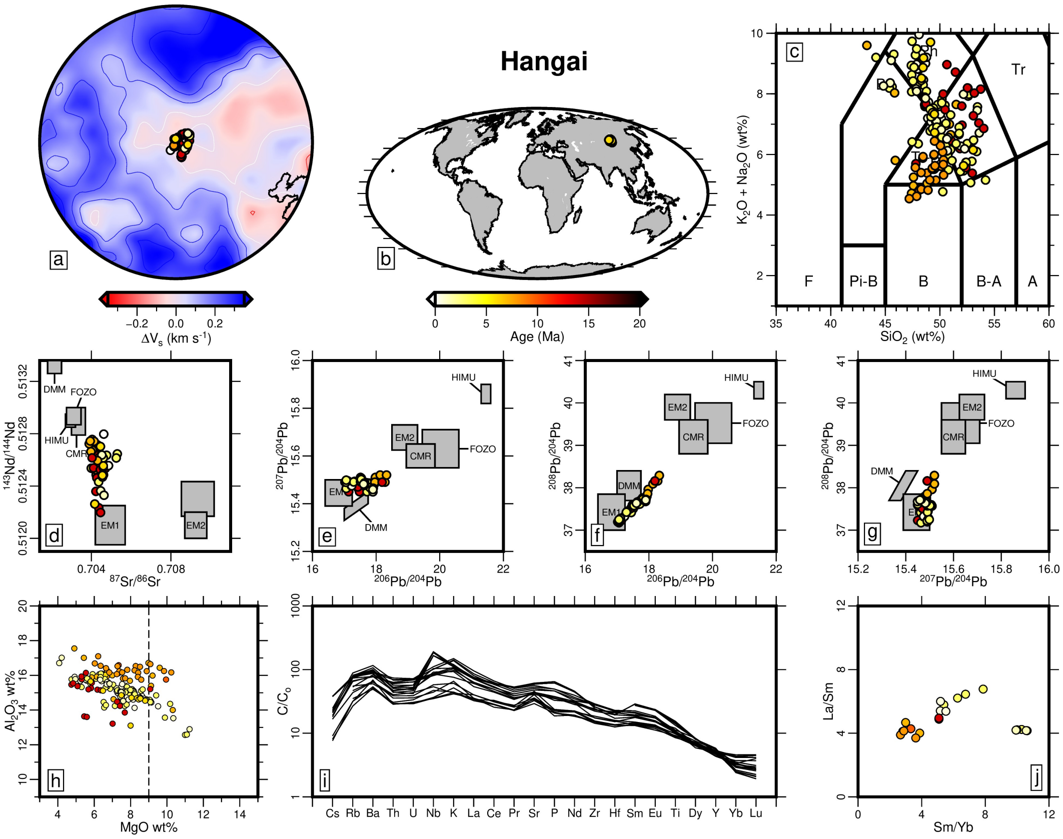
Hangai
a
b
c
ΔVs (km s-1)
Age (Ma)
SiO2 (wt%)
K2O + Na2O (wt%)
F
Pi-B
B
B-A
A
Tr
d
e
f
g
DMM
FOZO
HIMU
CMR
EM1
EM2
143Nd/144Nd
87Sr/86Sr
207Pb/204Pb
206Pb/204Pb
208Pb/204Pb
h
i
j
Al2O3 wt%
MgO wt%
C/Co
Cs Rb Ba Th U Nb K La Ce Pr Sr P Nd Zr Hf Sm Eu Ti Dy Y Yb Lu
La/Sm
Sm/Yb

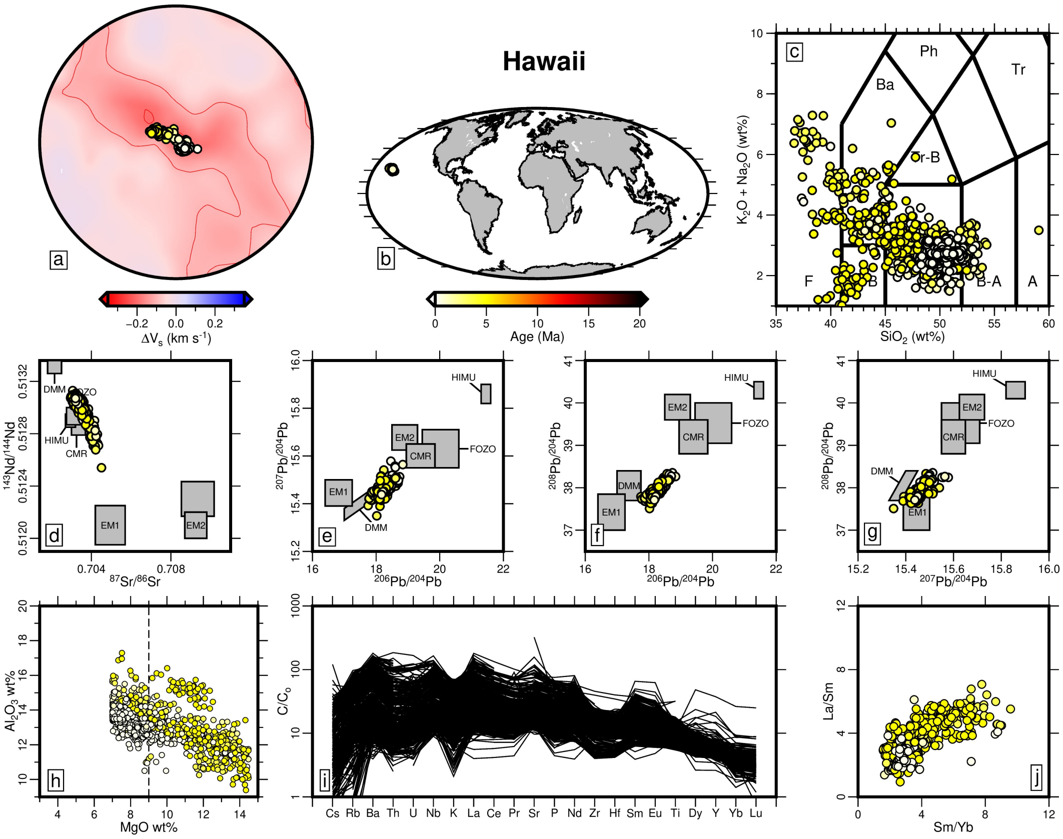
Hawaii
a
b
c
ΔVs (km s-1)
Age (Ma)
SiO2 (wt%)
K2O + Na2O (wt%)
F
B
B-A
A
Ba
Ph
Tr
Tr-B
d
e
f
g
DMM
FOZO
HIMU
CMR
EM1
EM2
143Nd/144Nd
87Sr/86Sr
207Pb/204Pb
206Pb/204Pb
208Pb/204Pb
h
i
j
Al2O3 wt%
MgO wt%
C/Co
Cs Rb Ba Th U Nb K La Ce Pr Sr P Nd Zr Hf Sm Eu Ti Dy Y Yb Lu
La/Sm
Sm/Yb

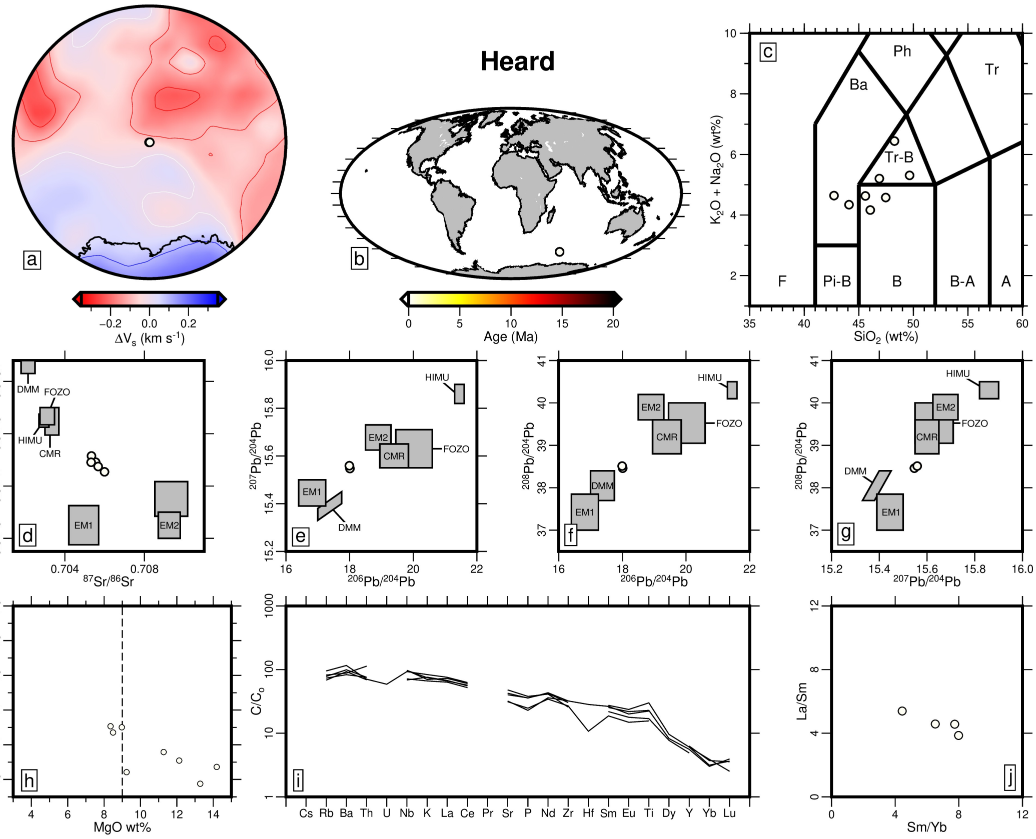
Heard
a
b
c
ΔVs (km s-1)
Age (Ma)
Ph
Tr
Ba
Tr-B
F
Pi-B
B
B-A
A
SiO2 (wt%)
K2O + Na2O (wt%)
DMM
FOZO
HIMU
CMR
EM1
EM2
d
e
f
g
h
i
j
87Sr/86Sr
143Nd/144Nd
206Pb/204Pb
207Pb/204Pb
208Pb/204Pb
Al2O3 wt%
MgO wt%
C/Co
Cs Rb Ba Th U Nb K La Ce Pr Sr P Nd Zr Hf Sm Eu Ti Dy Y Yb Lu
La/Sm
Sm/Yb

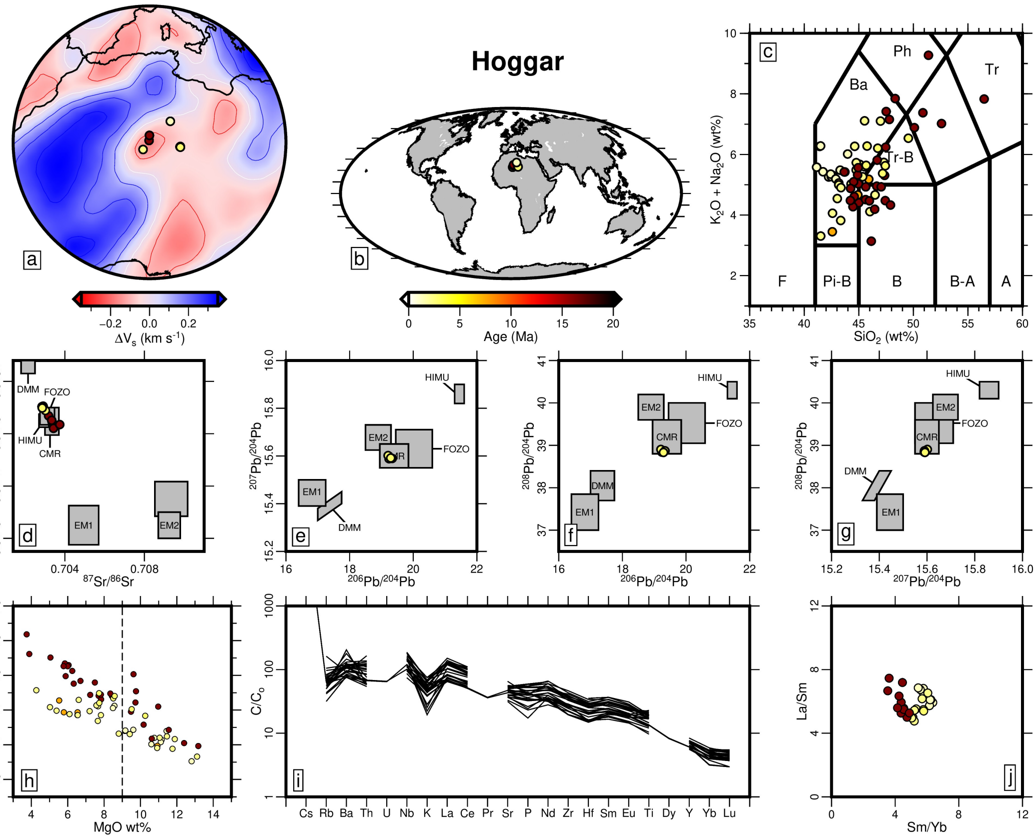
Hoggar
a
b
c
ΔVs (km s-1)
Age (Ma)
Ph
Tr
Ba
Tr-B
F
Pi-B
B
B-A
A
SiO2 (wt%)
K2O + Na2O (wt%)
DMM
FOZO
HIMU
CMR
EM1
EM2
d
e
f
g
h
i
j
87Sr/86Sr
143Nd/144Nd
206Pb/204Pb
207Pb/204Pb
208Pb/204Pb
Al2O3 wt%
MgO wt%
C/Co
Cs Rb Ba Th U Nb K La Ce Pr Sr P Nd Zr Hf Sm Eu Ti Dy Y Yb Lu
La/Sm
Sm/Yb

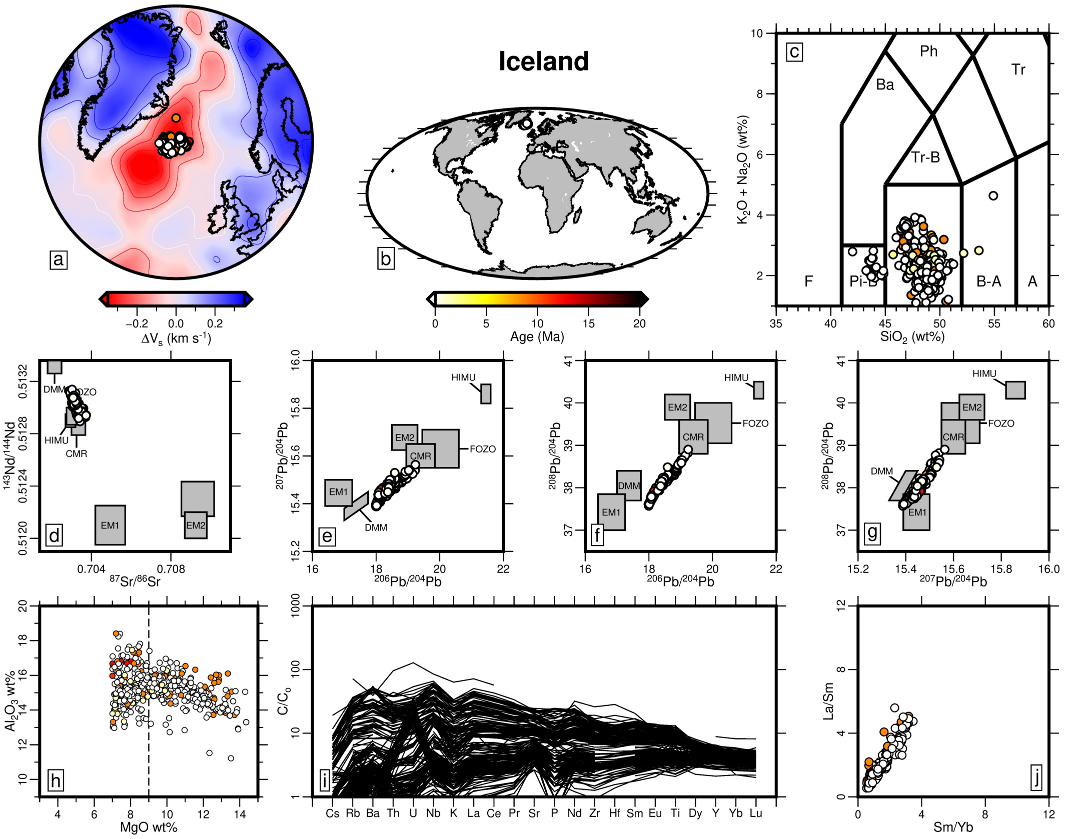
Iceland
a
ΔVs (km s-1)
b
Age (Ma)
c
K2O + Na2O (wt%)
SiO2 (wt%)
Ph
Tr
Ba
Tr-B
F
Pi-B
B-A
A
d
143Nd/144Nd
87Sr/86Sr
DMM
FOZO
HIMU
CMR
EM1
EM2
e
207Pb/204Pb
206Pb/204Pb
f
208Pb/204Pb
206Pb/204Pb
g
208Pb/204Pb
207Pb/204Pb
h
Al2O3 wt%
MgO wt%
i
C/Co
Cs Rb Ba Th U Nb K La Ce Pr Sr P Nd Zr Hf Sm Eu Ti Dy Y Yb Lu
j
La/Sm
Sm/Yb

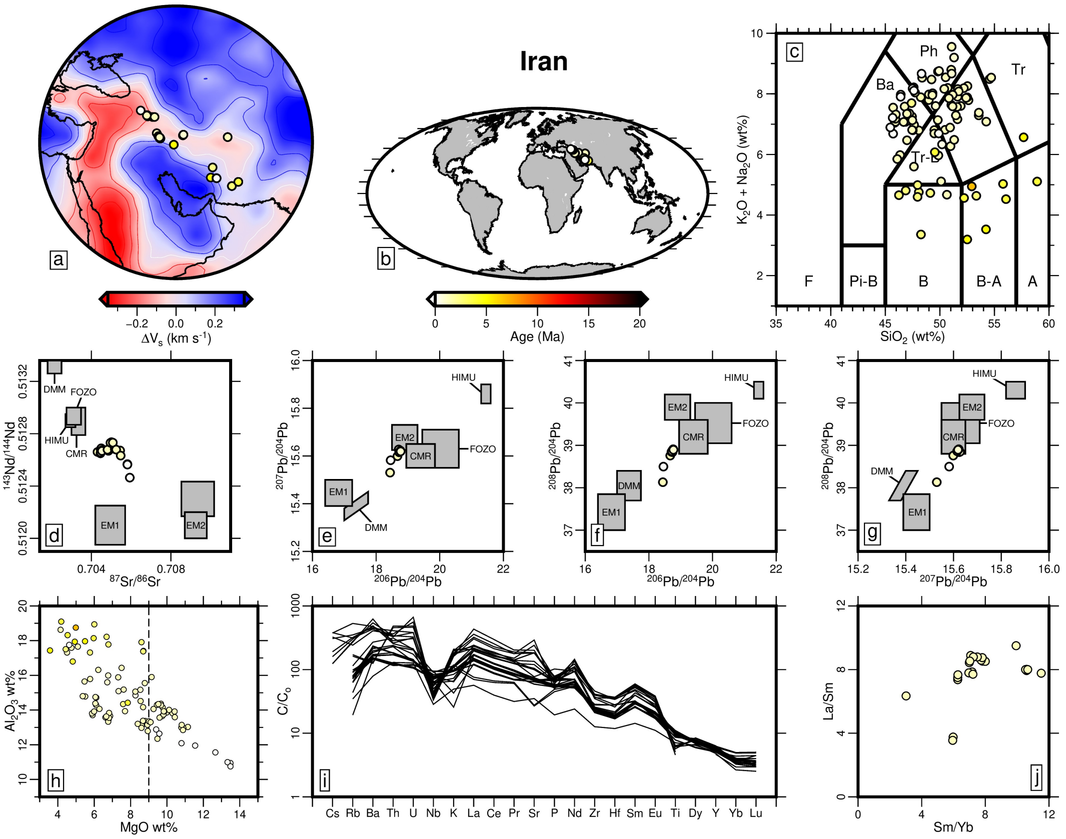
Iran
a
ΔVs (km s-1)
b
Age (Ma)
c
K2O + Na2O (wt%)
SiO2 (wt%)
Ph
Tr
Ba
Tr-B
F
Pi-B
B
B-A
A
d
143Nd/144Nd
87Sr/86Sr
DMM
FOZO
HIMU
CMR
EM1
EM2
e
207Pb/204Pb
206Pb/204Pb
f
208Pb/204Pb
206Pb/204Pb
g
208Pb/204Pb
207Pb/204Pb
h
Al2O3 wt%
MgO wt%
i
C/Co
Cs Rb Ba Th U Nb K La Ce Pr Sr P Nd Zr Hf Sm Eu Ti Dy Y Yb Lu
j
La/Sm
Sm/Yb

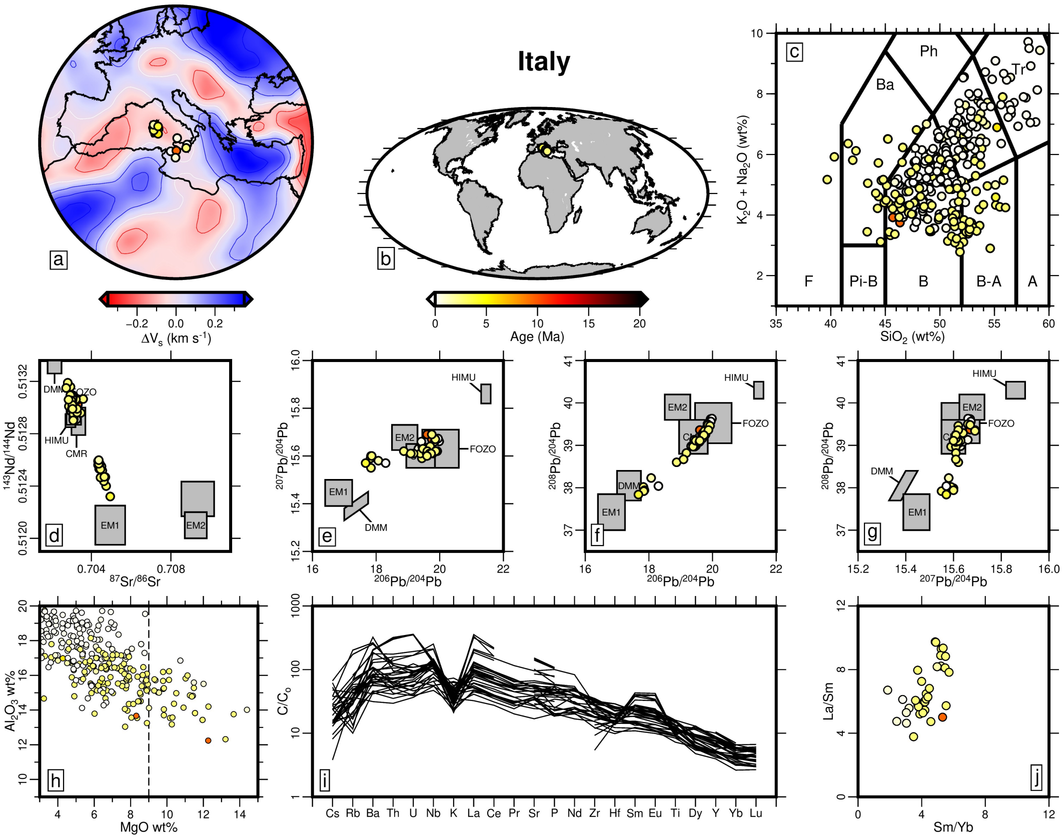
Italy
a
b
c
d
e
f
g
h
i
j
ΔVs (km s-1)
Age (Ma)
K2O + Na2O (wt%)
SiO2 (wt%)
F
Pi-B
B
B-A
A
Ba
Ph
Tr
143Nd/144Nd
87Sr/86Sr
207Pb/204Pb
206Pb/204Pb
208Pb/204Pb
207Pb/204Pb
DMM
FOZO
HIMU
CMR
EM1
EM2
Al2O3 wt%
MgO wt%
C/Co
Cs Rb Ba Th U Nb K La Ce Pr Sr P Nd Zr Hf Sm Eu Ti Dy Y Yb Lu
La/Sm
Sm/Yb

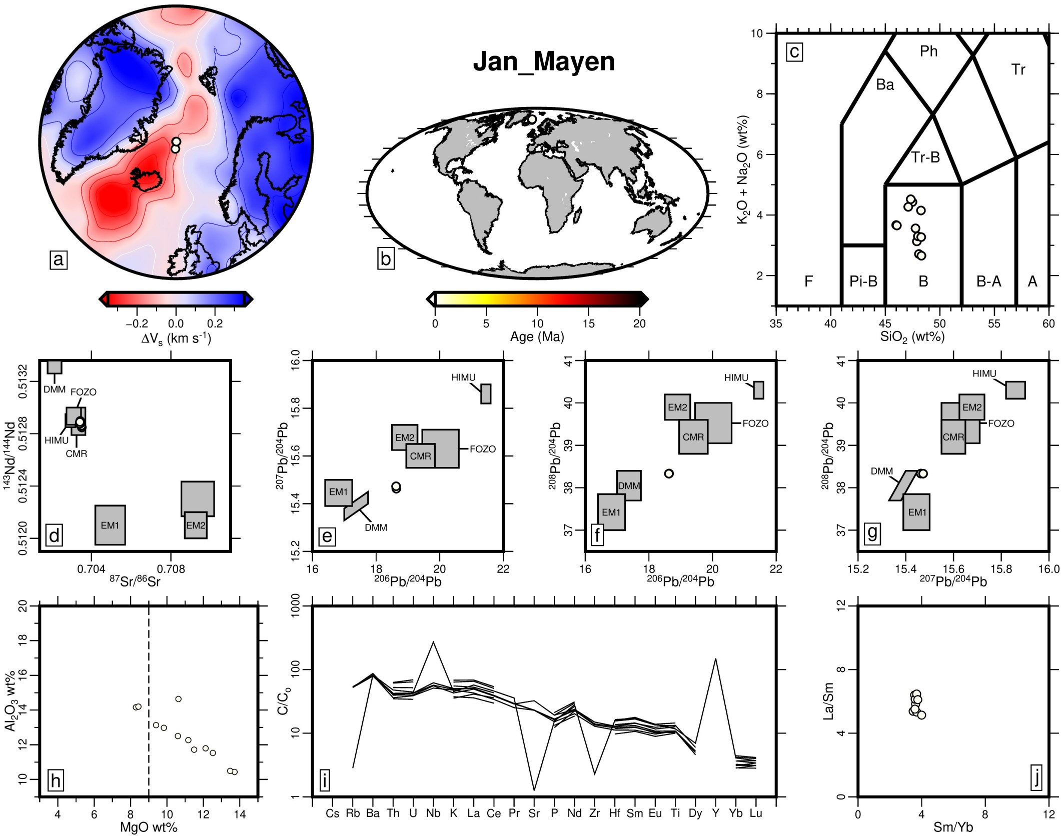
Jan_Mayen
a
b
c
d
e
f
g
h
i
j
ΔVs (km s-1)
Age (Ma)
K2O + Na2O (wt%)
SiO2 (wt%)
F
Pi-B
B
B-A
A
Ba
Ph
Tr
Tr-B
143Nd/144Nd
87Sr/86Sr
207Pb/204Pb
206Pb/204Pb
208Pb/204Pb
207Pb/204Pb
DMM
FOZO
HIMU
CMR
EM1
EM2
Al2O3 wt%
MgO wt%
C/Co
Cs Rb Ba Th U Nb K La Ce Pr Sr P Nd Zr Hf Sm Eu Ti Dy Y Yb Lu
La/Sm
Sm/Yb

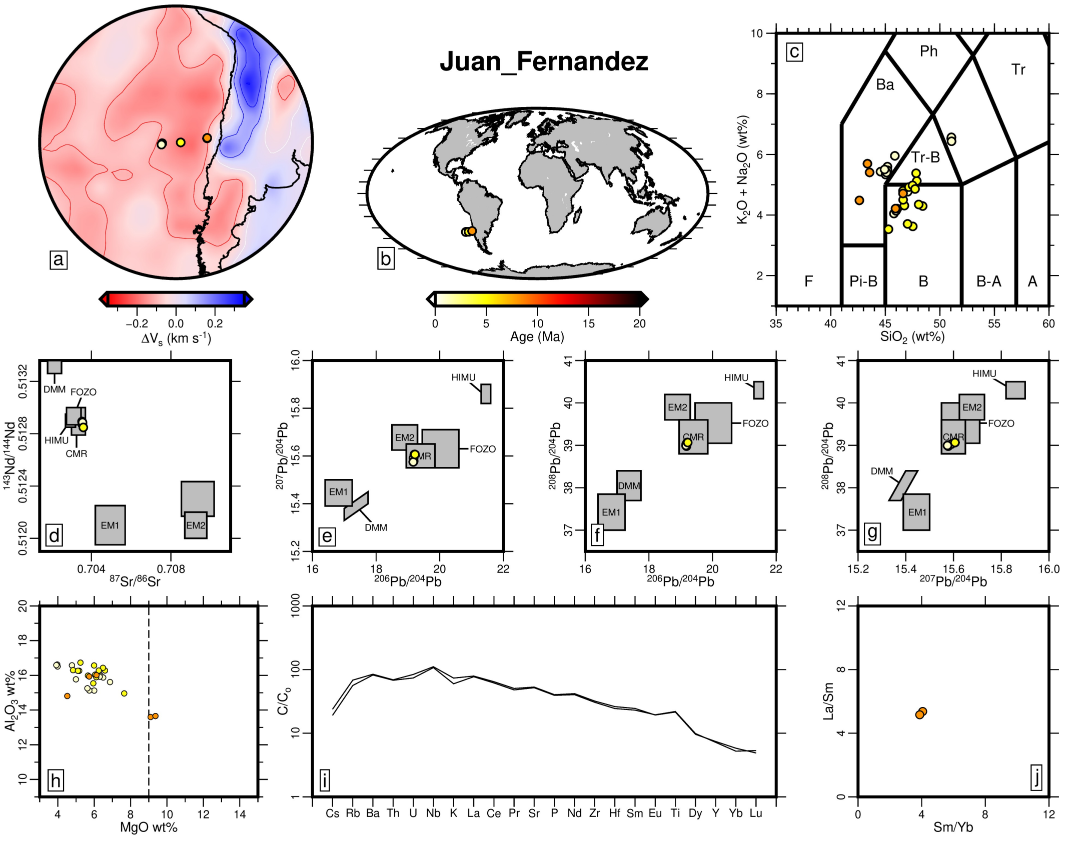
Juan_Fernandez
a
ΔVs (km s-1)
b
Age (Ma)
c
Ph
Tr
Ba
Tr-B
F
Pi-B
B
B-A
A
K2O + Na2O (wt%)
SiO2 (wt%)
d
DMM
FOZO
HIMU
CMR
EM1
EM2
143Nd/144Nd
87Sr/86Sr
e
207Pb/204Pb
206Pb/204Pb
f
208Pb/204Pb
206Pb/204Pb
g
208Pb/204Pb
207Pb/204Pb
h
Al2O3 wt%
MgO wt%
i
C/Co
Cs Rb Ba Th U Nb K La Ce Pr Sr P Nd Zr Hf Sm Eu Ti Dy Y Yb Lu
j
La/Sm
Sm/Yb

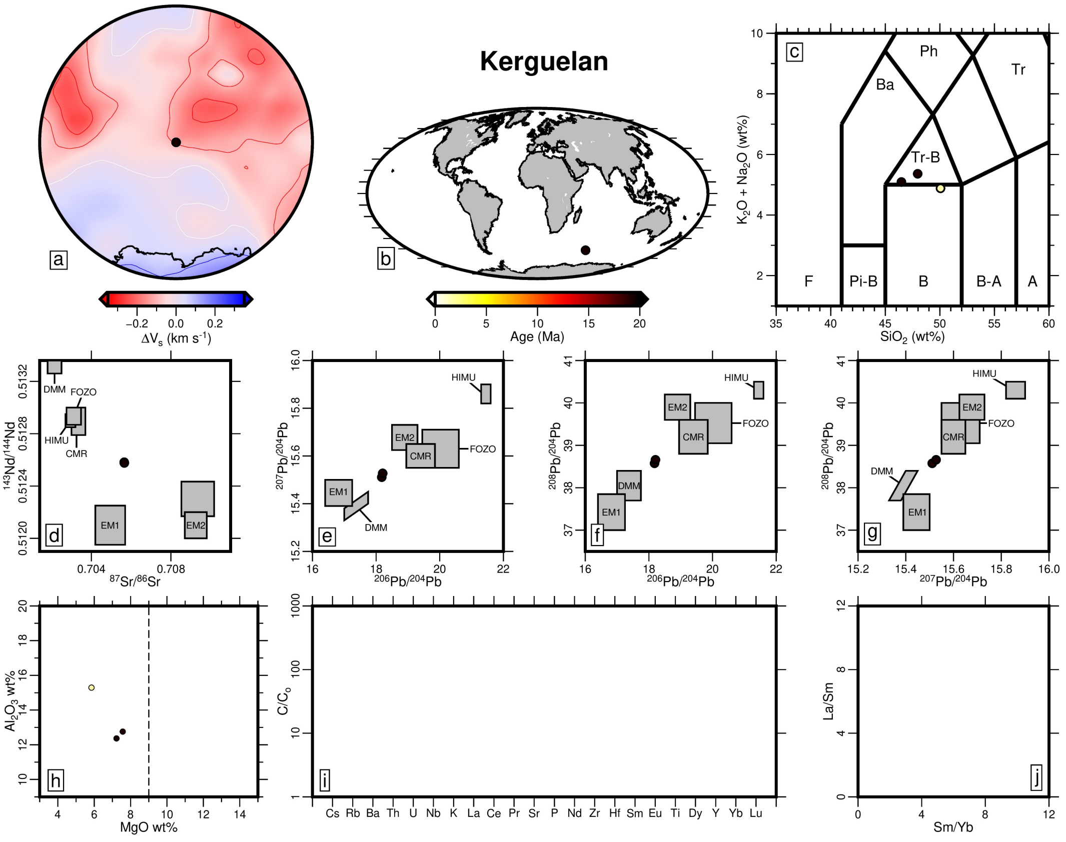
Kerguelan
a
ΔVs (km s-1)
b
Age (Ma)
c
Ph
Tr
Ba
Tr-B
F
Pi-B
B
B-A
A
K2O + Na2O (wt%)
SiO2 (wt%)
d
DMM
FOZO
HIMU
CMR
EM1
EM2
143Nd/144Nd
87Sr/86Sr
e
207Pb/204Pb
206Pb/204Pb
f
208Pb/204Pb
206Pb/204Pb
g
208Pb/204Pb
207Pb/204Pb
h
Al2O3 wt%
MgO wt%
i
C/Co
Cs Rb Ba Th U Nb K La Ce Pr Sr P Nd Zr Hf Sm Eu Ti Dy Y Yb Lu
j
La/Sm
Sm/Yb

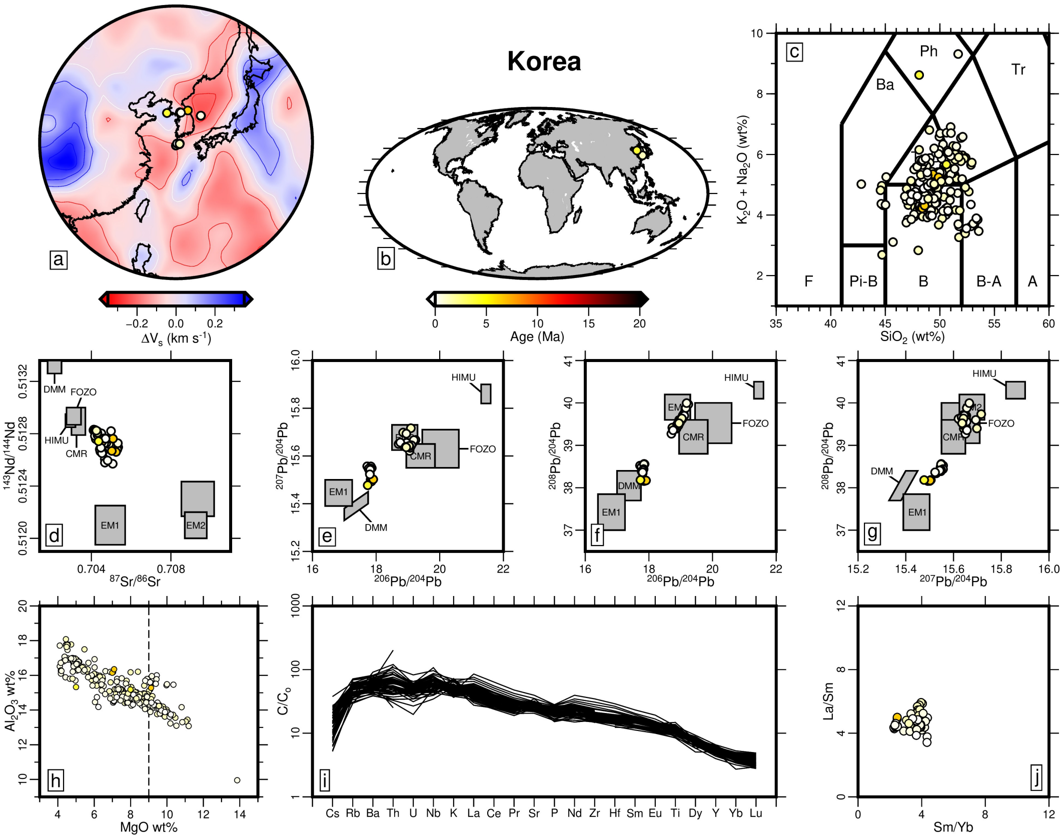
Korea
a
b
c
d
e
f
g
h
i
j
ΔVs (km s-1)
Age (Ma)
K2O + Na2O (wt%)
SiO2 (wt%)
Ph
Tr
Ba
F
Pi-B
B
B-A
A
143Nd/144Nd
87Sr/86Sr
207Pb/204Pb
206Pb/204Pb
208Pb/204Pb
207Pb/204Pb
DMM
FOZO
HIMU
CMR
EM1
EM2
Al2O3 wt%
MgO wt%
C/Co
Cs Rb Ba Th U Nb K La Ce Pr Sr P Nd Zr Hf Sm Eu Ti Dy Y Yb Lu
La/Sm
Sm/Yb

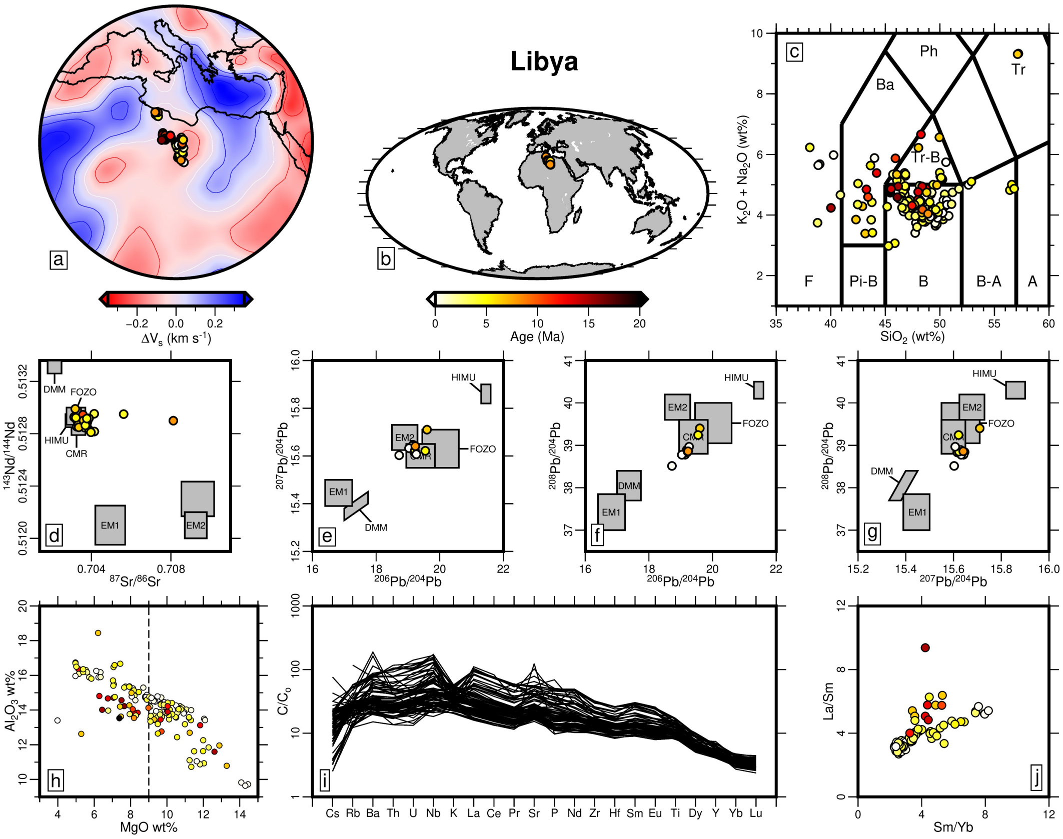
Libya
a
b
c
d
e
f
g
h
i
j
ΔVs (km s-1)
Age (Ma)
K2O + Na2O (wt%)
SiO2 (wt%)
Ph
Tr
Ba
Tr-B
F
Pi-B
B
B-A
A
143Nd/144Nd
87Sr/86Sr
207Pb/204Pb
206Pb/204Pb
208Pb/204Pb
207Pb/204Pb
DMM
FOZO
HIMU
CMR
EM1
EM2
Al2O3 wt%
MgO wt%
C/Co
Cs Rb Ba Th U Nb K La Ce Pr Sr P Nd Zr Hf Sm Eu Ti Dy Y Yb Lu
La/Sm
Sm/Yb

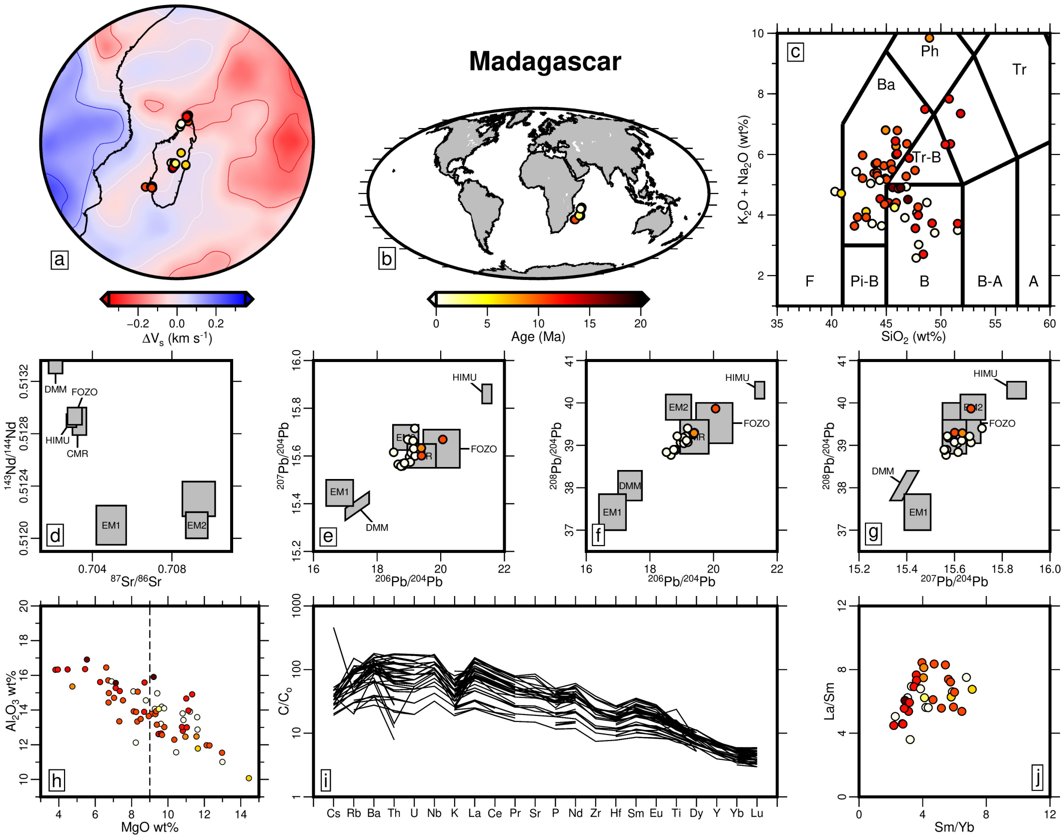
Madagascar
a
b
c
ΔVs (km s-1)
Age (Ma)
SiO2 (wt%)
K2O + Na2O (wt%)
Ph
Tr
Ba
Tr-B
F
Pi-B
B
B-A
A
143Nd/144Nd
87Sr/86Sr
DMM
FOZO
HIMU
CMR
EM1
EM2
207Pb/204Pb
206Pb/204Pb
208Pb/204Pb
Al2O3 wt%
MgO wt%
C/Co
Cs Rb Ba Th U Nb K La Ce Pr Sr P Nd Zr Hf Sm Eu Ti Dy Y Yb Lu
La/Sm
Sm/Yb

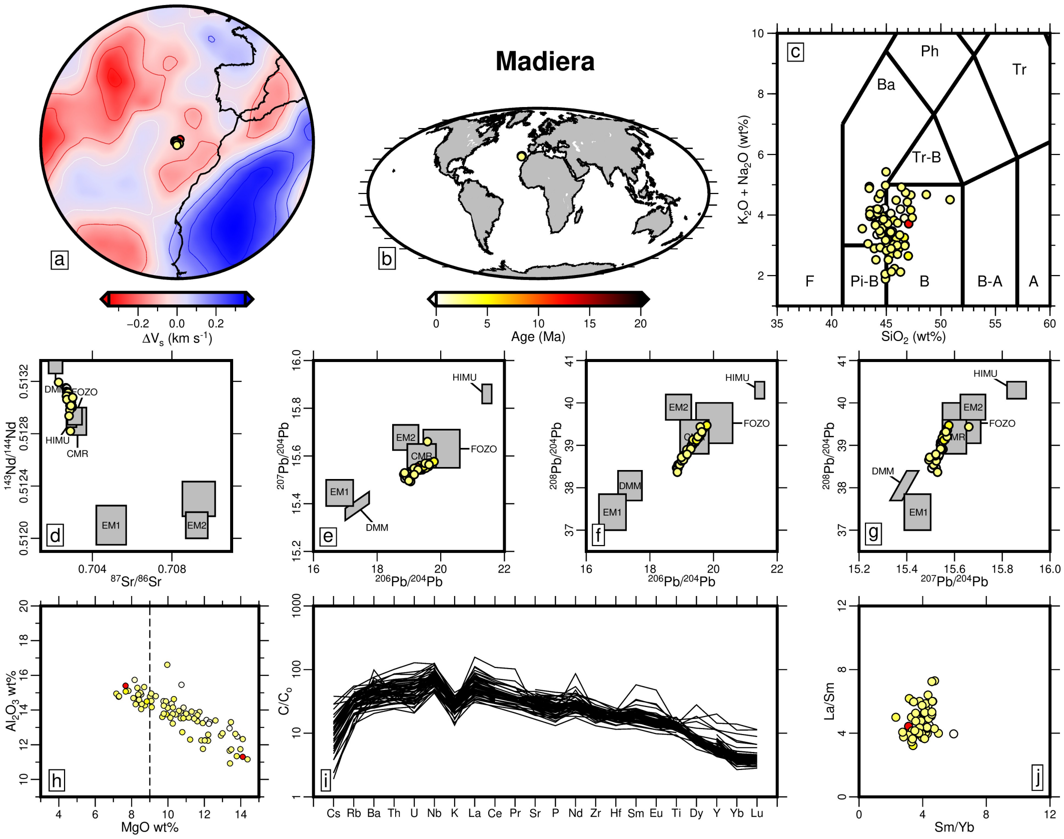
Madiera
a
b
c
ΔVs (km s-1)
Age (Ma)
SiO2 (wt%)
K2O + Na2O (wt%)
Ph
Tr
Ba
Tr-B
F
Pi-B
B
B-A
A
143Nd/144Nd
87Sr/86Sr
DMM
FOZO
HIMU
CMR
EM1
EM2
207Pb/204Pb
206Pb/204Pb
208Pb/204Pb
Al2O3 wt%
MgO wt%
C/Co
Cs Rb Ba Th U Nb K La Ce Pr Sr P Nd Zr Hf Sm Eu Ti Dy Y Yb Lu
La/Sm
Sm/Yb

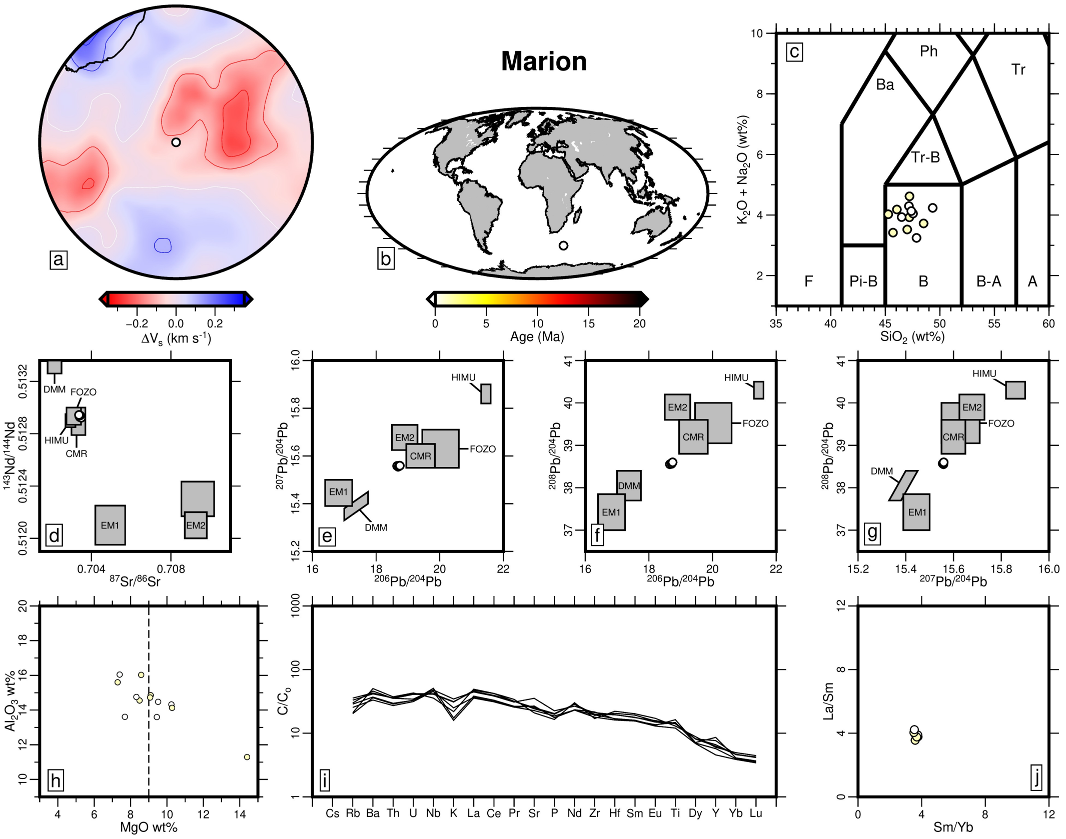
Marion
a
b
c
ΔVs (km s-1)
Age (Ma)
K2O + Na2O (wt%)
SiO2 (wt%)
Ph
Tr
Ba
Tr-B
F
Pi-B
B
B-A
A
143Nd/144Nd
87Sr/86Sr
DMM
FOZO
HIMU
CMR
EM1
EM2
207Pb/204Pb
206Pb/204Pb
208Pb/204Pb
Al2O3 wt%
MgO wt%
C/Co
Cs Rb Ba Th U Nb K La Ce Pr Sr P Nd Zr Hf Sm Eu Ti Dy Y Yb Lu
La/Sm
Sm/Yb

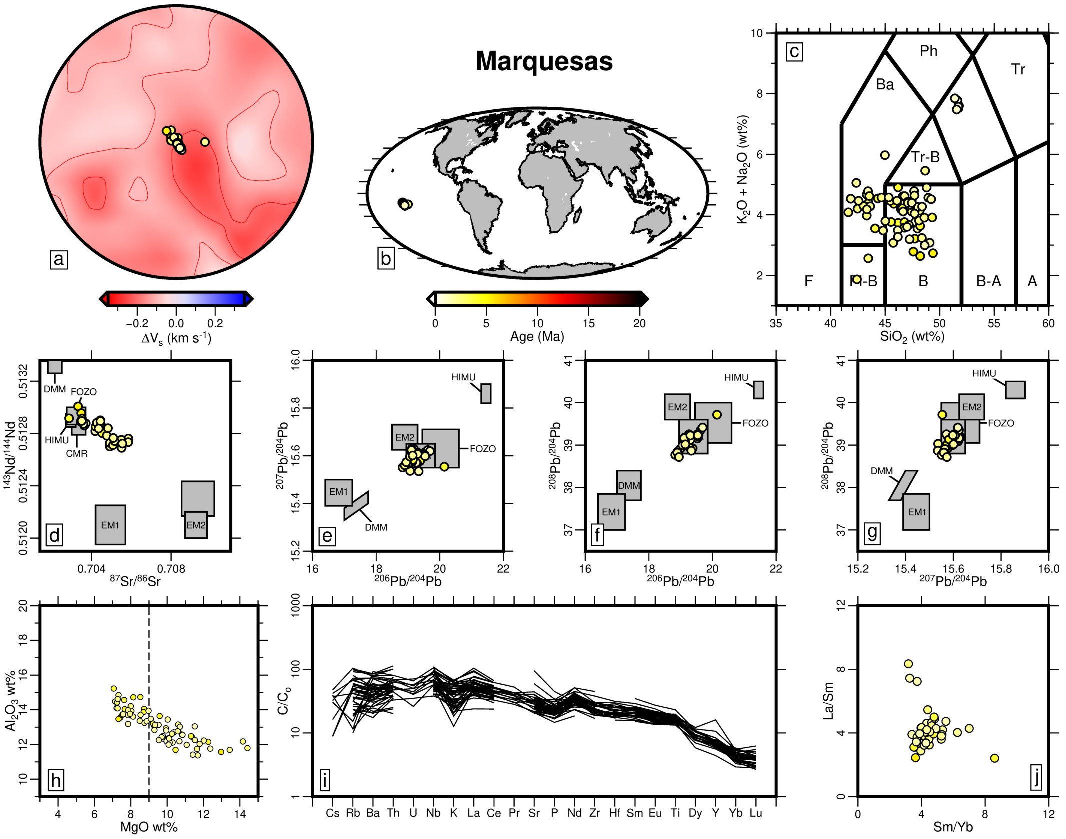
Marquesas
a
b
c
ΔVs (km s-1)
Age (Ma)
K2O + Na2O (wt%)
SiO2 (wt%)
Ph
Tr
Ba
Tr-B
F
Pi-B
B
B-A
A
143Nd/144Nd
87Sr/86Sr
DMM
FOZO
HIMU
CMR
EM1
EM2
207Pb/204Pb
206Pb/204Pb
208Pb/204Pb
Al2O3 wt%
MgO wt%
C/Co
Cs Rb Ba Th U Nb K La Ce Pr Sr P Nd Zr Hf Sm Eu Ti Dy Y Yb Lu
La/Sm
Sm/Yb

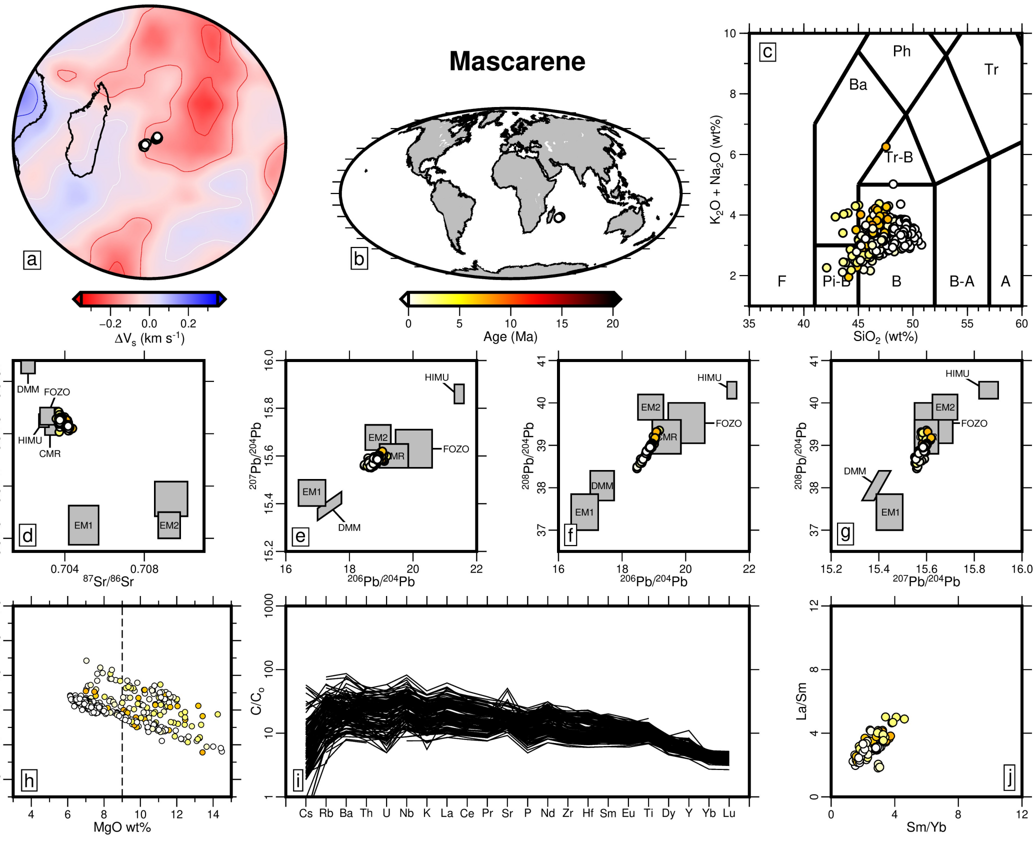
Mascarene
a
b
c
d
e
f
g
h
i
j
ΔVs (km s-1)
Age (Ma)
SiO2 (wt%)
K2O + Na2O (wt%)
Ph
Tr
Ba
Tr-B
F
Pi-B
B
B-A
A
DMM
FOZO
HIMU
CMR
EM1
EM2
87Sr/86Sr
143Nd/144Nd
206Pb/204Pb
207Pb/204Pb
208Pb/204Pb
MgO wt%
Al2O3 wt%
C/Co
Cs Rb Ba Th U Nb K La Ce Pr Sr P Nd Zr Hf Sm Eu Ti Dy Y Yb Lu
Sm/Yb
La/Sm

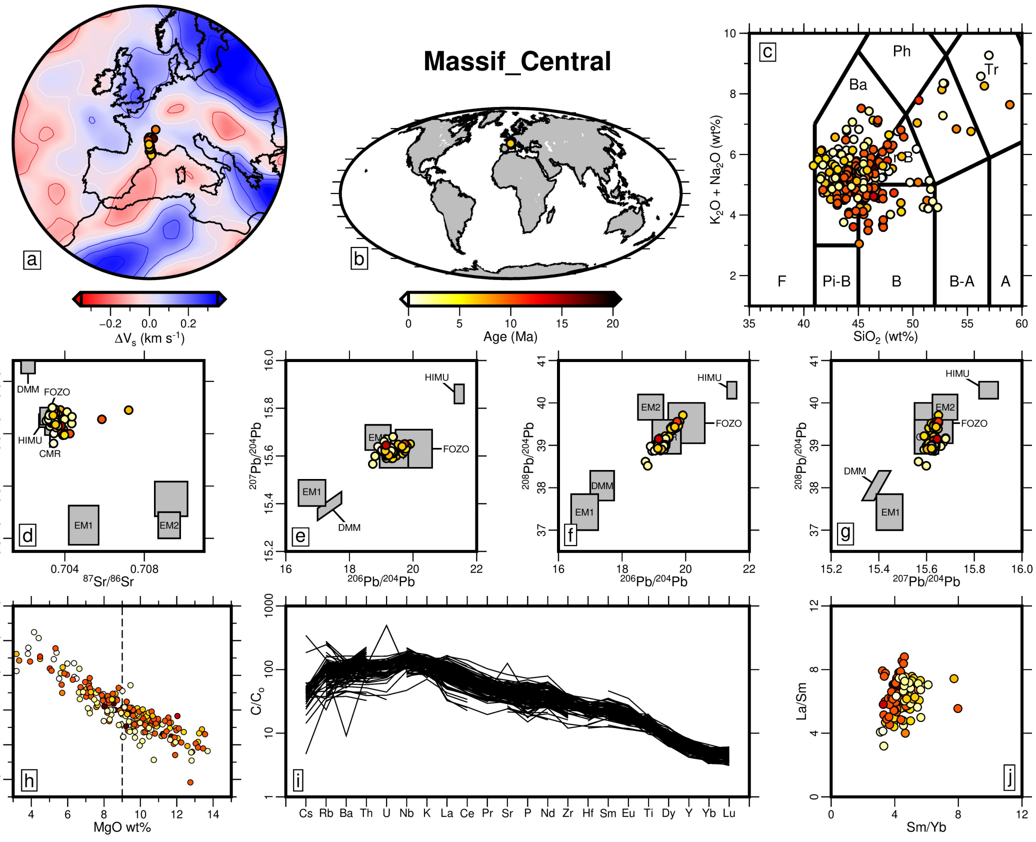
Massif_Central
a
b
c
d
e
f
g
h
i
j
ΔVs (km s-1)
Age (Ma)
SiO2 (wt%)
K2O + Na2O (wt%)
Ph
Tr
Ba
F
Pi-B
B
B-A
A
DMM
FOZO
HIMU
CMR
EM1
EM2
87Sr/86Sr
143Nd/144Nd
206Pb/204Pb
207Pb/204Pb
208Pb/204Pb
MgO wt%
Al2O3 wt%
C/Co
Cs Rb Ba Th U Nb K La Ce Pr Sr P Nd Zr Hf Sm Eu Ti Dy Y Yb Lu
Sm/Yb
La/Sm

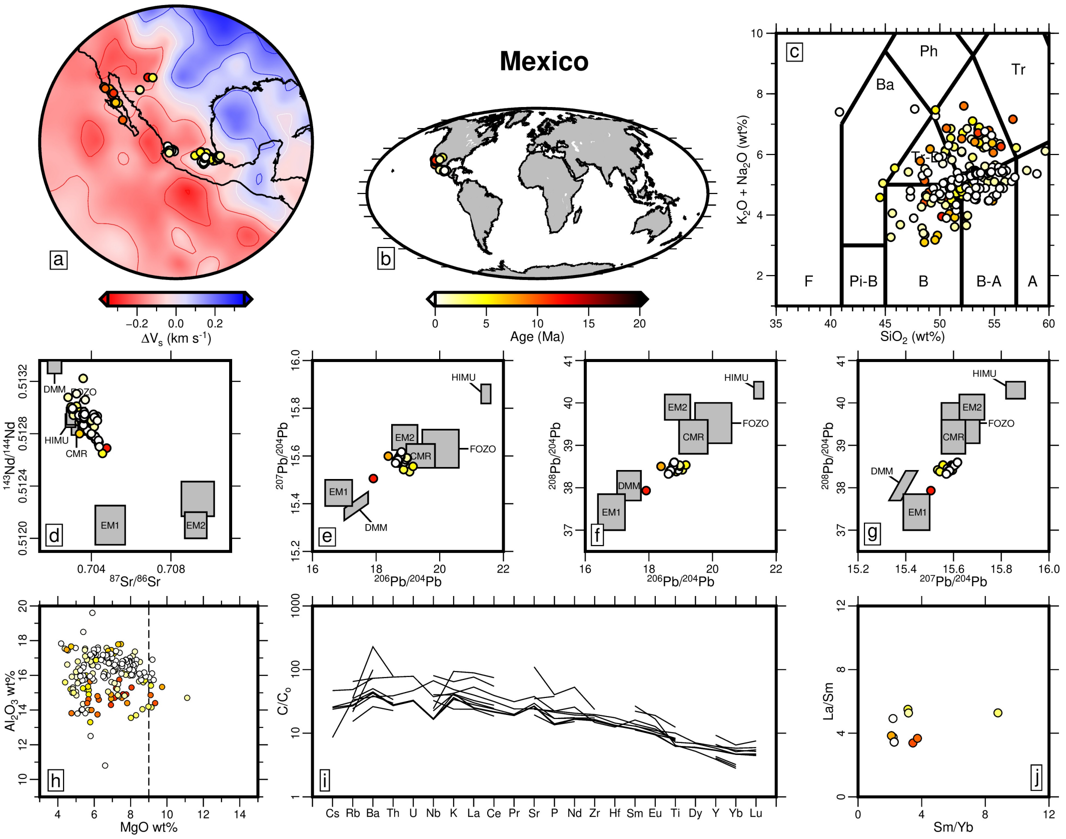
Mexico
a
b
c
ΔVs (km s-1)
Age (Ma)
SiO2 (wt%)
K2O + Na2O (wt%)
Ph
Tr
Ba
Tr-B
F
Pi-B
B
B-A
A
d
e
f
g
143Nd/144Nd
87Sr/86Sr
207Pb/204Pb
206Pb/204Pb
208Pb/204Pb
DMM
FOZO
HIMU
CMR
EM1
EM2
h
i
j
Al2O3 wt%
MgO wt%
C/Co
Cs Rb Ba Th U Nb K La Ce Pr Sr P Nd Zr Hf Sm Eu Ti Dy Y Yb Lu
La/Sm
Sm/Yb

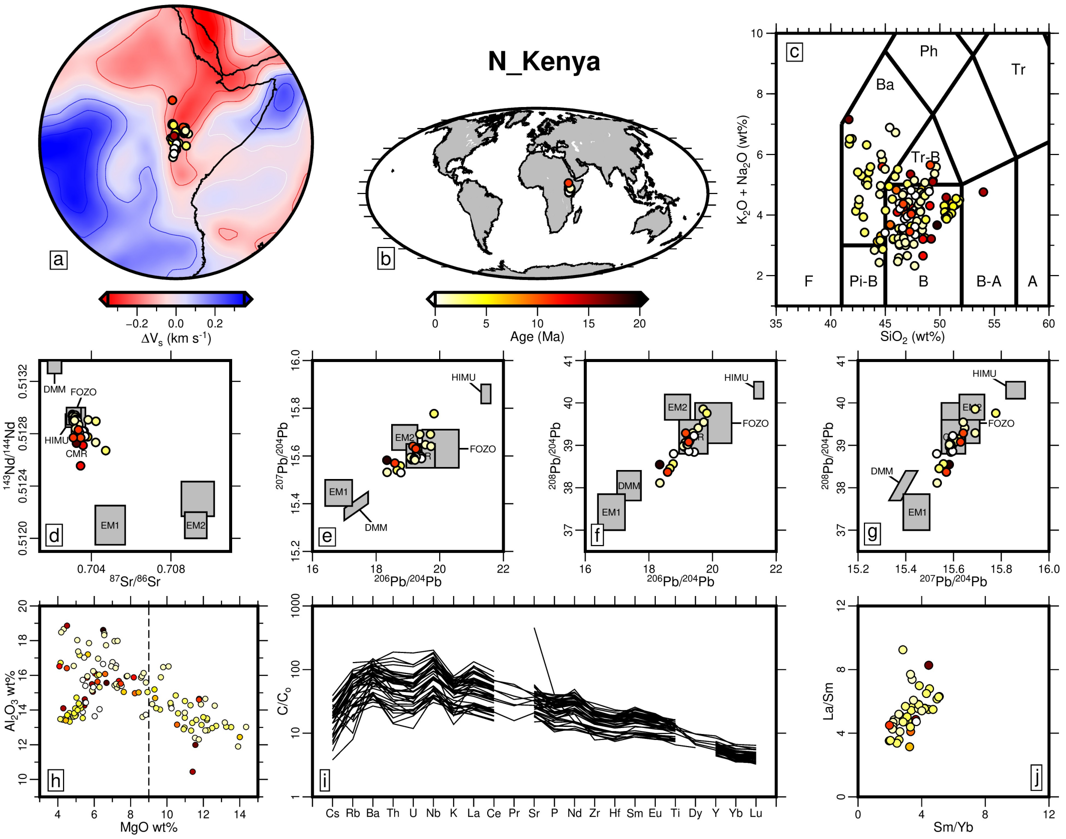
N_Kenya
a
b
c
ΔVs (km s-1)
Age (Ma)
SiO2 (wt%)
K2O + Na2O (wt%)
Ph
Tr
Ba
Tr-B
F
Pi-B
B
B-A
A
d
e
f
g
143Nd/144Nd
87Sr/86Sr
207Pb/204Pb
206Pb/204Pb
208Pb/204Pb
DMM
FOZO
HIMU
CMR
EM1
EM2
h
i
j
Al2O3 wt%
MgO wt%
C/Co
Cs Rb Ba Th U Nb K La Ce Pr Sr P Nd Zr Hf Sm Eu Ti Dy Y Yb Lu
La/Sm
Sm/Yb

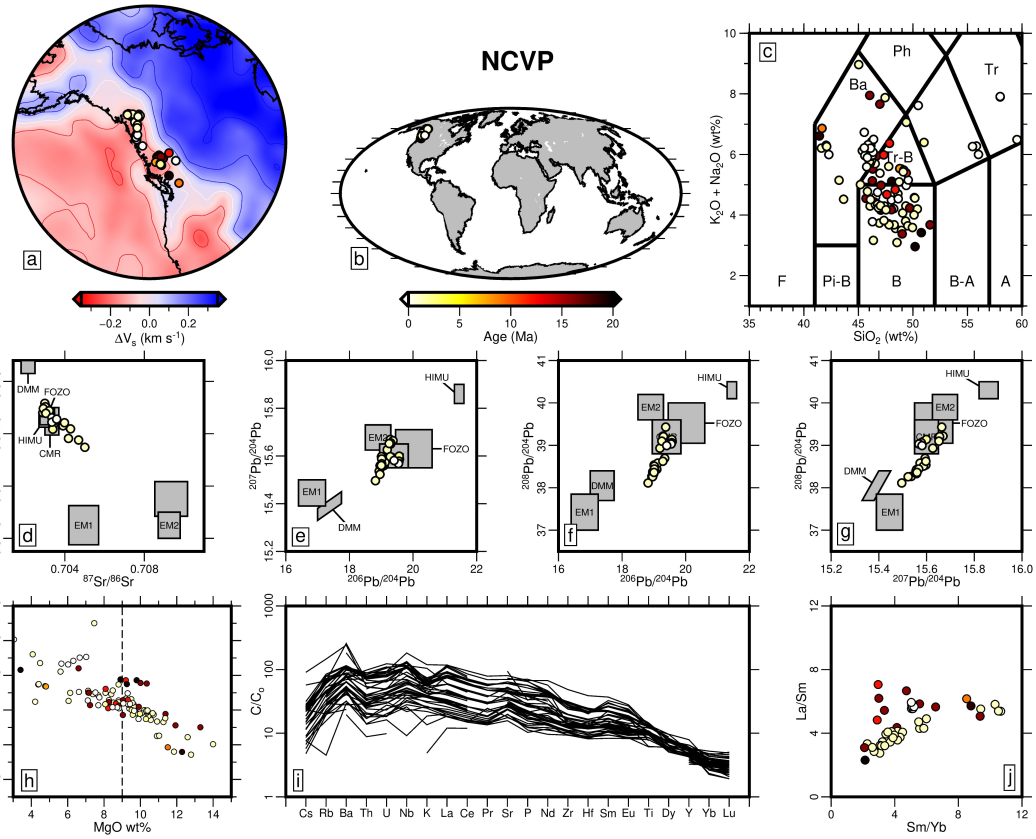
NCVP
a
b
c
d
e
f
g
h
i
j
ΔVs (km s-1)
Age (Ma)
K2O + Na2O (wt%)
SiO2 (wt%)
F
Pi-B
B
B-A
A
Ba
Ph
Tr
Tr-B
143Nd/144Nd
87Sr/86Sr
207Pb/204Pb
206Pb/204Pb
208Pb/204Pb
DMM
FOZO
HIMU
CMR
EM1
EM2
Al2O3 wt%
MgO wt%
C/Co
Cs Rb Ba Th U Nb K La Ce Pr Sr P Nd Zr Hf Sm Eu Ti Dy Y Yb Lu
La/Sm
Sm/Yb

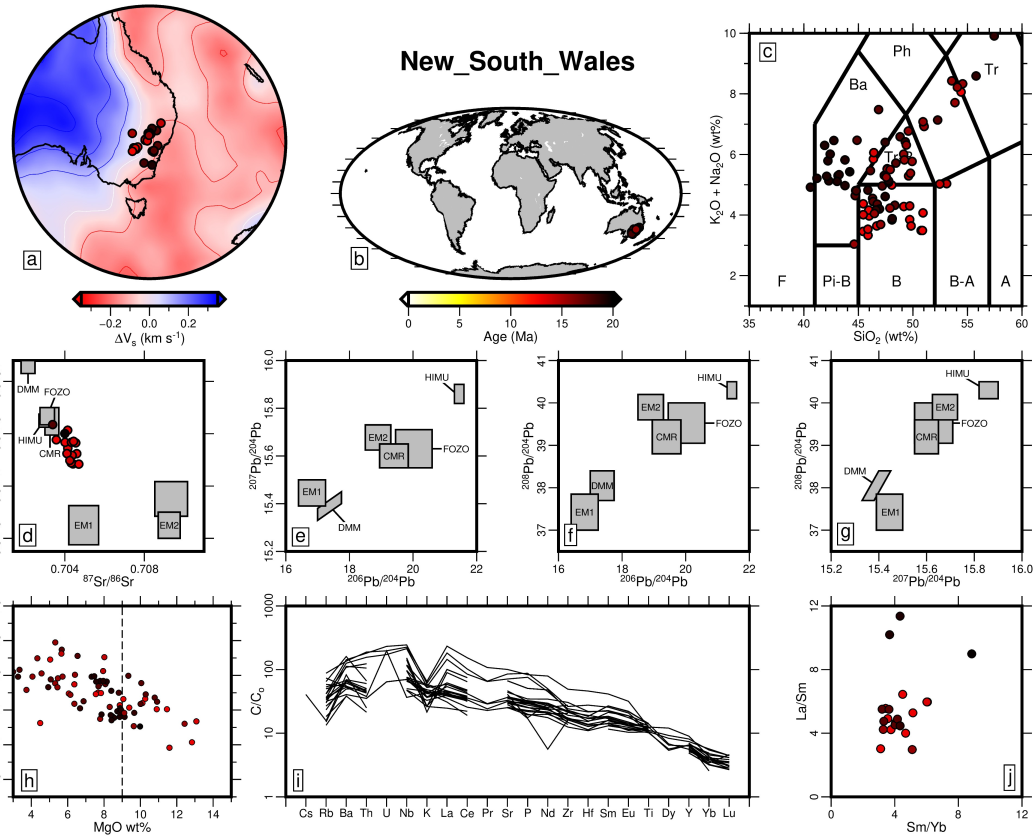
New_South_Wales
a
b
c
d
e
f
g
h
i
j
ΔVs (km s-1)
Age (Ma)
K2O + Na2O (wt%)
SiO2 (wt%)
F
Pi-B
B
B-A
A
Ba
Ph
Tr
Tr-B
143Nd/144Nd
87Sr/86Sr
207Pb/204Pb
206Pb/204Pb
208Pb/204Pb
DMM
FOZO
HIMU
CMR
EM1
EM2
Al2O3 wt%
MgO wt%
C/Co
Cs Rb Ba Th U Nb K La Ce Pr Sr P Nd Zr Hf Sm Eu Ti Dy Y Yb Lu
La/Sm
Sm/Yb

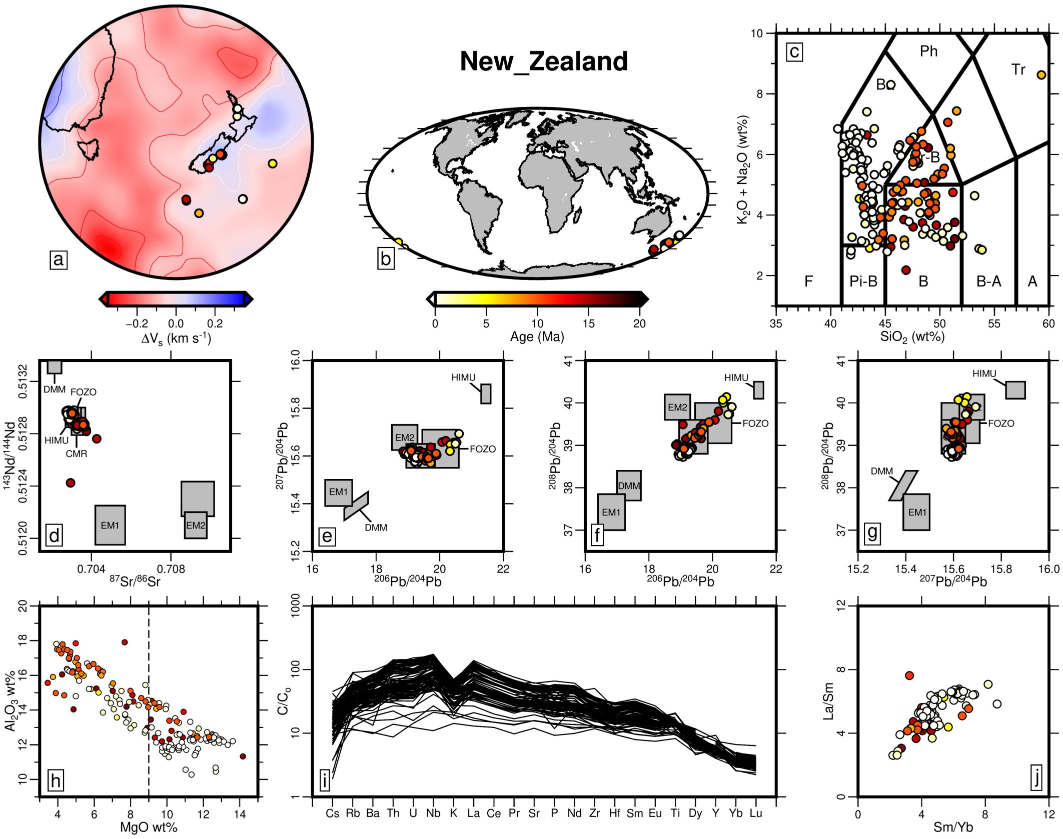
New_Zealand
a
ΔVs (km s-1)
–0.2
0.0
0.2
b
Age (Ma)
0
5
10
15
20
c
Ph
Tr
Bo
F
Pi-B
B
B-A
A
K2O + Na2O (wt%)
SiO2 (wt%)
d
DMM
FOZO
HIMU
CMR
EM1
EM2
143Nd/144Nd
87Sr/86Sr
e
207Pb/204Pb
206Pb/204Pb
f
208Pb/204Pb
206Pb/204Pb
g
208Pb/204Pb
207Pb/204Pb
h
Al2O3 wt%
MgO wt%
i
C/C0
Cs Rb Ba Th U Nb K La Ce Pr Sr P Nd Zr Hf Sm Eu Ti Dy Y Yb Lu
j
La/Sm
Sm/Yb

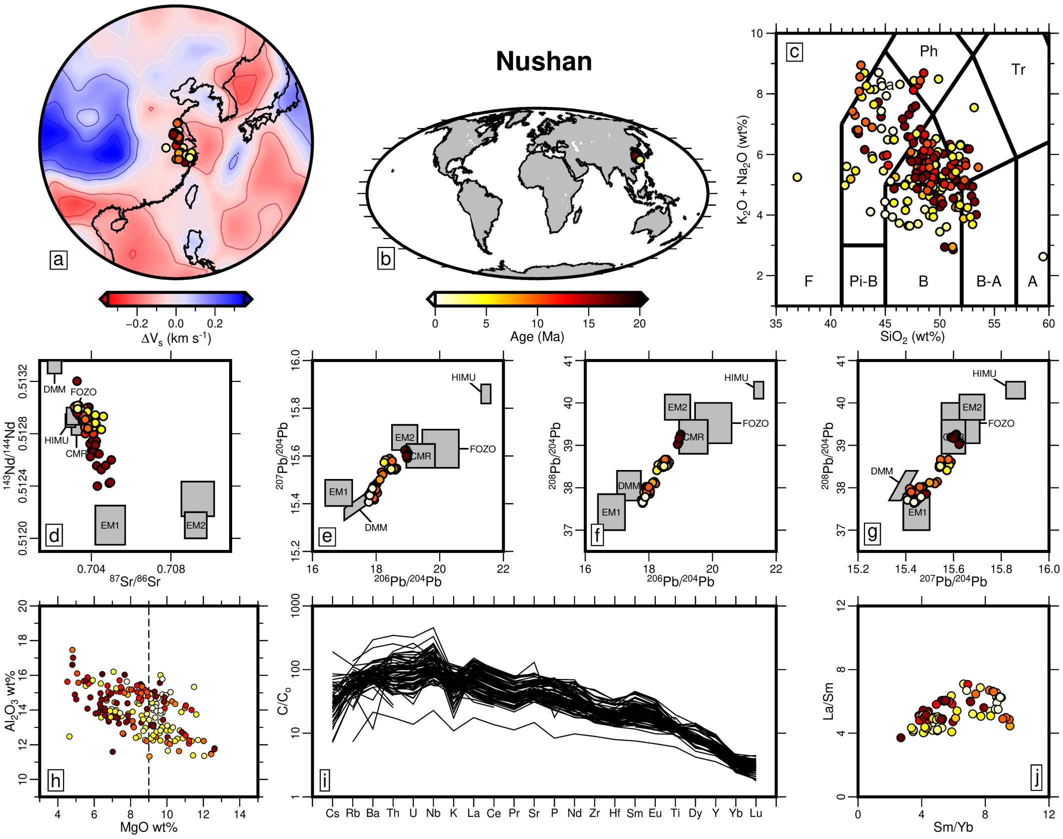
Nushan
a
ΔVs (km s-1)
–0.2
0.0
0.2
b
Age (Ma)
0
5
10
15
20
c
Ph
Tr
F
Pi-B
B
B-A
A
K2O + Na2O (wt%)
SiO2 (wt%)
d
DMM
FOZO
HIMU
CMR
EM1
EM2
143Nd/144Nd
87Sr/86Sr
e
207Pb/204Pb
206Pb/204Pb
f
208Pb/204Pb
206Pb/204Pb
g
208Pb/204Pb
207Pb/204Pb
h
Al2O3 wt%
MgO wt%
i
C/C0
Cs Rb Ba Th U Nb K La Ce Pr Sr P Nd Zr Hf Sm Eu Ti Dy Y Yb Lu
j
La/Sm
Sm/Yb

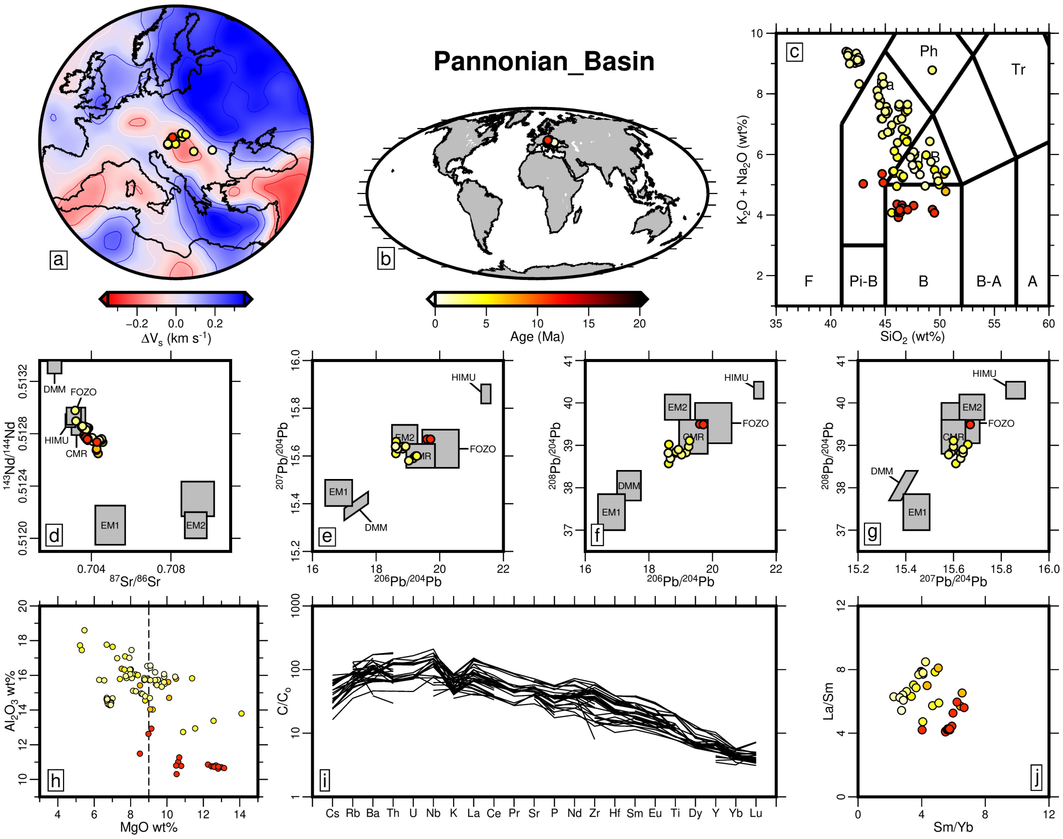
Pannonian_Basin
ΔVs (km s-1)
Age (Ma)
K2O + Na2O (wt%)
SiO2 (wt%)
Ph
Tr
F
Pi-B
B
B-A
A
143Nd/144Nd
87Sr/86Sr
DMM
FOZO
HIMU
CMR
EM1
EM2
207Pb/204Pb
206Pb/204Pb
208Pb/204Pb
Al2O3 wt%
MgO wt%
C/C0
Cs Rb Ba Th U Nb K La Ce Pr Sr P Nd Zr Hf Sm Eu Ti Dy Y Yb Lu
La/Sm
Sm/Yb

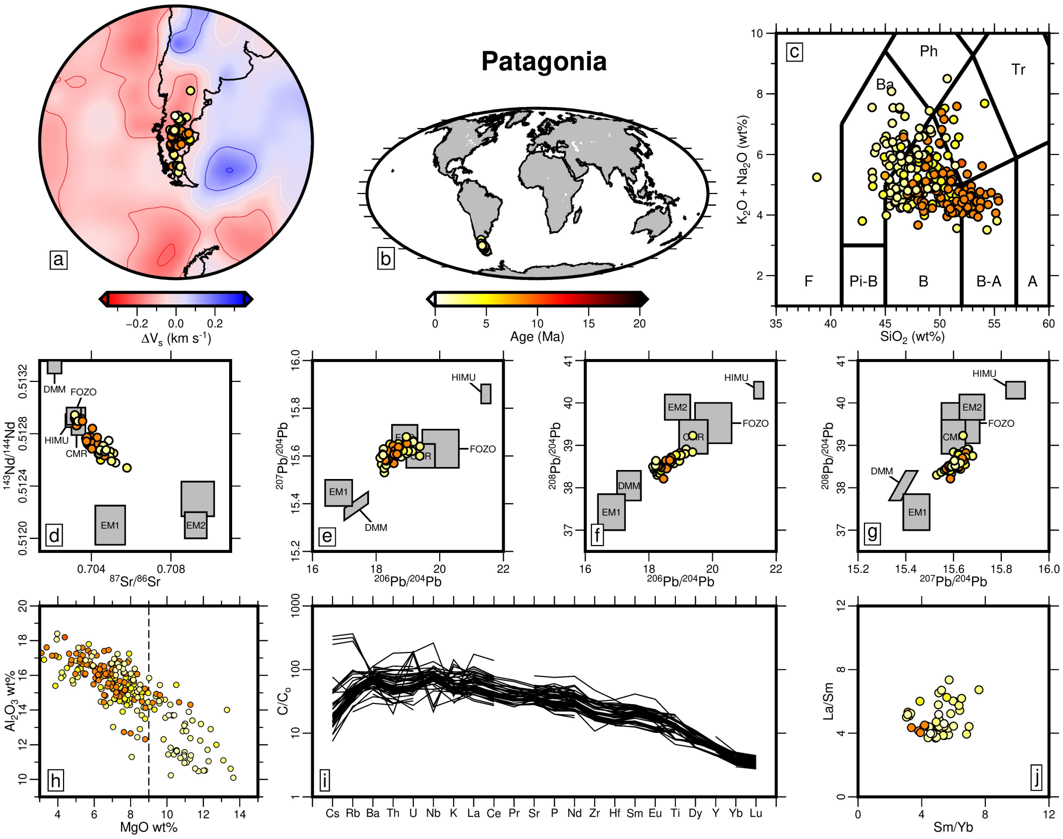
Patagonia
ΔVs (km s-1)
Age (Ma)
K2O + Na2O (wt%)
SiO2 (wt%)
Ph
Ba
Tr
F
Pi-B
B
B-A
A
143Nd/144Nd
87Sr/86Sr
DMM
FOZO
HIMU
CMR
EM1
EM2
207Pb/204Pb
206Pb/204Pb
208Pb/204Pb
Al2O3 wt%
MgO wt%
C/C0
Cs Rb Ba Th U Nb K La Ce Pr Sr P Nd Zr Hf Sm Eu Ti Dy Y Yb Lu
La/Sm
Sm/Yb

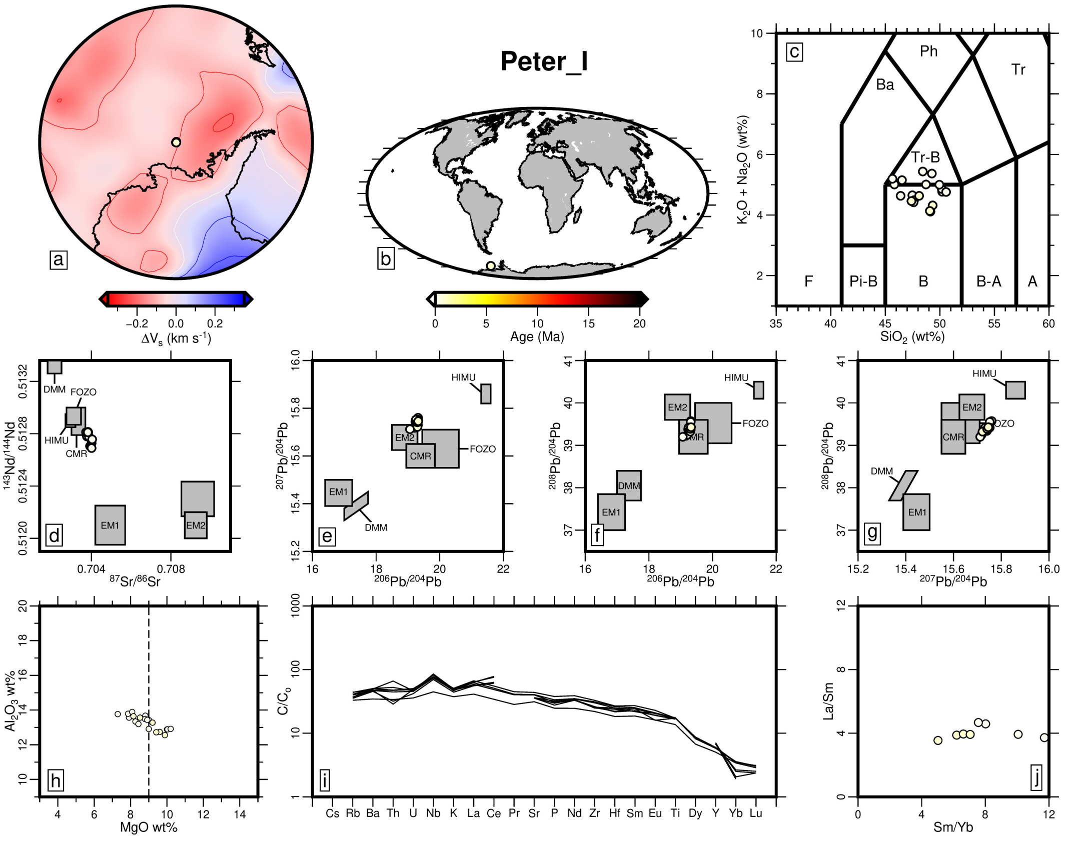
Peter_I
ΔVs (km s-1)
Age (Ma)
K2O + Na2O (wt%)
SiO2 (wt%)
Ph
Tr
Ba
Tr-B
F
Pi-B
B
B-A
A
143Nd/144Nd
87Sr/86Sr
207Pb/204Pb
206Pb/204Pb
208Pb/204Pb
207Pb/204Pb
DMM
FOZO
HIMU
CMR
EM1
EM2
Al2O3 wt%
MgO wt%
C/Co
Cs Rb Ba Th U Nb K La Ce Pr Sr P Nd Zr Hf Sm Eu Ti Dy Y Yb Lu
La/Sm
Sm/Yb

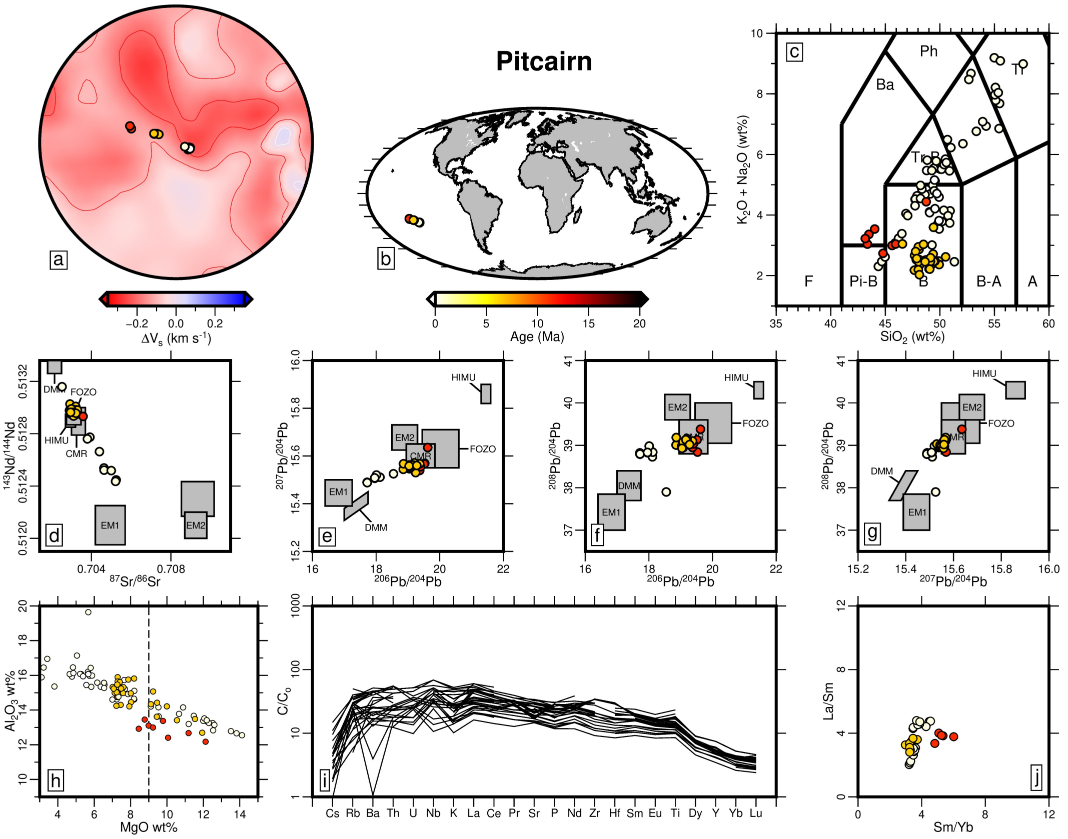
Pitcairn
ΔVs (km s-1)
Age (Ma)
K2O + Na2O (wt%)
SiO2 (wt%)
Ph
Tr
Ba
Tr-B
F
Pi-B
B
B-A
A
143Nd/144Nd
87Sr/86Sr
207Pb/204Pb
206Pb/204Pb
208Pb/204Pb
207Pb/204Pb
DMM
FOZO
HIMU
CMR
EM1
EM2
Al2O3 wt%
MgO wt%
C/Co
Cs Rb Ba Th U Nb K La Ce Pr Sr P Nd Zr Hf Sm Eu Ti Dy Y Yb Lu
La/Sm
Sm/Yb

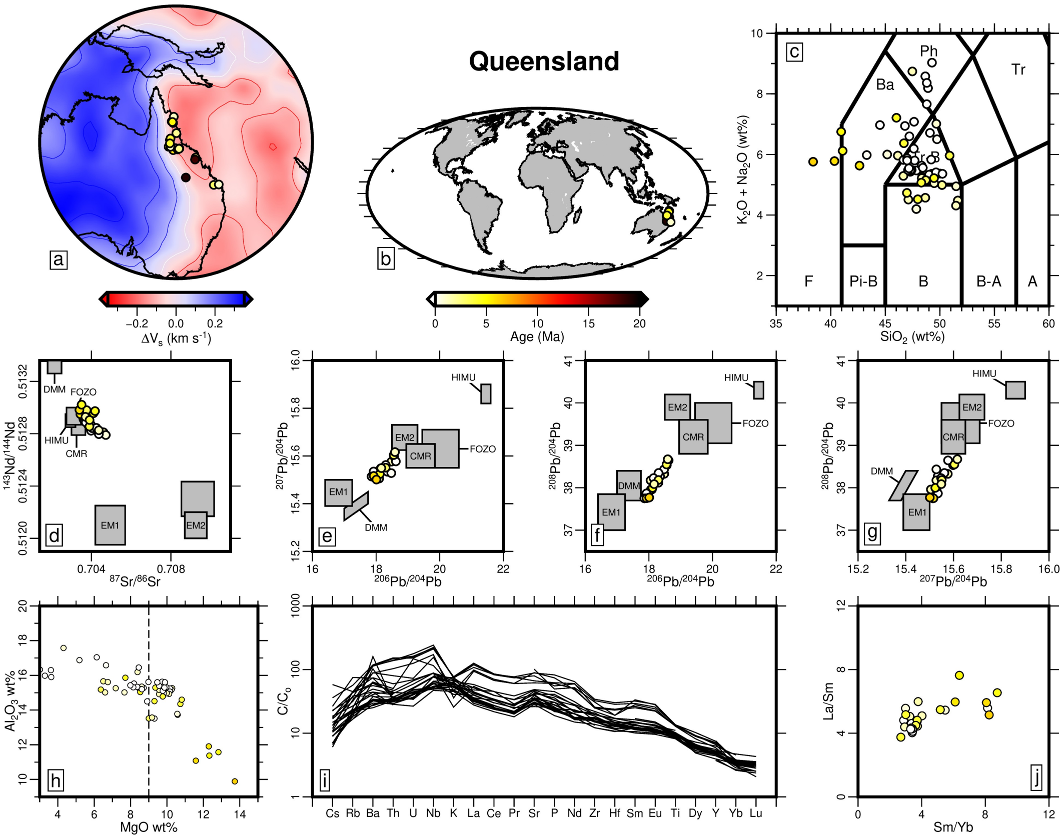
Queensland
a
b
c
ΔVs (km s-1)
Age (Ma)
K2O + Na2O (wt%)
SiO2 (wt%)
Ph
Tr
Ba
F
Pi-B
B
B-A
A
d
e
f
g
143Nd/144Nd
87Sr/86Sr
207Pb/204Pb
206Pb/204Pb
208Pb/204Pb
207Pb/204Pb
DMM
FOZO
HIMU
CMR
EM1
EM2
h
i
j
Al2O3 wt%
MgO wt%
C/Co
Cs Rb Ba Th U Nb K La Ce Pr Sr P Nd Zr Hf Sm Eu Ti Dy Y Yb Lu
La/Sm
Sm/Yb

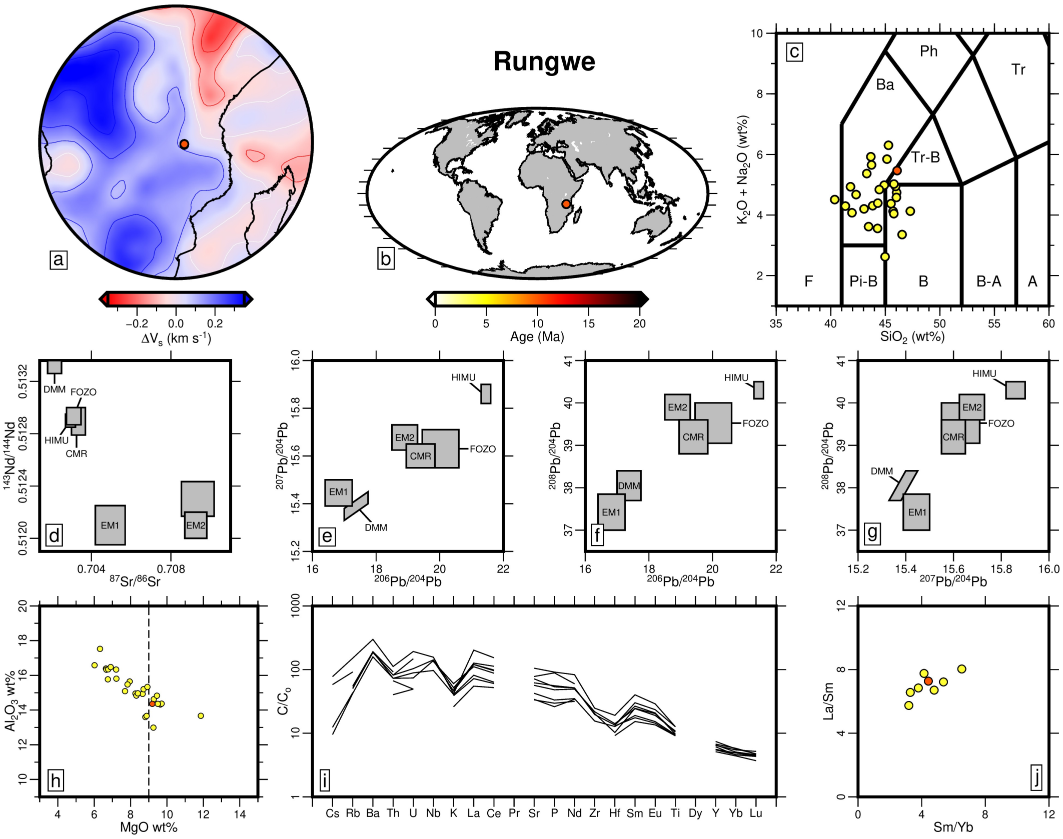
Rungwe
a
b
c
ΔVs (km s-1)
Age (Ma)
K2O + Na2O (wt%)
SiO2 (wt%)
Ph
Tr
Ba
Tr-B
F
Pi-B
B
B-A
A
d
e
f
g
143Nd/144Nd
87Sr/86Sr
207Pb/204Pb
206Pb/204Pb
208Pb/204Pb
207Pb/204Pb
DMM
FOZO
HIMU
CMR
EM1
EM2
h
i
j
Al2O3 wt%
MgO wt%
C/Co
Cs Rb Ba Th U Nb K La Ce Pr Sr P Nd Zr Hf Sm Eu Ti Dy Y Yb Lu
La/Sm
Sm/Yb

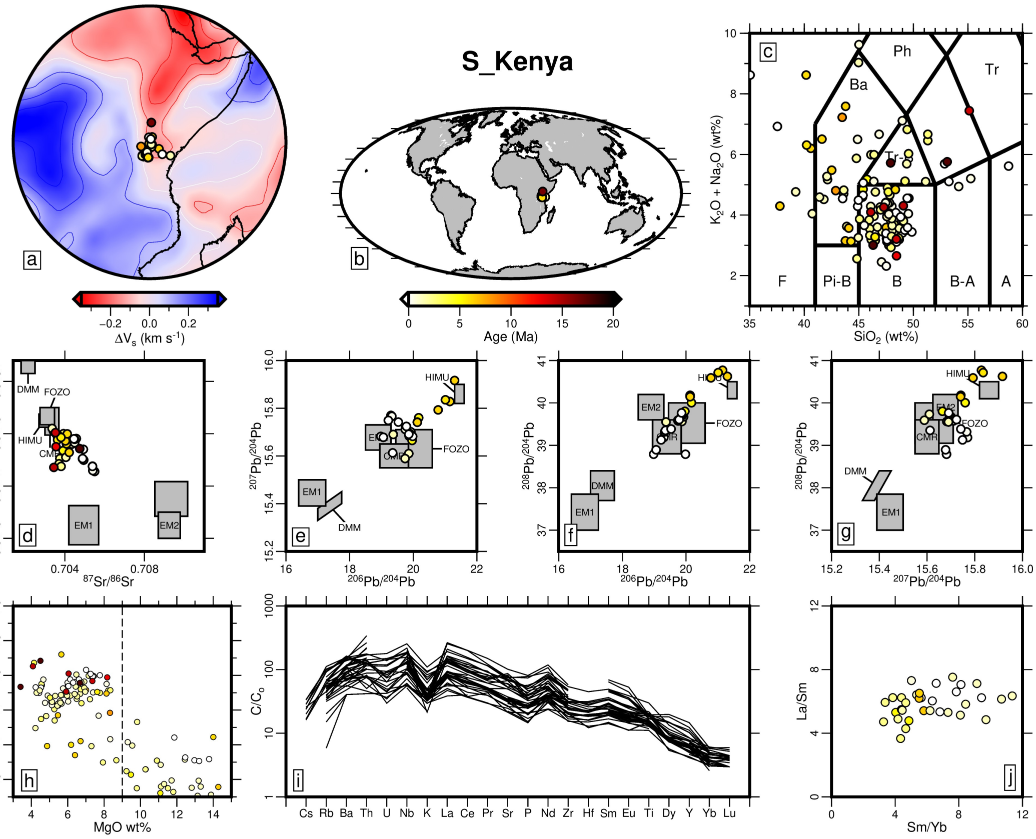
S_Kenya
a
ΔV_s (km s^-1)
b
Age (Ma)
c
Ph
Tr
Ba
Tr-B
F
Pi-B
B
B-A
A
K_2O + Na_2O (wt%)
SiO_2 (wt%)
d
DMM
FOZO
HIMU
CMR
EM1
EM2
143Nd/144Nd
87Sr/86Sr
e
207Pb/204Pb
206Pb/204Pb
f
208Pb/204Pb
g
h
Al_2O_3 wt%
MgO wt%
i
C/C_0
Cs Rb Ba Th U Nb K La Ce Pr Sr P Nd Zr Hf Sm Eu Ti Dy Y Yb Lu
j
La/Sm
Sm/Yb

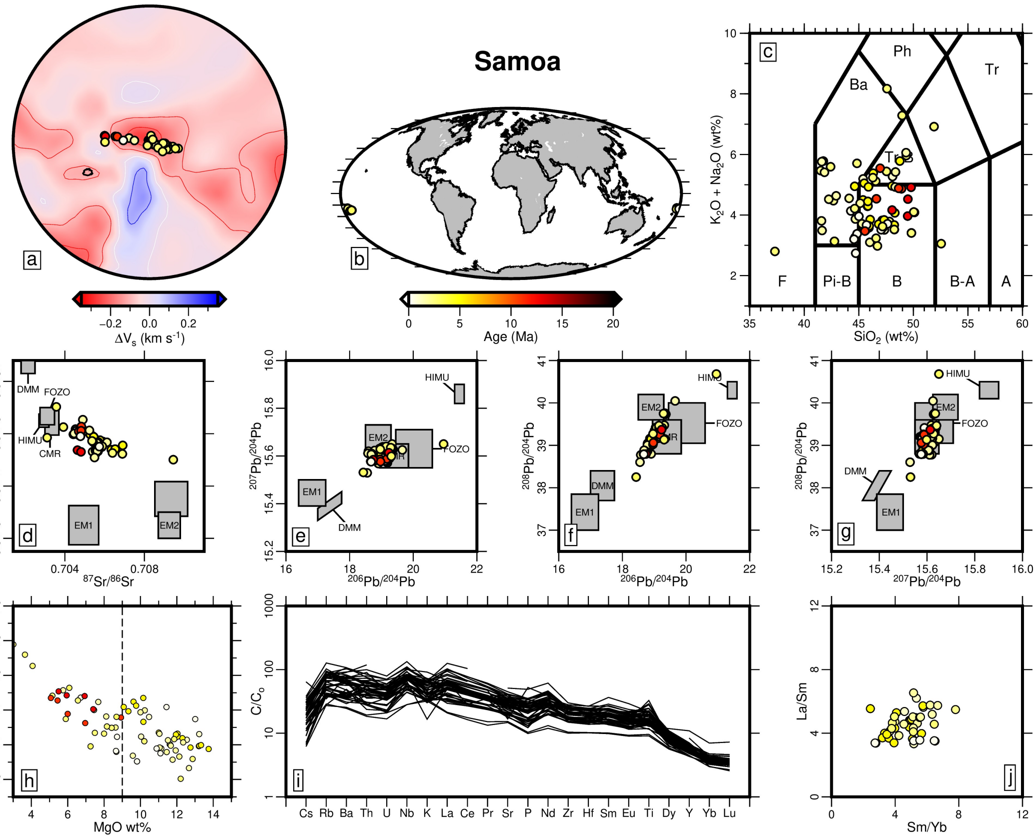
Samoa
a
ΔV_s (km s^-1)
b
Age (Ma)
c
Ph
Tr
Ba
Tr-B
F
Pi-B
B
B-A
A
K_2O + Na_2O (wt%)
SiO_2 (wt%)
d
DMM
FOZO
HIMU
CMR
EM1
EM2
143Nd/144Nd
87Sr/86Sr
e
207Pb/204Pb
206Pb/204Pb
f
208Pb/204Pb
g
h
Al_2O_3 wt%
MgO wt%
i
C/C_0
Cs Rb Ba Th U Nb K La Ce Pr Sr P Nd Zr Hf Sm Eu Ti Dy Y Yb Lu
j
La/Sm
Sm/Yb

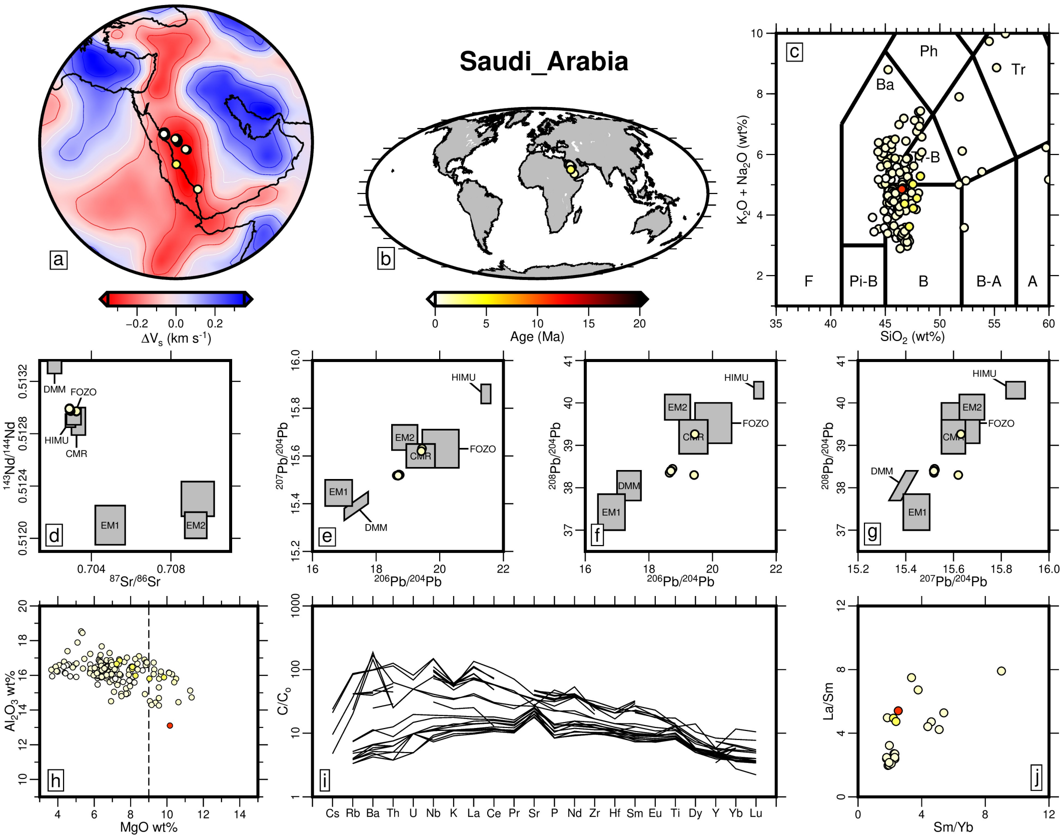
Saudi_Arabia
a
b
c
d
e
f
g
h
i
j
ΔVs (km s-1)
Age (Ma)
K2O + Na2O (wt%)
SiO2 (wt%)
F
Pi-B
B
B-A
A
Ba
Ph
Tr
T-B
143Nd/144Nd
87Sr/86Sr
207Pb/204Pb
206Pb/204Pb
208Pb/204Pb
DMM
FOZO
HIMU
CMR
EM1
EM2
Al2O3 wt%
MgO wt%
C/Co
Cs Rb Ba Th U Nb K La Ce Pr Sr P Nd Zr Hf Sm Eu Ti Dy Y Yb Lu
La/Sm
Sm/Yb

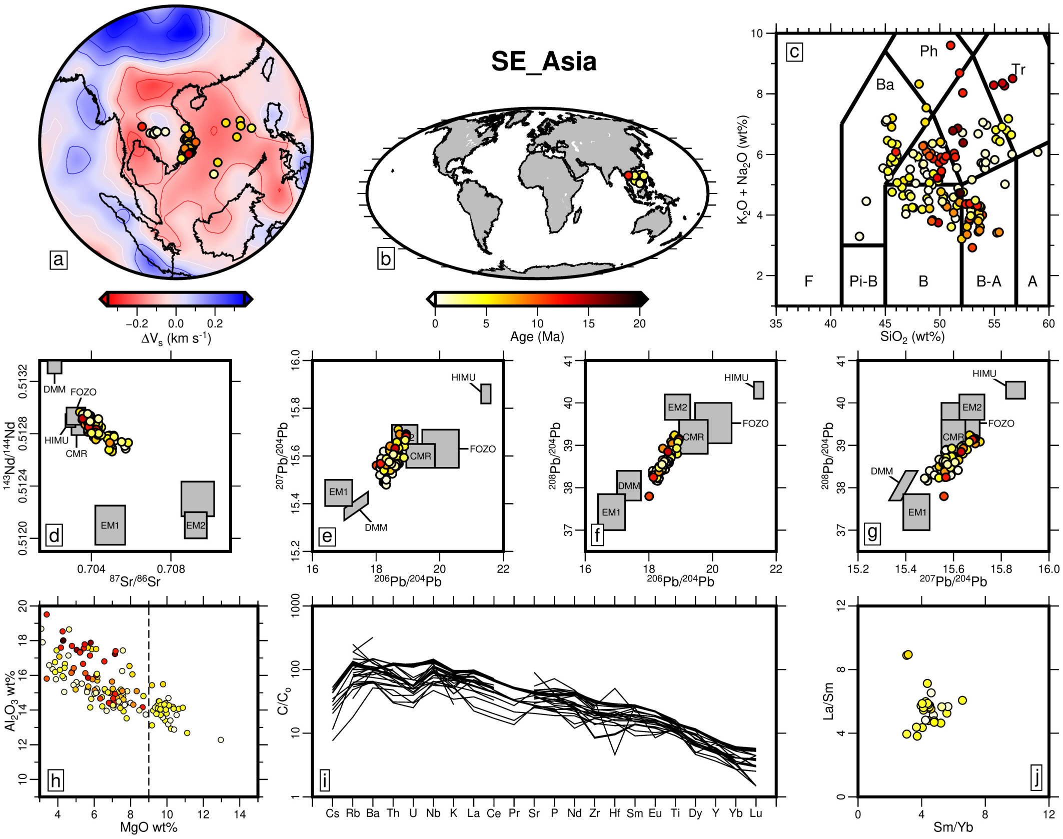
SE_Asia
a
b
c
d
e
f
g
h
i
j
ΔVs (km s-1)
Age (Ma)
K2O + Na2O (wt%)
SiO2 (wt%)
F
Pi-B
B
B-A
A
Ba
Ph
Tr
143Nd/144Nd
87Sr/86Sr
207Pb/204Pb
206Pb/204Pb
208Pb/204Pb
DMM
FOZO
HIMU
CMR
EM1
EM2
Al2O3 wt%
MgO wt%
C/Co
Cs Rb Ba Th U Nb K La Ce Pr Sr P Nd Zr Hf Sm Eu Ti Dy Y Yb Lu
La/Sm
Sm/Yb

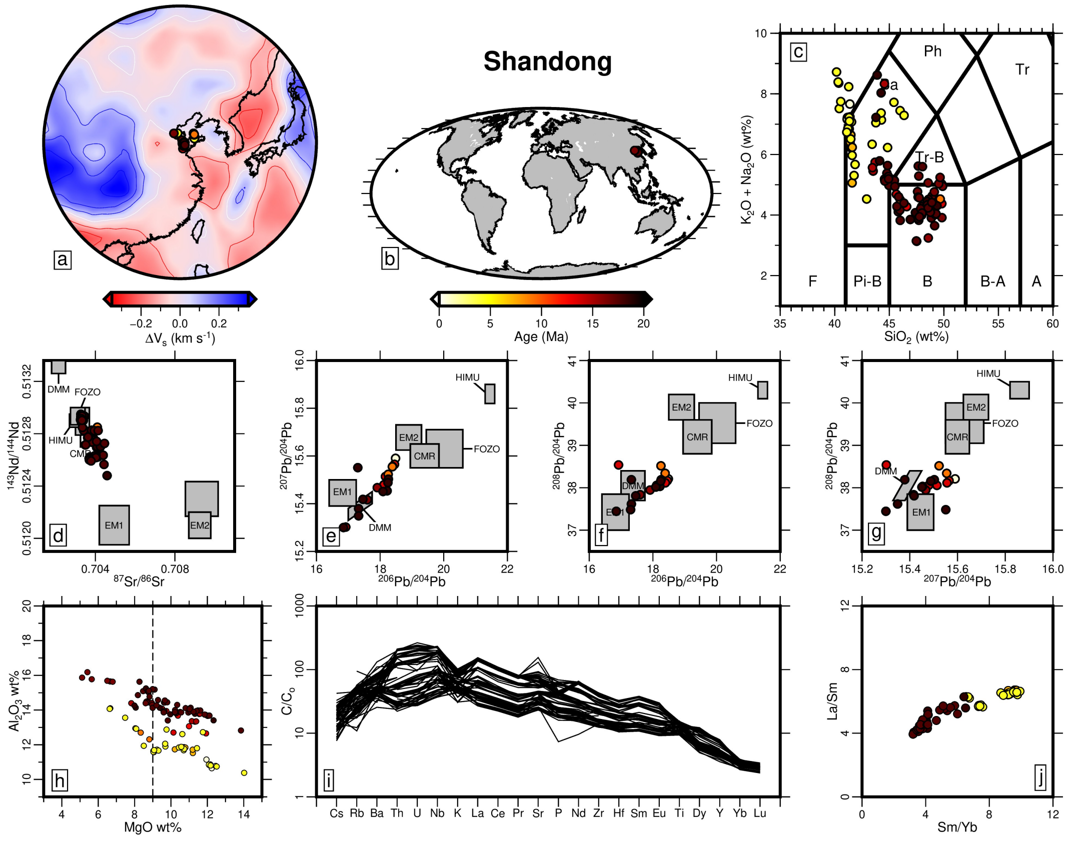
Shandong
a
b
c
Ph
Tr
Tr-B
F
Pi-B
B
B-A
A
K$_2$O + Na$_2$O (wt%)
SiO$_2$ (wt%)
ΔV$_s$ (km s^{-1})
Age (Ma)
d
e
f
g
DMM
FOZO
HIMU
CMR
EM1
EM2
^{143}Nd/^{144}Nd
^{87}Sr/^{86}Sr
^{207}Pb/^{204}Pb
^{206}Pb/^{204}Pb
^{208}Pb/^{204}Pb
h
i
j
Al$_2$O$_3$ wt%
MgO wt%
C/C$_0$
Cs Rb Ba Th U Nb K La Ce Pr Sr P Nd Zr Hf Sm Eu Ti Dy Y Yb Lu
La/Sm
Sm/Yb

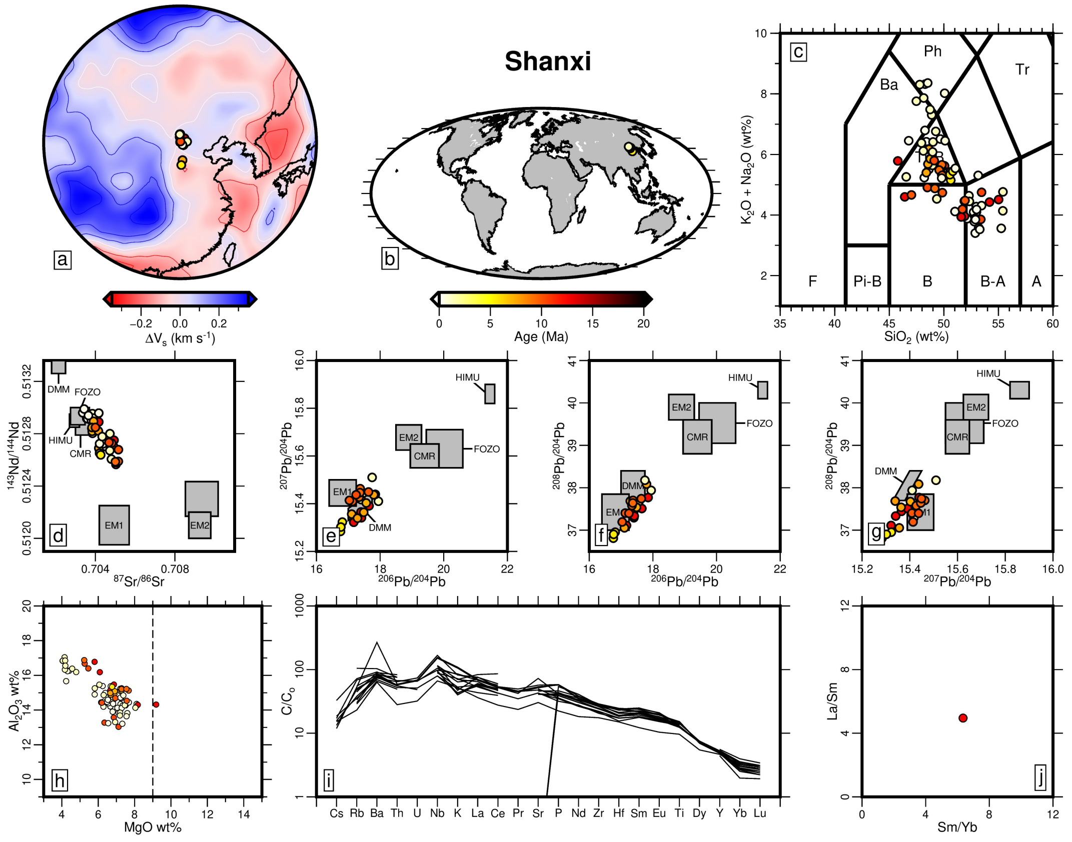
Shanxi
a
b
c
Ph
Ba
Tr
F
Pi-B
B
B-A
A
K$_2$O + Na$_2$O (wt%)
SiO$_2$ (wt%)
ΔV$_s$ (km s^{-1})
Age (Ma)
d
e
f
g
DMM
FOZO
HIMU
CMR
EM1
EM2
^{143}Nd/^{144}Nd
^{87}Sr/^{86}Sr
^{207}Pb/^{204}Pb
^{206}Pb/^{204}Pb
^{208}Pb/^{204}Pb
h
i
j
Al$_2$O$_3$ wt%
MgO wt%
C/C$_0$
Cs Rb Ba Th U Nb K La Ce Pr Sr P Nd Zr Hf Sm Eu Ti Dy Y Yb Lu
La/Sm
Sm/Yb

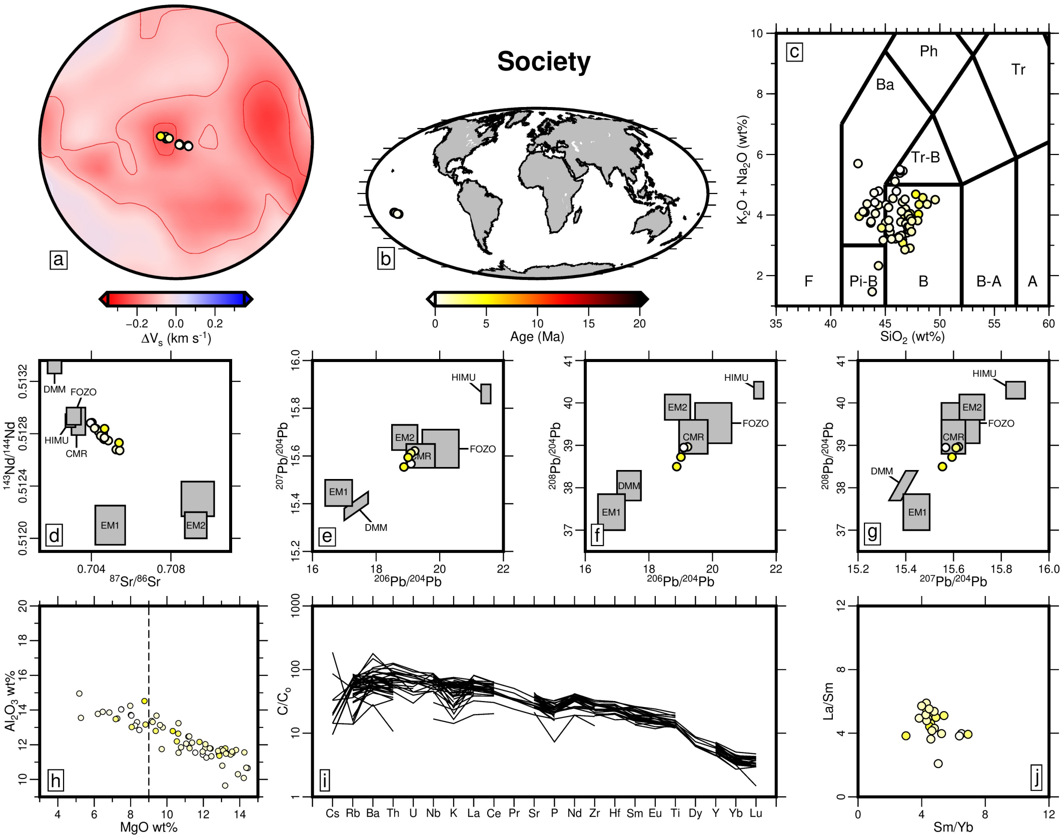
Society
a
b
c
d
e
f
g
h
i
j
ΔVs (km s-1)
Age (Ma)
K2O + Na2O (wt%)
SiO2 (wt%)
F
Pi-B
B
B-A
A
Ba
Ph
Tr
Tr-B
143Nd/144Nd
87Sr/86Sr
207Pb/204Pb
206Pb/204Pb
208Pb/204Pb
DMM
FOZO
HIMU
CMR
EM1
EM2
Al2O3 wt%
MgO wt%
C/Co
Cs Rb Ba Th U Nb K La Ce Pr Sr P Nd Zr Hf Sm Eu Ti Dy Y Yb Lu
La/Sm
Sm/Yb

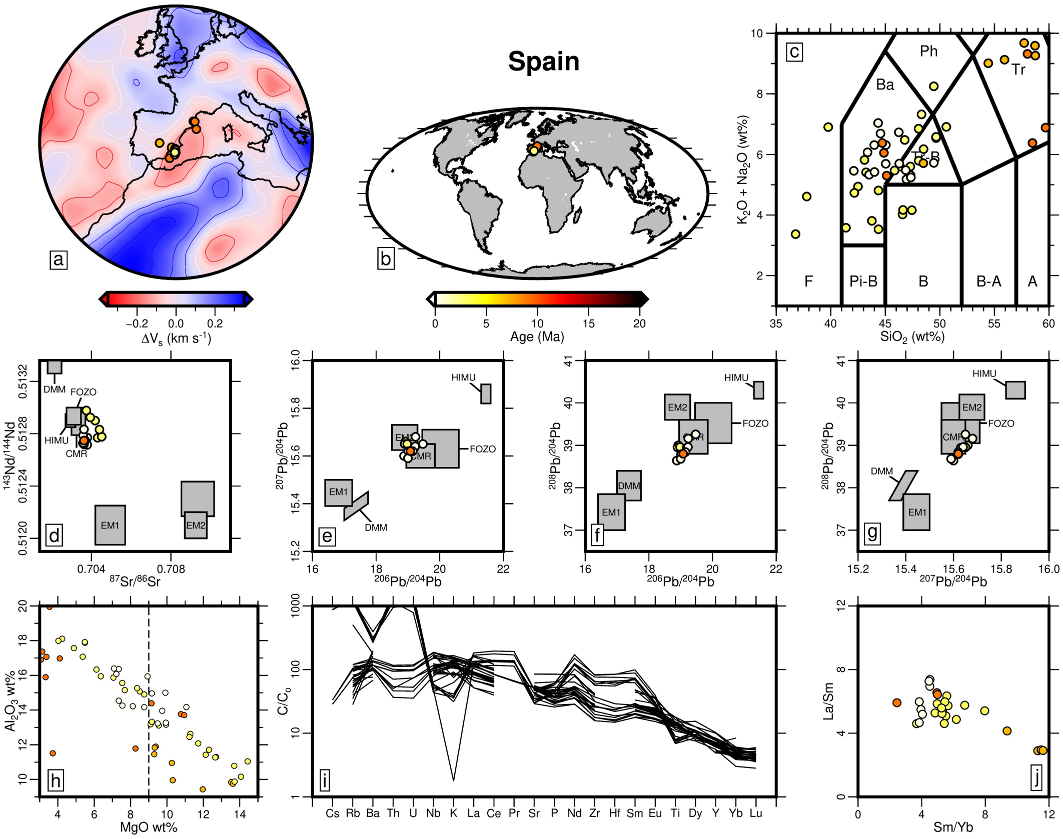
Spain
a
b
c
d
e
f
g
h
i
j
ΔVs (km s-1)
Age (Ma)
K2O + Na2O (wt%)
SiO2 (wt%)
F
Pi-B
B
B-A
A
Ba
Ph
Tr
Tr-B
143Nd/144Nd
87Sr/86Sr
207Pb/204Pb
206Pb/204Pb
208Pb/204Pb
DMM
FOZO
HIMU
CMR
EM1
EM2
Al2O3 wt%
MgO wt%
C/Co
Cs Rb Ba Th U Nb K La Ce Pr Sr P Nd Zr Hf Sm Eu Ti Dy Y Yb Lu
La/Sm
Sm/Yb

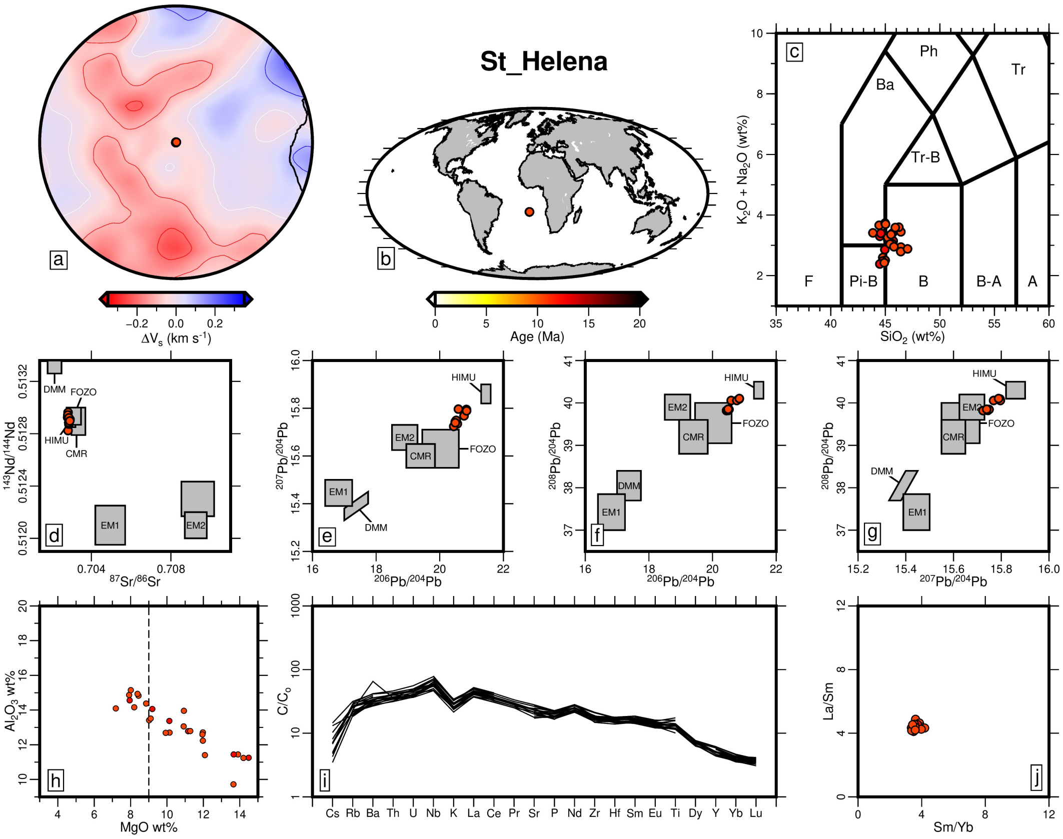
St_Helena
a
b
c
d
e
f
g
h
i
j
ΔVs (km s-1)
Age (Ma)
SiO2 (wt%)
K2O + Na2O (wt%)
Ph
Ba
Tr
Tr-B
F
Pi-B
B
B-A
A
143Nd/144Nd
87Sr/86Sr
207Pb/204Pb
206Pb/204Pb
208Pb/204Pb
DMM
FOZO
HIMU
CMR
EM1
EM2
Al2O3 wt%
MgO wt%
C/Co
Cs Rb Ba Th U Nb K La Ce Pr Sr P Nd Zr Hf Sm Eu Ti Dy Y Yb Lu
La/Sm
Sm/Yb

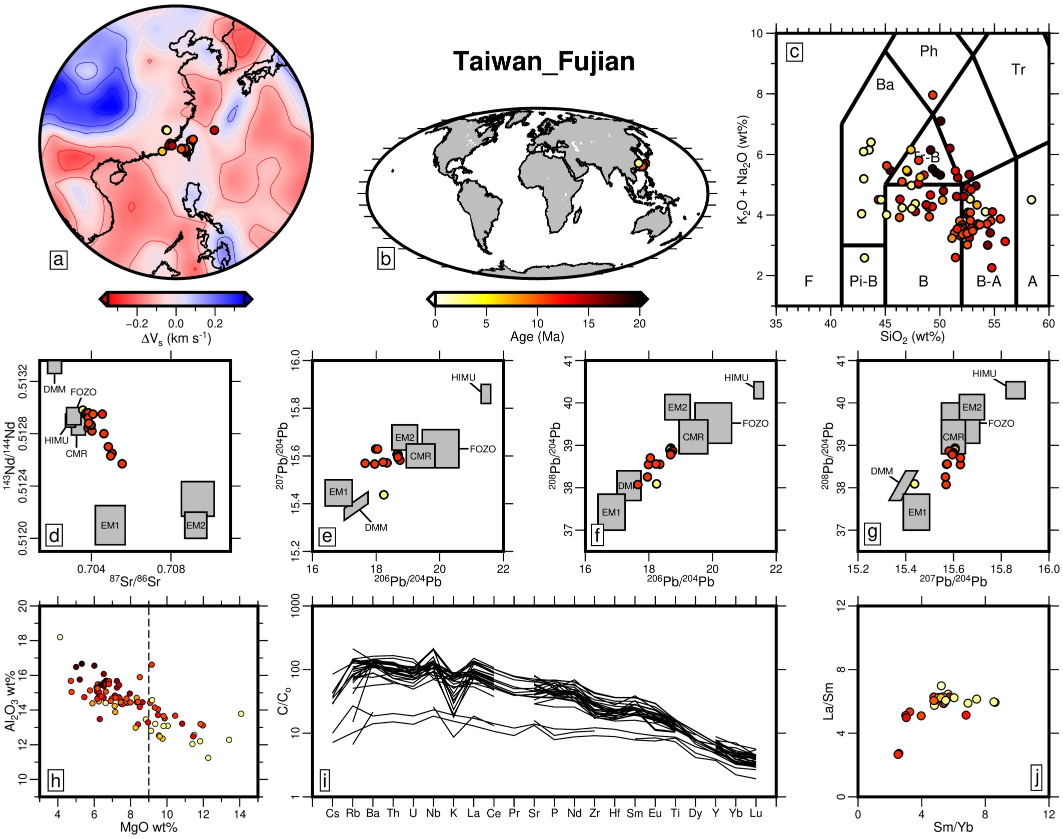
Taiwan_Fujian
a
b
c
d
e
f
g
h
i
j
ΔVs (km s-1)
Age (Ma)
SiO2 (wt%)
K2O + Na2O (wt%)
Ph
Ba
Tr
Tr-B
F
Pi-B
B
B-A
A
143Nd/144Nd
87Sr/86Sr
207Pb/204Pb
206Pb/204Pb
208Pb/204Pb
DMM
FOZO
HIMU
CMR
EM1
EM2
Al2O3 wt%
MgO wt%
C/Co
Cs Rb Ba Th U Nb K La Ce Pr Sr P Nd Zr Hf Sm Eu Ti Dy Y Yb Lu
La/Sm
Sm/Yb

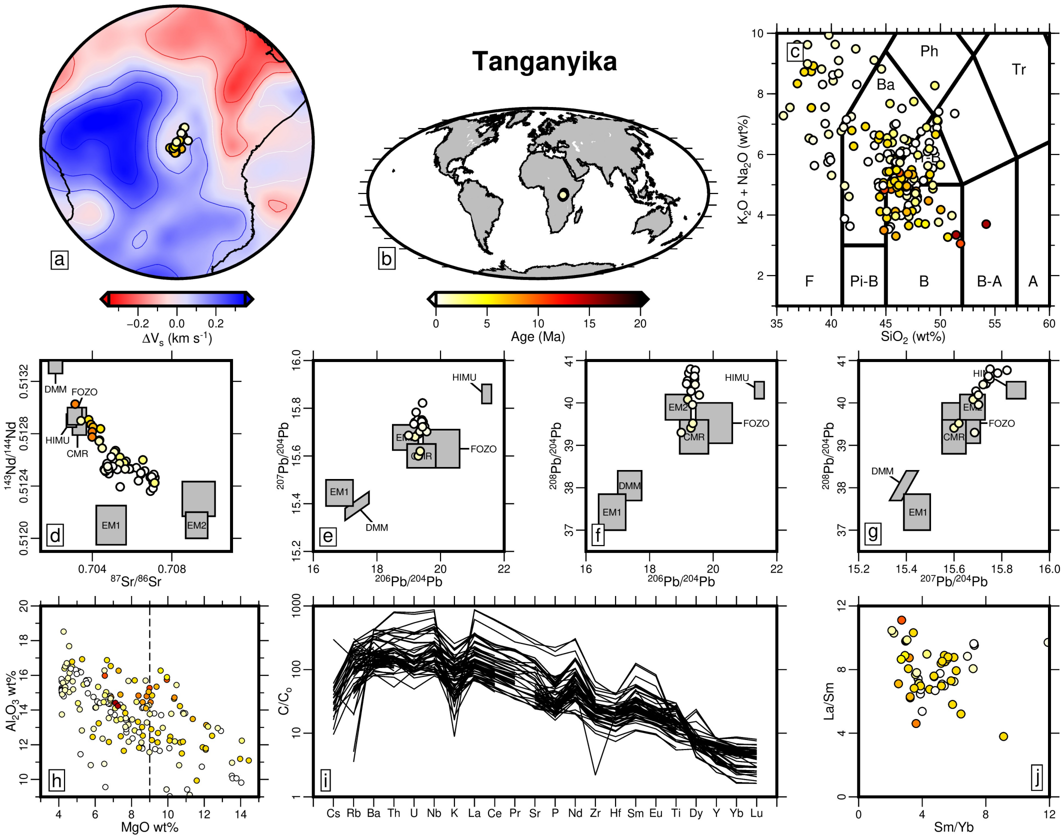
Tanganyika
a
b
c
d
e
f
g
h
i
j
ΔVs (km s-1)
Age (Ma)
SiO2 (wt%)
K2O + Na2O (wt%)
Ph
Tr
Ba
F
Pi-B
B
B-A
A
87Sr/86Sr
143Nd/144Nd
206Pb/204Pb
207Pb/204Pb
208Pb/204Pb
DMM
FOZO
HIMU
CMR
EM1
EM2
MgO wt%
Al2O3 wt%
C/Co
Cs Rb Ba Th U Nb K La Ce Pr Sr P Nd Zr Hf Sm Eu Ti Dy Y Yb Lu
Sm/Yb
La/Sm

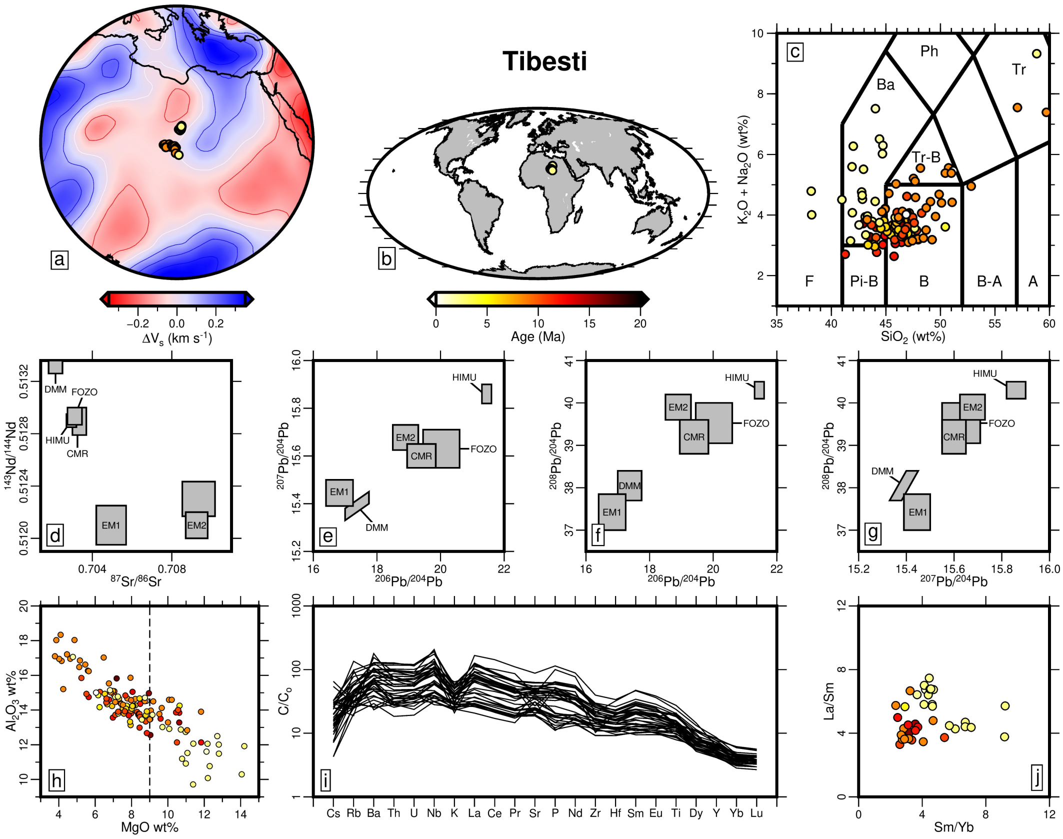
Tibesti
a
b
c
d
e
f
g
h
i
j
ΔVs (km s-1)
Age (Ma)
SiO2 (wt%)
K2O + Na2O (wt%)
Ph
Tr
Ba
Tr-B
F
Pi-B
B
B-A
A
87Sr/86Sr
143Nd/144Nd
206Pb/204Pb
207Pb/204Pb
208Pb/204Pb
DMM
FOZO
HIMU
CMR
EM1
EM2
MgO wt%
Al2O3 wt%
C/Co
Cs Rb Ba Th U Nb K La Ce Pr Sr P Nd Zr Hf Sm Eu Ti Dy Y Yb Lu
Sm/Yb
La/Sm

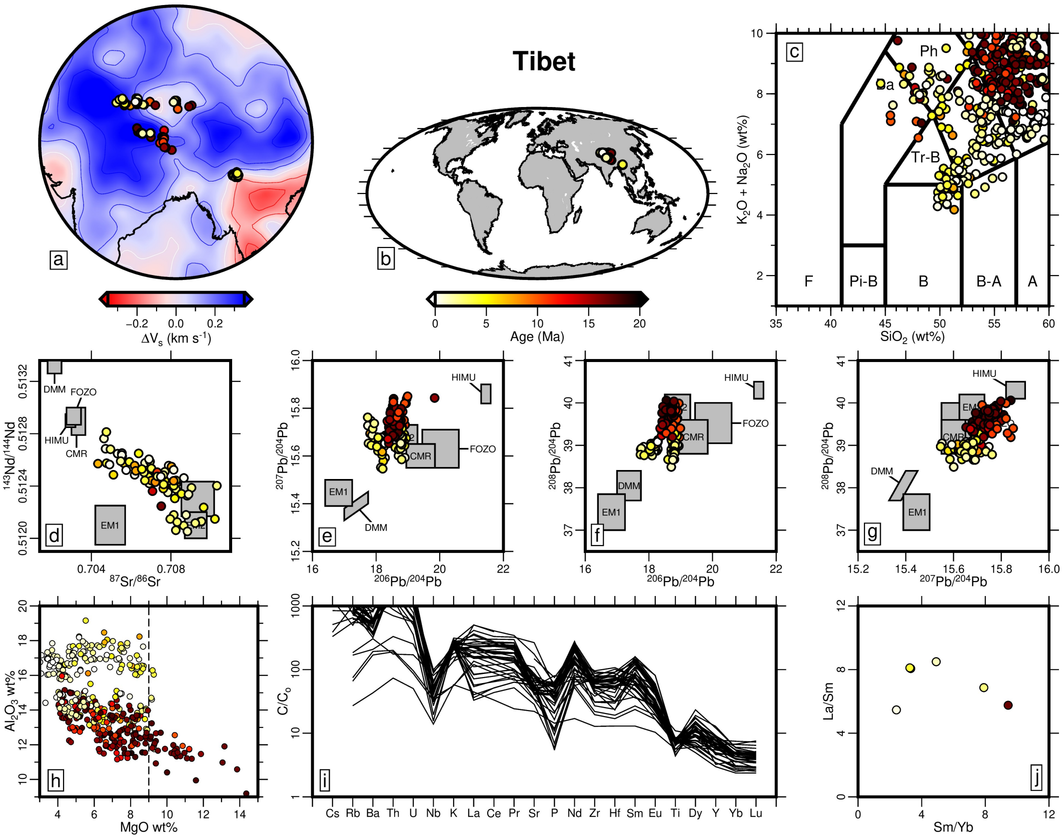
Tibet
a
b
c
d
e
f
g
h
i
j
ΔVs (km s-1)
Age (Ma)
SiO2 (wt%)
K2O + Na2O (wt%)
F
Pi-B
B
B-A
A
Ph
Tr-B
DMM
FOZO
HIMU
CMR
EM1
87Sr/86Sr
143Nd/144Nd
206Pb/204Pb
207Pb/204Pb
208Pb/204Pb
MgO wt%
Al2O3 wt%
C/Co
Cs Rb Ba Th U Nb K La Ce Pr Sr P Nd Zr Hf Sm Eu Ti Dy Y Yb Lu
Sm/Yb
La/Sm

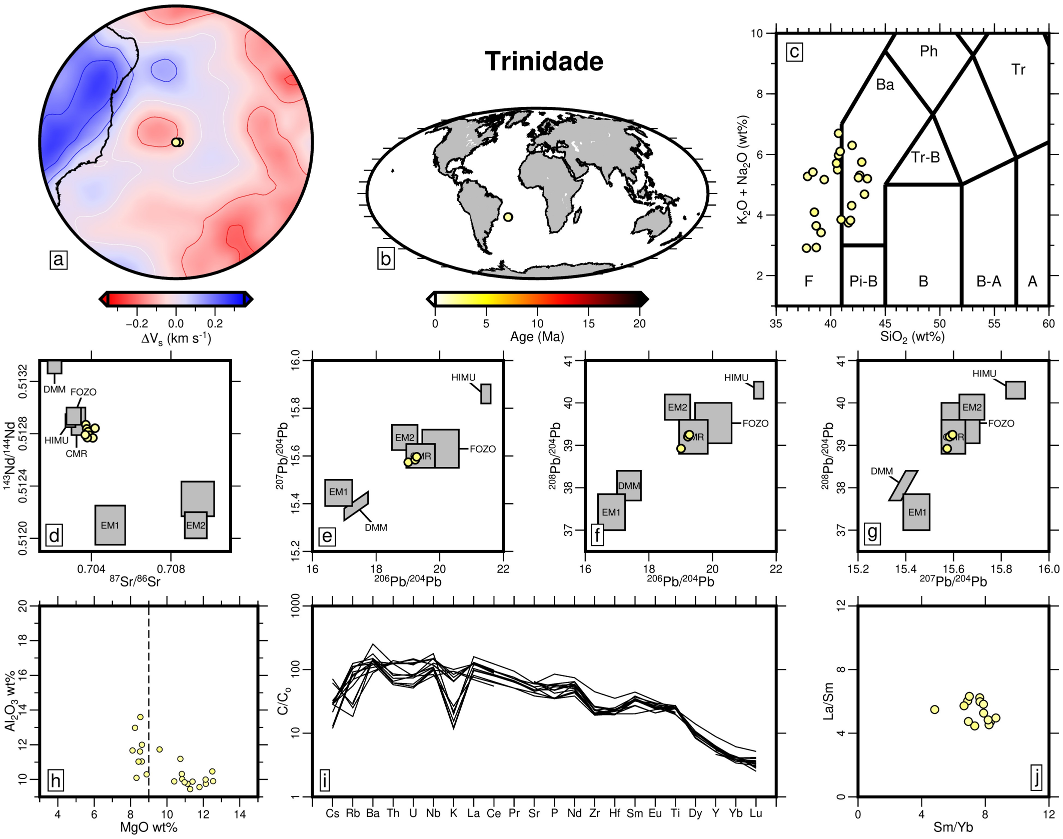
Trinidade
a
b
c
d
e
f
g
h
i
j
ΔVs (km s-1)
Age (Ma)
SiO2 (wt%)
K2O + Na2O (wt%)
F
Pi-B
B
B-A
A
Ba
Ph
Tr
Tr-B
DMM
FOZO
HIMU
CMR
EM1
EM2
87Sr/86Sr
143Nd/144Nd
206Pb/204Pb
207Pb/204Pb
208Pb/204Pb
MgO wt%
Al2O3 wt%
C/Co
Cs Rb Ba Th U Nb K La Ce Pr Sr P Nd Zr Hf Sm Eu Ti Dy Y Yb Lu
Sm/Yb
La/Sm

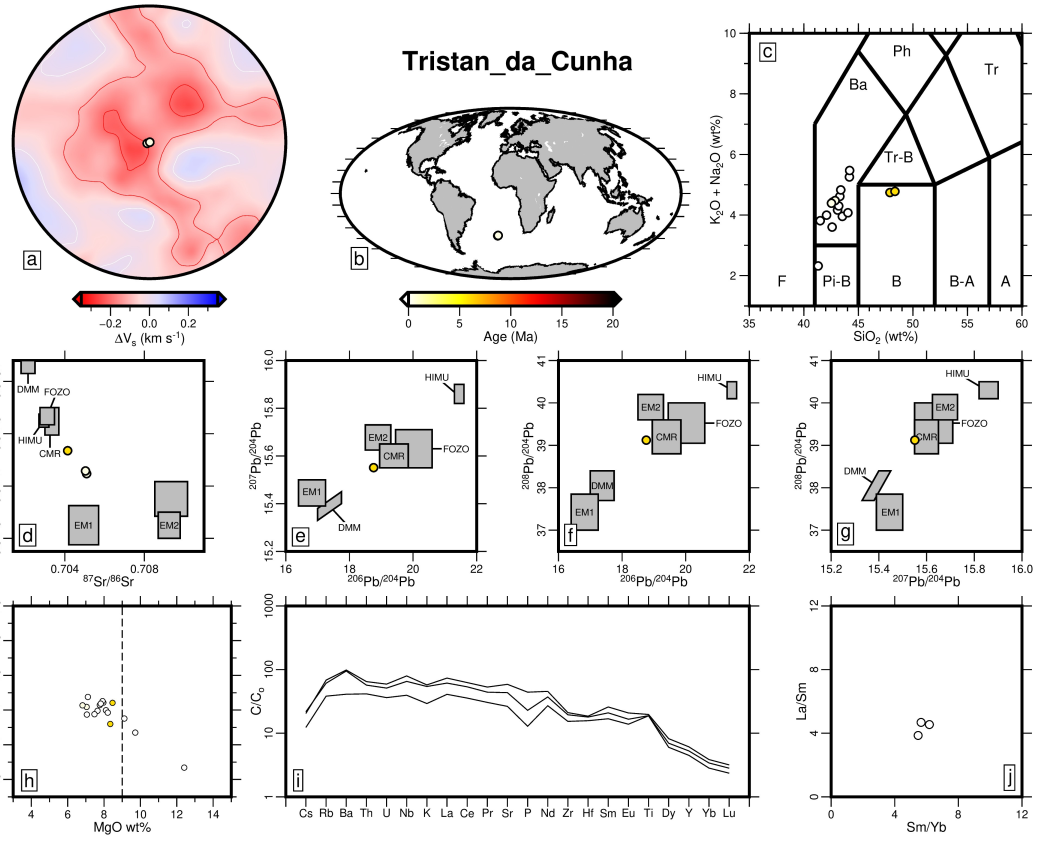
Tristan_da_Cunha
a
b
c
d
e
f
g
h
i
j
ΔVs (km s-1)
Age (Ma)
SiO2 (wt%)
K2O + Na2O (wt%)
Ph
Tr
Ba
Tr-B
F
Pi-B
B
B-A
A
143Nd/144Nd
87Sr/86Sr
207Pb/204Pb
206Pb/204Pb
208Pb/204Pb
DMM
FOZO
HIMU
CMR
EM1
EM2
Al2O3 wt%
MgO wt%
C/Co
Cs Rb Ba Th U Nb K La Ce Pr Sr P Nd Zr Hf Sm Eu Ti Dy Y Yb Lu
La/Sm
Sm/Yb

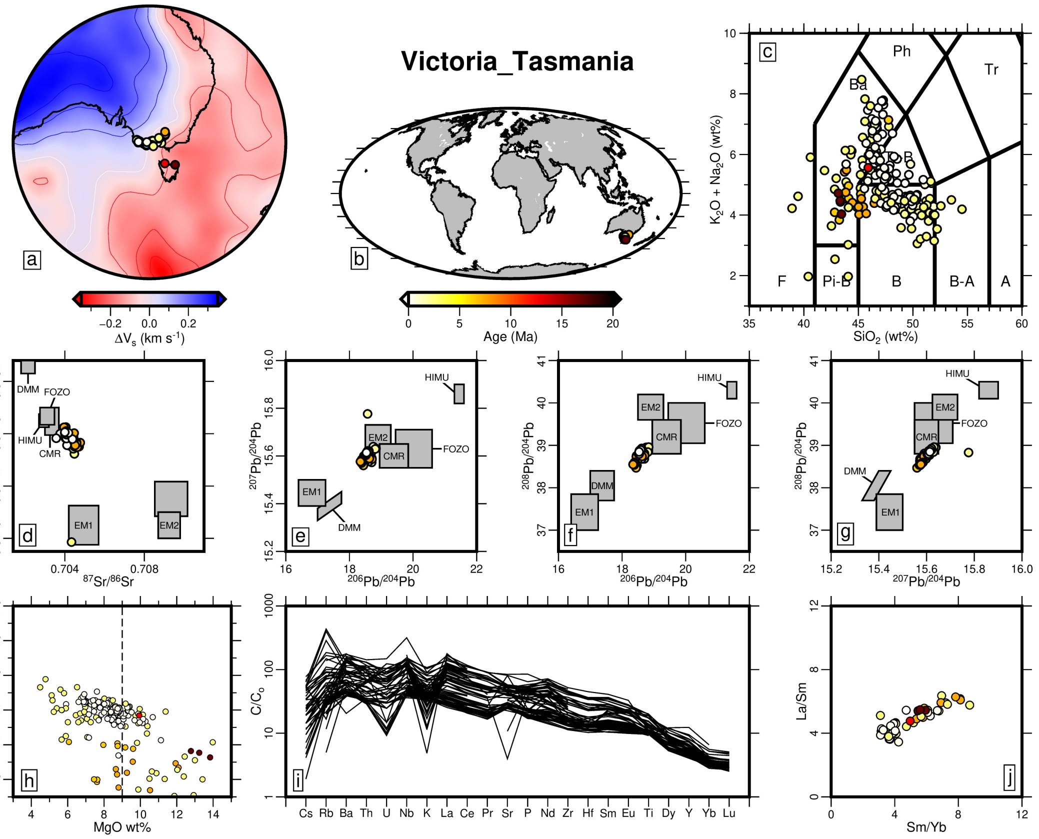
Victoria_Tasmania
a
b
c
d
e
f
g
h
i
j
ΔVs (km s-1)
Age (Ma)
SiO2 (wt%)
K2O + Na2O (wt%)
Ph
Tr
Ba
F
Pi-B
B
B-A
A
143Nd/144Nd
87Sr/86Sr
207Pb/204Pb
206Pb/204Pb
208Pb/204Pb
DMM
FOZO
HIMU
CMR
EM1
EM2
Al2O3 wt%
MgO wt%
C/Co
Cs Rb Ba Th U Nb K La Ce Pr Sr P Nd Zr Hf Sm Eu Ti Dy Y Yb Lu
La/Sm
Sm/Yb

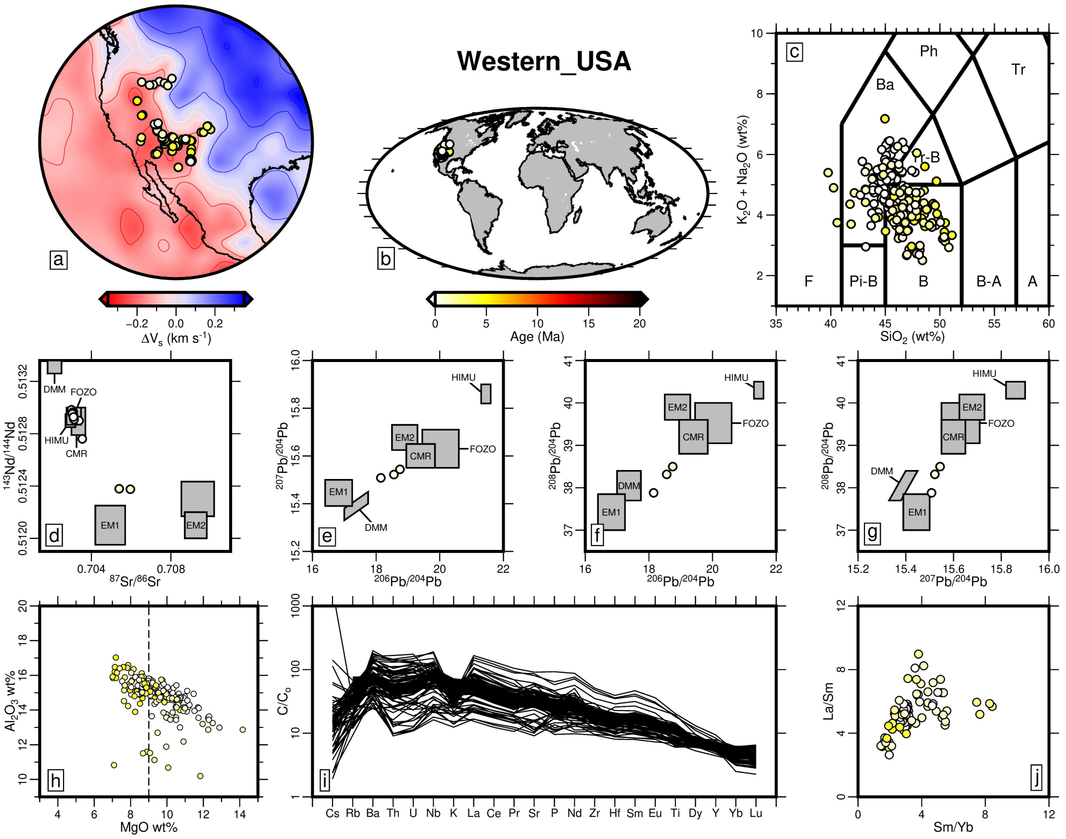
Western_USA
a
ΔVs (km s-1)
b
Age (Ma)
c
F
Pi-B
B
B-A
A
Ba
Ph
Tr
Tr-B
K2O + Na2O (wt%)
SiO2 (wt%)
d
143Nd/144Nd
87Sr/86Sr
DMM
FOZO
HIMU
CMR
EM1
EM2
e
207Pb/204Pb
206Pb/204Pb
f
208Pb/204Pb
206Pb/204Pb
g
208Pb/204Pb
207Pb/204Pb
h
Al2O3 wt%
MgO wt%
i
C/Co
Cs Rb Ba Th U Nb K La Ce Pr Sr P Nd Zr Hf Sm Eu Ti Dy Y Yb Lu
j
La/Sm
Sm/Yb

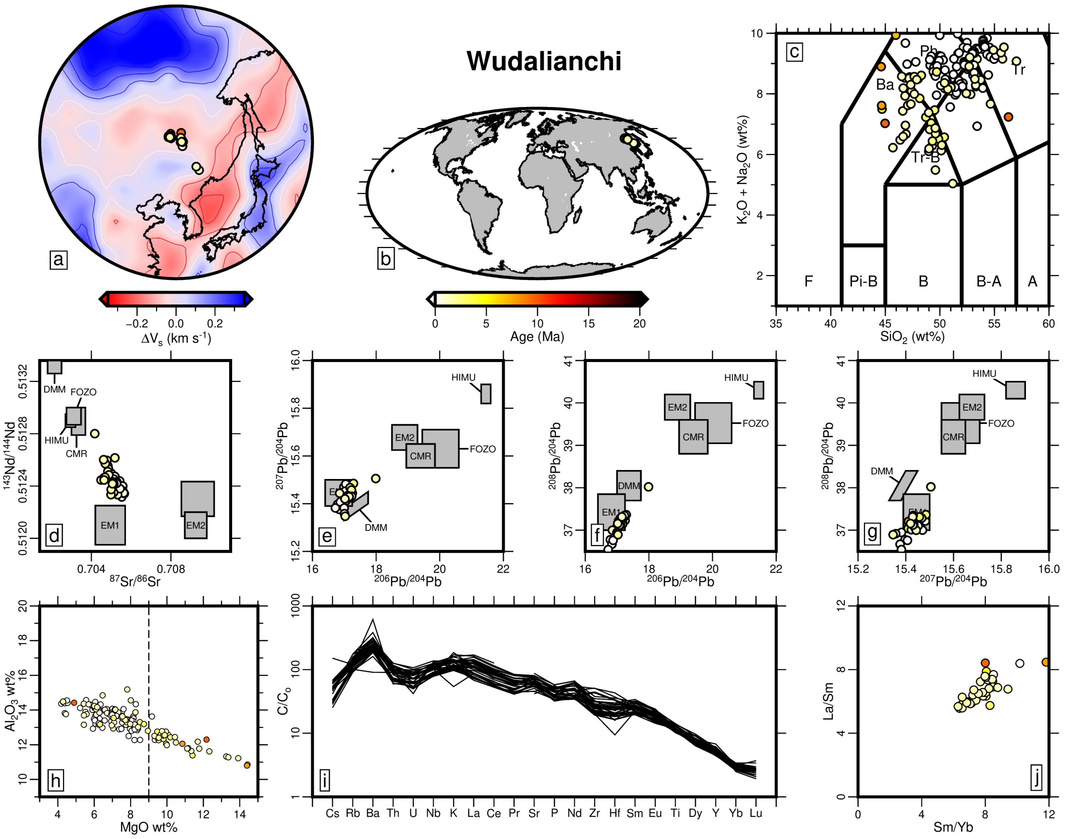
Wudalianchi
a
ΔVs (km s-1)
b
Age (Ma)
c
F
Pi-B
B
B-A
A
Ba
Ph
Tr
Tr-B
K2O + Na2O (wt%)
SiO2 (wt%)
d
143Nd/144Nd
87Sr/86Sr
DMM
FOZO
HIMU
CMR
EM1
EM2
e
207Pb/204Pb
206Pb/204Pb
f
208Pb/204Pb
206Pb/204Pb
g
208Pb/204Pb
207Pb/204Pb
h
Al2O3 wt%
MgO wt%
i
C/Co
Cs Rb Ba Th U Nb K La Ce Pr Sr P Nd Zr Hf Sm Eu Ti Dy Y Yb Lu
j
La/Sm
Sm/Yb

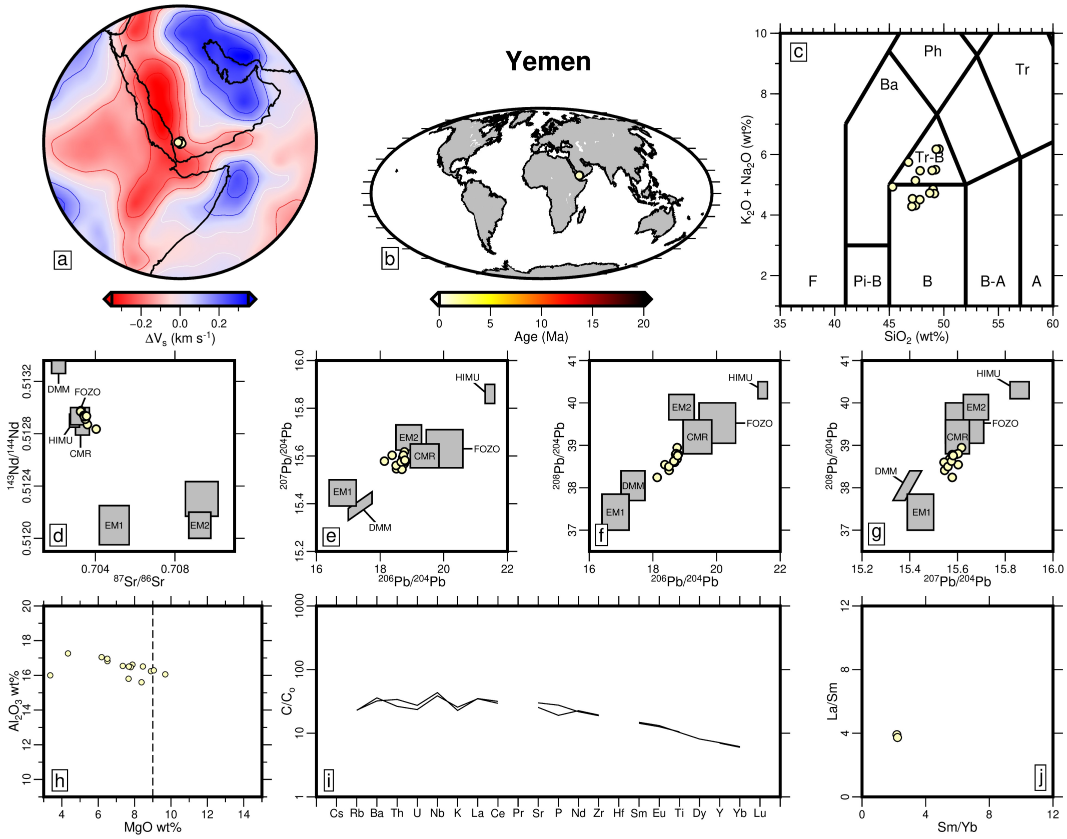
Yemen
a
b
c
d
e
f
g
h
i
j
ΔV$_s$ (km s^{-1})
Age (Ma)
$K_2O + Na_2O$ (wt%)
SiO_2 (wt%)
F
Pi-B
B
B-A
A
Ba
Ph
Tr
Tr-B
$^{143}Nd/^{144}Nd$
$^{87}Sr/^{86}Sr$
$^{207}Pb/^{204}Pb$
$^{206}Pb/^{204}Pb$
$^{208}Pb/^{204}Pb$
DMM
FOZO
HIMU
CMR
EM1
EM2
Al_2O_3 wt%
MgO wt%
C/C$_0$
Cs Rb Ba Th U Nb K La Ce Pr Sr P Nd Zr Hf Sm Eu Ti Dy Y Yb Lu
La/Sm
Sm/Yb

3 Supplementary Notes

3.1 Intraplate Geochemical Database

In order to analyze the relationship between intraplate volcanism and mantle temperature, a globally extensive database of $> 20,000$ Neogene and Quaternary geochemical samples has been compiled. Individual studies that comprise this database of continental and oceanic regions are listed in Tables 1 and 2, respectively. The GEOROC database hosted at `www.georoc.edu` was exploited as a starting point but many studies that were not included in this database have also been added by us. For studies that do not include radiometric dates, stratigraphic age constraints were used that encompass the range of ages stated in each individual study. In those studies where sample grid references are not provided, latitude and longitudes were chosen by extracting sample locations from relevant maps. Our global database also includes areas adjacent to actively rifting and subducting plate boundaries (e.g. Anatolia, western North America and East African Rift). A combination of geochemical review papers and the conclusions from individual studies were used to identify and remove samples that were probably generated by subduction zone processes (Table 3). To ensure trace element data quality, only those analyses published since 1990 were included. A comprehensive list of sample descriptions and compositions is provided in Supplementary Database 1. Several studies of Madagascar, Harrat Rahat and eastern Australia compiled within Database 1 are yet to be published by the contributing authors. These data are not included within Supplementary Database 1 [725, 902, 60].

3.2 Tomographic Models

The CAM2016Vsv-200 model is the result of a global study of upper mantle structure generated by waveform modeling of > 2 million vertical component Rayleigh waveforms [468]. This model prescribes an *a priori* crustal structure that is based upon the Crust1.0 compilation [567]. In contrast, the SEMUCB-WM1 model includes whole mantle coverage that is based upon a hybrid full waveform inversion of body and surface waves with periods of 32–300 s and 60–400 s, respectively [347]. This model exploits an adapted version of the Crust2.0 compilation designed to fit surface-wave group dispersion data at depths of 30–60 km [81]. Finally, the S40RTS model is a whole mantle representation. It includes surface waves with periods of 40–275 s, teleseismic body-wave travel times, and normal-mode splitting functions [830]. In this model, crust was accounted for using the Crust2.0 compilation [81]. Spatial resolution of all four tomographic models relies upon the global distribution of earthquakes and stations, which in turn determines the density of great circle paths for a given continent. In general, these models have horizontal and vertical resolutions of 200–600 km and 25–50 km, respectively. ΔV_s for these three models are show as velocity relative to the Preliminary Reference Earth Model [i.e. PREM; 302]. Constants used to convert the SL2013sv model into temperature using the V_s-T parameterization of ref. [475] is shown in Table 4.

3.3 Additional Spatial Correlation Tests

Figure 2a and b of main text demonstrate that the bulk of Neogene-Quaternary intraplate volcanism is concentrated within regions where negative ΔV_s anomalies occur at a depth of 150 ± 25 km and where the lithosphere < 100 km thick. These results are generated using the SL2013sv tomographic model and are replotted on Supplementary Figures 1a and d. The spatial association between intraplate volcanism and slow velocity anomalies is also evident using different global tomographic models of the upper mantle that include the CAM2016-Vsv-200, SEMUCB-WM1 and S40RTS models [Supplementary Figures 1b, c and d; 468, 347, 830]. 72%, 87% and 83% of bins containing intraplate samples are underlain by slow seismic velocities within the CAM2016-Vsv-200, SEMUCB-WM1 and S40RTS models, respectively. These values are significantly higher than the global percentage of bins that occur over slow velocities for each tomographic model (53%, 60% and 60%, respectively). The CAM2016-Vsv-200 model has been used to

calculate lithospheric thickness [804]. For this lithospheric thickness model, 92% of intraplate volcanic samples occur above lithosphere that is < 100 km thick, which is significantly higher than the global average (64%; Supplementary Figure 1f).

3.4 Additional Geochemical Correlation Tests

There is a positive correlation between La/Sm values and average ΔV_s values at depth of 150 ± 25 km for four different tomographic models of upper mantle ($R = 0.56$–0.72; Supplementary Figure 2). Since La/Sm and ΔV_s both decrease as temperature increases, this positive correlation suggests that intraplate volcanic compositions are principally modulated by asthenospheric temperature. Correlations shown in Supplementary Figure 2 are based upon a filtered, binned and averaged version of Database 1 (i.e. number of samples > 5, $9 <$ MgO < 14.5 wt%, age < 10 Ma, < 400 km from point of eruption, bin size of 1°). To ensure that these choices do not give rise to artificial correlation, these filtering parameters are varied together with the tomographic model. Supplementary Figure 3a shows how the value of R varies as bins with limited measurements are progressively removed. The value of R increases as the minimum number of samples required for a bin to be included increases from 1 to 5–10. Increasing this cut-off above ~ 10 causes the value of R to either flatten out or to start to decrease. As the minimum number of required samples increases, the number of bins that exceed this threshold decreases. This decrease causes the value of R required for the correlation to be significant to increase until the observed correlation between La/Sm and ΔV_s values becomes insignificant (i.e. when the gray dashed line exceeds the value of R for each tomographic model; Supplementary Figure 3a). Generally, there is a small increase in the value of R as the minimum required value of MgO wt% increases (Supplementary Figure 3b). There is little change in the value of R as a function of the maximum age threshold up to 15 Ma. When the threshold exceeds 15 Ma, there is a sharp decrease in correlation (Supplementary Figure 3c). There is no significant change in correlation as a function of maximum distance from point of eruption based on present-day plate speeds (Supplementary Figure 3d). Finally, as bin size decreases from 5° to 0.5° a slight increase in correlation is observed.

In Supplementary Figure 3f, the correlation between La/Sm and V_s for each tomographic model is shown as a function of depth. For all four models, the value of R is greatest between 100 and 175 km (i.e. $R > 0.6$). With the exception of the S40RTS model, the value of R sharply decreases to negligible values at depths > 250 km. The correlation between La/Sm and the S40RTS model slowly decreases at depths > 175 km. Since ray coverage is poor in the mid-mantle, whole-mantle models such as the S40RTS model are typically more strongly damped and therefore have poorer resolution than upper mantle models such as the SL2013sv model. Therefore, the extension of the correlation between La/Sm and ΔV_s values to deeper levels of the S40RTS model may result from smearing caused by the poorer resolution of this model. Overall, the choice of filtering parameters and the choice of tomographic model do not significantly affect the correlation between La/Sm and ΔV_s values at 150 ± 25 km.

3.5 Assessing Effects of Subduction and Rifting

Several provinces from Database 1 occur on oceanic islands close to, or directly above, mid-oceanic ridges (MORs). Lavas sampled from these islands can have anomalously low La/Sm ratios for a given ΔV_s since they are sourced from mantle that lies beneath thin, newly-formed, lithosphere. 1° bins containing these low La/Sm samples can give rise to an artificially strong correlation between La/Sm and ΔV_s values. However, when all bins from intraplate volcanic provinces in close proximity to MORs (e.g. Azores, Easter Island, Galapagos, Iceland, Jan Mayen) are removed from Database 1, the value of R is only modestly reduced ($R = 0.66$; Supplementary Figure 4a).

Flux of material from subducting slabs can significantly alter the composition and lithology of an overlying mantle wedge. If the slab rolls back, detaches or tears, asthenospheric mantle that upwells into the vacated space can generate intraplate volcanism. If the overlying mantle wedge retains chemical traces from subduction, the resultant compositions of melts generated within this region can be influenced.

Addition of volatiles, such as water, to the mantle wedge lowers the solidus and causes additional melting to occur for a given potential temperature [521]. In Supplementary Figure 4b, Database 1 is filtered to remove volcanic provinces that occur above areas of recent subduction (e.g. Anatolia, America, Canada, China, Italy, Iran, Mexico, New Zealand, Patagonia, southern Spain). There is no appreciable difference in correlation when these areas are excluded ($R = 0.65$; Supplementary Figure 4b).

3.6 Principal Component Analysis

A detailed description of how principal component analysis is carried out, together with a summary of the results obtained, is provided within the main text. Here, we present correlations between each principal component and La/Sm, ΔV_s and εNd values for a given binning configuration (Supplementary Figure 5). The first Principal Component, P_1, correlates positively with La/Sm and ΔV_s, but correlates negatively with ϵNd (Supplementary Figure 5a,d,g). The correlation coefficient, R, for each of these relationships is 0.87, 0.56 and -0.51, respectively. P_2 does not correlate significantly with La/Sm, ΔV_s or ϵNd (Supplementary Figure 5b,e,h). P_3 does not correlate significantly with either La/Sm or ΔV_s. However, bins with low values of La/Sm show a greater range of values of P_3 (Supplementary Figure 5c,f,i). This pattern may reflect small-scale heterogeneity within the upper mantle, which has less of an effect on melt composition when melting increases. Samples with positive values of P_3 have high concentrations of K and Ba that could reflect the role of slab-derived fluids during melting. Bins with negative P_3 values are depleted in K, which may indicate partial melting of metasomatized lithospheric mantle material [894, 792]. These processes give rise to a very weak negative correlation between P_3 and ϵNd since slab-derived fluids have low values of ϵNd ($R = -0.31$).

The principal component analysis described in the main text uses measurements that has been binned and averaged. Here, we show that the results of this analysis for raw measurements prior to binning do not significantly differ from those generated when binned values are used (Supplementary Figure 6). P_1 accounts for 77% of variance and it is the only component that is positively weighted for all elements. P_2 describes 15% of the total variance and it is dominated by variations in Yb. P_3 represents 8% of the overall variability and it is principally controlled by variations in K concentrations. Only P_1 correlates positively with V_s and negatively with ϵNd. With unbinned measurements, the correlation between each principal component and MgO wt% can be determined. Significantly, P_1 and P_2 do not correlate with MgO wt%. We therefore do not consider fractionation or accumulation of olivine or pyroxene to have had an important effect on the correlation between La/Sm and ΔV_s values.

3.7 Synthetic Principal Component Analysis

Within the main text, principal component analysis that has been carried out on a synthetic database is described. Here we show the resultant correlations between three principal components, M_1, M_2 and M_3, and three input parameters used to generate synthetic models (T_p, lithospheric thickness, εNd). M_1 strongly correlates with T_p and does not significantly correlate with lithospheric thickness or mantle composition (Supplementary Figure 7a,d,g). It is reasonable to conclude that both M_1 and P_1 are primarily sensitive to changes in T_p. M_2 strongly correlates with lithospheric thickness but does not correlate with either T_p or mantle composition (Supplementary Figure 7b,e,h). Finally, M_3 does not consistently correlate with T_p, lithospheric thickness or εNd (Supplementary Figure 7c,f,i).

A second test compares the principal components calculated for Database 1 with T_p and lithospheric thickness estimates obtained from geochemical and tomographic analysis. As expected, a correlation between P_1 and both geochemically and tomographically derived T_p values is observed (Supplementary Figure 8a,c). A correlation is also observed between P_1 and geochemically derived lithospheric thickness estimates (Supplementary Figure 8b). However, the bulk of lithospheric thickness estimates fall within a 10 km range, close to the uncertainty of any individual sample. It is therefore difficult to assess the significance of this correlation. P_2 has low variance and does not correlate with any estimates of either asthenospheric temperature or lithospheric thickness.

3.8 INVMEL Forward Modeling Method

Tables 4–9 present parameters used in INVMEL forward modeling runs. The complete procedure is described in Methods section of the main text.

Geochemical Temperature Estimates for 1° Bins

Methodology for estimating mantle potential temperature, T_p, and lithospheric thickness, a, from rare earth element (REE) concentrations is described in Methods section of main text. Results for each 1° bin shown on Figure 5a of main text are shown for completeness in Supplementary Figure 9 and Supplementary Table 10.

Regional Geochemical Data

For completeness, we include graphical representations of geochemical analyses collated for each region of Database 1. Regions are displayed in alphabetical order in Supplementary Figure 10.

References

[1] Abbott, M., 1969. Petrology of the Nandewar volcano, NSW, Australia, *Contributions to Mineralogy and Petrology*, **20**(2), 115–134.

[2] Abdel-Rahman, A.-F. M. & Nassar, P. E., 2004. Cenozoic volcanism in the Middle East: petrogenesis of alkali basalts from northern Lebanon, *Geological Magazine*, **141**(5), 545–563.

[3] Ablay, G., Carroll, M., Palmer, M., Martí, J., & Sparks, R., 1998. Basanite–phonolite lineages of the Teide–Pico Viejo volcanic complex, Tenerife, Canary Islands, *Journal of Petrology*, **39**(5), 905–936.

[4] Abraham, A. C., Francis, D., & Polvé, M., 2001. Recent alkaline basalts as probes of the lithospheric mantle roots of the northern Canadian Cordillera, *Chemical Geology*, **175**(3-4), 361–386.

[5] Abraham, A.-C., Francis, D., & Polvé, M., 2005. Origin of Recent alkaline lavas by lithospheric thinning beneath the northern Canadian Cordillera, *Canadian Journal of Earth Sciences*, **42**(6), 1073–1095.

[6] Abratis, M., Schmincke, H. U., & Hansteen, T., 2002. Composition and evolution of submarine volcanic rocks from the central and western Canary Islands, *International Journal of Earth Sciences*, **91**(4), 562–582.

[7] Abratis, M., Mädler, J., Hautmann, S., Leyk, H. J., Meyer, R., Lippolt, H. J., & Viereck-Götte, L., 2007. Two distinct Miocene age ranges of basaltic rocks from the Rhön and Heldburg areas (Germany) based on $^{40}Ar/^{39}Ar$ step heating data, *Chemie der Erde*, **67**(2), 133–150.

[8] Adiyaman, O., Chorowicz, J., Arnaud, O. N., Gündogdu, M. N., & Gourgaud, A., 2001. Late Cenozoic tectonics and volcanism along the North Anatolian Fault: new structural and geochemical data, *Tectonophysics*, **338**, 135–165.

[9] Agostini, S., Doglioni, C., Innocenti, F., Manetti, P., Tonarini, S., & Savasçin, M., 2007. The transition from subduction-related to intraplate Neogene magmatism in the Western Anatolia and Aegean area, in *Cenzozoic volcanism in the Mediterranean Area*, edited by L. Beccaluva, G. Bianchini, & M. Wilson, pp. 1–15, The Geological Society of America, Boulder, Colorado, Geological Society of America Special Paper 418.

[10] Aguillón-Robles, A., Caimus, T., Benoit, M., Bellon, H., Maury, R. C., Cotten, J., Bourgois, J., & Michaud, F., 2001. Late Miocene adakites and Nb- enriched basalts from Vizcaino Peninsula, Mexico: Indicators of East Pacific Rise subduction below southern Baja California?, *Geology*, **29**(6), 531–534.

[11] Ahijado, A., Casillas, R., & Hernández-Pacheco, A., 2001. The dyke swarms of the Amanay Massif, Fuerteventura, Canary Islands (Spain), *Journal of Asian Earth Sciences*, **19**, 333–345.

[12] Ahmadzadeh, G., Jahangiri, A., Lentz, D., & Mojtahedi, M., 2010. Petrogenesis of Plio-Quaternary post-collisional ultrapotassic volcanism in NW of Marand, NW Iran, *Journal of Asian Earth Sciences*, **39**(1-2), 37–50.

[13] Ahmed, A. H., Moghazi, A. K. M., Moufti, M. R., Dawood, Y. H., & Ali, K. A., 2016. Nature of the lithospheric mantle beneath the Arabian Shield and genesis of Al-spinel micropods: Evidence from the mantle xenoliths of Harrat Kishb, Western Saudi Arabia, *Lithos*, **240**, 119–139.

[14] Aït-Hamou, F., Dautria, J.-M., Cantagrel, J.-M., Dostal, J., & Briqueu, L., 2000. New geochronological and isotopic data on the Cenozoic volcanism of Ahaggar (southern Algeria): evidence for a mantle plume., *Geochemistry*, **330**, 829–836.

[15] Aka, F. T., Yokoyama, T., Kusakabe, M., Nakamura, E., Tanyileke, G., Ateba, B., Ngako, V., Nnange, J., & Hell, J., 2008. U-series dating of Lake Nyos maar basalts, Cameroon (West Africa): Implications for potential hazards on the Lake Nyos dam, *Journal of Volcanology and Geothermal Research*, **176**(2), 212–224.

[16] Al-Amri, A., Fnais, M., Abdel-Rahman, K., Mogren, S., & Al-Dabbagh, M., 2012. Geochronological dating and stratigraphic sequences of Harrat Lunayyir, NW Saudi Arabia, *International Journal of Physical Sciences*, **7**(20), 2791–2805.

[17] Al Kwatli, M. A., Gillot, P. Y., Zeyen, H., Hildenbrand, A., & Al Gharib, I., 2012. Volcano-tectonic evolution of the northern part of the Arabian plate in the light of new K–Ar ages and remote sensing: Harrat Ash Shaam volcanic province (Syria), *Tectonophysics*, **580**, 192–207.

[18] Al-Malabeh, A., 1994. Geochemistry of two volcanic cones from the intra-continental plateau basalt of Harra El-Jabban, NE-Jordan, *Geochemical Journal*, **28**(6), 517–540.

[19] Albarede, F. & Tamagnan, V., 1988. Modelling the recent geochemical evolution of the piton de la fournaise volcano, Reunion Island, 1931 - 1986, *Journal of Petrology*, **29**(5), 997–1030.

[20] Albarède, F., Luais, B., Fitton, G., Semet, M., Kaminski, E., Upton, B. G., Bachèlery, P., & Cheminée, J. L., 1997. The geochemical regimes of piton de la Fournaise volcano (Réunion) during the last 530000 years, *Journal of Petrology*, **38**(2), 171–201.

[21] Albert, H., Costa, F., & Martí, J., 2015. Timing of magmatic processes and unrest associated with mafic historical monogenetic eruptions in Tenerife Island, *Journal of Petrology*, **56**(10), 1945–1966.

[22] Aldanmaz, E., Pearce, J. A., Thirwall, M. F., & Mitchell, J. G., 2000. Petrogenetic evolution of late Cenozoic, post-collision volcanism in western Anatolia, Turkey, *Journal of Volcanology and Geothermal Research*, **102**, 67–95.

[23] Aldanmaz, E., Köprübaşı, Gürer, O. F., Kaymakçı, N., & Gourgaud, A., 2006. Geochemical constraints on the Cenozoic, OIB-type alkaline volcanic rocks of NW Turkey: Implications for mantle sources and melting processes, *Lithos*, **86**, 50–76.

[24] Aldanmaz, E., Pickard, M., Meisel, T., Altunkaynak, c., Sayıt, K., Şen, P., Hanan, B. B., & Furman, T., 2015. Source components and magmatic processes in the genesis of Miocene to Quaternary lavas in western Turkey: constraints from HSE distribution and Hf-Pb-Os isotopes, *Contributions to Mineral Petrology*, **170**, 23.

[25] Alene, M., Hart, W. K., Saylor, B. Z., Deino, A., Mertzman, S., Haile-Selassie, Y., & Gibert, L. B., 2017. Geochemistry of Woranso–Mille Pliocene basalts from west-central Afar, Ethiopia: Implications for mantle source characteristics and rift evolution, *Lithos*, **282-283**, 187–200.

[26] Ali, S., Ntaflos, T., & Upton, B. G., 2013. Petrogenesis and mantle source characteristics of Quaternary alkaline mafic lavas in the western Carpathian-Pannonian Region, Styria, Austria, *Chemical Geology*, **337-338**, 99–113.

[27] Alıcı, P., Temel, A., Gourgaud, A., Vidal, P., & Gündogdu, M. N., 2001. Quaternary tholeiitic to alkaline volcanism in the Karasu Valley, Dead Sea Rift Zone, Southeast Turkey: Sr-Nd-Pb-O isotopic and trace-element approached to crust-mantle interaction, *International Geology Review*, **43**, 120–138.

[28] Alıcı, P., Temel, A., & Gourgaud, A., 2002. Pb-Nd-Sr isotope and trace element geochemistry of Quaternary extension-related alkaline volcanism: a case study of Kula region (western Anatolia, Turkey), *Journal of Volcanology and Geothermal Research*, **115**, 487–510.

[29] Alıcı Şen, P., Temel, A., & Gourgaud, A., 2004. Petrogenetic modelling of Quaternary post-collisional volcanism: a case study of central and eastern Anatolia, *Geological Magazine*, **141**(1), 81–98.

[30] Allègre, C. J., Dupré, B., Lambert, B., & Richard, P., 1981. The subcontinental versus suboceanic debale, 1. Lead-Neodymium-Strontium isotopes in promary alcali basalts from a shield area: the Anaggar volcanoc suite, *Earth. Planet. Sci. Lett.*, **52**, 85–92.

[31] Allen, M. B., Kheirkhah, M., Neill, I., Emami, M. H., & McLeod, C. L., 2013. Generation of arc andwithin-plate chemical signatures in collision zone magmatism: Quaternary lavas from kurdistan province, Iran, *Journal of Petrology*, **54**(5), 887–911.

[32] Altherr, R., Henjes-Kunst, F., & Baumann, A., 1990. Asthenosphere versus lithosphere as possible sources for basaltic magmas erupted during formation of the Red Sea: constraints from Sr, Pb and Nd isotopes, *Earth and Planetary Science Letters*, **96**(3-4), 269–286.

[33] An, A.-R., Choi, S. H., Yu, Y., & Lee, D.-C., 2017. Petrogenesis of Late Cenozoic basaltic rocks from southern Vietnam, *Lithos*, **272**, 192–204.

[34] Ancochea, E., Hernán, F., Huertas, M. J., & Brändle, J. L., 2012. A basic radial dike swarm of Boa Vista (Cape Verde Archipelago); its significance in the evolution of the island, *Journal of Volcanology and Geothermal Research*, **243**, 24–37.

[35] Ancuta, L. D., Zeitler, P. K., Idleman, B. D., & Jordan, B. T., 2018. Whole-rock $^{40}Ar/^{39}Ar$ geochronology, geochemistry, and stratigraphy of intraplate Cenozoic volcanic rocks, central Mongolia, *Bulletin*, **130**(7-8), 1397–1408.

[36] Anderson, R. G., Resnick, J., Russell, J. K., Woodsworth, G. J., Villeneuve, M. E., & Grainger, N. C., 2001. The Cheslatta Lake suite: Miocene mafic, alkaline magmatism in central British Columbia, *Canadian Journal of Earth Sciences*, **38**(4), 697–717.

[37] Andreeva, O. A., Yarmolyuk, V. V., Andreeva, I. A., Ji, J. Q., & Li, W. R., 2014. The composition and sources of magmas of Changbaishan Tianchi volcano (China-North Korea), *Doklady Earth Sciences*, **456**(1), 572–578.

[38] Aoki, K.-I., Yoshida, T., Yusa, K., & Nakamura, Y., 1985. Journal of Volcanology and Geothermal Research, 25 (1985) 1–28, *Journal of Volcanology and Geothermal Research*, **25**, 1–28.

[39] Arevalo Jr, R., McDonough, W. F., Stracke, A., Willbold, M., Ireland, T. J., & Walker, R. J., 2013. Simplified mantle architecture and distribution of radiogenic power, *Geochemistry, Geophysics, Geosystems*, **14**(7), 2265–2285.

[40] Arger, J., Mitchell, J., & Westaway, R. W. C., 2000. Neogene and Quaternary volcanism of southeastern Turkey, in *Tectonics and Magmatism in Turkey and the Surrounding Area*, edited by E. Bozkurt, W. J. A., & J. D. A. Piper, pp. 459–487, The Geological Society of London, London, Special Publications 173.

[41] Argus, D. F., Gordon, R. G., & DeMets, C., 2011. Geologically current motion of 56 plates relative to the no-net-rotation reference frame, *Geochemistry, Geophysics, Geosystems*, **12**(11).

[42] Armienti, P., Tonarini, S., D'Orazio, M., & Innocenti, F., 2004. Genesis and evolution of Mt. Etna alkaline lavas: petrological and Sr-Nd-B isotope constraints, *Periodico di Mineralogia*, **73**(Special issue 1), 29–52.

[43] Arnaud, N., Vidal, P., Tapponnier, P., Matte, P., & Deng, W., 1992. The high K_2O volcanism of northwestern Tibet: Geochemistry and tectonic implications, *Earth and Planetary Science Letters*, **111**(2-4), 351–367.

[44] Atkinson, A., Griffin, T., & Stephenson, P., 1975. A major lava tube system from Undara Volcano, North Queensland, *Bulletin Volcanologique*, **39**(2), 266–293.

[45] Avanzinelli, R., Bindi, L., Menchetti, S., & Conticelli, S., 2004. Crystallisation and genesis of peralkaline magmas from Pantelleria Volcano, Italy: An integrated petrological and crystal-chemical study, *Lithos*, **73**(1-2), 41–69.

[46] Aviado, K. B., Rilling-Hall, S., Bryce, J. G., & Mukasa, S. B., 2015. Submarine and subaerial lavas in the West Antarctic Rift System: Temporal record of shifting magma source components from the lithosphere and asthenosphere, *Geochemistry Geophysics Geosystems*, **18**(1-2), 1541–1576.

[47] Ayalew, D., 1999. *Pétrologie et géochimie des ignimbrites des hauts plateaux éthiopiens: source, chronologie et impact environnmental [ie environnemental]*, Ph.D. thesis, Institut National Polytechnique de Lorraine.

[48] Ayalew, D. & Gibson, S. A., 2009. Head-to-tail transition of the Afar mantle plume: Geochemical evidence from a Miocene bimodal basalt-rhyolite succession in the Ethiopian Large Igneous Province, *Lithos*, **112**(3-4), 461–476.

[49] Ayalew, D., Marty, B., Barbey, P., Yirgu, G., & Ketefo, E., 2006. Sub-lithospheric source for Quaternary alkaline Tepi shield, southwest Ethiopia, *Geochemical Journal*, **40**(1), 47–56.

[50] Ayalew, D., Jung, S., Romer, R. L., Kersten, F., Pfänder, J. A., & Garbe-Schönberg, D., 2016. Petrogenesis and origin of modern Ethiopian rift basalts: Constraints from isotope and trace element geochemistry, *Lithos*, **258-259**, 1–14.

[51] Aydar, E. & Gourgaud, A., 2002. Garnet-bearing basalts: an example from Mt. Hasan, central Anatolia, Turkey, *Mineralogy and Petrology*, **75**(3-4), 185–201.

[52] Aydin, F., Karsli, O., & Chen, B., 2008. Petrogenesis of the Neogene alkaline volcanics with implications for post-collisional lithospheric thinning of the Eastern Pontides, NE Turkey, *Lithos*, **104**, 249–266.

[53] Azzouni-Sekkal, A., Bonin, B., Benhallou, A., Yahiaoui, R., & Liégeois, J.-P., 2007. Cenozoic alkaline volcanism of the Atakor massif, Hoggar, Algeria, *Special Paper 418: Cenozoic Volcanism in the Mediterranean Area*, **2418**(December), 321–340.

[54] Azzouzi, M. E., Maury, R. C., Bellon, H., Youbi, N., Cotten, J., & Kharbouch, F., 2010. Petrology and K-Ar chronology of the Neogene-Quaternary Middle Atlas basaltic province, Morocco, *Bulletin de la Societe Geologique de France*, **181**(3), 243–257.

[55] Bailey, K., Lloyd, F., Kearns, S., Stoppa, F., Eby, N., & Woolley, A., 2005. Melilitite at Fort Portal, Uganda: Another dimension to the carbonate volcanism, *Lithos*, **85**(1-4 SPEC. ISS.), 15–25.

[56] Baker, B. H., Goles, G. G., Leeman, W. P., & Lindstrom, M. M., 1977. Geochemistry and petrogenesis of a basalt-benmoreite-trachyte suite from the southern part of the Gregory Rift, Kenya, *Contributions to Mineralogy and Petrology*, **64**(3), 303–332.

[57] Baker, J., Snee, L., & Menzies, M., 1996. A brief Oligocene period of flood volcanism in Yemen: implications for the duration and rate of continental flood volcanism at the Afro-Arabian triple junction, *Earth and Planetary Science Letters*, **138**(1-4), 39–55.

[58] Baker, J., Menzies, M., Thirlwall, M., & Macpherson, C., 1997. Petrogenesis of Quaternary intraplate volcanism, Sana'a, Yemen: implications for plume-lithosphere interaction and polybaric melt hybridization, *Journal of Petrology*, **38**(10), 1359–1390.

[59] Bakhsh, R. A., 2015. Pliocene–Quaternary basalts from the Harrat Tufail, western Saudi Arabia: Recycling of ancient oceanic slabs and generation of alkaline intra-plate magma, *Journal of African Earth Sciences*, **112**, 37–54.

[60] Ball, P. W., 2020. *Global Relationships Between Intraplate Magmatism and Dynamic Topography*, Ph.D. thesis, University of Cambridge.

[61] Ball, P. W., White, N. J., Masoud, A., Nixon, S., Hoggard, M., Maclennan, J., Stuart, F., Oppenheimer, C., & Kröpelin, S., 2019. Quantifying asthenospheric and lithospheric controls on mafic magmatism across North Africa, *Geochemistry, Geophysics, Geosystems*, **20**, 3520–3555.

[62] Ballentine, C., Lee, D.-C., & Halliday, A., 1997. Hafnium isotopic studies of the Cameroon line and new HIMU paradoxes, *Chemical Geology*, **139**(1-4), 111–124.

[63] Barberi, F., Bonatti, E., Marinelli, G., & Varet, J., 1974. Transverse tectonics during the split of a continent: data from the Afar rift, *Tectonophysics*, **23**, 17–29.

[64] Barberi, F., Ferrara, G., Santacroce, R., Treuil, M., & Varet, J., 1975. A Transitional Basalt-Pantellerite Sequence of Fractional Crystallization, the Boina Centre (Afar Rift, Ethiopia), *Journal of Petrology*, **16**(1), 22–56.

[65] Barberi, F., Capaldi, G., GA SPARINI, P., Marinelli, G., Santacroce, R., Scandone, R., Treuil, M., & Varet, J., 1980. Recent basaltic volcanism of Jordan and its implications on the geodynamic hi-story of the Dead Sea shear zone, *Geodynamic evolution of the Afro-Arabian Rift System*, **1**, 667–683.

[66] Barberio, M., Donati, C., Donato, P., Yirgu, G., Peccerillo, A., & Wu, T., 1999. Petrology and geochemistry of Quaternary magmatism in the northern sector of the Ethiopian Rift between Debre Zeit and Awash Park, *Acta Vulcanologica*, **11**, 69–82.

[67] Bardintzeff, J.-M., Leyrit, H., Guillou, H., Guille, G., Bonin, B., Giret, A., & Brousse, R., 1994. Transition between tholeiitic and alkali basalts: Petrographical and geochemical evidence from Fangataufa, Pacific Ocean, and Kerguelen, Indian Ocean, *Geochemical Journal*, **28**(6), 489–515.

[68] Bardintzeff, J. M., Liégeois, J. P., Bonin, B., Bellon, H., & Rasamimanana, G., 2010. Madagascar volcanic provinces linked to the Gondwana break-up: Geochemical and isotopic evidences for contrasting mantle sources, *Gondwana Research*, **18**(2-3), 295–314.

[69] Bardintzeff, J.-M., Deniel, C., Guillou, H., Platevoet, B., Telouk, P., & Oun, K. M., 2012. Miocene to recent alkaline volcanism between Al Haruj and Waw an Namous (southern Libya), *International Journal of Earth Sciences*, **101**(4), 1047–1063.

[70] Barker, A. K., Holm, P. M., Peate, D. W., & Baker, J. A., 2009. Geochemical stratigraphy of submarine lavas (3–5 Ma) from the Flamengos Valley, Santiago, southern Cape Verde islands, *Journal of Petrology*, **50**(1), 169–193.

[71] Barker, A. K., Holm, P. M., Peate, D. W., & Baker, J. A., 2010. A 5 million year record of compositional variations in mantle sources to magmatism on Santiago, southern Cape Verde archipelago, *Contributions to Mineralogy and Petrology*, **160**(1), 133–154.

[72] Barker, D. S. & Nixon, P. H., 1989. High-Ca, low-alkali carbonatite volcanism at Fort Portal, Uganda, *Contributions to Mineralogy and Petrology*, **103**(2), 166–177.

[73] Barling, J. & Goldstein, S. L., 1990. Extreme isotopic variations in Heard Island lavas and the nature of mantle reservoirs, *Nature*, **348**(6296), 59.

[74] Barling, J., Goldstein, S. L., & Nicholls, I. A., 1994. Geochemistry of Heard Island (southern Indian Ocean): characterization of an enriched mantle component and implications for enrichment of the sub-Indian Ocean mantle, *Journal of Petrology*, **35**(4), 1017–1053.

[75] Barr, S. & Cooper, M., 2013. Late Cenozoic basalt and gabbro in the subsurface in the Phetchabun Basin, Thailand: Implications for the Southeast Asian Volcanic Province, *Journal of Asian Earth Sciences*, **76**, 169–184.

[76] Barr, S. & MacDonald, A., 1981. Geochemistry and geochronology of late Cenozoic basalts of Southeast Asia, *Geological Society of America Bulletin*, **92**(8_Part_II), 1069–1142.

[77] Barrat, J. A., Fourcade, S., Jahn, B. M., Cheminée, J. L., & Capdevila, R., 1998. Isotope (Sr, Nd, Pb, O) and trace-element geochemistry of volcanics from the Erta'Ale range (Ethiopia), *Journal of Volcanology and Geothermal Research*, **80**(1-2), 85–100.

[78] Barrat, J. A., Joron, J. L., Taylor, R. N., Fourcade, S., Nesbitt, R. W., & Jahn, B. M., 2003. Geochemistry of basalts from Manda Hararo, Ethiopia: LREE-depleted basalts in Central Afar, *Lithos*, **69**(1-2), 1–13.

[79] Barry, T. L., 2003. Petrogenesis of Cenozoic Basalts from Mongolia: Evidence for the Role of Asthenospheric versus Metasomatized Lithospheric Mantle Sources, *Journal of Petrology*, **44**(1), 55–91.

[80] Barsczus, H. G. & Liotard, J.-M., 1984. Etude pétrographique et géochimique de roches draguées dans l'Archipel des Marquises, Polynésie Française (Océan Pacifique Sud): existence d'un volcanisme basanitique dans le nord de cet archipel, *Comptes-rendus des séances de l'Académie des sciences. Série 2, Mécanique-physique, chimie, sciences de l'univers, sciences de la terre*, **299**(2), 61–64.

[81] Bassin, C., Laske, G., & Masters, G., 2000. The current limits of resolution for surface wave tomography in North America, *EOS Trans AGU*, **81**(F897).

[82] Basu, A. R., Wang Junwen, Huang Wankang, Xie Guanghong, & Tatsumoto, M., 1991. Major element, REE, and Pb, Nd and Sr isotopic geochemistry of Cenozoic volcanic rocks of eastern China: implications for their origin from suboceanic-type mantle reservoirs, *Earth and Planetary Science Letters*, **105**(1-3), 149–169.

[83] Batiza, R., 1977. Petrology and chemistry of Guadalupe Island: An alkalic seamount on a fossil ridge crest, *Geology*, **5**(12), 760–764.

[84] Baubron, J. C. & Maury, R. C., 1980. Age and petrology of the Jabal Abyad volcanic chain, Khaybar plateau, Saudi Arabia, *Geodynamic evolution of the Afro-Arabian rift system*, pp. 655–666.

[85] Baxter, A. N., Upton, B. G., & White, W. M., 1985. Petrology and geochemistry of Rodrigues Island, Indian Ocean, *Contributions to Mineralogy and Petrology*, **89**(1), 90–101.

[86] Beccaluva, L., Siena, F., Coltorti, M., Di Grande, A., Lo Giudice, A., Macciotta, G., Tassinari, R., & Vaccaro, C., 1998. Nephelinitic to tholeiitic magma generation in a transtensional tectonic setting: an integrated model for the Iblean volcanism, Sicily, *Journal of Petrology*, **39**(9), 1547–1576.

[87] Beccaluva, L., Bianchini, G., Bonadiman, C., Siena, F., & Vaccaro, C., 2004. Coexisting anorogenic and subduction-related metasomatism in mantle xenoliths from the Betic Cordillera (southern Spain), *Lithos*, **75**(1-2), 67–87.

[88] Beccaluva, L., Bianchini, G., Ellam, R. M., Marzola, M., Oun, K. M., Siena, F., & Stuart, F. M., 2008. The role of HIMU metasomatic components in the North African lithospheric mantle: petrological evidence from the Gharyan lherzolite xenoliths, NW Libya, *Geological Society, London, Special Publications*, **293**(1), 253–277.

[89] Beccaluva, L., Bianchini, G., Natali, C., & Siena, F., 2009. Continental flood basalts and mantle plumes: a case study of the Northern Ethiopian Plateau, *Journal of Petrology*, **50**(7), 1377–1403.

[90] Beier, C., Haase, K. M., & Hansteen, T. H., 2006. Magma evolution of the Sete Cidades volcano, São Miguel, Azores, *Journal of Petrology*, **47**(7), 1375–1411.

[91] Beier, C., Stracke, A., & Haase, K. M., 2007. The peculiar geochemical signatures of São Miguel (Azores) lavas: Metasomatised or recycled mantle sources?, *Earth and Planetary Science Letters*, **259**(1-2), 186–199.

[92] Beier, C., Haase, K. M., Abouchami, W., Krienitz, M.-S., & Hauff, F., 2008. Magma genesis by rifting of oceanic lithosphere above anomalous mantle: Terceira Rift, Azores, *Geochemistry, Geophysics, Geosystems*, **9**(12), 1–26.

[93] Beier, C., Haase, K. M., & Turner, S. P., 2012. Conditions of melting beneath the Azores, *Lithos*, **144**, 1–11.

[94] Bellieni, G., Brotzu, P., Morbidelli, L., Piccirillo, E., & Traversa, G., 1986. Petrology and mineralogy of Miocene fissural volcanism of the East Kenya Plateau, *Neues Jahrb. Mineral. Abh*, **154**, 153–178.

[95] Bellon, H. & Pouclet, A., 1980. Datations K-Ar de quelques laves du Rift-Ouest de l'Afrique Centrale; implications sur l'évolution magmatique et structurale, *Geologische Rundschau*, **69**(1), 49–62.

[96] Berger, J., Ennih, N., Mercier, J.-C. C., LiéGeois, J.-P., & Demaiffe, D., 2009. The role of fractional crystallization and late-stage peralkaline melt segregation in the mineralogical evolution of Cenozoic nephelinites/phonolites from Saghro (SE Morocco), *Mineralogical Magazine*, **73**(1), 59–82.

[97] Berger, J., Ennih, N., & Liégeois, J. P., 2014. Extreme trace elements fractionation in Cenozoic nephelinites and phonolites from the Moroccan Anti-Atlas (Eastern Saghro), *Lithos*, **210**, 69–88.

[98] Bergmanis, E. C., Sinton, J. M., & Trusdell, F. A., 2000. Rejuvenated volcanism along the southwest rift zone, East Maui, Hawai 'i, *Bulletin of Volcanology*, **62**(4-5), 239–255.

[99] Berhe, S. M., Desta, B., Nicoletti, M., & Teferra, M., 1987. Geology, geochronology and geodynamic implications of the Cenozoic magmatic province in W and SE Ethiopia, *Journal of the Geological Society*, **144**(2), 213–226.

[100] Berrahma, M., Delaloye, M., Faure-Muret, A., & Rachdi, H. E. N., 1993. Premières données géochronologiques sur le volcanisme alcalin du Jbel Saghro, Anti-Atlas, Maroc, *Journal of African Earth Sciences*, **17**(3), 333–341.

[101] Bertrand, H., Chazot, G., Blichert-Toft, J., & Thoral, S., 2003. Implications of widespread high-μ volcanism on the Arabian Plate for Afar mantle plume and lithosphere composition, *Chemical Geology*, **198**(1-2), 47–61.

[102] Bevier, M., 1981. The Rainbow Range, British Columbia: a Miocene peralkaline shield volcano, *Journal of Volcanology and Geothermal Research*, **11**, 225–251.

[103] Biabangard, H. & Moradian, A., 2008. Geology and geochemical evaluation of Taftan Volcano, Sistan and Baluchestan Province, southeast of Iran, *Chinese Journal of Geochemistry*, **27**(4), 356–369.

[104] Bianchini, G., Clocchiatti, R., Coltorti, M., Joron, J. L., Vaccaro, C.,*et al.*, 1998. Petrogenesis of mafic lavas from the northernmost sector of the Iblean district (Sicily), *European Journal of Mineralogy-Ohne Beihefte*, **10**(2), 301–316.

[105] Binard, N., Hekinian, R., Cheminee, J., & Stoffers, P., 1992. Styles of eruptive activity on intraplate volcanoes in the Society and Austral hot spot regions: Bathymetry, petrology, and submersible observations, *Journal of Geophysical Research: Solid Earth*, **97**(B10), 13999–14015.

[106] Binard, N., Maury, R. C., Guille, G., Talandier, J., Gillot, P. Y., & Cotten, J., 1993. Mehetia Island, South Pacific: geology and petrology of the emerged part of the Society hot spot, *Journal of Volcanology and Geothermal Research*, **55**(3-4), 239–260.

[107] Bindeman, I., Gurenko, A., Sigmarsson, O., & Chaussidon, M., 2008. Oxygen isotope heterogeneity and disequilibria of olivine crystals in large volume Holocene basalts from Iceland: Evidence for magmatic digestion and erosion of Pleistocene hyaloclastites, *Geochimica et Cosmochimica Acta*, **72**(17), 4397–4420.

[108] Bindi, L., Tasselli, F., Olmi, F., Peccerillo, A., & Menchetti, S., 2002. Crystal chemistry of clinopyroxenes from Linosa Volcano, Sicily Channel, Italy: implications for modelling the magmatic plumbing system, *Mineralogical Magazine*, **66**(6), 953–968.

[109] Bizouard, H., Barberi, F., & Varet, J., 1980. Mineralogy and petrology of Erta'Ale and Boina volcanic series, Afar rift, Ethiopia, *Journal of Petrology*, **21**(2), 401–436.

[110] Black, S., Macdonald, R., Barreiro, B. A., Dunkley, P. N., & Smith, M., 1998. Open system alkaline magmatism in northern Kenya: Evidence from U-series disequilibria and radiogenic isotopes, *Contributions to Mineralogy and Petrology*, **131**(4), 364–378.

[111] Blais, S., Miau, D., Guille, G., Maury, R., Cotten, J., & Guillou, H., 1997. Geology and petrology of Raiatea Island (Society Islands, French Polynesia), *Comptes Rendus de l'Academie des Sciences. Serie 2, Sciences de la Terre et des Planetes*, pp. 435–442.

[112] Blais, S., Guille, G., Guillou, H., Chauvel, C., Maury, R. C., & Caroff, M., 2000. Géologie, géochimie et géochronologie de l'île de Bora Bora (Société, Polynésie française), *Comptes Rendus de l'Académie des Sciences - Series IIA - Earth and Planetary Science*, **331**(9), 579–585.

[113] Blais, S., Guille, G., Guillou, H., Chauvel, C., Maury, R. C., Pernet, G., & Cotten, J., 2002. The island of Maupiti : the oldest emergent volcano in the Society hot spot chain (French Polynesia), *Bulletin de la Societe Geologique de France*, **173**(1), 45–55.

[114] Bloomer, S. H., Curtis, P. C., & Karson, J. A., 1989. Geochemical variation of Quaternary basaltic volcanics in the Turkana Rift, northern Kenya, *Journal of African Earth Sciences (and the Middle East)*, **8**(2-4), 511–532.

[115] Boccaletti, M., Getaneh, A., Mazzuoli, R., Tortorici, L., & Trua, T., 1995. Chemical variations in a bimodal magma system: the Plio-Quaternary volcanism in the Dera Nazret area (Main Ethiopian Rift, Ethiopia), *Africa Geoscience Review*, **2**(1), 37–60.

[116] Boggard, P. J. F. & Wörner, G., 2003. Petrogenesis of Basanitic to Tholeiitic Volcanic Rocks from the Miocene Vogelsberg, Central Germany, *Journal of Petrology*, **44**(3), 569–602.

[117] Bohrson, W. A. & Clague, D. A., 1988. Origin of ultramafic xenoliths containing exsolved pyroxenes from Hualalai Volcano, Hawaii, *Contributions to Mineralogy and Petrology*, **100**(2), 139–155.

[118] Boivin, P. & Bachèlery, P., 2009. Petrology of 1977 to 1998 eruptions of Piton de la Fournaise, La Réunion Island, *Journal of Volcanology and Geothermal Research*, **184**(1-2), 109–125.

[119] Bonatti, E., Harrison, C., Fisher, D., Honnorez, J., Schilling, J.-G., Stipp, J., & Zentilli, M., 1977. Easter volcanic chain (southeast Pacific): a mantle hot line, *Journal of Geophysical Research*, **82**(17), 2457–2478.

[120] Bongiolo, E. M., Pires, G. L., Geraldes, M. C., Santos, A. C., & Neumann, R., 2015. Geochemical modeling and Nd-Sr data links nephelinite-phonolite successions and xenoliths of Trindade Island (South Atlantic Ocean, Brazil), *Journal of Volcanology and Geothermal Research*, **306**, 58–73.

[121] Bonneville, A., Dosso, L., & Hildenbrand, A., 2006. Temporal evolution and geochemical variability of the South Pacific superplume activity, *Earth and Planetary Science Letters*, **244**(1-2), 251–269.

[122] Bosch, D., Maury, R. C., El Azzouzi, M., Bollinger, C., Bellon, H., & Verdoux, P., 2014. Lithospheric origin for Neogene-Quaternary Middle Atlas lavas (Morocco): Clues from trace elements and Sr-Nd-Pb-Hf isotopes, *Lithos*, **205**, 247–265.

[123] Bow, C. S. & Geist, D. J., 1992. Geology and petrology of Floreana Island, Galapagos Archipelago, Ecuador, *Journal of Volcanology and Geothermal Research*, **52**(1-3), 83–105.

[124] Boyce, J. A., Nicholls, I. A., Keays, R. R., & Hayman, P. C., 2015. Variation in parental magmas of Mt Rouse, a complex polymagmatic monogenetic volcano in the basaltic intraplate Newer Volcanics Province, southeast Australia, *Contributions to Mineralogy and Petrology*, **169**(2), 11.

[125] Brandon, A. D., Graham, D. W., Waight, T., & Gautason, B., 2007. ^{186}Os and ^{187}Os enrichments and high-$^{3}He/^{4}He$ sources in the Earth's mantle: Evidence from Icelandic picrites, *Geochimica et Cosmochimica Acta*, **71**(18), 4570–4591.

[126] Breddam, K., 2002. Kistufell: Primitive Melt from the Iceland Mantle Plume, *Journal of Petrology*, **43**(2), 345–373.

[127] Brenna, M., Cronin, S. J., Smith, I. E., Sohn, Y. K., & Németh, K., 2010. Mechanisms driving polymagmatic activity at a monogenetic volcano, Udo, Jeju Island, South Korea, *Contributions to Mineralogy and Petrology*, **160**(6), 931–950.

[128] Brenna, M., Cronin, S. J., Smith, I. E., Maas, R., & Sohn, Y. K., 2012. How small-volume basaltic magmatic systems develop: A case study from the jeju island volcanic field, Korea, *Journal of Petrology*, **53**(5), 985–1018.

[129] Brenna, M., Cronin, S. J., Smith, I. E., Sohn, Y. K., & Maas, R., 2012. Spatio-temporal evolution of a dispersed magmatic system and its implications for volcano growth, Jeju Island Volcanic Field, Korea, *Lithos*, **148**, 337–352.

[130] Brenna, M., Price, R., Cronin, S. J., Smith, I. E., Sohn, Y. K., Bom Kim, G., & Maas, R., 2014. Final magma storage depth modulation of explosivity andtrachyte-phonolite genesis at an intraplate volcano: A case study from Ulleung Island, South Korea, *Journal of Petrology*, **55**(4), 709–747.

[131] Breton, T., Nauret, F., Pichat, S., Moine, B., Moreira, M., Rose-Koga, E. F., Auclair, D., Bosq, C., & Wavrant, L. M., 2013. Geochemical heterogeneities within the Crozet hotspot, *Earth and Planetary Science Letters*, **376**, 126–136.

[132] Briot, D., Cantagrel, J. M., Dupuy, C., & Harmon, R. S., 1991. Geochemical evolution in crustal magma reservoirs: Trace-element and SrNdO isotopic variations in two continental intraplate series at Monts Dore, Massif Central, France, *Chemical Geology*, **89**(3-4), 281–303.

[133] Brotzu, P., Kazmin, V., Morbidelli, L., Piccirillo, E., Seife, M. B., & Traversa, G., 1980. Petrochemistry of the volcanics in the northern part of the main Ethiopian Rift, *Geodynamic Evolution of the Afro-Arabic Rift System. Atti Cony. Acc. Lincei*, **47**, 367–386.

[134] Brotzu, P., Ganzerli-Valentini, M. T., Morbidelli, L., Piccirillo, E. M., Stella, R., & Traversa, G., 1981. Basaltic volcanism in the northern sector of the main Ethiopian rift, *Journal of Volcanology and Geothermal Research*, **10**(4), 365–382.

[135] Brotzu, P., Morbidelli, L., Piccirillo, E., & Traversa, G., 1983. The basanite to peralkaline phonolite suite of the Plio-Quaternary Nyambeni multicentre volcanic range (East Kenya Plateau), *N Jahrb Mineral Abh*, **147**, 253–280.

[136] Brown, G. E., Schmidt, D. L., & Huffman Jr, A. C., 1989. *Geology of the Arabian Peninsula; shield area of western Saudi Arabia. U.S – Geological Survey Professional Paper No. 560-A*, United States Government Printing Office, Washington, U.S.A.

[137] Bruni, S., D'Orazio, M., Haller, M. J., Innocenti, F., Manetti, P., Pécskay, Z., & Tonarini, S., 2008. Time-evolution of magma sources in a continental back-arc setting: The Cenozoic basalts from Sierra de San Bernardo (Patagonia, Chubut, Argentina), *Geological Magazine*, **145**(5), 714–732.

[138] Bryan, S. E., Martí, J., & Leosson, M., 2002. Petrology and geochemistry of the Bandas des Sur Formation, Las Cañadas Edifice, Tenerife (Canary Islands), *Journal of Petrology*, **43**(10), 1815–1856.

[139] Bybee, G., Ashwal, L., & Wilson, A., 2010. New evidence for a volcanic arc on the western margin of a rifting Rodinia from ultramafic intrusions in the Andriamena region, north-central Madagascar, *Earth and Planetary Science Letters*, **293**(1-2), 42–53.

[140] Cajz, V., Rapprich, V., Erban, V., Pécskay, Z., & Radoň, M., 2009. Late Miocene volcanic activity in the České středohoří Mountains (Ohře/Eger Graben, northern Bohemia), *Geologica Carpathica*, **60**(6), 519–533.

[141] Calanchi, N., Colantoni, P., Rossi, P. L., Saitta, M., & Serri, G., 1989. The Strait of Sicily continental rift systems: Physiography and petrochemistry of the submarine volcanic centres, *Marine Geology*, **87**(1), 55–83.

[142] Calmus, T., Aguillón-Robles, A., Maury, R. C., Bellon, H., Benoit, M., Cotten, J., Bourgois, J., & Michaud, F., 2003. Spatial and temporal evolution of basalts and magnesian andesites (" bajaites") from Baja California, Mexico: The role of slab melts, *Lithos*, **66**(1-2), 77–105.

[143] Camp, V. E. & Roobol, M. J., 1989. The Arabian continental alkali basalt province: Part I. Evolution of Harrat Rahat, Kingdom of Saudi Arabia, *Geological Society of America Bulletin*, **101**(1), 71–95.

[144] Camp, V. E., Roobol, M. J., & Hooper, P. R., 1991. The Arabian continental alkali basalt province : Part II . Evolution of Harrats Khaybar, Ithnayn, and Kura, Kingdom of Saudi Arabia, *Geological Society of America Bulletin*, **103**(March), 363–391.

[145] Camp, V. E., Roobol, M. J., & Hooper, P. R., 1992. The Arabian continental alkali basalt province: Part III. Evolution of Harrat Kishb, Kingdom of Saudi Arabia, *Geological Society of America Bulletin*, **104**(4), 379–396.

[146] Campeny, M., Melgarejo, J. C., Mangas, J., Manuel, J., & Gonçalves, A. O., 2017. Recent carbonatitic magmatism in Angola: The dykes of the Chiva lagoon maar, *Boletin de la Sociedad Geologica Mexicana*, **69**(1), 209–222.

[147] Capaldi, G., Chiesa, S., Manetti, P., Orsi, G., & Poli, G., 1987. Tertiary anorogenic granites of the western border of the Yemen Plateau, *Lithos*, **20**(6), 433–444.

[148] Capedri, S., Venturelli, G., Salvioli-Mariani, E., Crawford, A. J., & Barbieri, M., 1989. Upper-mantle xenoliths and megacrysts in an alkali basalt from Tallante, south-eastern Spain, *European Journal of Mineralogy*, pp. 685–700.

[149] Caroff, M., Maury, R. C., Leterrier, J., Joron, J. L., Cotten, J., & Guille, G., 1993. Trace element behavior in the alkali basalt-comenditic trachyte series from Mururoa Atoll, French Polynesia, *Lithos*, **30**(1), 1–22.

[150] Caroff, M., Maury, R. C., Vidal, P., Guille, G., Dupuy, C., Cotten, J., Guillou, H., & Gillot, P. Y., 1995. Rapid temporal changes in ocean island basalt composition: Evidence from an 800 m deep drill hole in Eiao Shield (Marquesas), *Journal of Petrology*, **36**(5), 1333–1365.

[151] Caroff, M., Maury, R. C., Guille, G., & Cotten, J., 1997. Partial melting below Tubuai (Austral Islands, French Polynesia), *Contributions to Mineralogy and Petrology*, **127**(4), 369–382.

[152] Caroff, M., Guillou, H., Lamiaux, M., Maury, R. C., Guille, G., & Cotten, J., 1999. Assimilation of ocean crust by hawaiitic and mugearitic magmas: An example from Eiao (Marquesas), *Lithos*, **46**(2), 235–258.

[153] Carracedo, J. C., Rodríguez Badiola, E., & Soler, V., 1990. Aspectos volcanologicos y estructurales, evolucion petrologica e implicaciones den riesgo volcanico de la erupcion de 1730 en Lanzarote, Islas Canarias, *Estudios Geológicos*, **46**(1-2).

[154] Carracedo, J. C., Badiola, E. R., Guillou, H., De la Nuez, J., & Pérez Torrado, F. J., 2001. Geología y vulcanología de La Palma y El Hierro, Canarias Occidentales, *Estudios Geológicos*, **57**(5-6).

[155] Carrasco-Núñez, G., Righter, K., Chesley, J., Siebert, L., & Aranda-Gómez, J. J., 2005. Contemporaneous eruption of calc-alkaline and alkaline lavas in a continental arc (Eastern Mexican Volcanic Belt): Chemically heterogeneous but isotopically homogeneous source, *Contributions to Mineralogy and Petrology*, **150**(4), 423–440.

[156] Casadevall, T. & Dzurisin, D., 1987. Stratigraphy and petrology of the Uwekahuna Bluff section, Kilauea caldera, *Volcanism in Hawaii*, **1**, 351–375.

[157] Castillo, P. R., Hilton, D. R., & Halldórsson, S. A., 2014. Trace element and Sr-Nd-Pb isotope geochemistry of Rungwe Volcanic Province, Tanzania: implications for a Superplume source for East Africa Rift magmatism, *Frontiers in Earth Science*, **2**, 21.

[158] Cebriá, J. M. & López-Ruiz, J., 1996. A refined method for trace element modelling of nonmodal batch partial melting processes: The Cenozoic continental volcanism of Calatrava, central Spain, *Geochimica et Cosmochimica Acta*, **60**(8), 1355–1366.

[159] Cebriá, J. M., López-Ruiz, J., Doblas, M., Oyarzun, R., Hertogen, J., & Benito, R., 2000. Geochemistry of the Quaternary alkali basalts of Garrotxa (NE Volcanic Province, Spain): a case of double enrichment of the mantle lithosphere, *Journal of Volcanology and Geothermal Research*, **102**, 217–235.

[160] Cebriá, J. M., López-Ruiz, J., Carmona, J., & Doblas, M., 2009. Quantitative petrogenetic constraints on the Pliocene alkali basaltic volcanism of the SE Spain Volcanic Province, *Journal of Volcanology and Geothermal Research*, **185**(3), 172–180.

[161] Chadwick, J., Keller, R., Kamenov, G., Yogodzinski, G., & Lupton, J., 2014. The Cobb hot spot: HIMU-DMM mixing and melting controlled by a progressively thinning lithospheric lid, *Geochemistry, Geophysics, Geosystems*, **15**(8), 3107–3122.

[162] Chaffey, D. J., 1988. *Characterisation of ocean island basalt sources: St, Helena*, Ph.D. thesis, University of Leeds.

[163] Chakrabarti, R., Basu, A. R., Santo, A. P., Tedesco, D., & Vaselli, O., 2009. Isotopic and geochemical evidence for a heterogeneous mantle plume origin of the Virunga volcanics, Western rift, East African Rift system, *Chemical Geology*, **259**(3-4), 273–289.

[164] Charreteur, G. & Tegner, C., 2013. Magmatic emulsion texture formed by mixing during extrusion, Rauethafell composite complex, Breiethdalur volcano, eastern Iceland, *Bulletin of Volcanology*, **75**(6), 1–17.

[165] Chauvel, C. & Jahn, B.-M., 1984. Nd/Sr isotope and REE geochemistry of alkali basalts from the Massif Central, France, *Geochimica et Cosmochimica Acta*, **48**(1), 93–110.

[166] Chauvel, C., McDonough, W., Guille, G., Maury, R., & Duncan, R., 1997. Contrasting old and young volcanism in Rurutu Island, Austral chain, *Chemical Geology*, **139**(1-4), 125–143.

[167] Chauvel, C., Maury, R. C., Blais, S., Lewin, E., Guillou, H., Guille, G., Rossi, P., & Gutscher, M. A., 2012. The size of plume heterogeneities constrained by Marquesas isotopic stripes, *Geochemistry, Geophysics, Geosystems*, **13**(1).

[168] Chazot, G. & Bertrand, H., 1993. Mantle sources and magma-continental crust interactions during early Red Sea-Gulf of Aden rifting in southern Yemen: Elemental and Sr, Nd, Pb isotope evidence, *Journal of Geophysical Research: Solid Earth*, **98**(B2), 1819–1835.

[169] Cheminee, J. L., Hekinian, R., Talandier, J., Albarede, F., Devey, C. W., Francheteau, J., & Lancelot, Y., 1989. Geology of an active hot spot: Teahitia-Mehetia region in the South Central Pacific, *Marine Geophysical Researches*, **11**(1), 27–50.

[170] Chen, C., Frey, F., Rhodes, J., & Easton, R., 1996. Temporal geochemical evolution of Kilauea Volcano: comparison of Hilina and Puna Basalt, *Geophysical Monograph – American Geophysical Union*, **95**, 161–182.

[171] Chen, C.-H., Chung, S.-H., Hwang, H.-H., Chen, C.-H., & Chung, S.-L., 2001. Petrology and geochemistry of Neogene continental basalts and related rocks in Northern Taiwan (III): alkali basalts and tholiites from Shiting-Yinko area, *Western Pacific Earth Sciences*, **1**(1), 19–46.

[172] Chen, C. Y., 1993. High-magnesium primary magmas from Haleakala Volcano, east Maui, Hawaii: petrography, nickel, and major-element constraints, *Journal of Volcanology and Geothermal Research*, **55**(1-2), 143–153.

[173] Chen, C. Y. & Frey, F. A., 1983. Origin of Hawaiian tholeiite and alkalic basalt, *Nature*, **302**(5911), 785–789.

[174] Chen, C. Y., Frey, F. A., & Garcia, M. O., 1990. Evolution of alkalic lavas at Haleakala Volcano, east Maui, Hawaii, *Contributions to Mineralogy and Petrology*, **105**(2), 197–218.

[175] Chen, C. Y., Frey, F. A., Garcia, M. O., Dalrymple, G. B., & Hart, S. R., 1991. The tholeiite to alkalic basalt transition at Haleakala Volcano, Maui, Hawaii, *Contributions to Mineralogy and Petrology*, **106**(2), 183–200.

[176] Chen, H., Xia, Q. K., Ingrin, J., Deloule, E., & Bi, Y., 2017. Heterogeneous source components of intraplate basalts from NE China induced by the ongoing Pacific slab subduction, *Earth and Planetary Science Letters*, **459**, 208–220.

[177] Chen, J.-L., Xu, J.-F., Wang, B.-D., & Kang, Z.-Q., 2012. Cenozoic Mg-rich potassic rocks in the Tibetan Plateau: Geochemical variations, heterogeneity of subcontinental lithospheric mantle and tectonic implications, *Journal of Asian Earth Sciences*, **53**, 115–130.

[178] Chen, Y., Zhang, Y., Graham, D., Su, S., & Deng, J., 2007. Geochemistry of Cenozoic basalts and mantle xenoliths in Northeast China, *Lithos*, **96**(1-2), 108–126.

[179] Cheng, Q. C., Macdougall, J. D., & Lugmair, G. W., 1993. Geochemical studies of Tahiti, Teahitia and Mehetia, Society Island chain, *Journal of Volcanology and Geothermal Research*, **55**(1-2), 155–184.

[180] Cheng, Z. & Guo, Z., 2017. Post-collisional ultrapotassic rocks and mantle xenoliths in the Sailipu volcanic field of Lhasa terrane, south Tibet: Petrological and geochemical constraints on mantle source and geodynamic setting, *Gondwana Research*, **46**, 17–42.

[181] Cheng, Z., Guo, Z., Dingwell, D. B., Li, X., Zhang, M., Liu, J., Zhao, W., & Lei, M., 2020. Geochemistry and petrogenesis of the post-collisional high-K calc-alkaline magmatic rocks in Tengchong, SE Tibet, *Journal of Asian Earth Sciences*, p. 104309.

[182] Chernet, T. & Hart, W., 1999. Petrology and geochemistry of volcanism in the northern Main Ethiopian Rift-southern Afar transition region, *Acta Vulcanologica*, **11**, 21–42.

[183] Chernet, T., Hart, W. K., Aronson, J. L., & Walter, R. C., 1998. New age constraints on the timing of volcanism and tectonism in the northern Main Ethiopian Rift–southern Afar transition zone (Ethiopia), *Journal of Volcanology and Geothermal Research*, **80**(3-4), 267–280.

[184] Chiesa, S., Civetta, L., De Fino, M., La Volpe, L., & Orsi, G., 1989. The Yemen trap series: genesis and evolution of a continental flood basalt province, *Journal of Volcanology and Geothermal Research*, **36**(4), 337–350.

[185] Choi, S. H., Mukasa, S. B., Kwon, S. T., & Andronikov, A. V., 2006. Sr, Nd, Pb and Hf isotopic compositions of late Cenozoic alkali basalts in South Korea: Evidence for mixing between the two dominant asthenospheric mantle domains beneath East Asia, *Chemical Geology*, **232**(3-4), 134–151.

[186] Choo, M. K., Lee, M. J., Lee, J. I., Kim, K. H., & Park, K.-H., 2012. Geochemistry and Sr-Nd-Pb isotopic constraints on the petrogenesis of Cenozoic lavas from the Pali Aike and Morro Chico area (52°S), southern Patagonia, South America, *Island Arc*, **21**(4), 327–350.

[187] Chu, Z., Yan, Y., Zeng, G., Tian, W., Li, C., Yang, Y., & Guo, J., 2017. Petrogenesis of Cenozoic basalts in central-eastern China: Constraints from Re–Os and PGE geochemistry, *Lithos*, **278-281**, 72–83.

[188] Chu, Z. Y., Harvey, J., Liu, C. Z., Guo, J. H., Wu, F. Y., Tian, W., Zhang, Y. L., & Yang, Y. H., 2013. Source of highly potassic basalts in northeast China: Evidence from Re-Os, Sr-Nd-Hf isotopes and PGE geochemistry, *Chemical Geology*, **357**, 52–66.

[189] Chung, S., 1999. Trace Element and Isotope Characteristics of Cenozoic Basalts around the Tanlu Fault with Implications for the Eastern Plate Boundary between North and South China, *The Journal of Geology*, **108**(6), 301–311.

[190] Chung, S. L., su Sun, S., Tu, K., Chen, C. H., & yu Lee, C., 1994. Late Cenozoic basaltic volcanism around the Taiwan Strait, SE China: Product of lithosphere-asthenosphere interaction during continental extension, *Chemical Geology*, **112**(1-2), 1–20.

[191] Chung, S. L., Jahn, B. M., Chen, S. J., Lee, T., & Chen, C. H., 1995. Miocene basalts in northwestern Taiwan: Evidence for EM-type mantle sources in the continental lithosphere, *Geochimica et Cosmochimica Acta*, **59**(3), 549–555.

[192] Chuvashova, I., Rasskazov, S., & Yasnygina, T., 2017. Mid-Miocene thermal impact on the lithosphere by sub-lithospheric convective mantle material: Transition from high- to moderate-Mg magmatism beneath Vitim Plateau, Siberia, *Geoscience Frontiers*, **8**(4), 753–774.

[193] Chuvashova, I. S., Rasskazov, S. V., Yasnygina, T. A., Saranina, E. V., & Fefelov, N. N., 2007. Holocene volcanism in central Mongolia and Northeast China: Asynchronous decompressional and fluid melting of the mantle, *Journal of Volcanology and Seismology*, **1**(6), 372–396.

[194] Cinque, A., Civetta, L., Orsi, G., & Peccerillo, A., 1988. Geology and geochemistry of the island of Ustica (Southern Tyrrhenian Sea), *Boll. Soc. Ital. Miner. Petrol*, **43**, 987–1002.

[195] Civetta, L., Cornette, Y., Crisci, G., Gillot, P. Y., Orsi, G., & Requejo, C. S., 1984. Geology, geochronology and chemical evolution of the island of Pantelleria, *Geological Magazine*, **121**(6), 541–668.

[196] Clague, D. A., 1988. Petrology of ultramafic xenoliths from loihi seamount, Hawaii, *Journal of Petrology*, **29**(6), 1161–1186.

[197] Clague, D. A. & Beeson, M. H., 1980. Trace element geochemistry of the East Molokai volcanic series, Hawaii, *American Journal of Science*, **280**(A), 820–844.

[198] Clague, D. A. & Frey, F. A., 1982. Petrology and trace element geochemistry of the Honolulu volcanics, Oahu: Implications for the oceanic mantle below Hawaii, *Journal of Petrology*, **23**(3), 447–504.

[199] Clague, D. A., Jackson, E. D., & Wright, T. L., 1980. Petrology of Hualalai volcano, Hawaii: Implication for mantle composition, *Bulletin Volcanologique*, **43**(4), 641–656.

[200] Clague, D. A., Moore, J. G., Dixon, J. E., & Friesen, W. B., 1995. Petrology of submarine lavas from Kilauea's Puna ridge, Hawaii, *Journal of Petrology*, **36**(2), 299–349.

[201] Clark, J. G. & Dymond, J., 1977. Geochronology and petrochemistry of Easter and Sala y Gomez Islands: Implications for the origin of the Sala y Gomez Ridge, *Journal of Volcanology and Geothermal Research*, **2**(1), 29–48.

[202] Class, C. & Goldstein, S. L., 1997. Plume-lithosphere interactions in the ocean basins: constraints from the source mineralogy, *Earth and Planetary Science Letters*, **150**, 245–260.

[203] Class, C., Altherr, R., Volker, F., Eberz, G., & McCulloch, M. T., 1994. Geochemistry of Pliocene to Quaternary alkali basalts from the Huri Hills, northern Kenya, *Chemical Geology*, **113**(1-2), 1–22.

[204] Class, C., Goldstein, S. L., Altherr, R., & Bachèlery, P., 1998. The process of plume-lithosphere interactions in the ocean basins – the case of Grande Comore, *Journal of Petrology*, **39**(5), 881–903.

[205] Class, C., Goldstein, S. L., Stute, M., Kurz, M. D., & Schlosser, P., 2005. Grand Comore Island: A well-constrained "low $^{3}He/^{4}He$" mantle plume, *Earth and Planetary Science Letters*, **233**(3-4), 391–409.

[206] Claude-Ivanaj, C., Bourdon, B., & Allègre, C. J., 1998. Ra-Th-Sr isotope systematics in Grande Comore Island: A case study of plume-lithosphere interaction, *Earth and Planetary Science Letters*, **164**(1-2), 99–117.

[207] Claude-Ivanaj, C., Joron, J.-L., & Allègre, C. J., 2001. ^{238}U–^{230}Th–^{226}Ra fractionation in historical lavas from the Azores: long-lived source heterogeneity vs. metasomatism fingerprints, *Chemical Geology*, **176**(1-4), 295–310.

[208] Clément, J.-P., Caroff, M., Hémond, C., Tiercelin, J.-J., Bollinger, C., Guillou, H., & Cotten, J., 2003. Pleistocene magmatism in a lithospheric transition area: petrogenesis of alkaline and peralkaline lavas from the Baringo-Bogoria Basin, central Kenya Rift, *Canadian Journal of Earth Sciences*, **40**(9), 1239–1257.

[209] Cliff, R. A., Baker, P. E., & Mateer, N. J., 1991. Geochemistry of inaccessible island volcanics, *Chemical Geology*, **92**(4), 251–260.

[210] Coish, R. A., Kretschmar, L. M., & Journeay, J. M., 1998. Geochemistry of the Miocene Mount Noel Volcanic Complex, British Columbia and comparison with the Columbia River basalt, *Journal of Volcanology and Geothermal Research*, **83**(3-4), 269–285.

[211] Coleman, R. G., Gregory, R. T., & Brown, G. F., 1983. Cenozoic volcanic rocks of Saudi Arabia: evidence for a passive origin for the Red Sea Rift, Tech. rep., Saudi Arabian Deputy Ministery of Mineral Reseources.

[212] Condomines, M., Morand, P., Camus, G., & Duthou, L., 1982. Chronological and geochemical study of lavas from the Chaine des Puys, Massif Central, France: evidence for crustal contamination, *Contributions to Mineralogy and Petrology*, **81**(4), 296–303.

[213] Condomines, M., Carpentier, M., & Ongendangenda, T., 2015. Extreme radium deficit in the 1957 AD Mugogo lava (Virunga volcanic field, Africa): Its bearing on olivine-melilitite genesis, *Contributions to Mineralogy and Petrology*, **169**(3), 29.

[214] Conticelli, S., Sintoni, M. F., Abebe, T., Mazzarini, F., & Manetti, P., 1999. Petrology and geochemistry of ultramafic xenoliths and host lavas from the Ethiopian volcanic province: an insight into the upper mantle under Eastern Africa, *ACta Vulcanologica*, **11**(1), 143–159.

[215] Conticelli, S., Guarnieri, L., Farinelli, A., Mattei, M., Avanzinelli, R., Bianchini, G., Boari, E., Tommasini, S., Tiepolo, M., Prelević, D.,*et al.*, 2009. Trace elements and Sr–Nd–Pb isotopes of K-rich, shoshonitic, and calc-alkaline magmatism of the Western Mediterranean Region: genesis of ultrapotassic to calc-alkaline magmatic associations in a post-collisional geodynamic setting, *Lithos*, **107**(1-2), 68–92.

[216] Cook, C., Briggs, R. M., Smith, I. E., & Maas, R., 2005. Petrology and geochemistry of intraplate basalts in the South Auckland Volcanic Field, New Zealand: Evidence for two coeval magma suites from distinct sources, *Journal of Petrology*, **46**(3), 473–503.

[217] Cooper, A. F., Adam, L. J., Coulter, R. F., Eby, G. N., & McIntosh, W. C., 2007. Geology, geochronology and geochemistry of a basanitic volcano, White Island, Ross Sea, Antarctica, *Journal of Volcanology and Geothermal Research*, **165**(3-4), 189–216.

[218] Cooper, K. M., Reid, M. R., Dunbar, N., & McIntosh, W., 2002. Origin of mafic magmas beneath northwestern Tibet: Constraints from ^{230}Th-^{238}U disequilibria, *Geochemistry, Geophysics, Geosystems*, **3**(11), 1–23.

[219] Corsaro, R. A. & Cristofolini, R., 1996. Origin and differentiation of recent basaltic magmas from Mount Etna, *Mineralogy and Petrology*, **57**, 1–21.

[220] Cotten, J., Le Dez, A., Bau, M., Caroff, M., Maury, R. C., Dulski, P., Fourcade, S., Bohn, M., & Brousse, R., 1995. Origin of anomalous rare-earth element and yttrium enrichments in subaerially exposed basalts: evidence from French Polynesia, *Chemical Geology*, **119**, 115–138.

[221] Coulié, E., Quidelleur, X., Gillot, P.-Y., Courtillot, V., Lefèvre, J.-C., & Chiesa, S., 2003. Comparative K–Ar and Ar–Ar dating of Ethiopian and Yemenite Oligocene volcanism: implications for timing and duration of the Ethiopian traps, *Earth and Planetary Science Letters*, **206**(3-4), 477–492.

[222] Cousens, B., Dostal, J., & Hamilton, T., 1999. A near-ridge origin for seamounts at the southern terminus of the Pratt-Welker Seamount Chain, northeast Pacific Ocean, *Canadian Journal of Earth Sciences*, **36**(6), 1021–1031.

[223] Cousens, B. L. & Clague, D. A., 2015. Shield to rejuvenated stage volcanism on Kauai and Niihau, Hawaiian Islands, *Journal of Petrology*, **56**(8), 1547–1584.

[224] Cox, K. G., 1993. Continental magmatic underplating, *Phil. Trans. R. Soc. Lond. A*, **342**(1663), 155–166.

[225] Cristofolini, R., Corsaro, R., & Ferlito, C., 1991. Variazioni petrochimiche nella successione Etnea: un riesame in base a nuovi dati da campioni di superficie e da sondaggi, *Acta Vulcanol*, **1**(2).

[226] Crocket, J. H., 2000. PGE in fresh basalt, hydrothermal alteration products, and volcanic incrustations of Kilauea volcano, Hawaii, *Geochimica et Cosmochimica Acta*, **64**(10), 1791–1807.

[227] Crocket, J. H., 2002. Platinum-group elements in basalts from Maui, Hawai'i: Low abundances in Alkali basalts, *Canadian Mineralogist*, **40**(2), 595–609.

[228] Cucciniello, C., Melluso, L., Morra, V., Storey, M., Rocco, I., Franciosi, L., Grifa, C., Petrone, C., & Vincent, M., 2011. New ^{40}Ar-^{39}Ar ages and petrogenesis of the Massif d'Ambre volcano, northern Madagascar, *Geological Society of America Special Papers*, **478**, 257–281.

[229] Cucciniello, C., Melluso, L., le Roex, A. P., Jourdan, F., Morra, V., de' Gennaro, R., & Grifa, C., 2017. From olivine nephelinite, basanite and basalt to peralkaline trachyphonolite and comendite in the Ankaratra volcanic complex, Madagascar: ^{40}Ar/^{39}Ar ages, phase compositions and bulk-rock geochemical and isotopic evolution, *Lithos*, **274-275**, 363–382.

[230] Cucciniello, C., le Roex, A., Jourdan, F., Morra, V., Grifa, C., Franciosi, L., & Melluso, L., 2018. The mafic alkaline volcanism of SW Madagascar (Ankililoaka, Tulear region): ^{40}Ar-^{39}Ar ages, geochemistry and tectonic setting, *Journal of the Geological Society*, **175**(4), 627–641.

[231] Cundari, A., 1973. Petrology of the leucite-bearing lavas in New South Wales, *Journal of the Geological Society of Australia*, **20**(4), 466–492.

[232] Daoud, M. A., Maury, R. C., Barrat, J.-A., Taylor, R. N., Le Gall, B., Guillou, H., Cotten, J., & Rolet, J., 2010. A LREE-depleted component in the Afar plume: Further evidence from Quaternary Djibouti basalts, *Lithos*, **114**(3-4), 327–336.

[233] Dautria, J.-M. & Liotard, J.-M., 1990. Les basaltes d'affinité tholéiitique de la marge Méditérranéenne Française, *Comptes rendus de l'Académie des sciences. Série 2, Mécanique, Physique, Chimie, Sciences de l'univers, Sciences de la Terre*, **311**(7), 821–827.

[234] Dautria, J.-M., Liotard, J.-M., Cabanes, N., Girod, M., & Briqueu, L., 1987. Amphibole-rich xenoliths and host alkali basalts: petrogenetic constraints and implications on the recent evolution of the upper mantle beneath Ahaggar (Central Sahara, Southern Algeria), *Contributions to Mineral Petrology*, **95**, 133–144.

[235] Dautria, J. M., Dostal, J., Dupuy, C., & Liotard, J. M., 1988. Geochemistry and petrogenesis of alkali basalts from Tahalra (Hoggar, Northwest Africa), *Chemical Geology*, **69**(1-2), 17–35.

[236] Dautria, J. M., Dupuy, C., Takherist, D., & Dostal, J., 1992. Carbonate metasomatism in the lithospheric mantle: peridotitic xenoliths from a melilititic district of the Sahara basin, *Contributions to Mineralogy and Petrology*, **111**(1), 37–52.

[237] Dautria, J.-M., Liotard, J.-M., & Briot, D., 2004. Particularités de la contamination crustale des phonolites: exemple du Velay oriental (Massif central), *Comptes Rendus Geoscience*, **336**(11), 971–981.

[238] Dautria, J.-M., Liotard, J.-M., Bosch, D., & Alard, O., 2010. 160 Ma of sporadic basaltic activity on the Languedoc volcanic line (Southern France): A peculiar case of lithosphere–asthenosphere interplay, *Lithos*, **120**(1-2), 202–222.

[239] David, K., Schiano, P., & Allegre, C., 2000. Assessment of the Zr/Hf fractionation in oceanic basalts and continental materials during petrogenetic processes, *Earth and Planetary Science Letters*, **178**(3-4), 285–301.

[240] Davidson, J., Hassanzadeh, J., Berzins, R., Stockli, D. F., Bashukooh, B., Turrin, B., & Pandamouz, A., 2004. The geology of Damavand volcano, Alborz Mountains, northern Iran, *Bulletin of the Geological Society of America*, **116**(1-2), 16–29.

[241] Davidson, J. P. & Wilson, I. R., 1989. Evolution of an alkali basalt-trachyte suite from Jebel Marra volcano, Sudan, through assimilation and fractional crystallization, *Earth and Planetary Science Letters*, **95**(1-2), 141–160.

[242] Davies, G., Cliff, R., Norry, M., & Gerlach, D., 1989. A combined chemical and Pb-Sr-Nd isotope study of the Azores and Cape Verde hot-spots: the geodynamic implications, *Geological Society, London, Special Publications*, **42**(1), 231–255.

[243] Davies, G. D. & LLoyd, F. E., 1989. Pb–Sr–Nd isotope and trace element data bearing on the origin of the potassic subcontinental lithosphere beneath south-west Uganda, Kimberlites and Related Rocks, *Geological Society of Australia Special Publication*, **14**, 784–794.

[244] Davies, G. R. & Macdonald, R., 1987. Crustal influences in the petrogenesis of the naivasha basalt - comendite complex: Combined trace element and Sr-Nd-Pb isotope constraints, *Journal of Petrology*, **28**(6), 1009–1031.

[245] Davis, A., Clague, D., & Paduan, J., 2007. Diverse origins of xenoliths from seamounts at the continental margin, offshore central California, *Journal of Petrology*, **48**(5), 829–852.

[246] Davis, A., Clague, D., Paduan, J., Cousens, B., & Huard, J., 2010. Origin of volcanic seamounts at the continental margin of California related to changes in plate margins, *Geochemistry, Geophysics, Geosystems*, **11**(5).

[247] Davis, A. S., Gunn, S. H., Bohrson, W. A., Gray, L.-B., & Hein, J. R., 1995. Chemically diverse, sporadic volcanism at seamounts offshore southern and Baja California, *Geological Society of America Bulletin*, **107**(5), 554–570.

[248] Davis, A. S., Clague, D. A., Bohrson, W. A., Dalrymple, G. B., & Greene, H. G., 2002. Seamounts at the continental margin of California: A different kind of oceanic intraplate volcanism, *Geological Society of America Bulletin*, **114**(3), 316–333.

[249] Davis, M. G., Garcia, M. O., & Wallace, P., 2003. Volatiles in glasses from Mauna Loa Volcano, Hawai'i: Implications for magma degassing and contamination, and growth of Hawaiian volcanoes, *Contributions to Mineralogy and Petrology*, **144**(5), 570–591.

[250] Dawson, J. & Smith, J., 1992. Olivine-mica pyroxenite xenoliths from northern Tanzania: metasomatic products of upper-mantle peridotite, *Journal of Volcanology and Geothermal Research*, **50**(1-2), 131–142.

[251] Dawson, J., Smith, J., & Jones, A., 1985. A comparative study of bulk rock and mineral chemistry of olivine melilitites and associated rocks from East and South Africa, *Neues Jahrbuch für Mineralogie, Abhandlungen*, **152**, 143–175.

[252] Day, J. M., Pearson, D. G., Macpherson, C. G., Lowry, D., & Carracedo, J. C., 2009. Pyroxenite-rich mantle formed by recycled oceanic lithosphere: Oxygen-osmium isotope evidence from Canary Island lavas, *Geology*, **37**(6), 555–558.

[253] Day, J. M., Pearson, D. G., Macpherson, C. G., Lowry, D., & Carracedo, J. C., 2010. Evidence for distinct proportions of subducted oceanic crust and lithosphere in HIMU-type mantle beneath El Hierro and La Palma, Canary Islands, *Geochimica et Cosmochimica Acta*, **74**(22), 6565–6589.

[254] De Mulder, M. & Pasteels, P., 1986. K–Ar geochronology of the Karisimbi volcano (Virunga, Rwanda-Zaire), *Journal of African Earth Sciences*, **5**(6), 575–579.

[255] De Mulder, M., Hertogen, J., Deutsch, S., & André, L., 1986. The role of crustal contamination in the potassic suite of the Karisimbi Volcano (Virunga, African Rift Valley), *Chemical Geology*, **57**(1-2), 117–136.

[256] Debaille, V., Trønnes, R. G., Brandon, A. D., Waight, T. E., Graham, D. W., & Lee, C. T. A., 2009. Primitive off-rift basalts from Iceland and Jan Mayen: Os-isotopic evidence for a mantle source containing enriched subcontinental lithosphere, *Geochimica et Cosmochimica Acta*, **73**(11), 3423–3449.

[257] Delavault, H., Chauvel, C., Sobolev, A., & Batanova, V., 2015. Combined petrological, geochemical and isotopic modeling of a plume source: Example of Gambier Island, Pitcairn chain, *Earth and Planetary Science Letters*, **426**, 23–35.

[258] Demant, A., Lestrade, P., Lubala, R. T., Kampunzu, A. B., & Durieux, J., 1994. Volcanological and petrological evolution of Nyiragongo volcano, Virunga volcanic field, Zaire, *Bulletin of Volcanology*, **56**(1), 47–61.

[259] Demény, A., Vennemann, T. W., Hegner, E., Ahijado, A., Casillas, R., Nagy, G., Homonnay, Z., Gutierrez, M., & Szabó, C., 2004. H, O, Sr, Nd, and Pb isotopic evidence for recycled oceanic crust in the Transitional Volcanic Group of Fuerteventura, Canary Islands, Spain, *Chemical Geology*, **205**(1-2), 37–54.

[260] Demény, A., Casillas, R., Hegner, E., Vennemann, T. W., Nagy, G., & Sipos, P., 2010. Geochemical and H-O-Sr-Nd isotope evidence for magmatic processes and meteoric-water interactions in the basal complex of La Gomera, Canary Islands, *Mineralogy and Petrology*, **98**(1-2), 181–195.

[261] Demidjuk, Z., Turner, S., Sandiford, M., George, R., Foden, J., & Etheridge, M., 2007. U-series isotope and geodynamic constraints on mantle melting processes beneath the Newer Volcanic Province in South Australia, *Earth and Planetary Science Letters*, **261**(3-4), 517–533.

[262] Demir, T., Westaway, R., Bridgland, D., Pringle, M., Yurtmen, S., Beck, A., & Rowbotham, G., 2007. Ar-Ar dating of late Cenozoic basaltic volcanism in northern Syria: Implications for the history of incision by the River Euphrates and uplift of the northern Arabian Platform, *Tectonics*, **26**, TC3012.

[263] Deniel, C., 1998. Geochemical and isotopic (Sr, Nd, Pb) evidence for plume-lithosphere interactions in the genesis of Grande Comore magmas (Indian Ocean), *Chemical Geology*, **144**(3-4), 281–303.

[264] Deniel, C., Vidal, P., Coulon, C., Vellutini, P. J., & Piguet, P., 1994. Temporal evolution of mantle sources during continental rifting: the volcanism of Djibouti (Afar), *Journal of Geophysical Research*, **99**(B2), 2853–2869.

[265] Dercq, M., Arndt, N., Lapierre, H., & Yirgu, G., 2001. The volcanic plugs of Ethiopia are feeders of trachytes in shield volcanoes., *Comptes Rendus De L'Academie Des Sciences Serie II Fascicule A – Sciences De La Terre Et Des Planetes*, **332**(10), 609–615.

[266] Déruelle, B., N'ni, J., & Kambou, R., 1987. Mount Cameroon: an active volcano of the Cameroon Line, *Journal of African Earth Sciences (1983)*, **6**(2), 197–214.

[267] Déruelle, B., Moreau, C., Nkoumbou, C., Kambou, R., Lissom, J., Njonfang, E., Ghogomu, R., & Nono, A., 1991. The Cameroon line: a review, in *Magmatism in extensional structural settings*, pp. 274–327, Springer.

[268] Desonie, D. L. & Duncan, R. A., 1990. The Cobb-Eickelberg Seamount Chain: Hotspot volcanism with mid-ocean ridge basalt affinity, *Journal of Geophysical Research: Solid Earth*, **95**(B8), 12697–12711.

[269] Desonie, D. L., Duncan, R. A., & Natland, J., 1993. Temporal and geochemical variability of volcanic products of the Marquesas hotspot, *Journal of Geophysical Research: Solid Earth*, **98**(B10), 17649–17665.

[270] Devey, C. W., Hémond, C., & Stoffers, P., 2000. Metasomatic reactions between carbonated plume melts and mantle harzburgite: the evidence from Friday and Domingo Seamounts (Juan Fernandez chain, SE Pacific), *Contributions to Mineralogy and Petrology*, **139**(1), 68–84.

[271] Di Battistini, G., Montanini, A., & Zerbi, M., 1990. Geochemistry of volcanic rocks from south-eastern Montiferro, *Neues Jahrb. Miner. Abh.*, **162**, 35–67.

[272] Di Bella, M., Russo, S., Petrelli, M., & Peccerillo, A., 2008. Origin and evolution of the Pleistocene magmatism of Linosa Island (Sicily Channel, Italy), *European Journal of Mineralogy*, **20**(4), 587–601.

[273] Di Giuseppe, P., Agostini, S., Lustrino, M., Karaoğlu, O., Savaşçin, Mehmet, Y., Manetti, P., & Ersoy, Y., 2017. Transition from Compression to Strike-slip Tectonics Revealed by Miocene–Pleistocene Volcanism West of the Karliova Triple Junction (East Anatolia), *Journal of Petrology*, **58**(10), 2055–2087.

[274] Di Grande, A., Mazzoleni, P., Lo Giudice, A., Beccaluva, L., Macciotta, G., & Siena, F., 2002. Subaerial Plio-Pleistocene volcanism in the geo-petrographic and structural context of the north/central Iblean region (Sicily), *Periodico di Mineralogia*, **71**(2), 159–189.

[275] Di Muro, A., Métrich, N., Vergani, D., Rosi, M., Armienti, P., Fougeroux, T., Deloule, E., Arienzo, I., & Civetta, L., 2014. The shallow plumbing system of Piton de la Fournaise Volcano (La Réunion Island, Indian Ocean) revealed by the major 2007 caldera-forming eruption, *Journal of Petrology*, **55**(7), 1287–1315.

[276] Díaz-Bravo, B. A., Gómez-Tuena, A., Ortega-Obregón, C., & Pérez-Arvizu, O., 2014. The origin of intraplate magmatism in the western Trans-Mexican volcanic belt, *Geosphere*, **10**(2), 340–373.

[277] Ding, L., Kapp, P., Zhong, D., & Deng, W., 2003. Cenozoic volcanism in Tibet: evidence for a transition from oceanic to continental subduction, *Journal of Petrology*, **44**(10), 1833–1865.

[278] Dixon, T. H., Batiza, R., Futa, K., & Martin, D., 1984. Petrochemistry, age and isotopic composition of alkali basalts from Ponape Island, Western Pacific, *Chemical Geology*, **43**(1-2), 1–28.

[279] Dobosi, G., 1995. Late-Cenozoic alkalic basalt magmatism in northern Hungary and Slovakia: petrology, source compositions and relationship to tectonics, *Acta Vulcano.*, **7**, 199–207.

[280] Dong, Y., Xiao, L., Zhou, H., Du, J., Zhang, N., Xiang, H., Wang, C., Zhao, Z., & Huang, H., 2010. Volcanism of the Nanpu Sag in the Bohai Bay Basin, Eastern China: Geochemistry, petrogenesis, and implications for tectonic setting, *Journal of Asian Earth Sciences*, **39**(3), 173–191.

[281] Dongmo, A. K., Wandji, P., Pouclet, A., Vicat, J.-P., Cheilletz, A., Nkouathio, D. G., Alexandrov, P., & Tchoua, F. M., 2001. Evolution volcanologique du mont Manengouba (Ligne du Cameroun); nouvelles données pétrographiques, géochimiques et géochronologiques, *Comptes Rendus de l'Académie des Sciences-Series IIA-Earth and Planetary Science*, **333**(3), 155–162.

[282] D'Orazio, M., Agostini, S., Mazzarini, F., Innocenti, F., Manetti, P., Haller, M. J., & Lahsen, A., 2000. The Pali Aike Volcanic Field, Patagonia: Slab-window magmatism near the tip of South America, *Tectonophysics*, **321**(4), 407–427.

[283] D'Orazio, M., Innocenti, F., Manetti, P., Haller, M. J., Di Vincenzo, G., & Tonarini, S., 2005. The Late Pliocene mafic lavas from the Camusú Aike volcanic field ($\sim 50°$S, Argentina): Evidence for geochemical variability in slab window magmatism, *Journal of South American Earth Sciences*, **18**(2), 107–124.

[284] Dostal, J., Dupuy, C., Zhai, M., & Zhi, X., 1988. Geochemistry and origin of Pliocene alkali basaltic lavas from Anhui-Jiangsu, eastern China, *Geochemical Journal*, **22**(4), 165–176.

[285] Dostal, J., Hamilton, T. S., & Church, B. N., 1996. The chilcotin basalts, british columbia (canada): geochemistry, petrogenesis and tectonic significance, *Neue Jahrb Miner Abhand Neues Jahrbuch Fur Mineralogie - Abhandlungen*, **170**(2), 207–229.

[286] Doucelance, R., Escrig, S., Moreira, M., Gariépy, C., & Kurz, M. D., 2003. Pb-Sr-He isotope and trace element geochemistry of the Cape Verde Archipelago, *Geochimica et Cosmochimica Acta*, **67**(19), 3717–3733.

[287] Downes, H., 1984. Sr and Nd isotope geochemistry of coexisting alkaline magma series, Cantal, Massif Central, France, *Earth and Planetary Science Letters*, **69**(2), 321–334.

[288] Downes, H., 1987. Tertiary and quaternary volcanism in the Massif Central, France, *Geological Society, London, Special Publications*, **30**(1), 517–530.

[289] Downs, D. T., Stelten, M. E., Champion, D. E., Dietterich, H. R., Nawab, Z., Zahran, H., Hassan, K., & Shawali, J., 2018. Volcanic history of the northernmost part of the Harrat Rahat volcanic field, Saudi Arabia, *Geosphere*, **14**(3), 1253–1282.

[290] DuBray, E. A., Stoeser, D. B., & McKee, E. H., 1991. Age and petrology of the Tertiary As Sirat volcanic field, Southwestern Saudi Arabia, Tech. rep., United States Department of the Interior Geological Survey.

[291] Duffield, W. A., Bullen, T., Clynne, M., Fournier, R., Janik, C., Lanphere, M., Lowenstern, J., Smith, J. G., Giorgis, L., Kahsai, G.,*et al.*, 1997. Geothermal potential of the Alid volcanic center, Danakil Depression, Eritrea, Tech. rep., US Geological Survey,.

[292] Duggen, S., Hoernle, K., van den Bogaard, P., & Garbe-Schönberg, D., 2005. Post-collisional transition from subduction-to intraplate-type magmatism in the westernmost Mediterranean: Evidence for continental-edge delamination of subcontinental lithosphere, *Journal of Petrology*, **46**(6), 1155–1201.

[293] Duggen, S., Hoernle, K. A., Hauff, F., Klügel, A., Bouabdellah, M., & Thirlwall, M. F., 2009. Flow of Canary mantle plume material through a subcontinental lithospheric corridor beneath Africa to the Mediterranean, *Geology*, **37**(3), 283–286.

[294] Duncan, R. A. & Al-amri, A. M., 2013. Timing and composition of volcanic activity at Harrat Lunayyir , western Saudi Arabia, *Journal of Volcanology and Geothermal Research*, **260**(April 2007), 103–116.

[295] Duncan, R. A., Kent, A. J., Thornber, C. R., Schlieder, T. D., & Al-Amri, A. M., 2016. Timing and composition of continental volcanism at Harrat Hutaymah, western Saudi Arabia, *Journal of Volcanology and Geothermal Research*, **313**, 1–14.

[296] Dunworth, E. A. & Wilson, M., 1998. Olivine melilitites of the SW German Tertiary volcanic province: Mineralogy and petrogenesis, *Journal of Petrology*, **39**(10), 1805–1836.

[297] Duprat, H. I., Friis, J., Holm, P. M., Grandvuinet, T., & Sørensen, R. V., 2007. The volcanic and geochemical development of São Nicolau, Cape Verde Islands: constraints from field and $^{40}Ar/^{39}Ar$ evidence, *Journal of Volcanology and Geothermal Research*, **162**(1-2), 1–19.

[298] Dupuy, C., Barsczus, H. G., Liotard, J., & Dostal, J., 1988. Trace element evidence for the origin of ocean island basalts: an example from the Austral Islands (French Polynesia), *Contributions to Mineralogy and Petrology*, **98**(3), 293–302.

[299] Dupuy, C., Barsczus, H. G., Dostal, J., Vidal, P., & Liotard, J. M., 1989. Subducted and recycled lithosphere as the mantle source of ocean island basalts from southern Polynesia, central Pacific, *Chemical Geology*, **77**(1), 1–18.

[300] Dupuy, C., Vidal, P., Maury, R. C., & Guille, G., 1993. Basalts from Mururoa, Fangataufa and Gambier islands (French Polynesia): Geochemical dependence on the age of the lithosphere, *Earth and Planetary Science Letters*, **117**, 89–100.

[301] Dyhr, C. T. & Holm, P. M., 2010. A volcanological and geochemical investigation of Boa Vista, Cape Verde Islands; $^{40}Ar/^{39}Ar$ geochronology and field constraints, *Journal of Volcanology and Geothermal Research*, **189**(1-2), 19–32.

[302] Dziewonski, A. M., Chou, T. A., & Woodhouse, J. H., 1981. Determination of earthquake source parameters from waveform data for studies of global and regional seismicity, *Journal of Geophysical Research*, **86**(B4), 2825–2852.

[303] Dzurisin, D., Lockwood, J. P., Casadevall, T. J., & Rubin, M., 1995. The Uwekahuna Ash Member of the Puna Basalt: product of violent phreatomagmatic eruptions at Kilauea volcano, Hawaii, between 2800 and 2100 14C years ago, *Journal of Volcanology and Geothermal Research*, **66**(1-4), 163–184.

[304] Eason, D. E. & Sinton, J. M., 2009. Lava shields and fissure eruptions of the Western Volcanic Zone, Iceland: Evidence for magma chambers and crustal interaction, *Journal of Volcanology and Geothermal Research*, **186**(3-4), 331–348.

[305] Eason, D. E., Sinton, J. M., Grönvold, K., & Kurz, M. D., 2015. Effects of deglaciation on the petrology and eruptive history of the Western Volcanic Zone, Iceland, *Bulletin of Volcanology*, **77**(6).

[306] Edwards, B., Russell, J., & Anderson, R., 2002. Subglacial, phonolitic volcanism at Hoodoo Mountain volcano, northern Canadian Cordillera, *Bulletin of Volcanology*, **64**(3-4), 254–272.

[307] Edwards, B. R., Skilling, I. P., Cameron, B., Haynes, C., Lloyd, A., & Hungerford, J. H., 2009. Evolution of an englacial volcanic ridge: Pillow Ridge tindar, Mount Edziza volcanic complex, NCVP, British Columbia, Canada, *Journal of Volcanology and Geothermal Research*, **185**(4), 251–275.

[308] Edwards, B. R., Russell, J. K., & Simpson, K., 2011. Volcanology and petrology of Mathews Tuya, northern British Columbia, Canada: Glaciovolcanic constraints on interpretations of the 0.730 Ma Cordilleran paleoclimate, *Bulletin of Volcanology*, **73**(5), 479–496.

[309] Eggins, S., Green, D. H., & Falloon, T. J., 1991. The Tasmantid Seamounts: shallow melting and contamination of an EM1 mantle plume, *Earth and Planetary Science Letters*, **107**(3-4), 448–462.

[310] Eisele, J., Sharma, M., Galer, S. J., Blichert-Toft, J., Devey, C. W., & Hofmann, A. W., 2002. The role of sediment recycling in EM-1 inferred from Os, Pb, Hf, Nd, Sr isotope and trace element systematics of the Pitcairn hotspot, *Earth and Planetary Science Letters*, **196**(3-4), 197–212.

[311] Eisele, S., Reißig, S., Freundt, A., Kutterolf, S., Nürnberg, D., Wang, K., & Kwasnitschka, T., 2015. Pleistocene to Holocene offshore tephrostratigraphy of highly explosive eruptions from the southwestern Cape Verde Archipelago, *Marine Geology*, **369**, 233–250.

[312] Ekici, T., Macpherson, C. G., & Otlu, N., 2012. Polybaric melting of a single mantle source during the Neogene Siverek phase of the Karacadağ Volcanic Complex, SE Turkey, *Lithos*, **146–147**, 152–163.

[313] Ekici, T., Macpherson, C. G., Otlu, N., & Fontignie, D., 2014. Foreland Magmatism during the Arabia–Eurasia Collision: Pliocene–Quaternary Activity of the Karacadağ Volcanic Complex, SW Turkey, *Journal of Petrology*, **55**, 1753–1777.

[314] El Azzouzi, M., Bernard-Griffiths, J., Bellon, H., Maury, R. C., Piqué, A., Fourcade, S., Cotten, J., & Hernandez, J., 1999. Évolution des sources du volcanisme marocain au cours du Néogène, *Comptes Rendus de l'Académie des Sciences-Series IIA - Earth and Planetary Science*, **329**(2), 95–102.

[315] El-Hasan, T. & Al-Malabeh, A., 2008. Geochemistry, Mineralogy and Petrogenesis of El-Lajjoun Pleistocene Alkali Basalt of Central Jordan, *Jordan Journal of Earth and Environmental Sciences*, **1**(2), 53–62.

[316] Elliott, T., Blichert-Toft, J., Heumann, A., Koetsier, G., & Forjaz, V., 2007. The origin of enriched mantle beneath São Miguel, Azores, *Geochimica et Cosmochimica Acta*, **71**(1), 219–240.

[317] Ellis, D. J., 1976. High pressure cognate inclusions in the Newer Volcanics of Victoria, *Contributions to Mineralogy and Petrology*, **58**(2), 149–180.

[318] Embey-isztin, A., Downes, H., James, D. E., Upton, B. G., Dobosi, G., Ingram, G. A., Harmon, R. S., & Scharbert, H. G., 1993. The petrogenesis of pliocene alkaline volcanic rocks from the Pannonian Basin, Eastern Central Europe, *Journal of Petrology*, **34**(2), 317–343.

[319] Ersoy, Y., Helvacı, C., Sözbilir, H., Erkül, F., & Bozkurt, E., 2008. A geochemical approach to Neogen-Quaternary volcanic activity of western Anatolia: An example of episodic bimodal volcanism within the Selendi Basin, Turkey, *Chemical Geology*, **255**, 265–282.

[320] Escrig, S., Doucelance, R., Moreira, M., & Allègre, C. J., 2005. Os isotope systematics in Fogo Island: evidence for lower continental crust fragments under the Cape Verde Southern Islands, *Chemical Geology*, **219**(1-4), 93–113.

[321] Esin, S. V., Ashchepkov, I. V., Ponomarchuk, V. A., Yamamoto, M., Travin, A. V., & Kiselyeva, V. Y., 1995. *Petrogenesis of alkaline basaltoids from the Vitim plateau: Baikal rift zone*, 2, Russian Academy of Sciences, Novosibirsk.

[322] Esperança, S. & Crisci, G. M., 1995. The island of Pantelleria: A case for the development of DMM-HIMU isotopic compositions in a long-lived extensional setting, *Earth and Planetary Science Letters*, **136**(3-4), 167–182.

[323] Espinoza, F., Morata, D., Pelleter, E., Maury, R. C., Suárez, M., Lagabrielle, Y., Polvé, M., Bellon, H., Cotten, J., De la Cruz, R., & Guivel, C., 2005. Petrogenesis of the Eocene and Mio-Pliocene alkaline basaltic magmatism in Meseta Chile Chico, southern Patagonia, Chile: Evidence for the participation of two slab windows, *Lithos*, **82**(3-4 SPEC. ISS.), 315–343.

[324] Ewart, A., Chappell, B., & Menzies, M., 1988. An overview of the geochemical and isotopic characteristics of the eastern Australian CainozoicVolcanic Provinces, *Journal of Petrology*, **1**, 225–273.

[325] Fan, Q. & Hooper, P. R., 1991. The cenozoic basaltic rocks of Eastern China: Petrology and chemical composition, *Journal of Petrology*, **32**(4), 765–810.

[326] Fan, Q. C., Chen, S. S., Zhao, Y. W., Zou, H. B., Li, N., & Sui, J. L., 2014. Petrogenesis and evolution of Quaternary basaltic rocks from the Wulanhada area, North China, *Lithos*, **206-207**(1), 289–302.

[327] Farley, K., Basu, A., & Craig, H., 1993. He, Sr and Nd isotopic variations in lavas from the Juan Fernandez Archipelago, SE Pacific, *Contributions to Mineralogy and Petrology*, **115**(1), 75–87.

[328] Fedele, L., Lustrino, M., Melluso, L., Morra, V., & D'Amelio, F., 2007. The Pliocene Montiferro volcanic complex (central-western Sardinia, Italy): Geochemical observations and petrological implications, *Periodico di Mineralogia*, **76**(2), 101–136.

[329] Feigenson, M. D., 1984. Geochemistry of Kauai volcanics and a mixing model for the origin of Hawaiian alkali basalts, *Contributions to Mineralogy and Petrology*, **87**(2), 109–119.

[330] Feigenson, M. D., Hofmann, A. W., & Spera, F. J., 1983. Case studies on the origin of basalt - II. The transition from tholeiitic to alkalic volcanism on Kohala volcano, Hawaii, *Contributions to Mineralogy and Petrology*, **84**(4), 390–405.

[331] Feraud, G., Gastaud, J., Schmincke, H., Pritchard, G., Lietz, J., & Bleil, U., 1981. New K-Ar ages, chemical analyses and magnetic data of rocks from the islands of Santa Maria (Azores), Porto Santo and Madeira (Madeira Archipelago) and Gran Canaria (Canary Islands), *Bulletin volcanologique*, **44**(3), 359–375.

[332] Feyissa, D. H., Shinjo, R., Kitagawa, H., Meshesha, D., & Nakamura, E., 2017. Petrologic and geochemical characterization of rift-related magmatism at the northernmost Main Ethiopian Rift: Implications for plume-lithosphere interaction and the evolution of rift mantle sources, *Lithos*, **282-283**, 240–261.

[333] Field, L., Blundy, J., Calvert, A., & Yirgu, G., 2013. Magmatic history of Dabbahu, a composite volcano in the Afar Rift, Ethiopia, *Bulletin of the Geological Society of America*, **125**(1-2), 128–147.

[334] Finlayson, V., Konter, J., Konrad, K., Koppers, A., Jackson, M., & Rooney, T., 2018. Sr–Pb–Nd–Hf isotopes and $^{40}Ar/^{39}Ar$ ages reveal a Hawaii–Emperor-style bend in the Rurutu hotspot, *Earth and Planetary Science Letters*, **500**, 168–179.

[335] Finocchiaro, S., 1994. *Petrologia della successione delle lave etnee con riferimento ai livelli alcalini antichi sulla base di dati da campioni di sottosuolo*, Ph.D. thesis, Università degli Studi di Catania.

[336] Fisk, M. R., Upton, B. G., Ford, C. E., & White, W. M., 1988. Geochemical and experimental study of the genesis of magmas of Reunion Island, Indian Ocean, *Journal of Geophysical Research*, **93**(B5), 4933–4950.

[337] Fitton, J., 1987. The Cameroon line, West Africa: a comparison between oceanic and continental alkaline volcanism, *Geological Society, London, Special Publications*, **30**(1), 273–291.

[338] Fitton, J., Kilburn, C., Thirlwall, M., & Hughes, D., 1983. 1982 eruption of Mount Cameroon, west Africa, *Nature*, **306**(5941), 327.

[339] Fitton, J. G., Saunders, A. D., Kempton, P. D., & Hardarson, B. S., 2003. Does depleted mantle form an intrinsic part of the Iceland plume?, *Geochemistry, Geophysics, Geosystems*, **4**(3), 1–14.

[340] Foden, J., Song, S. H., Turner, S., Elburg, M., Smith, P., Van der Steldt, B., & Van Penglis, D., 2002. Geochemical evolution of lithospheric mantle beneath SE South Australia, *Chemical Geology*, **182**(2-4), 663–695.

[341] Fodor, R., Bauer, G., Jacobs, R., & Bornhorst, T., 1987. Kahoolawe Island, Hawaii: Tholeiitic, alkalic, and unusual hydrothermal "enrichment" characteristics, *Journal of Volcanology and Geothermal Research*, **31**, 171–176.

[342] Fodor, R. V., Frey, F. A., Bauer, G. R., & Clague, D. A., 1992. Ages, rare-earth element enrichment, and petrogenesis of tholeiitic and alkalic basalts from Kahoolawe Island, Hawaii, *Contributions to Mineralogy and Petrology*, **110**(4), 442–462.

[343] Fodor, R. V., Mukasa, S. B., & Sial, A. N., 1998. Isotopic and trace-element indications of lithospheric and asthenospheric components in Tertiary alkalic basalts, northeastern Brazil, *Lithos*, **43**(4), 197–217.

[344] Fosso, J., Ménard, J. J., Bardintzeff, J. M., Wandji, P., Tchoua, F. M., & Bellon, H., 2005. Les laves du mont Bangou : Une première manifestation volcanique Éocène, à affinité transitionnelle, de la Ligne du Cameroun, *Comptes Rendus - Geoscience*, **337**(3), 315–325.

[345] Francis, D. & Ludden, J., 1995. The signature of amphibole in mafic alkaline lavas, a study in the northern Canadian Cordillera, *Journal of Petrology*, **36**(5), 1171–1191.

[346] Franz, G., Steiner, G., Volker, F., Pudlo, D., & Hammerschmidt, K., 1999. Plume related alkaline magmatism in central Africa-the Meidob Hills (W Sudan), *Chemical Geology*, **157**(1-2), 27–47.

[347] French, S. W. & Romanowicz, B. A., 2014. Whole-mantle radially anisotropic shear velocity structure from spectral-element waveform tomography, *Geophysical Journal International*, **199**(3), 1303–1327.

[348] Fretzdorff, S. & Haase, K. M., 2002. Geochemistry and petrology of lavas from the submarine flanks of Réunion Island (western Indian Ocean): Implications for magma genesis and the mantle source, *Mineralogy and Petrology*, **75**(3-4), 153–184.

[349] Fretzdorff, S., Haase, K., & Garbe-Schönberg, C.-D., 1996. Petrogenesis of lavas from the Umu Volcanic Field in the young Hotspot Region west of Easter Island, southeastern Pacific, *Lithos*, **38**(1-2), 23–40.

[350] Frey, F., Green, D., & Roy, S., 1978. Integrated models of basalt petrogenesis: a study of quartz tholeiites to olivine melilitites from south eastern Australia utilizing geochemical and experimental petrological data, *Journal of petrology*, **19**(3), 463–513.

[351] Frey, F., Wise, W., Garcia, M., West, H., Kwon, S., & Kennedy, A., 1990. Evolution of Mauna Kea Volcano, Hawaii: Petrologic and geochemical constraints on postshield volcanism, *Journal of Geophysical Research*, **95**, 1271–1300.

[352] Frey, F. A., Garcia, M. O., & Roden, M. F., 1994. Geochemical characteristics of Koolau Volcano: Implications of intershield geochemical differences among Hawaiian volcanoes, *Geochimica et Cosmochimica Acta*, **58**(5), 1441–1462.

[353] Friedman, E., Polat, A., Thorkelson, D. J., & Frei, R., 2016. Lithospheric mantle xenoliths sampled by melts from upwelling asthenosphere: The Quaternary Tasse alkaline basalts of southeastern British Columbia, Canada, *Gondwana Research*, **33**, 209–230.

[354] Furman, T., 1995. Melting of metasomatized subcontinental lithosphere: undersaturated mafic lavas from Rungwe, Tanzania, *Contributions to Mineralogy and Petrology*, **122**(1-2), 97–115.

[355] Furman, T. & Graham, D., 1999. Erosion of lithospheric mantle beneath the East African Rift system: Geochemical evidence from the Kivu volcanic province, *Developments in Geotectonics*, **24**(C), 237–262.

[356] Furman, T., Frey, F. A., & Meyer, P. S., 1992. Petrogenesis of evolved basalts and rhyolites at austurhorn, Southeastern Iceland: The role of fractional crystallization, *Journal of Petrology*, **33**(6), 1405–1445.

[357] Furman, T., Bryce, J. G., Karson, J., & Iotti, A., 2004. East African Rift System (EARS) plume structure: Insights from Quaternary mafic lavas of Turkana, Kenya, *Journal of Petrology*, **45**(5), 1069–1088.

[358] Furman, T., Kaleta, K. M., Bryce, J. G., & Hanan, B. B., 2006. Tertiary mafic lavas of Turkana, Kenya: Constraints on East African plume structure and the occurrence of high-μ volcanism in Africa, *Journal of Petrology*, **47**(6), 1221–1244.

[359] Gaffney, A. M., 2002. Environments of Crystallization and Compositional Diversity of Mauna Loa Xenoliths, *Journal of Petrology*, **43**(6), 963–981.

[360] Gaffney, A. M., Nelson, B. K., & Blichert-Toft, J., 2004. Geochemical constraints on the role of oceanic lithospere in intra-volcano heterogeneity at West Maui, Hawaii, *Journal of Petrology*, **45**(8), 1663–1687.

[361] Galipp, K., Klügel, A., & Hansteen, T. H., 2006. Changing depths of magma fractionation and stagnation during the evolution of an oceanic island volcano: La Palma (Canary Islands), *Journal of Volcanology and Geothermal Research*, **155**(3-4), 285–306.

[362] Gamble, J. A., Adams, C. J., Morris, P. A., Wysoczanski, R. J., Handler, M., & Timm, C., 2018. The geochemistry and petrogenesis of Carnley Volcano, Auckland Islands, SW Pacific, *New Zealand Journal of Geology and Geophysics*, **61**(4), 480–497.

[363] Gao, Y., Hou, Z., Kamber, B. S., Wei, R., Meng, X., & Zhao, R., 2007. Lamproitic rocks from a continental collision zone: evidence for recycling of subducted Tethyan oceanic sediments in the mantle beneath southern Tibet, *Journal of Petrology*, **48**(4), 729–752.

[364] Garapić, G., Jackson, M. G., Hauri, E. H., Hart, S. R., Farley, K. A., Blusztajn, J. S., & Woodhead, J. D., 2015. A radiogenic isotopic (He-Sr-Nd-Pb-Os) study of lavas from the Pitcairn hotspot: Implications for the origin of EM-1 (enriched mantle 1), *Lithos*, **228-229**, 1–11.

[365] Garcia, M. O., 1996. Petrography and olivine and glass chemistry of lavas from the Hawaii Scientific Drilling Project, *Journal of Geophysical Research: Solid Earth*, **101**(B5), 11701–11713.

[366] Garcia, M. O., Frey, F. A., & Grooms, D. G., 1986. Petrology of volcanic rocks from Kaula Island, Hawaii, *Contributions to Mineralogy and Petrology*, **94**, 461–471.

[367] Garcia, M. O., Rhodes, J. M., Wolfe, E. W., Ulrich, G. E., & Ho, R. A., 1992. Petrology of lavas from episodes 2-47 of the Puu Oo eruption of Kilauea Volcano, Hawaii: Evaluation of magmatic processes, *Bulletin of Volcanology*, **55**(1-2), 1–16.

[368] Garcia, M. O., Foss, D. J. P., West, H. B., & Mahoney, J. J., 1995. Geochemical and Isotopic Evolution of Loihi Volcano, Hawaii, *Journal of Petrology*, **36**(6), 1647–1671.

[369] Garcia, M. O., Rubin, K. H., Norman, M. D., Rhodes, J. M., Graham, D. W., Muenow, D. W., & Spencer, K., 1998. Petrology and geochronology of basalt breccia from the 1996 earthquake swarm of Loihi Seamount, Hawaii: magmatic history of its 1996 eruption, *Bulletin of Volcanology*, **59**(8), 577–592.

[370] Garcia, M. O., Pietruszka, A. J., Rhodes, J. M., & Swanson, K., 2000. Magmatic processes during the prolonged Pu'u 'O'o eruption of Kilauea Volcano, Hawaii, *Journal of Petrology*, **41**(7), 967–990.

[371] Garcia, M. O., Pietruszka, A. J., & Rhodes, J. M., 2003. A Petrologic Perspective of Kilauea Volcano's Summit Magma Reservoir, *Journal of Petrology*, **44**(12), 2313–2339.

[372] Gasparon, M., Innocenti, F., Manetti, P., Peccerillo, A., & Tsegaye, A., 1993. Genesis of the pliocene to recent bimodal mafic-felsic volcanism in the Debre Zeyt area, central Ethiopia: volcanological and geochemical constraints, *Journal of African Earth Sciences*, **17**(2), 145–165.

[373] Geist, D., White, W. M., Albarede, F., Harpp, K., Reynolds, R., Blichert-Toft, J., & Kurz, M. D., 2002. Volcanic evolution in the Galápagos: The dissected shield of Volcan Ecuador, *Geochemistry, Geophysics, Geosystems*, **3**(10).

[374] Geist, D. J., McBirney, A. R., & Duncan, R. A., 1985. Geology of Santa Fe Island: The oldest Galapagos volcano, *Journal of Volcanology and Geothermal Research*, **12**, 203–212.

[375] Geist, D. J., Naumann, T. R., Standish, J. J., Kurz, M. D., Harpp, K. S., White, W. M., & Fornari, D. J., 2005. Wolf volcano, Galápagos Archipelago: Melting and magmatic evolution at the margins of mantle plume, *Journal of Petrology*, **46**(11), 2197–2224.

[376] Geist, D. J., Fornari, D. J., Kurz, M. D., Harpp, K. S., Soule, S. A., Perfit, M. R., & Koleszar, A. M., 2006. Submarine Fernandina: Magmatism at the leading edge of the Galápagos hot spot, *Geochemistry, Geophysics, Geosystems*, **7**(12).

[377] Geldmacher, J. & Hoernle, K., 2000. The 72 Ma geochemical evolution of the Madeira hotspot (eastern North Atlantic): recycling of oceanic lithosphere, *Earth and Planetary Science Letters*, **183**, 73–92.

[378] Geldmacher, J., Hoernle, K., Klügel, A., v.d. Bogaard, P., Wombacher, F., & Berning, B., 2006. Origin and geochemical evolution of the Madeira-Tore Rise (eastern North Atlantic), *Journal of Geophysical Research: Solid Earth*, **111**(9).

[379] Gencalioglu-Kuscu, G., 2011. Geochemical characterization of a Quaternary monogenetic volcano in Erciyes Volcanic Complex: Cora Maar (Central Anatolian Volcanic Province, Turkey), *International Journal of Earth Sciences*, **100**, 1967–1985.

[380] Gencalioglu-Kuscu, G. & Geneli, F., 2010. Review of post-collisional volcanism in the Central Anatolian Volcanic Province (Turkey), with special reference to the Tepekoy Volcanic Complex, *International Journal of Earth Sciences*, **99**, 593–621.

[381] Genske, F. S., Turner, S. P., Beier, C., & Schaefer, B. F., 2012. The petrology and geochemistry of lavas from the western Azores islands of Flores and Corvo, *Journal of Petrology*, **53**(8), 1673–1708.

[382] George, R., 1999. The petrogenesis of Plio-Pleistocene alkaline volcanic rocks from the Tosa Sucha region, Arba Minch, southern main Ethiopian rift, *Acta Vulcanol.*, **11**, 121–131.

[383] George, R. M. & Rogers, N. W., 2002. Plume dynamics beneath the African plate inferred from the geochemistry of the Tertiary basalts of southern Ethiopia, *Contributions to Mineralogy and Petrology*, **144**(3), 286–304.

[384] Gerlach, D. C., Cliff, R. A., Davies, G. R., Norry, M., & Hodgson, N., 1988. Magma sources of the Cape Verdes archipelago: Isotopic and trace element constraints, *Geochimica et Cosmochimica Acta*, **52**(12), 2979–2992.

[385] Ghorbani, M. R. & Middlemost, E. A., 2000. Geochemistry of pyroxene inclusions from the Warrumbungle Volcano, New South Wales, Australia, *American Mineralogist*, **85**(10), 1349–1367.

[386] Gibson, S. A. & Geist, D., 2010. Geochemical and geophysical estimates of lithospheric thickness variation beneath Galápagos, *Earth and Planetary Science Letters*, **300**(3-4), 275–286.

[387] Gibson, S. A., Thompson, R. N., Day, J. A., Humphris, S. E., & Dickin, A. P., 2005. Melt-generation processes associated with the Tristan mantle plume: Constraints on the origin of EM-1, *Earth and Planetary Science Letters*, **237**(3-4), 744–767.

[388] Giordano, F., D'Antonio, M., Civetta, L., Tonarini, S., Orsi, G., Ayalew, D., Yirgu, G., Dell'Erba, F., Di Vito, M., & Isaia, R., 2014. Genesis and evolution of mafic and felsic magmas at Quaternary volcanoes within the Main Ethiopian Rift: Insights from Gedemsa and Fanta'Ale complexes, *Lithos*, **188**, 130–144.

[389] Gluhak, T. M. & Rosenberg, D., 2013. Geochemical discrimination of basaltic sources as a tool for provenance analyses of bifacial tools in the southern Levant: First results from the Jezreel Valley, Israel, *Journal of Archaeological Science*, **40**(3), 1611–1622.

[390] Godano, R. F., 2000. *Il magmatismo Plio-Quaternario sardo. Correlazioni con l'evoluzione geodinamica della Sardegna*, Ph.D. thesis, University of Rome.

[391] Gómez-Tuena, A., LaGatta, A. B., Langmuir, C. H., Goldstein, S. L., Fernando, O. G., & Carrasco-Núñez, G., 2003. Temporal control of subduction magmatism in the eastern Trans-Mexican Volcanic Belt: Mantle sources, slab contributions, and crustal contamination, *Geochemistry, Geophysics, Geosystems*, **4**(8).

[392] Gorring, M., Singer, B., Gowers, J., & Kay, S. M., 2003. Plio-Pleistocene basalts from the Meseta del Lago Buenos Aires, Argentina: Evidence for asthenosphere-lithosphere interactions during slab window magmatism, *Chemical Geology*, **193**(3-4), 215–235.

[393] Gorring, M. L. & Kay, S. M., 2001. Mantle processes and sources of neogene slab window magmas from Southern Patagonia, Argentina, *Journal of Petrology*, **42**(6), 1067–1094.

[394] Gorring, M. L., Kay, S. M., Zeitler, P. K., Ramos, V. A., Rubiolo, D., Fernandez, M. I., & Panza, J. L., 1997. Neogene Patagonian plateau lavas: continental magmas associated with ridge collision at the Chile Triple Junction, *Tectonics*, **16**(1), 1–17.

[395] Goto, Y. & McPhie, J., 1996. A Miocene basanite peperitic dyke at Stanley, northwestern Tasmania, Australia, *Journal of Volcanology and Geothermal Research*, **74**(1-2), 111–120.

[396] Goto, Y. & McPhie, J., 2004. Morphology and propagation styles of Miocene submarine basanite lavas at Stanley, northwestern Tasmania, Australia, *Journal of Volcanology and Geothermal Research*, **130**(3-4), 307–328.

[397] Gourgaud, A. & Vincent, P. M., 2004. Petrology of two continental alkaline intraplate series at Emi Koussi volcano, Tibesti, Chad, *Journal of Volcanology and Geothermal Research*, **129**(4), 261–290.

[398] Grachev, A., 1998. The Khamar-Daban Ridge as a hotspot of the Baikal Rift from data of chemical geodynamics, *Izvestiya. Physics of the Solid Earth*, **34**(3), 175–200.

[399] Grachev, A. F., 2017. The nature of Late Cenozoic volcanism in the Udokan Range, northwestern segment of the Baikal Rift Zone, *Russian Journal of Earth Sciences*, **17**(1), 1–9.

[400] Graham, D. W., Humphris, S. E., Jenkins, W. J., & Kurz, M. D., 1992. Helium isotope geochemistry of some volcanic rocks from Saint Helena, *Earth and Planetary Science Letters*, **110**(1-4), 121–131.

[401] Green, T. H., 1992. Petrology and geochemistry of basaltic rocks from the Balleny Is, Antarctica, *Australian Journal of Earth Sciences*, **39**(5), 603–617.

[402] Grützner, T., Prelević, D., & Akal, C., 2013. Geochemistry and origin of ultramafic enclaves and their basanitic host rock from Kula Volcano, Turkey, *Lithos*, **180–181**, 58–73.

[403] Guillou, H., Guille, G., Brousse, R., & Bardintzeff, J.-M., 1990. Tholeiitic to alkali basalts evolution of the Fangataufa atoll volcanic basement (French Polynesia), *Bulletin de la Societe Geologique de France*, **8**(3), 537–549.

[404] Guillou, H., Garcia, M. O., & Turpin, L., 1997. Unspiked K-Ar dating of young volcanic rocks from Loihi and Pitcairn hot spot seamounts, *Journal of Volcanology and Geothermal Research*, **78**(3-4), 239–249.

[405] Guillou, H., Sinton, J., Laj, C., Kissel, C., & Szeremeta, N., 2000. New K–Ar ages of shield lavas from Waianae volcano, Oahu, Hawaiian archipelago, *Journal of Volcanology and Geothermal Research*, **96**(3-4), 229–242.

[406] Guillou, H., Maury, R. C., Guille, G., Chauvel, C., Rossi, P., Pallares, C., Legendre, C., Blais, S., Liorzou, C., & Deroussi, S., 2014. Volcanic successions in Marquesas eruptive centers: A departure from the Hawaiian model, *Journal of Volcanology and Geothermal Research*, **276**, 173–188.

[407] Guimarães, A. R., Fitton, J. G., Kirstein, L. A., & Barfod, D. N., 2020. Contemporaneous intraplate magmatism on conjugate South Atlantic margins: A hotspot conundrum, *Earth and Planetary Science Letters*, **536**, 116147.

[408] Guo, P., Niu, Y., Ye, L., Liu, J., Sun, P., Cui, H., Zhang, Y., Gao, J., Su, L., Zhao, J., & Feng, Y., 2014. Lithosphere thinning beneath west North China Craton: Evidence from geochemical and Sr-Nd-Hf isotope compositions of Jining basalts, *Lithos*, **202-203**, 37–54.

[409] Guo, P., Niu, Y., Sun, P., Ye, L., Liu, J., Zhang, Y., xing Feng, Y., & xin Zhao, J., 2016. The origin of Cenozoic basalts from central Inner Mongolia, East China: The consequence of recent mantle metasomatism genetically associated with seismically observed paleo-Pacific slab in the mantle transition zone, *Lithos*, **240-243**, 104–118.

[410] Guo, Z., Wilson, M., Liu, J., & Mao, Q., 2006. Post-collisional, potassic and ultrapotassic magmatism of the northern Tibetan Plateau: Constraints on characteristics of the mantle source, geodynamic setting and uplift mechanisms, *Journal of Petrology*, **47**(6), 1177–1220.

[411] Guo, Z., Wilson, M., Zhang, M., Cheng, Z., & Zhang, L., 2013. Post-collisional, K-rich mafic magmatism in south Tibet: constraints on Indian slab-to-wedge transport processes and plateau uplift, *Contributions to Mineralogy and Petrology*, **165**(6), 1311–1340.

[412] Guo, Z., Wilson, M., Zhang, L., Zhang, M., Cheng, Z., & Liu, J., 2014. The role of subduction channel mélanges and convergent subduction systems in the petrogenesis of post-collisional K-rich mafic magmatism in NW Tibet, *Lithos*, **198–199**, 184–201.

[413] Guo, Z., Cheng, Z., Zhang, M., Zhang, L., Li, X., & Liu, J., 2015. Post-collisional high-K calc-alkaline volcanism in Tengchong volcanic field, SE Tibet: constraints on Indian eastward subduction and slab detachment, *Journal of the Geological Society*, **172**(5), 624–640.

[414] Guo, Z., Cheng, Z., Zhang, M., Zhang, L., Li, X., & Liu, J., 2015. Post-collisional high-K calc-alkaline volcanism in Tengchong volcanic field, SE Tibet: constraints on Indian eastward subduction and slab detachment, *Journal of the Geological Society*, **172**(5), 624–640.

[415] Gurenko, A., Sobolev, A., & Kononkova, N., 1992. New petrological data on Icelandic rift alkali basalts, *Geochem. Int*, **29**, 41–53.

[416] Gurenko, A. A. & Chaussidon, M., 2002. Oxygen isotope variations in primitive tholeiites of Iceland: Evidence from a SIMS study of glass inclusions, olivine phenocrysts and pillow rim glasses, *Earth and Planetary Science Letters*, **205**(1-2), 63–79.

[417] Gurenko, A. A., Hoernle, K. A., Hauff, F., Schmincke, H. U., Han, D., Miura, Y. N., & Kaneoka, I., 2006. Major, trace element and Nd-Sr-Pb-O-He-Ar isotope signatures of shield stage lavas from the central and western Canary Islands: Insights into mantle and crustal processes, *Chemical Geology*, **233**(1-2), 75–112.

[418] Haase, K. & Devey, C. W., 1996. Geochemistry of lavas from the Ahu and Tupa volcanic fields, Easter Hotspot, southeast Pacific: Implications for intraplate magma genesis near a spreading axis, *Earth and Planetary Science Letters*, **137**(1-4), 129–143.

[419] Haase, K. M., Stoffers, P., & Garbe-Schönberg, C. D., 1997. The petrogenetic evolution of lavas from Easter Island and neighbouring seamounts, near-ridge hotspot volcanoes in the SE Pacific, *Journal of Petrology*, **38**(6), 785–813.

[420] Haase, K. M., Goldschmidt, B., & Garbe-Schönberg, C. D., 2004. Petrogenesis of Tertiary Continental Intra-plate Lavas from the Westerwald Region, Germany, *Journal of Petrology*, **45**(5), 883–905.

[421] Hagedorn, B., Gersonde, R., Gohl, K., & Hubberten, H.-W., 2007. Petrology, geochemistry and K/Ar age constraints of the eastern De Gerlache Seamount alkaline basalts (Bellingshausen Sea, southeast Pacific), *Polarforschung*, **76**(3), 87–94.

[422] Hagos, M., Koeberl, C., Kabeto, K., & Koller, F., 2010. Geochemical characteristics of the alkaline basalts and the phonolite-trachyte plugs of the Axum area, Northern Ethiopia, *Austrian Journal of Earth Sciences*, **103**(2).

[423] Hagos, M., Koeberl, C., & de Vries, B. v. W., 2016. The Quaternary volcanic rocks of the northern Afar Depression (northern Ethiopia): Perspectives on petrology, geochemistry, and tectonics, *Journal of African Earth Sciences*, **117**, 29–47.

[424] Haileab, B., Brown, F. H., McDdougall, I., & Gathogo, P. N., 2004. Gombe Group basalts and initiation of Pliocene deposition in the Turkana depression, northern Kenya and southern Ethiopia, *Geological Magazine*, **141**(1), 41–53.

[425] Halliday, A., Dickin, A., Fallick, A., & Fitton, J., 1988. Mantle dynamics: a Nd, Sr, Pb and O isotopic study of the Cameroon line volcanic chain, *Journal of Petrology*, **29**(1), 181–211.

[426] Halliday, A. N., Davidson, J. P., Holden, P., DeWolf, C., Lee, D. C., & Fitton, J. G., 1990. Trace-element fractionation in plumes and the origin of HIMU mantle beneath the Cameroon line, *Nature*, **347**(6293), 523–528.

[427] Handley, H. K., Turner, S., Berlo, K., Beier, C., & Saal, A. E., 2011. Insights into the Galápagos plume from uranium-series isotopes of recently erupted basalts, *Geochemistry, Geophysics, Geosystems*, **12**(9), 1–16.

[428] Hanyu, T., Tatsumi, Y., Senda, R., Miyazaki, T., Chang, Q., Hirahara, Y., Takahashi, T., Kawabata, H., Suzuki, K., Kimura, J.-I.,*et al.*, 2011. Geochemical characteristics and origin of the HIMU reservoir: A possible mantle plume source in the lower mantle, *Geochemistry, Geophysics, Geosystems*, **12**(2).

[429] Hanyu, T., Dosso, L., Ishizuka, O., Tani, K., Hanan, B. B., Adam, C., Nakai, S., Senda, R., Chang, Q., & Tatsumi, Y., 2013. Geochemical diversity in submarine HIMU basalts from Austral Islands, French Polynesia, *Contributions to Mineralogy and Petrology*, **166**(5), 1285–1304.

[430] Hao, L.-L., Wang, Q., Wyman, D. A., Qi, Y., Ma, L., Huang, F., Zhang, L., Xia, X. P., & Ou, Q., 2018. First identification of mafic igneous enclaves in Miocene lavas of southern Tibet with implications for Indian continental subduction, *Geophysical Research Letters*, **45**(16), 8205–8213.

[431] Harangi, S., Vaselli, O., Kovacs, R., Tonarini, S., Coradossi, N., & Ferraro, D., 1994. Volcanological and magmatological studies on the Neogene basaltic volcanoes of the southern Little Hungarian Plain, Pannonian Basin (western Hungary), *Mineralogica et Petrographica Acta*, **37**(January), 183–197.

[432] Hardarson, B., Fitton, J., Ellam, R., & Pringle, M., 1997. Rift relocation — A geochemical and geochronological investigation of a palaeo-rift in northwest Iceland, *Earth and Planetary Science Letters*, **153**(3-4), 181–196.

[433] Hards, V. L., Kempton, P. D., & Thompson, R. N., 1995. The heterogeneous Iceland plume: new insights from the alkaline basalts of the Snaefell volcanic centre, *Journal of the Geological Society*, **152**(6), 1003–1009.

[434] Hards, V. L., Kempton, P. D., Thompson, R. N., & Greenwood, P. B., 2000. The magmatic evolution of the Snaefell volcanic centre; an example of volcanism during incipient rifting in Iceland, *Journal of Volcanology and Geothermal Research*, **99**(1-4), 97–121.

[435] Hare, A., Cas, R., Musgrave, R., & Phillips, D., 2005. Magnetic and chemical stratigraphy for the Werribee Plains basaltic lava flow-field, Newer Volcanics Province, southeast Australia: implications for eruption frequency, *Australian Journal of Earth Sciences*, **52**(1), 41–57.

[436] Harnois, L. & Stevenson, R. K., 2006. Major and trace elements geochemistry of basalts and trachyphonolites from Huahine Island, Society archipelago (French Polynesia), *Bulletin de la Societe Geologique de France*, **177**(4), 179–190.

[437] Harpp, K. S., Fornari, D. J., Geist, D. J., & Kurz, M. D., 2003. Genovesa Submarine Ridge: A manifestation of plume-ridge interaction in the northern Galápagos Islands, *Geochemistry, Geophysics, Geosystems*, **4**(9).

[438] Harpp, K. S., Wanless, V. D., Otto, R. H., Hoernle, K., & Werner, R., 2005. The Cocos and Carnegie aseismic ridges: A trace element record of long-term plume–spreading center interaction, *Journal of Petrology*, **46**(1), 109–133.

[439] Harris, N. R., 1998. *Isotopic, geochemical and geochronological constrains on the origin and evolution of Cenozoic volcanism, Baikal Rift Zone, Siberia*, Ph.D. thesis, Massachusetts Institute of Technology.

[440] Hart, S., Coetzee, M., Workman, R., Blusztajn, J., Johnson, K., Sinton, J., Steinberger, B., & Hawkins, J., 2004. Genesis of the Western Samoa seamount province: age, geochemical fingerprint and tectonics, *Earth and Planetary Science Letters*, **227**(1-2), 37–56.

[441] Hart, S. R. & Jackson, M. G., 2014. Ta'u and Ofu/Olosega volcanoes: The "Twin Sisters" of Samoa, their P, T, X melting regime, and global implications, *Geochemistry, Geophysics, Geosystems*, **15**, 2301–2318.

[442] Hart, S. R., Blijsztajn, J., & Craddock, C., 1995. Cenozoic volcanism in Antarctica: Jones Mountains and Peter I Island, *Geochimica et Cosmochimica Acta*, **59**(16), 3379–3388.

[443] Hart, W. K., Woldegabriel, G., Walter, R. C., & Mertzman, S. A., 1989. Basaltic volcanism in Ethiopia: constraints on continental rifting and mantle interactions, *Journal of Geophysical Research*, **94**(B6), 7731–7748.

[444] Haskins, E. H. & Garcia, M. O., 2004. Scientific drilling reveals geochemical heterogeneity within the Ko'olau shield, Hawai'i, *Contributions to Mineralogy and Petrology*, **147**(2), 162–188.

[445] Hauri, E., 2002. SIMS analysis of volatiles in silicate glasses, 2: isotopes and abundances in Hawaiian melt inclusions, *Chemical Geology*, **183**, 115–141.

[446] Hauri, E. H. & Hart, S. R., 1994. Correction to "Constraints on melt migration from mantle plumes: A trace element study of peridotite xenoliths from Savai'i, Western Samoa" by Erik H. Hauri and Stanley R. Hart, *Journal of Geophysical Research: Solid Earth*, **99**(B12), 24301–24321.

[447] Hauri, E. H. & Hart, S. R., 1997. Rhenium abundances and systematics in oceanic basalts, *Chemical Geology*, **139**(1-4), 185–205.

[448] Hauri, E. H. & Kurz, M. D., 1997. Melt migration and mantle chromatography, 2: a time-series Os isotope study of Mauna Loa volcano, Hawaii, *Earth and Planetary Science Letters*, **153**(1-2), 21–36.

[449] Head, E. M., Shaw, A. M., Wallace, P. J., Sims, K. W., & Carn, S. A., 2011. Insight into volatile behavior at Nyamuragira volcano (DR Congo, Africa) through olivine-hosted melt inclusions, *Geochemistry, Geophysics, Geosystems*, **12**(10).

[450] Heimann, A., Steinitz, G., Mor, D., & Shaliv, G., 1996. The Cover Basalt Formation, its age and its regional and tectonic setting: implications from K–Ar and ^{40}Ar–^{39}Ar geochronology, *Israel Journal of Earth-Sciences*, **45**(5).

[451] Hekinian, R., Bideau, D., Stoffers, P., Cheminee, J. L., Muhe, R., Puteanus, D., & Binard, N., 1991. Submarine intraplate volcanism in the South Pacific: Geological setting and petrology of the Society and the Austral regions, *Journal of Geophysical Research: Solid Earth*, **96**(B2), 2109–2138.

[452] Hekinian, R., Cheminée, J., Dubois, J., Stoffers, P., Scott, S., Guivel, C., Garbe-Schönberg, D., Devey, C., Bourdon, B., Lackschewitz, K.,*et al.*, 2003. The Pitcairn hotspot in the South Pacific: distribution and composition of submarine volcanic sequences, *Journal of volcanology and geothermal research*, **121**(3-4), 219–245.

[453] Helz, R., 1980. Crystallization history of Kilauea Iki lava lake as seen in drill core recovered in 1967–1979, *Bulletin Volcanologique*, **43**(4), 675–701.

[454] Helz, R. T. & Thornber, C. R., 1987. Geothermometry of Kilauea Iki lava lake, Hawaii, *Bulletin of Volcanology*, **49**, 651–668.

[455] Hemond, C., Arndt, N. T., Lichtenstein, U., Hofmann, A. W., Oskarsson, N., & Steinthorsson, S., 1993. The heterogeneous Iceland plume: Nd-Sr-O isotopes and trace element constraints, *Journal of Geophysical Research*, **98**(B9), 15833.

[456] Hémond, C., Devey, C. W., & Chauvel, C., 1994. Source compositions and melting processes in the Society and Austral plumes (South Pacific Ocean): Element and isotope (Sr, Nd, Pb, Th) geochemistry, *Chemical Geology*, **115**(1-2), 7–45.

[457] Hertogen, J., Vanlerberghe, L., & Namegabe, M., 1985. Geochemical evolution of the Nyiragongo volcano (Virunga, Western African Rift, Zaire), *Bulletin of the Geological Society of Finland*, **57**(1-2), 21–35.

[458] Hicks, A., Barclay, J., Mark, D. F., & Loughlin, S., 2012. Tristan da Cunha: Constraining eruptive behavior using the 40Ar/39Ar dating technique, *Geology*, **40**(8), 723–726.

[459] Hildenbrand, A., Gillot, P. Y., & Le Roy, I., 2004. Volcano-tectonic and geochemical evolution of an oceanic intra-plate volcano: Tahiti-Nui (French Polynesia), *Earth and Planetary Science Letters*, **217**(3-4), 349–365.

[460] Hildenbrand, A., Madureira, P., Marques, F. O., Cruz, I., Henry, B., & Silva, P., 2008. Multi-stage evolution of a sub-aerial volcanic ridge over the last 1.3 Myr: S. Jorge Island, Azores Triple Junction, *Earth and Planetary Science Letters*, **273**(3-4), 289–298.

[461] Hildenbrand, A., Weis, D., Madureira, P., & Marques, F. O., 2014. Recent plate re-organization at the Azores Triple Junction: Evidence from combined geochemical and geochronological data on Faial, S. Jorge and Terceira volcanic islands, *Lithos*, **210**, 27–39.

[462] Hildner, E., Klügel, A., & Hansteen, T. H., 2012. Barometry of lavas from the 1951 eruption of Fogo, Cape Verde Islands: Implications for historic and prehistoric magma plumbing systems, *Journal of Volcanology and Geothermal Research*, **217**, 73–90.

[463] Hilton, D., Halldórsson, S., Barry, P., Fischer, T., de Moor, J., Ramirez, C., Mangasini, F., & Scarsi, P., 2011. Helium isotopes at Rungwe Volcanic Province, Tanzania, and the origin of East African plateaux, *Geophysical Research Letters*, **38**(21).

[464] Ho, K. S., Chen, J. C., & Juang, W. S., 2000. Geochronology and geochemistry of late Cenozoic basalts from the Leiqiong area, southern China, *Journal of Asian Earth Sciences*, **18**(3), 307–324.

[465] Ho, K. S., Chen, J. C., Lo, C. H., & Zhao, H. L., 2003. ^{40}Ar-^{39}Ar dating and geochemical characteristics of late Cenozoic basaltic rocks from the Zhejiang-Fujian region, SE China: Eruption ages, magma evolution and petrogenesis, *Chemical Geology*, **197**(1-4), 287–318.

[466] Ho, K. S., Liu, Y., Chen, J. C., & Yang, H. J., 2008. Elemental and Sr-Nd-Pb isotopic compositions of late Cenozoic Abaga basalts, Inner Mongolia: Implications for petrogenesis and mantle process, *Geochemical Journal*, **42**(4), 339–357.

[467] Ho, K. S., Ge, W. C., Chen, J. C., You, C. F., Yang, H. J., & Zhang, Y. L., 2013. Late Cenozoic magmatic transitions in the central Great Xing'an Range, Northeast China: Geochemical and isotopic constraints on petrogenesis, *Chemical Geology*, **352**, 1–18.

[468] Ho, T., Priestley, K., & Debayle, E., 2016. A global horizontal shear velocity model of the upper mantle from multimode Love wave measurements, *Geophysical journal international*, **207**(1), 542–561.

[469] Hoang, N., Flower, M. F., & Carlson, R. W., 1996. Major, trace element, and isotopic compositions of Vietnamese basalts: interaction of hydrous EM1-rich asthenosphere with thinned Eurasian lithosphere, *Geochimica et cosmochimica Acta*, **60**(22), 4329–4351.

[470] Hoàng, N., Flower, M. F., Xuân, P. T., Quỳ, H. V., & Son, T. T., 2013. Collision-induced basalt eruptions at Pleiku and Buôn Mê Thuot, south-central Viet Nam, *Journal of Geodynamics*, **69**, 65–83.

[471] Hoang, T. H. A., Choi, S. H., Yu, Y., Pham, T. H., Nguyen, K. H., & Ryu, J.-S., 2018. Geochemical constraints on the spatial distribution of recycled oceanic crust in the mantle source of late Cenozoic basalts, Vietnam, *Lithos*, **296**, 382–395.

[472] Hoernle, K. & Schmincke, H. U., 1993. The role of partial melting in the 15-MA geochemical evolution of gran canaria: A blob model for the Canary hotspot, *Journal of Petrology*, **34**(3), 599–626.

[473] Hoernle, K., White, J. D., van den Bogaard, P., Hauff, F., Coombs, D. S., Werner, R., Timm, C., Garbe-Schönberg, D., Reay, A., & Cooper, A. F., 2006. Cenozoic intraplate volcanism on New Zealand: Upwelling induced by lithospheric removal, *Earth and Planetary Science Letters*, **248**(1-2), 350–367.

[474] Hofmann, C., Courtillot, V., Feraud, G., Rochette, P., Yirgu, G., Ketefo, E., & Pik, R., 1997. Timing of the Ethiopian flood basalt event and implications for plume birth and global change, *Nature*, **389**(6653), 838.

[475] Hoggard, M. J., Czarnota, K., Richards, F. D., Huston, D. L., Jaques, A. L., & Ghelichkhan, S., 2020. Global distribution of sediment-hosted metals controlled by craton edge stability, *Nature Geoscience*, **13**(7), 504–510.

[476] Hole, M. J., Kempton, P. D., & Millar, I. L., 1993. Trace-element and isotopic characteristics of small-degree melts of the asthenosphere: Evidence from the alkalic basalts of the Antarctic Peninsula, *Chemical Geology*, **109**(1-4), 51–68.

[477] Holm, P. M., Wilson, J. R., Christensen, B. P., Hansen, L., Hansen, S. L., Hein, K. M., Mortensen, A. K., Pedersen, R., Plesner, S., & Runge, M. K., 2005. Sampling the Cape Verde mantle plume: evolution of melt compositions on Santo Antão, Cape Verde Islands, *Journal of Petrology*, **47**(1), 145–189.

[478] Hsu, C. N. & Chen, J. C., 1998. Geochemistry of late Cenozoic basalts from Wudalianchi and Jingpohu areas, Heilongjiang Province, northeast China, *Journal of Asian Earth Sciences*, **16**(4), 385–405.

[479] Hsu, C. N., Chen, J. C., & Ho, K. S., 2000. Geochemistry of cenozoic volcanic rocks from Kirin Province, northeast China, *Geochemical Journal*, **34**(1), 33–58.

[480] Huang, F., Chen, J.-L., Xu, J.-F., Wang, B.-D., & Li, J., 2015. Os–Nd–Sr isotopes in Miocene ultrapotassic rocks of southern Tibet: Partial melting of a pyroxenite-bearing lithospheric mantle?, *Geochimica et Cosmochimica Acta*, **163**, 279–298.

[481] Huang, X. L., Niu, Y., Xu, Y. G., Ma, J. L., Qiu, H. N., & Zhong, J. W., 2013. Geochronology and geochemistry of Cenozoic basalts from eastern Guangdong, SE China: Constraints on the lithosphere evolution beneath the northern margin of the South China Sea, *Contributions to Mineralogy and Petrology*, **165**(3), 437–455.

[482] Hudgins, T. R., Mukasa, S. B., Simon, A. C., Moore, G., & Barifaijo, E., 2015. Melt inclusion evidence for CO2-rich melts beneath the western branch of the East African Rift: implications for long-term storage of volatiles in the deep lithospheric mantle, *Contributions to Mineralogy and Petrology*, **169**(5).

[483] Hungerford, J. D. G., Edwards, B. R., Skilling, I. P., & Cameron, B. I., 2014. Evolution of a subglacial basaltic lava flow field: Tennena volcanic center, Mount Edziza volcanic complex, British Columbia, Canada, *Journal of Volcanology and Geothermal Research*, **272**, 39–58.

[484] Hunt, A. C., Parkinson, I. J., Harris, N. B., Barry, T. L., Rogers, N. W., & Yondon, M., 2012. Cenozoic volcanism on the Hangai Dome, Central Mongolia: Geochemical evidence for changing melt sources and implications for mechanisms of melting, *Journal of Petrology*, **53**(9), 1913–1942.

[485] Hutchison, W., Pyle, D. M., Mather, T. A., Yirgu, G., Biggs, J., Cohen, B. E., Barfod, D. N., & Lewi, E., 2016. The eruptive history and magmatic evolution of Aluto volcano: new insights into silicic peralkaline volcanism in the Ethiopian rift, *Journal of Volcanology and Geothermal Research*, **328**, 9–33.

[486] Ibrahim, K. M., Tarawneh, K., & Rabba', I., 2003. Phases of activity and geochemistry of basaltic dike systems in northeast Jordan parallel to the Red Sea, *Journal of Asian Earth Sciences*, **21**(5), 467–472.

[487] Ibrahim, K. M., Shaw, J., Baker, J., Khoury, H., Rabba, I., & Tarawneh, K., 2006. Pliocene-Pleistocene volcanism in northwestern Arabian plate (Jordan): I. Geology and geochemistry of the Asfar Volcanic Group, *Neues Jahrbuch für Geologie und Paläontologie - Abhandlungen*, **242**(2-3), 145–170.

[488] Ielsch, G., Caroff, M., Barsczus, H. G., Maury, R. C., Guillou, H., Guille, G., & Cotten, J., 1998. Geochemistry of Ua Huka basalts (Marquesas): partial melting variations and mantle source heterogeneity, *Earth and Planetary Science Letters*, **326**, 413–420.

[489] Ilani, S., Harlavan, Y., Tarawneh, K., Rabba, I., Weinberger, R., Ibrahim, K. M., Peltz, S., & Steinitz, G., 2001. New K-Ar ages of basalts from the Harrat Ash Shaam volcanic field in Jordan: Implications for the span and duration of the upper-mantle upwelling beneath the western Arabian plate, *Geology*, **29**(2), 171–174.

[490] Ionov, D. A., Ashchepkov, I. V., Stosch, H.-G., Witt-Eickschen, G., & Seck, H. A., 1993. Garnet Peridotite Xenoliths from the Vitim Volcanic Field, Baikal Region: the Nature of the Garnet–Spinel Peridotite Transition Zone in the Continental Mantle, *Journal of Petrology*, **34**(6), 1141–1175.

[491] Irving, A. & Menzies, M., 1991. Isotopic evidence for variably enriched MORB lithospheric mantle in xenoliths from North Queensland, Australia, in *International Kimberlite Conference: Extended Abstracts*, vol. 5, pp. 186–187.

[492] Ivanov, A. V., Arzhannikov, S. G., Demonterova, E. I., Arzhannikova, A. V., & Orlova, L. A., 2011. Jom-Bolok Holocene volcanic field in the East Sayan Mts., Siberia, Russia: Structure, style of eruptions, magma compositions, and radiocarbon dating, *Bulletin of Volcanology*, **73**(9), 1279–1294.

[493] Ivanov, A. V., Demonterova, E. I., He, H., Perepelov, A. B., Travin, A. V., & Lebedev, V. A., 2015. Volcanism in the Baikal rift: 40 years of active-versus-passive model discussion, *Earth-Science Reviews*, **148**, 18–43.

[494] Iverson, N. A., Kyle, P. R., Dunbar, N. W., McIntosh, W. C., & Pearce, N. J. G., 2014. Geochemistry, Geophysics, Geosystems, *Geochemistry, Geophysics, Geosystems*, **15**, 4180–4202.

[495] Jackson, M. C., Frey, F. A., Garcia, M. O., & Wilmoth, R. A., 1999. Geology and geochemistry of basaltic lava flows and dikes from the Trans-Koolau tunnel, Oahu, Hawaii, *Bulletin of Volcanology*, **60**(5), 381–401.

[496] Jackson, M. G., Hart, S. R., Koppers, A. A., Staudigel, H., Konter, J., Blusztajn, J., Kurz, M., & Russell, J. A., 2007. The return of subducted continental crust in Samoan lavas, *Nature*, **448**(7154), 684–687.

[497] Jackson, M. G., Hart, S. R., Konter, J. G., Koppers, A. A., Staudigel, H., Kurz, M. D., Blusztajn, J., & Sinton, J. M., 2010. Samoan hot spot track on a "hot spot highway": Implications for mantle plumes and a deep Samoan mantle source, *Geochemistry, Geophysics, Geosystems*, **11**(12).

[498] Jackson, M. G., Price, A. A., Blichert-Toft, J., Kurz, M. D., & Reinhard, A. A., 2017. Geochemistry of lavas from the Caroline hotspot, Micronesia: Evidence for primitive and recycled components in the mantle sources of lavas with moderately elevated $^{3}He/^{4}He$, *Chemical Geology*, **455**, 385–400.

[499] Johansen, T. S., Hauff, F., Hoernle, K., Klügel, A., & Kokfelt, T. F., 2005. Basanite to phonolite differentiation within 1550-1750 yr: U-Th-Ra isotopic evidence from the A.D. 1585 eruption on La Palma, Canary Islands, *Geology*, **33**(11), 897–900.

[500] Johnson, J. S., Gibson, S. A., Thompson, R. N., & Nowell, G. M., 2005. Volcanism in the Vitim Volcanic Field, Siberia: Geochemical evidence for a mantle plume beneath the Baikal Rift zone, *Journal of Petrology*, **46**(7), 1309–1344.

[501] Johnson, K., Graham, D., Rubin, K., Nicolaysen, K., Scheirer, D., Forsyth, D., Baker, E., & Douglas-Priebe, L., 2000. Boomerang Seamount: The active expression of the Amsterdam–St. Paul hotspot, Southeast Indian Ridge, *Earth and Planetary Science Letters*, **183**(1-2), 245–259.

[502] Johnson, K. T., Sinton, J. M., & Price, R. C., 1986. Petrology of seamounts northwest of Samoa and their relation to Samoan volcanism, *Bulletin of volcanology*, **48**(4), 225–235.

[503] Jónasson, K., 2005. Magmatic evolution of the Heidarspordur ridge, NE-Iceland, *Journal of volcanology and geothermal research*, **147**(1-2), 109–124.

[504] Jordan, S. C., Jowitt, S. M., & Cas, R. A. F., 2015. Origin of temporal-compositional variations during the eruption of Lake Purrumbete Maar, Newer Volcanics Province, southeastern Australia, *Bulletin of Volcanology*, **77**(1), 883.

[505] Jørgensen, J. Ø. & Holm, P. M., 2002. Temporal variation and carbonatite contamination in primitive ocean island volcanics from Sao Vicente, Cape Verde Islands, *Chemical Geology*, **192**(3-4), 249–267.

[506] Juang, W.-S., 1996. Geochronology and geochemistry of basalts in the western foothills, Taiwan, *Bulletin of National Museum of Natural Science*, **7**, 45–98.

[507] Jung, S., 1995. Geochemistry and petrogenesis of rift-related Tertiary alkaline rocks from the Rhon area (central Germany), *Neues Jahrbuch fur Mineralogie, Abhandlungen*, **169**(3), 193–226.

[508] Jung, S. & Hoernes, S., 2000. The major-and trace-element and isotope (Sr, Nd, O) geochemistry of Cenozoic alkaline rift-type volcanic rocks from the Rhön area (central Germany): petrology, mantle source characteristics and implications for asthenosphere–lithosphere interactions, *Journal of Volcanology and Geothermal Research*, **99**(1-4), 27–53.

[509] Jung, S. & Masberg, P., 1998. Major-and trace-element systematics and isotope geochemistry of Cenozoic mafic volcanic rocks from the Vogelsberg (central Germany): constraints on the origin of continental alkaline and tholeiitic basalts and their mantle sources, *Journal of Volcanology and Geothermal Research*, **86**(1-4), 151–177.

[510] Jung, S., Pfänder, J. A., Brügmann, G., & Stracke, A., 2005. Sources of primitive alkaline volcanic rocks from the Central European Volcanic Province (Rhön, Germany) inferred from Hf, Os and Pb isotopes, *Contributions to Mineralogy and Petrology*, **150**(5), 546–559.

[511] Jung, S., Vieten, K., Romer, R. L., Mezger, K., Hoernes, S., & Satir, M., 2012. Petrogenesis of Tertiary alkaline magmas in the Siebengebirge, Germany, *Journal of Petrology*, **53**(11), 2381–2409.

[512] Kabeto, K., Sawada, Y., Iizumi, S., & Wakatsuki, T., 2001. Mantle sources and magma-crust interactions in volcanic rocks from the northern Kenya rift: Geochemical evidence, *Lithos*, **56**(2-3), 111–139.

[513] Kabeto, K., Sawada, Y., & Wakatsuki, T., 2001. Different evolution trends in alkaline evolved lavas from the Northern Kenya Rift, *Journal of African Earth Sciences*, **32**(3), 419–433.

[514] Kaczmarek, M.-A., Bodinier, J.-L., Bosch, D., Tommasi, A., Dautria, J.-M., & Kechid, S., 2016. Metasomatized Mantle Xenoliths as a Record of the Lithospheric Mantle Evolution of the Northern Edge of the Ahaggar Swell, In Teria (Algeria), *Journal of Petrology*, **57**(2), 345–382.

[515] Kagou Dongmo, A., Nkouathio, D., Pouclet, A., Bardintzeff, J. M., Wandji, P., Nono, A., & Guillou, H., 2010. The discovery of late Quaternary basalt on Mount Bambouto: Implications for recent widespread volcanic activity in the southern Cameroon Line, *Journal of African Earth Sciences*, **57**(1-2), 96–108.

[516] Kalfoun, F., 2001. *Géochimie du Niobium du Tantale: distribution et fractionnement de ces deux éléments dans les différents réservoirs terrestres*, Ph.D. thesis, L'Universite Montpellier II.

[517] Kamgang, P., Chazot, G., Njonfang, E., Ngongang, N. B. T., & Tchoua, F. M., 2013. Mantle sources and magma evolution beneath the Cameroon Volcanic Line: Geochemistry of mafic rocks from the Bamenda Mountains (NW Cameroon), *Gondwana Research*, **24**(2), 727–741.

[518] Kampunzu, A. B., Sebagenzi, M. N., Caron, J. P.-H., & Vellutini, P. J., 1982. Petrochemical comparison of the lavas of the southern Kivu field (Bukavu) and northern Kivu field (Virunga), Zaire, *ANN. SOC. GEOL. BELGIQUE*, **105**, 25–29.

[519] Kampunzu, A. B., Kanika, M., Caron, J. P.-H., Lubala, R. T., & J, V. P., 1984. The transitional basalts of the continental rift: an examples from Haute-Ruzizi in the Central African Rift (Kivu-Zaire), *Geologische Rundschau*, **73**, 895–916.

[520] Kampunzu, A. B., Bonhomme, M. G., & Kanika, M., 1998. Geochronology of volcanic rocks and evolution of the Cenozoic western branch of the East African Rift system, *Journal of African Earth Sciences*, **26**(3), 441–461.

[521] Katz, R. F., Spiegelmann, M., & Langmuir, C. H., 2003. A new parameterization of hydrous mantle melting, *Geochemistry, Geophysics, Geosystems*, **4**(9).

[522] Kauahikaua, J., Cashman, K., Clague, D., Champion, D., & Hagstrum, J., 2002. Emplacement of the most recent lava flows on Hualālai Volcano, Hawai'i, *Bulletin of Volcanology*, **64**(3-4), 229–253.

[523] Kawabata, H., Hanyu, T., Chang, Q., Kimura, J. I., Nichols, A. R., & Tatsumi, Y., 2011. The petrology and geochemistry of St. Helena alkali basalts: Evaluation of the oceanic crust-recycling model for HIMU OIB, *Journal of Petrology*, **52**(4), 791–838.

[524] Keller, J., Zaitsev, A. N., & Wiedenmann, D., 2006. Primary magmas at Oldoinyo Lengai: the role of olivine melilitites, *Lithos*, **91**(1-4), 150–172.

[525] Kempton, P. D. Y., Fitton, J. G., Saunders, A. D., Nowell, G. M., Taylor, R. N., Hardarson, B. S., & Pearson, G., 2000. The Iceland plume in space and time: a Sr-Nd-Pb-Hf study of the North Atlantic rifted margin, *Earth and Planetary Science Letters*, **177**, 255–271.

[526] Keskin, M., Pearce, J. A., & Mitchell, J. G., 1998. Volcano-stratigraphy and geochemistry of collision-related volcanism on the Erzurum-Kars Plateua, northeastern Turkey, *Journal of Volcanology and Geothermal Research*, **85**, 355–404.

[527] Kheirkhah, M., Allen, M. B., & Emami, M., 2009. Quaternary syn-collision magmatism from the Iran/Turkey borderlands, *Journal of Volcanology and Geothermal Research*, **182**, 1–12.

[528] Kieffer, B., Arndt, N., Lapierre, H., Bastien, F., Bosch, D., Pecher, A., Yirgu, G., Ayalew, D., Weis, D., Jerram, D. A.,*et al.*, 2004. Flood and shield basalts from Ethiopia: magmas from the African superswell, *Journal of Petrology*, **45**(4), 793–834.

[529] Kipf, A., Hauff, F., Werner, R., Gohl, K., van den Bogaard, P., Hoernle, K., Maicher, D., & Klügel, A., 2014. Seamounts off the West Antarctic margin: A case for non-hotspot driven intraplate volcanism, *Gondwana Research*, **25**(4), 1660–1679.

[530] Kitagawa, H., Kobayashi, K., Makishima, A., & Nakamura, E., 2008. Multiple pulses of the mantle plume: Evidence from Tertiary icelandic lavas, *Journal of Petrology*, **49**(7), 1365–1396.

[531] Klöcking, M., 2018. *Continental magmatism and dynamic topography*, Ph.D. thesis, University of Cambridge.

[532] Klöcking, M., White, N. J., Maclennan, J., McKenzie, D., & Fitton, J. G., 2018. Quantitative relationships between basalt geochemistry, shear wave velocity, and asthenospheric temperature beneath western North America, *Geochemistry, Geophysics, Geosystems*, **19**, 3376–3404.

[533] Klöcking, M., Hoggard, M., Tribaldos, V. R., Richards, F., Guimarães, A., Maclennan, J., & White, N., 2020. A tale of two domes: Neogene to recent volcanism and dynamic uplift of northeast Brazil and southwest Africa, *Earth and Planetary Science Letters*, **547**, 116464.

[534] Klügel, A., Hoernle, K. A., & Schmincke, H.-U., 2000. The chemically zoned 1949 eruption on La Palma (Canary Islands): Petrologic evolution and magma supply dynamics of a rift zone eruption, *Journal of Geophysical Research*, **105**, 5997–6016.

[535] Klügel, A., Galipp, K., Hoernle, K., Hauff, F., & Groom, S., 2017. Geochemical and volcanological evolution of la palma, Canary Islands, *Journal of Petrology*, **58**(6), 1227–1248.

[536] Knesel, K. M., Souza, Z. S., Vasconcelos, P. M., Cohen, B. E., & Silveira, F. V., 2011. Young volcanism in the Borborema Province, NE Brazil, shows no evidence for a trace of the Fernando de Noronha plume on the continent, *Earth and Planetary Science Letters*, **302**(1-2), 38–50.

[537] Knutson, J., N.D. Whole-rock geochemical data from Eastern Australia's Cenozoic Volcanic Provinces, Tech. rep., Geoscience Australia.

[538] Kogarko, L., Levskii, L., & Gushchina, N., 2003. Isotope sources of hot spots in the Trindade and Martin Vaz Islands, southwestern Atlantic, in *Doklady Earth Sciences*, vol. 393, pp. 1116–1119, Pleiades Publishing, Ltd, Rhode Town.

[539] Kogiso, T., Tatsumi, Y., Shimoda, G., & Barsczus, H. G., 1997. High μ (HIMU) ocean island basalts in southern Polynesia: new evidence for whole mantle scale recycling of subducted oceanic crust, *Journal of Geophysical Research*, **102**(96), 8085–8103.

[540] Koh, J. S., Yun, S.-H., Hyeon, G. B., Lee, M. W., & Gil, Y.-W., 2005. Petrology of the basalt in the Udo monogenetic volcano, Jeju Island, *Journal of the Petrological Society of Korea*, **14**(1), 45–60.

[541] Kokfelt, T. F., Hoernle, K., Hauff, F., Fiebig, J., Werner, R., & Garbe-Schönberg, D., 2006. Combined trace element and Pb-Nd-Sr-O isotope evidence for recycled oceanic crust (upper and lower) in the Iceland mantle plume, *Journal of Petrology*, **47**(9), 1705–1749.

[542] Kokfelt, T. F., Hoernle, K., Lundstrom, C., Hauff, F., & van den Bogaard, C., 2009. Time-scales for magmatic differentiation at the Snaefellsjökull central volcano, western Iceland: Constraints from U-Th-Pa-Ra disequilibria in post-glacial lavas, *Geochimica et Cosmochimica Acta*, **73**(4), 1120–1144.

[543] Kolb, M., Paulick, H., Kirchenbaur, M., & Münker, C., 2012. Petrogenesis of mafic to felsic lavas from the oligocene siebengebirge volcanic field (Germany): Implications for the origin of intracontinental volcanism in central Europe, *Journal of Petrology*, **53**(11), 2349–2379.

[544] Konter, J., Staudigel, H., Blichert-Toft, J., Hanan, B., Polvé, M., Davies, G., Shimizu, N., & Schiffman, P., 2009. Geochemical stages at Jasper Seamount and the origin of intraplate volcanoes, *Geochemistry, Geophysics, Geosystems*, **10**(2).

[545] Konter, J. G. & Jackson, M. G., 2012. Large volumes of rejuvenated volcanism in Samoa: Evidence supporting a tectonic influence on late-stage volcanism, *Geochemistry, Geophysics, Geosystems*, **13**(1).

[546] Konter, J. G., Pietruszka, A. J., Hanan, B. B., Finlayson, V. A., Craddock, P. R., Jackson, M. G., & Dauphas, N., 2016. Unusual δ^{56}Fe values in Samoan rejuvenated lavas generated in the mantle, *Earth and Planetary Science Letters*, **450**, 221–232.

[547] Koornneef, J. M., Stracke, A., Bourdon, B., Meier, M. A., Jochum, K. P., Stoll, B., & Grönvold, K., 2012. Melting of a two-component source beneath Iceland, *Journal of Petrology*, **53**(1), 127–157.

[548] Koyaguchi, T., 1984. Volcanic rocks in the Samburu Hills, northern Kenya, *African Study Monographs, Supplementary Issue*, **2**, 147–179.

[549] Krienitz, M. S., Haase, K. M., Mezger, K., Eckardt, V., & Shaikh-Mashail, M. A., 2006. Magma genesis and crustal contamination of continental intraplate lavas in northwestern Syria, *Contributions to Mineralogy and Petrology*, **151**(6), 698–716.

[550] Krienitz, M. S., Haase, K. M., Mezger, K., & Shaikh-Mashail, M. A., 2007. Magma genesis and mantle dynamics at the Harrat Ash Shamah volcanic field (southern Syria), *Journal of Petrology*, **48**(8), 1513–1542.

[551] Krienitz, M.-S., Haase, K., Mezger, K., van den Bogaard, P., Thiemann, V., & Shaikh-Mashail, M., 2009. Tectonic events, continental intraplate volcanism, and mantle plume activity in northern Arabia: Constraints from geochemistry and Ar-Ar dating of Syrian lavas, *Geochemistry, Geophysics, Geosystems*, **10**(4).

[552] Krienitz, M.-S., Garbe-Schönberg, C.-D., Romer, R., Meixner, A., Haase, K., & Stroncik, N. A., 2012. Lithium isotope variations in ocean island basalts – implications for the development of mantle heterogeneity, *Journal of Petrology*, **53**(11), 2333–2347.

[553] Kröchert, J. & Buchner, E., 2009. Age distribution of cinder cones within the Bandas del Sur Formation, southern Tenerife, Canary Islands, *Geological Magazine*, **146**(2), 161–172.

[554] Kuehn, C., Guest, B., Russell, J. K., & Benowitz, J. A., 2015. The Satah Mountain and Baldface Mountain volcanic fields: Pleistocene hot spot volcanism in the Anahim Volcanic Belt, west-central British Columbia, Canada, *Bulletin of Volcanology*, **77**(3).

[555] Kuepouo, G., Tchouankoue, J. P., Nagao, T., & Sato, H., 2006. Transitional tholeiitic basalts in the Tertiary Bana volcano-plutonic complex, Cameroon Line, *Journal of African Earth Sciences*, **45**(3), 318–332.

[556] Kuritani, T., Kimura, J. I., Miyamoto, T., Wei, H., Shimano, T., Maeno, F., Jin, X., & Taniguchi, H., 2009. Intraplate magmatism related to deceleration of upwelling asthenospheric mantle: Implications from the Changbaishan shield basalts, northeast China, *Lithos*, **112**(3-4), 247–258.

[557] Kuritani, T., Yokoyama, T., Kitagawa, H., Kobayashi, K., & Nakamura, E., 2011. Geochemical evolution of historical lavas from Askja Volcano, Iceland: Implications for mechanisms and timescales of magmatic differentiation, *Geochimica et Cosmochimica Acta*, **75**(2), 570–587.

[558] Kuritani, T., Kimura, J. I., Ohtani, E., Miyamoto, H., & Furuyama, K., 2013. Transition zone origin of potassic basalts from Wudalianchi volcano, northeast China, *Lithos*, **156-159**, 1–12.

[559] Kürkçüoğlu, B., Pickard, M., Şen, P., Hanan, B. B., Sayit, K., Plummer, C., Sen, E., Yurur, T., & Furman, T., 2015. Geochemistry of mafic lavas from Sivas, Turkey and the evolution of Anatolian lithosphere, *Lithos*, **232**, 229–241.

[560] Kürüm, S., Önal, A., Boztuğ, D., Spell, T., & Arslan, M., 2008. $^{40}Ar/^{39}Ar$ age and geochemistry of the post-collisional Miocene Yamadağ volcanics in the Arapkir area (Malatya Province), eastern Anatolia, Turkey, *Journal of Asian Earth Science*, **33**, 229–251.

[561] Kurz, M. D. & Geist, D., 1999. Dynamics of the Galapagos hotspot from helium isotope geochemistry, *Geochimica et Cosmochimica Acta*, **63**(23-24), 4139–4156.

[562] Lanphere, M. A. & Frey, F. A., 1987. Geochemical evolution of Kohala Volcano, Hawaii, *Contributions to Mineralogy and Petrology*, **95**(1), 100–113.

[563] Lara, L. E., Reyes, J., Jicha, B. R., & Díaz-Naveas, J., 2018. $^{40}Ar/^{39}Ar$ Geochronological Constraints on the Age Progression Along the Juan Fernández Ridge, SE Pacific, *Frontiers in Earth Science*, **6**, 194.

[564] Larrea, P., França, Z., Lago, M., Widom, E., Galé, C., & Ubide, T., 2012. Magmatic processes and the role of antecrysts in the genesis of Corvo Island (Azores Archipelago, Portugal), *Journal of Petrology*, **54**(4), 769–793.

[565] Larrea, P., Galé, C., Ubide, T., Widom, E., Lago, M., & França, Z., 2014. Magmatic evolution of Graciosa (Azores, Portugal), *Journal of Petrology*, **55**(11), 2125–2154.

[566] Larsson, D., Grönvold, K., Oskarsson, N., & Gunnlaugsson, E., 2002. Hydrothermal alteration of plagioclase and growth of secondary feldspar in the Hengill Volcanic Centre, SW Iceland, *Journal of volcanology and geothermal research*, **114**(3-4), 275–290.

[567] Laske, G., Masters, G., Ma, Z., & Pasyanos, M., 2013. Update on CRUST1.0 – A 1-degree global model of Earth's crust, in *Geophys. Res. Abstr*, vol. 15, p. 2658.

[568] Lassiter, J. C., Blichert-Toft, J., Hauri, E. H., & Barsczus, H. G., 2003. Isotope and trace element variations in lavas from Raivavae and Rapa, Cook-Austral islands: Constraints on the nature of HIMU- and EM-mantle and the origin of mid-plate volcanism in French Polynesia, *Chemical Geology*, **202**(1-2), 115–138.

[569] Le Bas, M., 1987. Nephelinites and carbonatites, *Geological Society, London, Special Publications*, **30**(1), 53–83.

[570] Le Dez, A., Maury, R. C., Vidal, P., Bellon, H., Cotten, J., & Brousse, R., 1996. Geology and geochemistry of Nuku Hiva, Marquesas: temporal trends in a large Polynesian shield volcano, *Oceanographic Literature Review*, **10**(43), 1013.

[571] Le Roex, A. P., Cliff, R. A., & Adair, B. J., 1990. Tristan da Cunha, South Atlantic: Geochemistry and petrogenesis of a basanite-phonolite lava series, *Journal of Petrology*, **31**(4), 779–812.

[572] le Roex, A. P., Chevallier, L., Verwoerd, W. J., & Barends, R., 2012. Petrology and geochemistry of Marion and Prince Edward Islands, Southern Ocean: Magma chamber processes and source region characteristics, *Journal of Volcanology and Geothermal Research*, **223-224**, 11–28.

[573] Lease, N. A. & Abdel-Rahman, A. F. M., 2008. The Euphrates volcanic field, northeastern Syria: Petrogenesis of Cenozoic basanites and alkali basalts, *Geological Magazine*, **145**(5), 685–701.

[574] Lebedev, V., Sharkov, E., Ünal, E., & Keskin, M., 2016. Late pleistocene Tendürek volcano (Eastern Anatolia, Turkey): I. Geochronology and petrographic characteristics of igneous rocks, *Petrology*, **24**(2), 127–152.

[575] Lebedev, V. A., Bubnov, S. N., Chernyshev, I. V., Chugaev, A. V., Dudauri, O. Z., & Vashakidze, G. T., 2007. Geochronology and Genesis of Subalkaline Basaltic Lava Rivers at the Dzhavakheti Highland, Lesser Caucasus: K–Ar and Sr–Nd Isotopic Data, *Geochemistry International*, **45**(3), 211–225.

[576] Lebedev, V. A., Bubnov, S. N., Dudauri, O. Z., & Vashakidze, G. T., 2008. Geochronology of Pliocene volcanism in the Dzhavakheti Highland (the Lesser Caucasus). Part 1: Western part of the Dzhavakheti Highland, *Stratigraphy and Geological Correlation*, **16**(2), 204–224.

[577] Lebedev, V. A., Bubnov, S. N., Dudauri, O. Z., & Vashakidze, G. T., 2008. Geochronology of Pliocene volcanism in the Dzhavakheti Highland (the Lesser Caucasus). Part 1: Eastern part of the Dzhavakheti Highland. Regional geological correlation, *Stratigraphy and Geological Correlation*, **16**(5), 553–574.

[578] Lechmann, A., Burg, J.-P., Ulmer, P., Guillong, M., & Faridi, M., 2018. Metasomatized mantle as the source of Mid-Miocene-Quaternary volcanism in NW-Iranian Azerbaijan: Geochronological and geochemical evidence, *Lithos*, **304**, 311–328.

[579] Lee, D. C., Halliday, A. N., Fitton, J. G., & Poli, G., 1994. Isotopic variations with distance and time in the volcanic islands of the Cameroon line: evidence for a mantle plume origin, *Earth and Planetary Science Letters*, **123**(1-3), 119–138.

[580] Lee, M. J., Lee, J. I., Kim, T. H., Lee, J., & Nagao, K., 2015. Age, geochemistry and Sr-Nd-Pb isotopic compositions of alkali volcanic rocks from Mt. Melbourne and the western Ross Sea, Antarctica, *Geosciences Journal*, **19**(4), 681–695.

[581] Leeman, W. P., Gerlach, D. C., Garcia, M. O., & West, H. B., 1994. Geochemical variations in lavas from Kahoolawe volcano, Hawaii: evidence for open system evolution of plume-derived magmas, *Contributions to Mineralogy and Petrology*, **116**(1-2), 62–77.

[582] Legendre, C., Caroff, M., Leyrit, H., Nehlig, P., & Thièblemont, D., 2001. Les premières phases d ' édification du stratovolcan du géologie et géochimie du secteur de l ' Élancèze, *Sciences-New York*, **332**, 617–624.

[583] Legendre, C., Maury, R. C., Guillou, H., Cotten, J., Caroff, M., Blais, S., & Guille, G., 2003. Evolution géologique et pétrologique de l'île de Huahine (archipel de la Société, Polynésie française) : Un volcan-bouclier intraocéanique original, *Bulletin de la Societe Geologique de France*, **174**(2), 115–124.

[584] Legendre, C., Maury, R. C., Caroff, M., Guillou, H., Cotten, J., Chauvel, C., Bollinger, C., Hémond, C., Guille, G., Blais, S., Rossi, P., & Savanier, D., 2005. Origin of exceptionally abundant phonolites on Ua Pou Island (Marquesas, French Polynesia): Partial melting of basanites followed by crustal contamination, *Journal of Petrology*, **46**(9), 1925–1962.

[585] Legendre, C., Maury, R. C., Savanier, D., Cotten, J., Chauvel, C., Hémond, C., Bollinger, C., Guille, G., Blais, S., & Rossi, P., 2005. The origin of intermediate and evolved lavas in the Marquesas archipelago: An example from Nuku Hiva island (French Polynesia), *Journal of Volcanology and Geothermal Research*, **143**(4), 293–317.

[586] Lemarchand, F., 1987. The volcanic series of Fayal (The Azores) - petrological and geochemical study, *Canadian Journal of Earth Sciences*, **24**(2), 334–353.

[587] LeMasurier, W. E., Thomson, J. W., Baker, P., Kyle, P., Rowley, P., Smellie, J., & Verwoerd, W., 1990. *Volcanoes of the Antarctic Plate and Southern Ocean*, vol. 48, American Geophysical Union, Washington D.C., U.S.A.

[588] LeMasurier, W. E., Choi, S. H., Kawachi, Y., Mukasa, S. B., & Rogers, N. W., 2011. Evolution of pantellerite-trachyte-phonolite volcanoes by fractional crystallization of basanite magma in a continental rift setting, Marie Byrd Land, Antarctica, *Contributions to Mineralogy and Petrology*, **162**(6), 1175–1199.

[589] LeMasurier, W. E., Choi, S. H., Hart, S. R., Mukasa, S., & Rogers, N., 2016. Reconciling the shadow of a subduction signature with rift geochemistry and tectonic environment in Eastern Marie Byrd Land, Antarctica, *Lithos*, **260**, 134–153.

[590] Lénat, J. F., Boivin, P., Deniel, C., Gillot, P. Y., & Bachèlery, P., 2009. Age and nature of deposits on the submarine flanks of Piton de la Fournaise (Reunion Island), *Journal of Volcanology and Geothermal Research*, **184**(1-2), 199–207.

[591] Lenoir, X., Garrido, C. J., Bodinier, J. L., & Dautria, J. M., 2000. Contrasting lithospheric mantle domains beneath the Massif Central (France) revealed by geochemistry of peridotite xenoliths, *Earth and Planetary Science Letters*, **181**(3), 359–375.

[592] Li, H. Y., Huang, X. L., & Guo, H., 2014. Geochemistry of Cenozoic basalts from the Bohai Bay Basin: Implications for a heterogeneous mantle source and lithospheric evolution beneath the eastern North China Craton, *Lithos*, **196-197**, 54–66.

[593] Li, Y. Q., Ma, C. Q., Robinson, P. T., Zhou, Q., & Liu, M. L., 2015. Recycling of oceanic crust from a stagnant slab in the mantle transition zone: Evidence from Cenozoic continental basalts in Zhejiang Province, SE China, *Lithos*, **230**, 146–165.

[594] Li, Y. Q., Ma, C. Q., & Robinson, P. T., 2016. Petrology and geochemistry of Cenozoic intra-plate basalts in east-central China: Constraints on recycling of an oceanic slab in the source region, *Lithos*, **262**, 27–43.

[595] Liotard, J., Barsczus, H. G., Dupuy, C., & Dostal, J., 1986. Geochemistry and origin of basaltic lavas from Marquesas Archipelago, French Polynesia, *Contributions to Mineralogy and Petrology*, **92**(2), 260–268.

[596] Liotard, J. M., Briot, D., & Boivin, P., 1988. Petrological and geochemical relationships between pyroxene megacrysts and associated alkali-basalts from Massif Central (France), *Contributions to Mineralogy and Petrology*, **98**(1), 81–90.

[597] Liotard, J.-M., Dautria, J.-M., & Cantagrel, J.-M., 1995. Pétrogenèse des néphélinites de la région de Craponne-sur-Arzon (Nord-Velay): intervention possible d'un composant carbonatitique, *Comptes rendus de l'Académie des sciences. Série 2. Sciences de la terre et des planètes*, **320**(11), 1043–1050.

[598] Liotard, J.-M., Briqueu, L., Dautria, J.-M., & Jakni, B., 1999. Basanites and nephelinites from Bas-Languedoc: mantllic heterogeneities and crustal contamination, *Bulletin de la Societe Geologique de France*, **170**(4), 423–433.

[599] Liotard, J. M., Dautria, J. M., Bosch, D., Condomines, M., Mehdizadeh, H., & Ritz, J. F., 2008. Origin of the absarokite-banakite association of the Damavand volcano (Iran): Trace elements and Sr, Nd, Pb isotope constraints, *International Journal of Earth Sciences*, **97**(1), 89–102.

[600] Lipman, P. W., Rhodes, J. M., & Dalrymple, G. B., 1990. The Nincole basalt - implications for the structural evolution of Mauna Loa volcano, Hawaii, *Bulletin of Volcanology*, **53**, 1–19.

[601] Litasov, K. D., Foley, S. F., & Litasov, Y. D., 2000. Magmatic modification and metasomatism of the subcontinental mantle beneath the Vitim volcanic field (East Siberia): Evidence from trace element data on pyroxenite and peridotite xenoliths from Miocene picrobasalt, *Lithos*, **54**(1-2), 83–114.

[602] Liu, C. Q., Masuda, A., & Xie, G. H., 1992. Isotope and trace-element geochemistry of alkali basalts and associated megacrysts from the Huangyishan volcano, Kuandian, Liaoning, NE China, *Chemical Geology*, **97**(3-4), 219–231.

[603] Liu, C. Q., Masuda, A., & Xie, G. H., 1994. Major- and trace-element compositions of Cenozoic basalts in eastern China: Petrogenesis and mantle source, *Chemical Geology*, **114**(1-2), 19–42.

[604] Liu, D., Zhao, Z., Zhu, D.-C., Niu, Y., Widom, E., Teng, F.-Z., DePaolo, D. J., Ke, S., Xu, J.-F., Wang, Q.,*et al.*, 2015. Identifying mantle carbonatite metasomatism through Os–Sr–Mg isotopes in Tibetan ultrapotassic rocks, *Earth and Planetary Science Letters*, **430**, 458–469.

[605] Liu, Y., Gao, S., Kelemen, P. B., & Xu, W., 2008. Recycled crust controls contrasting source compositions of Mesozoic and Cenozoic basalts in the North China Craton, *Geochimica et Cosmochimica Acta*, **72**(9), 2349–2376.

[606] Longpré, M. A., Troll, V. R., Walter, T. R., & Hansteen, T. H., 2009. Volcanic and geochemical evolution of the teno massif, tenerife, Canary Islands: Some repercussions of giant landslides on ocean island magmatism, *Geochemistry, Geophysics, Geosystems*, **10**(12).

[607] Longpré, M. A., Klügel, A., Diehl, A., & Stix, J., 2014. Mixing in mantle magma reservoirs prior to and during the 2011-2012 eruption at El Hierro, Canary Islands, *Geology*, **42**(4), 315–318.

[608] Lopes, R. P., Ulbrich, M. N., & Ulbrich, H., 2014. The volcanic-subvolcanic rocks of the Fernando de Noronha archipelago, Southern Atlantic Ocean: Mineral chemistry, *Central European Journal of Geosciences*, **6**(4), 422–456.

[609] López Ruiz, J. & Rodríguez Badiola, E., 1985. La region volcanica Mio-Pleistocena del ne de España, *Estudios Geológicos*, **41**, 105–126.

[610] Lowenstern, J. B., Charlier, B. L., Clynne, M. A., & Wooden, J. L., 2006. Extreme U-Th disequilibrium in rift-related basalts, rhyolites and granophyric granite and the timescale of rhyolite generation, intrusion and crystallization at Alid volcanic center, Eritrea, *Journal of Petrology*, **47**(11), 2105–2122.

[611] Lubala, R., Kampunzu, A., Caron, J., & Vellutini, P., 1982. The nature and interpretation of the Lugulu basalts in South-Kivu, *Comptes Rendus De L'Academie Des Sciences Serie II*, **294**(5), 325–328.

[612] Lubala, R., Kampunzu, A., Caron, J. P.-H., & Vellutini, P., 1987. Petrology and geodynamic significance of the Tertiary alkaline lavas from the Kahuzi–Biega region, Western Rift, Kivu, Zaire, *Geological Journal*, **22**(S2), 515–535.

[613] Lucassen, F., Franz, G., Romer, R. L., Pudlo, D., & Dulski, P., 2008. Nd, Pb, and Sr isotope composition of Late Mesozoic to Quaternary intra-plate magmatism in NE-Africa (Sudan, Egypt): High-μ signatures from the mantle lithosphere, *Contributions to Mineralogy and Petrology*, **156**(6), 765–784.

[614] Lucassen, F., Pudlo, D., Franz, G., Romer, R. L., & Dulski, P., 2013. Cenozoic intra-plate magmatism in the Darfur volcanic province: Mantle source, phonolite-trachyte genesis and relation to other volcanic provinces in NE Africa, *International Journal of Earth Sciences*, **102**(1), 183–205.

[615] Lundstrom, C. C., Hoernle, K., & Gill, J., 2003. U-series disequilibria in volcanic rocks from the Canary Islands: Plume versus lithospheric melting, *Geochimica et Cosmochimica Acta*, **67**(21), 4153–4177.

[616] Lustrino, M., 2000. Petrogenesis of tholeiitic volcanic rocks from central-southern Sardinia, *Mineral. Petrogr. Acta*, **43**, 1–16.

[617] Lustrino M. & Sharkov, E., 2006. Neogene volcanic activity of western Syria and its relationship with Arabian plate kinematics, *Journal of Geodynamics*, **42**(4-5), 115–139.

[618] Lustrino, M. & Wilson, M., 2007. The circum-Mediterranean anorogenic Cenozoic igneous province, *Earth-Science Reviews*, **81**(1-2), 1–65.

[619] Lustrino, M., Melluso, L., Morra, V., & Secchi, F., 1996. Petrology of Plio-Quaternary volcanic rocks from central Sardinia, *Per. Mineral*, **65**, 275–287.

[620] Lustrino, M., Melluso, L., & Morra, V., 2000. The role of lower continental crust and lithospheric mantle in the genesis of Plio-Pleistocene volcanic rocks from Sardinia (Italy), *Earth and Planetary Science Letters*, **180**(3-4), 259–270.

[621] Lustrino, M., Melluso, L., & Morra, V., 2002. The transition from alkaline to tholeiitic magmas: A case study from the Orosei-Dorgali Pliocene volcanic district (NE Sardinia, Italy), *Lithos*, **63**(1-2), 83–113.

[622] Lustrino, M., Keskin, M., Mattioli, M., Lebedev, V. A., Chugaev, A., Sharkov, E., & Kavak, O., 2010. Early activity of the largest Cenozoic shield volcano in the circum-Mediterranean area: Mt. Karacadağ, SE Turkey, *European Journal of Mineralogy*, **22**, 343–362.

[623] Lustrino, M., Cucciniello, C., Melluso, L., Tassinari, C. C. G., dè Gennaro, R., & Serracino, M., 2012. Petrogenesis of Cenozoic volcanic rocks in the NW sector of the Gharyan volcanic field, Libya, *Lithos*, **155**, 218–235.

[624] Lustrino, M., Fedele, L., Melluso, L., Morra, V., Ronga, F., Geldmacher, J., Duggen, S., Agostini, S., Cucciniello, C., Franciosi, L., & Meisel, T., 2013. Origin and evolution of Cenozoic magmatism of Sardinia (Italy). A combined isotopic (Sr-Nd-Pb-O-Hf-Os) and petrological view, *Lithos*, **180-181**, 138–158.

[625] Ma, G. S., Malpas, J., Suzuki, K., Lo, C. H., Wang, K. L., Iizuka, Y., & Xenophontos, C., 2013. Evolution and origin of the Miocene intraplate basalts on the Aleppo Plateau, NW Syria, *Chemical Geology*, **335**, 149–171.

[626] Ma, G. S. K., Malpas, J., Xenophontos, C., & Chan, G. H. N., 2011. Petrogenesis of latest miocene-quaternary continental intraplate volcanism along the northern Dead Sea Fault System (Al Ghab-Homs volcanic field), western Syria: Evidence for lithosphere-asthenosphere interaction, *Journal of Petrology*, **52**(2), 401–430.

[627] Maaløe, S., Sørensen, I., & Hertogen, J., 1986. The trachybasaltic suite of Jan Mayen, *Journal of Petrology*, **27**(2), 439–466.

[628] Maaløe, S., Tumyr, O., & James, D., 1989. Population density and zoning of olivine phenocrysts in tholeiites from Kauai, Hawaii, *Contributions to Mineralogy and Petrology*, **101**(2), 176–186.

[629] Maaløe, S., James, D., Smedley, P., Petersen, S., & Garmann, L. B., 1992. The Koloa volcanic suite of Kauai, Hawaii, *Journal of Petrology*, **33**(4), 761–784.

[630] Macdonald, R., McGarvie, D. W., Pinkerton, H., Smith, R. L., & Palacz, Z. A., 1990. Petrogenetic evolution of the torfajökull volcanic complex, Iceland II. The role of magma mixing, *Journal of Petrology*, **31**(2), 461–481.

[631] Macdonald, R., Davies, G., Upton, B., Dunkley, P., Smith, M., & Leat, P., 1995. Petrogenesis of Silali volcano, Gregory Rift, Kenya, *Journal of the Geological Society*, **152**(4), 703–720.

[632] MacDonald, R., Rogers, N. W., Fitton, J. G., Black, S., & Smith, M., 2001. Plume-Lithosphere Interactions in the Generation of the Basalts of the Kenya Rift, East Africa, *Journal of Petrology*, **42**(5), 877–900.

[633] MacDonald, R., Belkin, H. E., Fitton, J. G., Rogers, N. W., Nejbert, K., Tindle, A. G., & Marshall, A. S., 2008. The roles of fractional crystallization, magma mixing, crystal mush remobilization and volatile-melt interactions in the genesis of a young basalt-peralkaline rhyolite suite, the greater Olkaria volcanic complex, Kenya Rift valley, *Journal of Petrology*, **49**(8), 1515–1547.

[634] Macedo, J., 1989. Petrologia e geoquímica de lavas da ilha de S. Nicolau (arquipélago de Cabo Verde), *Garcia de Orta, Sér. Geol., Lisboa*, **12**(1-2), 1–20.

[635] Macedo, J. R., Alves, C. M., & Palácios, T., 1995. Petrologia das lavas da Ilha da Boavista (arquipélago de Cabo Verde), *Garcia De Orta, Sér. Geologia, Lisboa*, **16**(1–2), 1–18.

[636] MacLennan, J., 2008. Concurrent mixing and cooling of melts under Iceland, *Journal of Petrology*, **49**(11), 1931–1953.

[637] Maclennan, J., McKenzie, D., & Gronvold, K., 2001. Plume-driven upwelling under Central Iceland, *Earth and Planetary Science Letters*, **194**(1-2), 67–82.

[638] Maclennan, J., McKenzie, D., Gronvöld, K., & Slater, L., 2001. Crustal accretion under Northern Iceland, *Earth and Planetary Science Letters*, **191**(3-4), 295–310.

[639] Maclennan, J., McKenzie, D., Hilton, F., Gronvöld, K., & Shimizu, N., 2003. Geochemical variability in a single flow from northern Iceland, *Journal of Geophysical Research: Solid Earth*, **108**(B1), ECV–4 1–21.

[640] Macpherson, C. G., Chiang, K. K., Hall, R., Nowell, G. M., Castillo, P. R., & Thirlwall, M. F., 2010. Plio-Pleistocene intra-plate magmatism from the southern Sulu Arc, Semporna peninsula, Sabah, Borneo: Implications for high-Nb basalt in subduction zones, *Journal of Volcanology and Geothermal Research*, **190**(1-2), 25–38.

[641] Madeira, J., Mata, J., Mourão, C., da Silveira, A. B., Martins, S., Ramalho, R., & Hoffmann, D. L., 2010. Volcano-stratigraphic and structural evolution of Brava Island (Cape Verde) based on $^{40}Ar/^{39}Ar$, U–Th and field constraints, *Journal of Volcanology and Geothermal Research*, **196**(3-4), 219–235.

[642] Madureira, P., Mata, J., Mattielli, N., Queiroz, G., & Silva, P., 2011. Mantle source heterogeneity, magma generation and magmatic evolution at Terceira Island (Azores archipelago): constraints from elemental and isotopic (Sr, Nd, Hf, and Pb) data, *Lithos*, **126**(3-4), 402–418.

[643] Madureira, P., Moreira, M., Mata, J., Nunes, J. C., Gautheron, C., Lourenço, N., Carvalho, R., & de Abreu, M. P., 2014. Helium isotope systematics in the vicinity of the Azores triple junction: Constraints on the Azores geodynamics, *Chemical Geology*, **372**, 62–71.

[644] Mana, S., Furman, T., Carr, M. J., Mollel, G. F., Mortlock, R. A., Feigenson, M. D., Turrin, B. D., & Swisher, C. C., 2012. Geochronology and geochemistry of the Essimingor volcano: Melting of metasomatized lithospheric mantle beneath the North Tanzanian Divergence zone (East African Rift), *Lithos*, **155**, 310–325.

[645] Mancini, A., Mattsson, H. B., & Bachmann, O., 2015. Origin of the compositional diversity in the basalt-to-dacite series erupted along the Heidarspordur ridge, NE Iceland, *Journal of Volcanology and Geothermal Research*, **301**, 116–127.

[646] Mangan, M. T., Heliker, C. C., Mattox, T. N., Kauahikaua, J. P., & Helz, R. T., 1995. Episode 49 of the Pu'u 'O'o-Kupaianaha eruption of Kilauea volcano-breakdown of a steady-state eruptive era, *Bulletin of Volcanology*, **57**(2), 127–135.

[647] Mangas, J., Perez-Torrado, F. J., Massare, D., & Clocchiatti, R., 1993. Phonolitic origin of Roque Nublo ignimbrites of Gran Canaria (Canary Islands, Spain) from clinopyroxene melt inclusion studies, *European Journal of Mineralogy*, **5**(1), 97–106.

[648] Manning, C. J. & Thirlwall, M. F., 2014. Isotopic evidence for interaction between Öræfajökull mantle and the Eastern Rift Zone, Iceland, *Contributions to Mineralogy and Petrology*, **167**(1), 1–22.

[649] Manthei, C. D., Ducea, M. N., Girardi, J. D., Patchett, P. J., & Gehrels, G. E., 2010. Isotopic and geochemical evidence for a recent transition in mantle chemistry beneath the western Canadian Cordillera, *Journal of Geophysical Research*, **115**(B2), 1–19.

[650] Marcantonio, F., Zindler, A., Elliott, T., & Staudigel, H., 1995. Os isotope systematics of La Palma, Canary Islands: Evidence for recycled crust in the mantle source of HIMU ocean islands, *Earth and Planetary Science Letters*, **133**(3-4), 397–410.

[651] Marcelot, G., Dupuy, C., Dostal, J., Rançon, J. P., & Pouclet, A., 1989. Geochemistry of mafic volcanic rocks from the Lake Kivu (Zaire and Rwanda) section of the western branch of the African Rift, *Journal of Volcanology and Geothermal Research*, **39**(1), 73–88.

[652] Marques, L. S., Ulbrich, M. N., Ruberti, E., & Tassinari, C. G., 1999. Petrology, geochemistry and Sr-Nd isotopes of the Trindade and Martin Vaz volcanic rocks (Southern Atlantic Ocean), *Journal of Volcanology and Geothermal Research*, **93**(3-4), 191–216.

[653] Marquez, A. & De Ignacio, C., 2002. Mineralogical and geochemical constraints for the origin and evolution of magmas in Sierra Chichinautzin, central Mexican Volcanic Belt, *Lithos*, **62**(1-2), 35–62.

[654] Martí, J., Pinel, V., Lõpez, C., Geyer, A., Abella, R., Tárraga, M., Blanco, M. J., Castro, A., & Rodríguez, C., 2013. Causes and mechanisms of the 2011-2012 El Hierro (Canary Islands) submarine eruption, *Journal of Geophysical Research: Solid Earth*, **118**(3), 823–839.

[655] Martin, A. P., Cooper, A. F., & Price, R. C., 2013. Petrogenesis of Cenozoic, alkalic volcanic lineages at Mount Morning, West Antarctica and their entrained lithospheric mantle xenoliths: Lithospheric versus asthenospheric mantle sources, *Geochimica et Cosmochimica Acta*, **122**, 127–152.

[656] Martins, S., Mata, J., Munhá, J., Mendes, M. H., Maerschalk, C., Caldeira, R., & Mattielli, N., 2010. Chemical and mineralogical evidence of the occurrence of mantle metasomatism by carbonate-rich melts in an oceanic environment (Santiago Island, Cape Verde), *Mineralogy and Petrology*, **99**(1-2), 43–65.

[657] Marzoli, A., Renne, P. R., Piccirillo, E. M., Castorina, F., Bellieni, G., Melfi, A. J., Nyobe, J. B., & N'ni, J., 1999. Silicic magmas from the continental Cameroon Volcanic Line (Oku, Bambouto and Ngaoundere): ^{40}Ar-^{39}Ar dates, petrology, Sr-Nd-O isotopes and their petrogenetic significance, *Contributions to Mineralogy and Petrology*, **135**(2-3), 133–150.

[658] Marzoli, A., Piccirillo, E. M., Renne, P. R., Bellieni, G., Iacumin, M., Nyobe, J. B., & Tongwa, A. T., 2000. The cameroon volcanic line revisited: Petrogenesis of continental basaltic magmas from lithospheric and asthenospheric mantle sources, *Journal of Petrology*, **41**(1), 87–109.

[659] Marzoli, A., Aka, F. T., Merle, R., Callegaro, S., & N'ni, J., 2015. Deep to shallow crustal differentiation of within-plate alkaline magmatism at Mt. Bambouto volcano, Cameroon Line, *Lithos*, **220-223**, 272–288.

[660] Mata, J. & Munhá, J., 2004. Madeira Island alkaline lava spinels: Petrogenetic implications, *Mineralogy and Petrology*, **81**(1-2), 85–111.

[661] Mata, J., Kerrich, R., MacRae, N. D., & Wu, T. W., 1998. Elemental and isotopic (Sr, Nd, and Pb) characteristics of Madeira Island basalts: evidence for a composite HIMU - EM I plume fertilizing lithosphere, *Canadian Journal of Earth Sciences*, **35**, 980–997.

[662] Mattey, D. P., 1982. The minor and trace element geochemistry of volcanic rocks from Truk, Ponape and Kusaie, Eastern Caroline Islands; the evolution of a young hot spot trace across Old Pacific Ocean Crust, *Contributions to Mineralogy and Petrology*, **80**(1), 1–13.

[663] Mattsson, H. B., 2012. Rapid magma ascent and short eruption durations in the Lake Natron–Engaruka monogenetic volcanic field (Tanzania): a case study of the olivine melilititic Pello Hill scoria cone, *Journal of Volcanology and Geothermal Research*, **247**, 16–25.

[664] Mattsson, H. B. & Oskarsson, N., 2005. Petrogenesis of alkaline basalts at the tip of a propagating rift: Evidence from the Heimaey volcanic centre, south Iceland, *Journal of Volcanology and Geothermal Research*, **147**(3-4), 245–267.

[665] Maury, R. C., El Azzouzi, M., Bellon, H., Liotard, J.-M., Guille, G., Barsczus, H. G., Chauvel, C., Diraison, C., Dupuy, C., Vidal, P., & Brousse, R., 1994. Geology and petrology of Tubuai (Austral Islands, French Polynesia), *C.R. Acad. Sci. Paris*, **318**, 1341–1347.

[666] Mayer, B., Jung, S., Romer, R. L., Stracke, A., Haase, K. M., & Garbe-Schönberg, C. D., 2013. Petrogenesis of tertiary hornblende-bearing lavas in the rhön, germany, *Journal of Petrology*, **54**(10), 2095–2123.

[667] Maza, M., Dautria, J.-M., Briqueu, L., & Cantagrel, J.-M., 1995. Massif annulaire de l'Achkal: un témoin d'un magmatisme alcalin d'âge oligocène supérieur au Hoggar centro-oriental, *Bulletin Service géologique d'Algérie*, **6**, 61–77.

[668] Mazarovich, A., Frikh-Kar, D., Kogarko, L., Koporulin, V., Rikhter, A., Akhmetev, M., & Zolotarev, B., 1990. *Tectonics and Magmatism of the Cape Verde Islands*, Nauka, Moscow, Russia.

[669] McBride, J. S., Lambert, D. D., Nicholls, I. A., & Price, R. C., 2001. Osmium isotopic evidence for crust-mantle interaction in the genesis of continental intraplate basalts from the Newer Volcanics Province, Southeastern Australia, *Journal of Petrology*, **42**(6), 1197–1218.

[670] McDonough, W., McCulloch, M., & Sun, S., 1985. Isotopic and geochemical systematics in Tertiary-Recent basalts from southeastern Australia and implications for the evolution of the sub-continental lithosphere, *Geochimica et Cosmochimica Acta*, **49**(10), 2051–2067.

[671] McDonough, W. F. & Sun, S. S., 1995. The composition of the Earth, *Chemical Geology*, **120**(3-4), 223–253.

[672] McDougall, I., Embleton, B., & Stone, D., 1981. Origin and evolution of Lord Howe Island, southwest Pacific Ocean, *Journal of the Geological Society of Australia*, **28**(1-2), 155–176.

[673] McGarvie, D. W., Macdonald, R., Pinkerton, H., & Smith, R. L., 1990. Petrogenetic evolution of the torfajökull volcanic complex, Iceland II. The role of magma mixing, *Journal of Petrology*, **31**(2), 461–481.

[674] McGee, B. M., 2005. Characteristics and origin of the Weldborough sapphire, NE Tasmania, *Unpublished BSc thesis): University of Tasmania, School of Earth Science, Hobart, Australia.*

[675] McGee, L. E., Millet, M. A., Smith, I. E. M., Németh, K., & Lindsay, J. M., 2012. The inception and progression of melting in a monogenetic eruption: Motukorea Volcano, the Auckland Volcanic Field, New Zealand, *Lithos*, **155**, 360–374.

[676] McGee, L. E., Smith, I. E., Millet, M. A., Handley, H. K., & Lindsay, J. M., 2013. Asthenospheric control of melting processes in a monogenetic basaltic system: A case study of the Auckland volcanic field, New Zealand, *Journal of Petrology*, **54**(10), 2125–2153.

[677] McKenzie, D. & O'Nions, R. K., 1995. The Source Regions of Ocean Island Basalts, *Journal of Petrology*, **36**(1), 133–159.

[678] McMillan, N. J., Dickin, A. P., & Haag, D., 2000. Evolution of magma source regions in the Rio Grande rift, Southern New Mexico, *Bulletin of the Geological Society of America*, **112**(10), 1582–1593.

[679] McNab, F., Ball, P. W., Hoggard, M. J., & White, N. J., 2018. Neogene uplift and magmatism of Anatolia: Insights from drainage analysis and basaltic geochemistry, *Geochemistry, Geophysics, Geosystems*, **19**(1), 175–213.

[680] Mehdizadeh, H., Liotard, J.-M., & Dautria, J.-M., 2002. Geochemical characteristics of an intracontinental shoshonitic association: the example of the Damavand volcano, Iran, *Comptes Rendus Geoscience*, **334**(2), 111–117.

[681] Melluso, L. & Morra, V., 2000. Petrogenesis of Late Cenozoic mafic alkaline rocks of the Nosy Be archipelago (northern Madagascar): relationships with the Comorean magmatism, *Journal of Volcanology and Geothermal Research*, **96**(1-2), 129–142.

[682] Melluso, L., Morra, V., Brotzu, P., Franciosi, L., Grifa, C., Lustrino, M., Morbidelli, P., Riziky, H., & Vincent, M., 2007. The Cenozoic alkaline magmatism in central-northern Madagascar: A brief overview, *Periodico di Mineralogia*, **76**(3), 169–180.

[683] Melluso, L., Morra, V., Riziky, H., Veloson, J., Lustrino, M., Del Gatto, L., & Modeste, V., 2007. Petrogenesis of a basanite–tephrite–phonolite volcanic suite in the Bobaomby (Cap d'Ambre) peninsula, northern Madagascar, *Journal of African Earth Sciences*, **49**(1-2), 29–42.

[684] Melluso, L., le Roex, A. P., & Morra, V., 2011. Petrogenesis and Nd-, Pb-, Sr-isotope geochemistry of the Cenozoic olivine melilitites and olivine nephelinites ("ankaratrites") in Madagascar, *Lithos*, **127**(3-4), 505–521.

[685] Melluso, L., Cucciniello, C., le Roex, A. P., & Morra, V., 2016. The geochemistry of primitive volcanic rocks of the Ankaratra volcanic complex, and source enrichment processes in the genesis of the Cenozoic magmatism in Madagascar, *Geochimica et Cosmochimica Acta*, **185**, 435–452.

[686] Meng, F.-C., Safonova, I., Chen, S.-S., & Rioual, P., 2018. Late Cenozoic intra-plate basalts of the Greater Khingan Range in NE China and Khangai Province in Central Mongolia, *Gondwana Research*, **63**, 65–84.

[687] Menzies, M. & Murthy, V. R., 1980. Mantle metasomatism as a precursor to the genesis of alkaline magmas-isotopic evidence, *American Jounal of Science*, **280**, 622–638.

[688] Menzies, M. A. & J, W. M., N.D. Whole-rock geochemical analyses from the Aïr volcanic field, Niger, Tech. rep., University of Leeds.

[689] Mertz, D. F., Löhnertz, W., Nomade, S., Pereira, A., Prelević, D., & Renne, P. R., 2015. Temporal-spatial evolution of low-SiO2 volcanism in the Pleistocene West Eifel volcanic field (West Germany) and relationship to upwelling asthenosphere, *Journal of Geodynamics*, **88**, 59–79.

[690] Meshesha, D. & Shinjo, R., 2007. Crustal contamination and diversity of magma sources in the northwestern Ethiopian volcanic province, *Journal of Mineralogical and Petrological Sciences*, pp. 0706050032–0706050032.

[691] Métrich, N., Zanon, V., Créon, L., Hildenbrand, A., Moreira, M., & Marques, F. O., 2014. Is the 'Azores hotspot' a wetspot? Insights from the geochemistry of fluid and melt inclusions in olivine of Pico basalts, *Journal of Petrology*, **55**(2), 377–393.

[692] Meyer, R., Abratis, M., Viereck-Götte, L., Mädler, J., Hertogen, J., & Romer, R., 2002. Mantelquellen des Vulkanismus in der thüringischen Rhön, *Beitr. Geol. Thüringen*, **9**, 75–105.

[693] Meyzen, C. M., Marzoli, A., Bellieni, G., & Levresse, G., 2016. Magmatic activity on a motionless plate: The case of East Island, Crozet Archipelago (Indian Ocean), *Journal of Petrology*, **57**(7), 1409–1436.

[694] Michael, P., 1995. Regionally distinctive sources of depleted MORB: Evidence from trace elements and H_2O, *Earth and Planetary Science Letters*, **131**(3–4), 301–320.

[695] Miller, C., Schuster, R., Klötzli, U., Frank, W., & Purtscheller, F., 1999. Post-collisional potassic and ultrapotassic magmatism in SW Tibet: geochemical and Sr–Nd–Pb–O isotopic constraints for mantle source characteristics and petrogenesis, *Journal of Petrology*, **40**(9), 1399–1424.

[696] Miller, C., Zanetti, a., Thöni, M., Konzett, J., & Klötzli, U., 2012. Mafic and silica-rich glasses in mantle xenoliths from Wau-en-Namus, Libya: Textural and geochemical evidence for peridotite-melt reactions, *Lithos*, **128-131**, 11–26.

[697] Millet, M.-A., Doucelance, R., Schiano, P., David, K., & Bosq, C., 2008. Mantle plume heterogeneity versus shallow-level interactions: a case study, the São Nicolau Island, Cape Verde archipelago, *Journal of Volcanology and Geothermal Research*, **176**(2), 265–276.

[698] Millet, M.-A., Doucelance, R., Baker, J. A., & Schiano, P., 2009. Reconsidering the origins of isotopic variations in Ocean Island Basalts: insights from fine-scale study of São Jorge Island, Azores archipelago, *Chemical Geology*, **265**(3-4), 289–302.

[699] Mirnejad, H., Hassanzadeh, J., Cousens, B. L., & Taylor, B. E., 2010. Geochemical evidence for deep mantle melting and lithospheric delamination as the origin of the inland Damavand volcanic rocks of northern Iran, *Journal of Volcanology and Geothermal Research*, **198**(3-4), 288–296.

[700] Mo, X., Zhao, Z., Deng, J., Flower, M., Yu, X., Luo, Z., Li, Y., Zhou, S., Dong, G., Zhu, D.,*et al.*, 2006. Petrology and geochemistry of postcollisional volcanic rocks from the Tibetan plateau: implications for lithosphere heterogeneity and collision-induced asthenospheric mantle flow, *Special Papers – Geological Society of America*, **409**, 507.

[701] Mohr, P., Mitchell, J. G., & Raynolds, R. G., 1980. Quaternary volcanism and faulting at O'A caldera, central ethiopian rift, *Bulletin Volcanologique*, **43**(1), 173–189.

[702] Mollel, G. F., 2007. *Petrochemistry and geochronology of Ngorongoro volcanic highland complex (NVHC) and its relationship to Laetoli and Olduvai Gorge, Tanzania*, Ph.D. thesis, Rutgers University-Graduate School-New Brunswick.

[703] Mollel, G. F., Swisher III, C. C., Feigenson, M. D., & Carr, M. J., 2008. Geochemical evolution of Ngorongoro Caldera, Northern Tanzania: Implications for crust–magma interaction, *Earth and Planetary Science Letters*, **271**(1-4), 337–347.

[704] Momme, P., Óskarsson, N., & Keays, R. R., 2003. Platinum-group elements in the Icelandic rift system: melting processes and mantle sources beneath Iceland, *Chemical Geology*, **196**(1-4), 209–234.

[705] Montanini, A., Barbieri, M., & Castorina, F., 1994. The role of fractional crystallisation, crustal melting and magma mixing in the petrogenesis of rhyolites and mafic inclusion-bearing dacites from the Monte Arci volcanic complex (Sardinia, Italy), *Journal of Volcanology and Geothermal Research*, **61**(1-2), 95–120.

[706] Moore, G., Marone, C., Carmichael, I. S., & Renne, P., 1994. Basaltic volcanism and extension near the intersection of the Sierra Madre volcanic province and the Mexican Volcanic Belt, *Geological Society of America Bulletin*, **106**(3), 383–394.

[707] Moore, J., White, W. M., Paul, D., Duncan, R. A., Abouchami, W., & Galer, S. J., 2011. Evolution of shield-building and rejuvenescent volcanism of Mauritius, *Journal of Volcanology and Geothermal Research*, **207**(1-2), 47–66.

[708] Moore, J. G. & Campbell, J. F., 1987. Age of tilted reefs, Hawaii, *Journal of Geophysical Research: Solid Earth*, **92**(B3), 2641–2646.

[709] Moore, J. G. & Clague, D. A., 1992. Volcano growth and evolution of the island of hawaii, *Geological Society of America Bulletin*, **104**(11), 1471–1484.

[710] Moore, J. G., Clague, D. A., Ludwig, K. R., & Mark, R. K., 1990. Subsidence and volcanism of the Haleakala Ridge, Hawaii, *Journal of Volcanology and Geothermal Research*, **42**(3), 273–284.

[711] Moore, J. G., Hickson, C. J., & Calk, L. C., 1995. Tholeiitic-alkalic transition at subglacial volcanoes, Tuya region, British Columbia, Canada, *Journal of Geophysical Research: Solid Earth*, **100**(B12), 24577–24592.

[712] Moore, R. B., 1983. Distribution of differentiated tholeiitic basalts on the lower east rift zone of Kilauea Volcano, Hawaii: a possible guide to geothermal exploration., *Geology*, **11**(3), 136–140.

[713] Moore, R. B., Clague, D. A., Rubin, M., & Bohrson, W. A., 1987. Hualalai volcano: a preliminary summary of geologic, petrologic, and geophysical data, in *Volcanism in Hawaii*, edited by R. W. Decker, T. L. Wright, & P. H. Stauffer, chap. 20, pp. 571–585, U.S. Geological Survey.

[714] Moreno, F. A. P., Demant, A., Cochemé, J.-J., Dostal, J., & Montigny, R., 2003. The quaternary moctezuma volcanic field: A tholeiitic to alkali basaltic episode in the central sonoran basin and range province, méxico, *Special Papers – Geological Society of America*, pp. 439–456.

[715] Morris, P. A., 1986. Constraints on the origin of mafic alkaline volcanics and included xenoliths from Oberon, New South Wales, Australia, *Contributions to Mineralogy and Petrology*, **93**, 207–214.

[716] Moufti, M. R., Moghazi, A. M., & Ali, K. A., 2012. Geochemistry and Sr–Nd–Pb isotopic composition of the Harrat Al-Madinah Volcanic Field , Saudi Arabia, *Gondwana Research*, **21**(2-3), 670–689.

[717] Moufti, M. R., Moghazi, A. M., & Ali, K. A., 2013. ^{40}Ar/^{39}Ar geochronology of the Neogene-Quaternary Harrat Al-Madinah intercontinental volcanic field, Saudi Arabia: Implications for duration and migration of volcanic activity, *Journal of Asian Earth Sciences*, **62**, 253–268.

[718] Mourão, C., Mata, J., Doucelance, R., Madeira, J., da Silveira, A. B., Silva, L. C., & Moreira, M., 2010. Quaternary extrusive calciocarbonatite volcanism on Brava Island (Cape Verde): a nephelinite-carbonatite immiscibility product, *Journal of African Earth Sciences*, **56**(2-3), 59–74.

[719] Mourão, C., Mata, J., Doucelance, R., Madeira, J., Millet, M.-A., & Moreira, M., 2012. Geochemical temporal evolution of Brava Island magmatism: constraints on the variability of Cape Verde mantle sources and on carbonatite–silicate magma link, *Chemical Geology*, **334**, 44–61.

[720] Mouty, M., Delaloye, M., Fontignie, D., Piskin, O., & Wagner, J.-J., 1992. The volcanic activity in Syria and Lebanon between Jurassic and Actual, *Schweizerische Mineralogische und Petrographische Mitteilungen*, **72**(1), 91–105.

[721] Mukhopadhyay, S., Lassiter, J. C., Farley, K. A., & Bogue, S. W., 2003. Geochemistry of Kauai shield-stage lavas: Implications for the chemical evolution of the Hawaiian plume, *Geochemistry, Geophysics, Geosystems*, **4**(1).

[722] Mungall, J. E. & Martin, R., 1995. Petrogenesis of basalt-comendite and basalt-pantellerite suites, Terceira, Azores, and some implications for the origin of ocean-island rhyolites, *Contributions to Mineralogy and Petrology*, **119**(1), 43–55.

[723] Muravyeva, N., Belyatsky, B., & Senin, V., 2013. Sr-Nd isotopic disequilibrium of clinopyroxenes from the ultrapotassic effusive rocks of the East African rift system: Mixing of melts and source heterogeneity, *Geochemistry International*, **51**(6), 505.

[724] Muravyeva, N. S., Belyatsky, B. V., Senin, V. G., & Ivanov, A. V., 2014. Sr-Nd-Pb isotope systematics and clinopyroxene-host disequilibrium in ultra-potassic magmas from Toro-Ankole and Virunga, East-African Rift: Implications for magma mixing and source heterogeneity, *Lithos*, **210-211**, 260–277.

[725] Murcia, H., 2015. *Monogenetic volcanism in the western Arabian Peninsula: Insights from Late Quaternary eruptions in northern Harrat Rahat, Kingdom of Saudi Arabia*, Ph.D. thesis, The University of Auckland.

[726] Murcia, H., Németh, K., El-Masry, N., Lindsay, J., Moufti, M., Wameyo, P., Cronin, S., Smith, I., & Kereszturi, G., 2015. The Al-Du'aythah volcanic cones, Al-Madinah City: implications for volcanic hazards in northern Harrat Rahat, Kingdom of Saudi Arabia, *Bulletin of Volcanology*, **77**(6), 54.

[727] Nakamura, Y. & Aoki, K., 1980. The 1977 eruption of Nyiragongo volcano, eastern Africa, and chemical composition of the ejecta, *Bulletin of the Volcanological Society of Japan*, **25**(2), 17–32.

[728] Nasir, S. & Al-Fuqha, H., 1988. Spinel-Lherzolite Xenoliths from the Aritain Volcano, NE-Jordan, *Mineralogy and Petrology*, **38**(2), 127–137.

[729] Nasir, S. J., Everard, J. L., McClenaghan, M. P., Bombardieri, D., & Worthing, M. A., 2010. The petrology of high pressure xenoliths and associated Cenozoic basalts from Northeastern Tasmania, *Lithos*, **118**(1-2), 35–49.

[730] Natali, C., Beccaluva, L., Bianchini, G., & Siena, F., 2011. Rhyolites associated to Ethiopian CFB: clues for initial rifting at the Afar plume axis, *Earth and Planetary Science Letters*, **312**(1-2), 59–68.

[731] Natali, C., Beccaluva, L., Bianchini, G., Ellam, R. M., Savo, A., Siena, F., & Stuart, F. M., 2016. High-MgO lavas associated to CFB as indicators of plume-related thermochemical effects: The case of ultra-titaniferous picrite-basalt from the Northern Ethiopian-Yemeni Plateau, *Gondwana Research*, **34**, 29–48.

[732] Naumann, T. R., Geist, D. J., & Kurz, M. D., 2002. Petrology and Geochemistry of Volcan Cerro Azul: Petrologic Diversity among the Western Galapagos Volcanoes, *Journal of Petrology*, **43**(5), 859–883.

[733] Naumov, V. B., Portnyagin, M. V., Tolstykh, M. L., & Yarmolyuk, V. V., 2003. Composition of magmatic melts from the southern Baikal volcanic region: A study of inclusions in olivine from trachybasalts, *Geochemistry International*, **41**(3), 213–223.

[734] Naumov, V. B., Portnyagin, M. V., Tolstykh, M. L., & Yarmolyuk, V. V., 2006. Chemical composition and crystallization conditions of trachybasalts from the Dzhida field, Southern Baikal volcanic area: Evidence from melt and fluid inclusions, *Geochemistry International*, **44**(3), 286–295.

[735] Ndiaye, A., Ngom, P. M.,*et al.*, 2014. The Geodynamic Context of the Cenozoic Volcanism of the Cap-Vert Peninsula (Senegal), *International Journal of Geosciences*, **5**(12), 1521.

[736] Neave, D. A., Maclennan, J., Hartley, M. E., Edmonds, M., & Thordarson, T., 2014. Crystal storage and transfer in basaltic systems: The skuggafjöll eruption, Iceland, *Journal of Petrology*, **55**(12), 2311_2346.

[737] Neill, I., Meliksetian, K., Allen, M. B., Navasardyan, G., & Kuiper, K., 2015. Petrogenesis of mafic collision zone magmatism: The Armenian sector of the Turkish-Iranian Plateau, *Chemical Geology*, **403**, 24–41.

[738] Nelson, D. R., McCulloch, M. T., & Sun, S.-S., 1986. The origins of ultrapotassic rocks as inferred from Sr, Nd and Pb isotopes, *Geochimica et Cosmochimica Acta*, **50**(2), 231–245.

[739] Nelson, W. R., Furman, T., van Keken, P. E., Shirey, S. B., & Hanan, B. B., 2012. Os-Hf isotopic insight into mantle plume dynamics beneath the East African Rift System, *Chemical Geology*, **320**, 66–79.

[740] Neumann, E.-R., Martí, J., Mitjavila, J., & Wulff-Pedersen, E., 1999. Origin and implications of mafic xenoliths associated with Cenozoic extension-related volcanism in the Valencia Trough, NE Spain, *Mineralogy and Petrology*, **65**(1-2), 113–139.

[741] Ngonge, E. D., de Hollanda, M. H. B. M., Pimentel, M. M., & de Oliveira, D. C., 2016. Petrology of the alkaline rocks of the Macau Volcanic Field, NE Brazil, *Lithos*, **266-267**, 453–470.

[742] Nicholson, H. & Latin, D., 1992. Olivine tholeiites from Krafla, Iceland: Evidence for variations in melt fraction within a plume, *Journal of Petrology*, **33**(5), 1105–1124.

[743] Nixon, S., 2011. *Intra-plate magmatism of the Al Haruj volcanic field, Libya*, Ph.D. thesis, University of Cambridge.

[744] Njome, M. S., Suh, C. E., Sparks, R. S. J., Ayonghe, S. N., & Fitton, J. G., 2008. The Mount Cameroon 1959 compound lava flow field: morphology, petrography and geochemistry, *Swiss Journal of Geosciences*, **101**(1), 85–98.

[745] Nkouandou, O. F. & Temdjim, R., 2011. Petrology of spinel lherzolite xenoliths and host basaltic lava from Ngao Voglar Volcano, Adamawa Massif (Cameroon Volcanic Line, West Africa): Equilibrium conditions and mantle characteristics, *Journal of Geosciences*, **56**(4), 375–387.

[746] Nkouathio, D. G., Ménard, J. J., Wandji, P., & Bardintzeff, J. M., 2002. The Tombel graben (West Cameroon): A recent monogenetic volcanic field of the Cameroon Line, *Journal of African Earth Sciences*, **35**(2), 285–300.

[747] Nkouathio, D. G., Kagou Dongmo, A., Bardintzeff, J. M., Wandji, P., Bellon, H., & Pouclet, A., 2008. Evolution of volcanism in graben and horst structures along the Cenozoic Cameroon Line (Africa): Implications for tectonic evolution and mantle source composition, *Mineralogy and Petrology*, **94**(3-4), 287–303.

[748] Nkoumbou, C., Déruelle, B., & Velde, D., 1995. Petrology of Mt Etinde nephelinite series, *Journal of Petrology*, **36**(2), 373–395.

[749] Nohda, S., Kaneoka, I., Hanyu, T., Xu, S., & Uto, K., 2005. Systematic variation of Sr-, Nd- and Pb- isotopes with time in lavas of Mauritius, Réunion hotspot, *Journal of Petrology*, **46**(3), 504–521.

[750] Nomade, S., Renne, P. R., Mo, X., Zhao, Z., & Zhou, S., 2004. Miocene volcanism in the Lhasa block, Tibet: spatial trends and geodynamic implications, *Earth and Planetary Science Letters*, **221**(1-4), 227–243.

[751] Nono, A., Déruelle, B., Demaiffe, D., & Kambou, R., 1994. Tchabal Nganha volcano in Adamawa (Cameroon): petrology of a continental alkaline lava series, *Journal of Volcanology and Geothermal Research*, **60**(2), 147–178.

[752] Norman, M. D. & Garcia, M. O., 1999. Primitive magmas and source characteristics of the Hawaiian plume: petrology and geochemistry of shield picrites, *Earth and Planetary Science Letters*, **168**(1-2), 27–44.

[753] Norman, M. D., Garcia, M. O., Kamenetsky, V. S., & Nielsen, R. L., 2002. Olivine-hosted melt inclusions in Hawaiian picrites: Equilibration, melting, and plume source characteristics, *Chemical Geology*, **183**(1-4), 143–168.

[754] Norman, M. D., Garcia, M. O., & Bennett, V. C., 2004. Rhenium and chalcophile elements in basaltic glasses from Ko'olau and Moloka'i volcanoes: Magmatic outgassing and composition of the Hawaiian plume, *Geochimica et Cosmochimica Acta*, **68**(18), 3761–3777.

[755] Notsu, K., Fujitani, T., Ui, T., Matsuda, J., & Ercan, T., 1995. Geochemical features of collision-related volcanic rocks in central and eastern Anatolia, Turkey, *Journal of Volcanology and Geothermal Research*, **64**, 171–192.

[756] O'Connor, J., Stoffers, P., van den Bogaard, P., & McWilliams, M., 1999. First seamount age evidence for significantly slower African plate motion since 19 to 30 Ma, *Earth and Planetary Science Letters*, **171**(4), 575–589.

[757] Omrani, J., Agard, P., Whitechurch, H., Benoit, M., Prouteau, G., & Jolivet, L., 2008. Arc-magmatism and subduction history beneath the Zagros Mountains, Iran: A new report of adakites and geodynamic consequences, *Lithos*, **106**(3-4), 380–398.

[758] Oppenheimer, C., Moretti, R., Kyle, P. R., Eschenbacher, A., Lowenstern, J. B., Hervig, R. L., & Dunbar, N. W., 2011. Mantle to surface degassing of alkalic magmas at Erebus volcano, Antarctica, *Earth and Planetary Science Letters*, **306**(3-4), 261–271.

[759] O'Reilly, S. Y. & Griffin, W., 1984. Sr isotopic heterogeneity in primitive basaltic rocks, southeastern Australia: correlation with mantle metasomatism, *Contributions to Mineralogy and Petrology*, **87**(3), 220–230.

[760] O'Reilly, S. Y. & Zhang, M., 1995. Geochemical characteristics of lava-field basalts from eastern Australia and inferred sources: connections with the subcontinental lithospheric mantle?, *Contributions to Mineral Petrology*, **121**, 148–170.

[761] Ottonello, G., Piccardo, G. B., Joron, J. L., & Treuil, M., 1978. Evolution of the upper mantle under the Assab Region (Ethiopia): Suggestions from petrology and geochemistry of tectonitic ultramafic xenoliths and host basaltic lavas, *Geologische Rundschau*, **67**(2), 547–575.

[762] Oyan, V., Keskin, M., Lebedev, V. A., Chugaev, A. V., & Sharkov, E. V., 2016. Magmatic evolution of the Early Pliocene Etrüsk stratovolcano, Eastern Anatolia Collision Zone, Turkey, *Lithos*, **256–257**, 88–108.

[763] Oyan, V., Keskin, M., Lebedev, V. A., Chugaev, A. V., Sharkov, E. V., & Ünal, E., 2017. Petrology and Geochemistry of the Quaternary Mafic Volcanism to the NE of Lake Van, Eastern Anatolian Collision Zone, Turkey, *Journal of Petrology*, **58**(9), 1701–1728.

[764] Özdemir, Y., Karaoğlu, O., Tolluoğlu, A. U., & Güleç, N., 2006. Volcanostratigraphy and petrogenesis of the Nemrut stratovolcano (East Anatolian High Plateau): The most recent post-collisional volcanism in Turkey, *Chemical Geology*, **226**, 189–211.

[765] Palacz, Z. A. & Saunder, A. D., 1986. Coupled trace element and isotope enrichment in the Cook-Austral-Samoa Islands, Southwest Pacific, *Earth and Planetary Science Letters*, **79**(3-4), 270–280.

[766] Pallares, C., Maury, R. C., Bellon, H., Royer, J. Y., Calmus, T., Aguillón-Robles, A., Cotten, J., Benoit, M., Michaud, F., & Bourgois, J., 2007. Slab-tearing following ridge-trench collision: Evidence from Miocene volcanism in Baja California, México, *Journal of Volcanology and Geothermal Research*, **161**(1-2), 95–117.

[767] Pallister, J. S., 1987. Magmatic history of Red Sea rifting: perspective from the central Saudi Arabian coastal plain., *Geological Society of America Bulletin*, **98**(4), 400–417.

[768] Pang, K. N., Chung, S. L., Zarrinkoub, M. H., Wang, F., Kamenetsky, V. S., & Lee, H. Y., 2015. Quaternary high-Mg ultrapotassic rocks from the Qal'eh Hasan Ali maars, southeastern Iran: petrogenesis and geodynamic implications, *Contributions to Mineralogy and Petrology*, **170**(3), 1–19.

[769] Panter, K. S., Hart, S. R., Kyle, P., Blusztanjn, J., & Wilch, T., 2000. Geochemistry of Late Cenozoic basalts from the Crary Mountains: Characterization of mantle sources in Marie Byrd Land, Antarctica, *Chemical Geology*, **165**(3-4), 215–241.

[770] Panter, K. S., Blusztajn, J., Hart, S. R., Kyle, P. R., Esser, R., & McIntosh, W. C., 2006. The origin of HIMU in the SW Pacific: Evidence from intraplate volcanism in Southern New Zealand and Subantarctic Islands, *Journal of Petrology*, **47**(9), 1673–1704.

[771] Parlak, O., Kop, A., Ünlügenç, U. C., & Demirkol, C., 1998. Geochronology and geochemistry of basaltic rocks in the Karasu Graben around Kırıkhan (Hatay), S. Turkey, *Turkish Journal of Earth Sciences*, **7**, 53–61.

[772] Paslick, C., Halliday, A., James, D., & Dawson, J. B., 1995. Enrichment of the continental lithosphere by OIB melts: Isotopic evidence from the volcanic province of northern Tanzania, *Earth and Planetary Science Letters*, **130**, 109–126.

[773] Paslick, C. R., Halliday, A. N., Lange, R. A., James, D., & Dawson, J. B., 1996. Indirect crustal contamination: evidence from isotopic and chemical disequilibria in minerals from alkali basalts and nephelinites from northern Tanzania, *Contributions to Mineralogy and Petrology*, **125**(4), 277–292.

[774] Pasteels, P., Villeneuve, M., De Paepe, P., & Klerkx, J., 1989. Timing of the volcanism of the southern Kivu province: implications for the evolution of the western branch of the East African Rift system, *Earth and Planetary Science Letters*, **94**(3-4), 353–363.

[775] Paton, S. M., 1992. *The relationship between extension and volcanism in Western Turkey, the Aegean Sea and Central Greece*, Ph.D. thesis, University of Cambridge.

[776] Paul, B., Hergt, J., & Woodhead, J., 2005. Mantle heterogeneity beneath the Cenozoic volcanic provinces of central Victoria inferred from trace-element and Sr, Nd, Pb and Hf isotope data, *Australian Journal of Earth Sciences*, **52**(2), 243–260.

[777] Paul, D., White, W. M., & Blichert-Toft, J., 2005. Geochemistry of Mauritius and the origin of rejuvenescent volcanism on oceanic island volcanoes, *Geochemistry, Geophysics, Geosystems*, **6**(6).

[778] Paul, D., Kamenetsky, V. S., Hofmann, A. W., & Stracke, A., 2007. Compositional diversity among primitive lavas of Mauritius, Indian Ocean: Implications for mantle sources, *Journal of Volcanology and Geothermal Research*, **164**(1-2), 76–94.

[779] Pearce, J., Bender, J., De Long, S., Kidd, W., Low, P., Güner, Y., Saroglu, F., Yilmaz, Y., Moorbath, S., & Mitchell, J., 1990. Genesis of collision volcanism in Eastern Anatolia, Turkey, *Journal of Volcanology and Geothermal Research*, **44**, 189–229.

[780] Peate, D. W., Baker, J. A., Jakobsson, S. P., Waight, T. E., Kent, A. J., Grassineau, N. V., & Skovgaard, A. C., 2009. Historic magmatism on the Reykjanes Peninsula, Iceland: A snap-shot of melt generation at a ridge segment, *Contributions to Mineralogy and Petrology*, **157**(3), 359–382.

[781] Peate, D. W., Breddam, K., Baker, J. A., Kurz, M. D., Barker, A. K., Prestvik, T., Grassineau, N., & Skovgaard, A. C., 2010. Compositional characteristics and spatial distribution of enriched Icelandic mantle components, *Journal of Petrology*, **51**(7), 1447–1475.

[782] Peccerillo, A., 2005. *Plio-quaternary volcanism in Italy*, Springer, The Netherlands.

[783] Peccerillo, A., Berberio, M. R., Yirgu, G., Ayalew, D., Barbieri, M., & Wu, T. W., 2003. Relationships between Mafic and Peralkaline Silicic Magmatism in Continental Rift Settings: a Petrological, Geochemical and Isotopic Study of the Gedemsa Volcano, Central Ethiopian Rift, *Journal of Petrology*, **44**(11), 2003–2032.

[784] Pelleter, A. A., Caroff, M., Cordier, C., Bachelery, P., Nehlig, P., Debeuf, D., & Arnaud, N., 2014. Melilite-bearing lavas in Mayotte (France): An insight into the mantle source below the Comores, *Lithos*, **208**, 281–297.

[785] Peng, Z. C., Zartman, R. E., Futa, K., & Chen, D. G., 1986. Pb-, Sr- and Nd-isotopic systematics and chemical characteristics of Cenozoic basalts, Eastern China, *Chemical Geology*, **59**, 3–33.

[786] Perepelov, A. B., Tsypukova, S. S., Demonterova, E. I., Pavlova, L. A., Travin, A. V., & Bat-Ulzii, D., 2010. The first mineralogical, geochemical, and isotope-geochronological data on neogene alkaline basaltic volcanism of the Heven Zalu Uriin Sar'dag Plateau (Northern Mongolia), *Doklady Earth Sciences*, **434**(1), 1230–1234.

[787] Peters, B. J., Day, J. M., & Taylor, L. A., 2016. Early mantle heterogeneities in the Réunion hotspot source inferred from highly siderophile elements in cumulate xenoliths, *Earth and Planetary Science Letters*, **448**, 150–160.

[788] Petrone, C. M., Francalanci, L., Carlson, R. W., Ferrari, L., & Conticelli, S., 2003. Unusual coexistence of subduction-related and intraplate-type magmatism: Sr, Nd and Pb isotope and trace element data from the magmatism of the San Pedro-Ceboruco graben (Nayarit, Mexico), *Chemical Geology*, **193**(1-2), 1–24.

[789] Phillips, E. H., Sims, K. W., Blichert-Toft, J., Aster, R. C., Gaetani, G. A., Kyle, P. R., Wallace, P. J., & Rasmussen, D. J., 2018. The nature and evolution of mantle upwelling at Ross Island, Antarctica, with implications for the source of HIMU lavas, *Earth and Planetary Science Letters*, **498**, 38–53.

[790] Pik, R., Deniel, C., Coulon, C., Yirgu, G., & Marty, B., 1999. Isotopic and trace element signatures of Ethiopian flood basalts: Evidence for plume-lithosphere interactions, *Geochimica et Cosmochimica Acta*, **63**(15), 2263–2279.

[791] Pilet, S., Hernandez, J., Sylvester, P., & Poujol, M., 2005. The metasomatic alternative for ocean island basalt chemical heterogeneity, *Earth and Planetary Science Letters*, **236**(1-2), 148–166.

[792] Pilet, S., Baker, M. B., Müntener, O., & Stolper, E. M., 2011. Monte Carlo simulations of metasomatic enrichment in the lithosphere and implications for the source of alkaline basalts, *Journal of Petrology*, **52**(7-8), 1415–1442.

[793] Platz, T., Foley, S. F., & André, L., 2004. Low-pressure fractionation of the Nyiragongo volcanic rocks, Virunga Province, D.R. Congo, *Journal of Volcanology and Geothermal Research*, **136**(3-4), 269–295.

[794] Polat, A., Kerrich, R., & Casey, J. F., 1997. Geochemistry of Quaternary basalts erupted along the East Anatolian and Dead Sea fault zones of southern Turkey: implications for mantle sources, *Lithos*, **40**, 55–68.

[795] Pollock, M., Edwards, B., Hauksdóttir, S., Alcorn, R., & Bowman, L., 2014. Geochemical and lithostratigraphic constraints on the formation of pillow-dominated tindars from Undirhlíar quarry, Reykjanes Peninsula, southwest Iceland, *Lithos*, **200-201**(1), 317–333.

[796] Pouclet, A., Lee, J.-S., Vidal, P., Cousens, B., & Bellon, H., 1995. Cretaceous to Cenozoic volcanism in South Korea and in the Sea of Japan: magmatic constraints on the opening of the back-arc basin, *Geological Society, London, Special Publications*, **81**(1), 169–191.

[797] Pouclet, A., Bellon, H., & Bram, K., 2016. The Cenozoic volcanism in the Kivu rift: Assessment of the tectonic setting, geochemistry, and geochronology of the volcanic activity in the South-Kivu and Virunga regions, *Journal of African Earth Sciences*, **121**, 219–246.

[798] Prægel, N.-O. & Holm, P. M., 2006. Lithospheric contributions to high-MgO basanites from the Cumbre Vieja Volcano, La Palma, Canary Islands and evidence for temporal variation in plume influence, *Journal of volcanology and geothermal research*, **149**(3-4), 213–239.

[799] Prelević, D., Akal, C., Foley, S. F., Romer, R. L., Stracke, A., & van den Bogaard, P., 2012. Ultrapotassic mafic rocks as geochemical proxies for post-collisional dynamics of orogenic lithospheric mantle: the case of southwestern Anatolia, Turkey, *Journal of Petrology*, **53**(5), 1019–1005.

[800] Prestvik, T., Barnes, C. G., Sundvoll, B., & Duncan, R. A., 1990. Petrology of Peter I Øy (Peter I Island), West Antarctica, *Journal of Volcanology and Geothermal Research*, **44**(3-4), 315–338.

[801] Prestvik, T., Goldberg, S., Karlsson, H., & Grönvold, K., 2001. Anomalous strontium and lead isotope signatures in the off-rift Öræfajökull central volcano in south-east iceland evidence for enriched endmember(s) of the iceland mantle plume?, *Earth and Planetary Science Letters*, **190**(3-4), 211–220.

[802] Price, R., Gray, C., & Frey, F., 1997. Strontium isotopic and trace element heterogeneity in the plains basalts of the Newer Volcanic Province, Victoria, Australia, *Geochimica et Cosmochimica Acta*, **61**(1), 171–192.

[803] Price, R. C., Gray, C. M., Wilson, R. E., Frey, F. A., & Taylor, S. R., 1991. The effects of weathering on rare-earth element, Y and Ba abundances in Tertiary basalts from southeastern Australia, *Chemical Geology*, **93**(3-4), 245–265.

[804] Priestley, K., McKenzie, D., & Ho, T., 2019. A lithosphere-asthenosphere boundary – a global model derived from multimode surface-wave tomography and petrology, in *Lithospheric Discontinuities*, edited by H. Yuan & B. Romanowicz, chap. 6, pp. 111–123, American Geophysical Union, Washington, D.C.

[805] Prytulak, J., Avanzinelli, R., Koetsier, G., Kreissig, K., Beier, C., & Elliott, T., 2014. Melting versus contamination effects on ^{238}U–^{230}Th–^{226}Ra and ^{235}U–^{231}Pa disequilibria in lavas from São Miguel, Azores, *Chemical Geology*, **381**, 94–109.

[806] Puzankov, Y. M. & Bobrov, V., 1997. Geochemistry of the volcanic rocks from Easter and Sala y Gomez islands, *Geochemistry International*, **35**(7), 609–619.

[807] Quane, S. L., Garcia, M. O., Guillou, H., & Hulsebosch, T. P., 2000. Magmatic history of the East Rift Zone of Kilauea Volcano, Hawaii based on drill core from SOH 1, *Journal of Volcanology and Geothermal Research*, **102**(3-4), 319–338.

[808] Radivojević, M., Toljić, M., Turki, S. M., Bojić, Z., Šarić, K., & Cvetković, V., 2015. Neogene to Quaternary basalts of the Jabal Eghei (Nuqay) area (south Libya): Two distinct volcanic events or continuous volcanism with gradual shift in magma composition?, *Journal of Volcanology and Geothermal Research*, **293**, 57–74.

[809] Ramos, V. A. & Kay, S. M., 1992. Southern Patagonian plateau basalts and deformation: Backarc testimony of ridge collisions, *Tectonophysics*, **205**(1-3), 261–282.

[810] Rankenburg, K., Lassiter, J. C., & Brey, G., 2005. The role of continental crust and lithospheric mantle in the genesis of Cameroon Volcanic Line lavas: Constraints from isotopic variations in lavas and megacrysts from the Biu and Jos Plateaux, *Journal of Petrology*, **46**(1), 169–190.

[811] Rasskazov, S., Ivanov, A., Boven, A., & André, L., 1997. Late Cenozoic reactivation of the Early Pre-Cambrian Aldan Shield: trace element constraints on magmatic sources beneath the Udocan ridge, Siberia, Russia, in *Proc. 30th Int. Geol. Cong*, pp. 153–167.

[812] Rasskazov, S., Saranina, E., Demonterova, E., Maslovskaya, M., & Ivanov, A., 2002. Mantle components in Late Cenozoic volcanics of the East Sayan (from Pb, Sr, and Nd isotopes), *Geologia and Geophysica*, **43**(12), 1065–1079.

[813] Rasskazov, S. V., Boven, A., & Andre, L., 1997. Evolution of magmatism in the Northeastern Baikal rift system, *Petrologiya*, **5**(2), 115–136.

[814] Rasskazov, S. V., Chuvashova, I. S., Liu, Y., Meng, F., Yasnygina, T. A., Fefelov, N. N., & Saranina, E. V., 2011. Proportions of lithospheric and asthenospheric components in Late Cenozoic K and K-Na lavas in Heilongjiang Province, Northeastern China, *Petrology*, **19**(6), 568–600.

[815] Ray, J. S., Mahoney, J. J., Duncan, R. A., Ray, J., Wessel, P., & Naar, D. F., 2012. Chronology and geochemistry of lavas from the Nazca Ridge and Easter Seamount Chain: an 30 Myr hotspot record, *Journal of Petrology*, **53**(7), 1417–1448.

[816] Reid, M. R., Schleiffarth, W. K., Cosca, M. A., Delph, J. R., Blichert-Toft, J., & Cooper, K. M., 2017. Shallow melting of MORB-like mantle under hot continental lithosphere, Central Anatolia, *Geochemistry, Geophysics, Geosystems*, **18**, 1866–1888.

[817] Reiners, P. W. & Nelson, B. K., 1998. Temporal-compositional-isotopic trends in rejuvenated-stage magmas of Kauai, Hawaii, and implications for mantle melting processes, *Geochimica et Cosmochimica Acta*, **62**(13), 2347–2368.

[818] Reinhard, A., Jackson, M., Harvey, J., Brown, C., & Koornneef, J., 2016. Extreme differences in 87Sr/86Sr between Samoan lavas and the magmatic olivines they host: Evidence for highly heterogeneous 87Sr/86Sr in the magmatic plumbing system sourcing a single lava, *Chemical Geology*, **439**, 120–131.

[819] Révillon, S., Arndt, N. T., Hallot, E., Kerr, A. C., & Tarney, J., 1999. Petrogenesis of picrites from the Caribbean Plateau and the North Atlantic magmatic province, *Lithos*, **49**(1-4), 1–21.

[820] Reyes, J., Lara, L. E., Hauff, F., Hoernle, K., Morata, D., Selles, D., & Cooper, O., 2019. Petrogenesis of shield volcanism from the Juan Fernández Ridge, Southeast Pacific: Melting of a low-temperature pyroxenite-bearing mantle plume, *Geochimica et Cosmochimica Acta*, **257**, 311–335.

[821] Rhodes, J. M., 1983. Homogeneity of lava flows: chemical data for historic Mauna Loan eruptions, *Journal of Geophysical Research: Solid Earth*, **88**(S02), A869–A879.

[822] Rhodes, J. M., 1996. Geochemical stratigraphy of lava flows sampled by the Hawaii Scientific Drilling Project, *Journal of Geophysical Research: Solid Earth*, **101**(B5), 11729–11746.

[823] Rhodes, J. M. & Hart, S. R., 1995. Episodic trace element and isotopic variations in historical mauna loa lavas: Implications for magma and plume dynamics, *Geophysical Monograph Series*, **92**, 263–288.

[824] Rhodes, J. M. & Vollinger, M. J., 2004. Composition of basaltic lavas sampled by phase-2 of the Hawaii Scientific Drilling Project: Geochemical stratigraphy and magma types, *Geochemistry, Geophysics, Geosystems*, **5**(3).

[825] Richardson-Bunbury, J. M., 1992. *Basalts of Kula and their relation to extension in western Turkey*, Ph.D. thesis, University of Cambridge.

[826] Righter, K. & Carmichael, I. S., 1992. Hawaiites and related lavas in the Atenguillo Graben, western Mexican Volcanic Belt, *Geological Society of America Bulletin*, **104**(12), 1592–1607.

[827] Righter, K. & Rosas-Elguera, J., 2001. Alkaline Lavas in the Volcanic Front of the Western Mexican Volcanic Belt: Geology and Petrology of the Ayutla and Tapalpa Volcanic Fields, *Journal of Petrology*, **42**(12), 2333–2361.

[828] Righter, K., Carmichael, I. S., Becker, T. A., & Renne, P. R., 1995. Pliocene-Quaternary volcanism and faulting at the intersection of the Gulf of California and the Mexican Volcanic Belt, *Geological Society of America Bulletin*, **107**(5), 612–626.

[829] Riisager, P., Knight, K. B., Baker, J. A., Peate, I. U., Al-Kadasi, M., Al-Subbary, A., & Renne, P. R., 2005. Paleomagnetism and $^{40}Ar/^{39}Ar$ Geochronology of Yemeni Oligocene volcanics: Implications for timing and duration of Afro-Arabian traps and geometry of the Oligocene paleomagnetic field, *Earth and Planetary Science Letters*, **237**(3-4), 647–672.

[830] Ritsema, J., Deuss, A., Van Heijst, H., & Woodhouse, J., 2011. S40RTS: a degree-40 shear-velocity model for the mantle from new Rayleigh wave dispersion, teleseismic traveltime and normal-mode splitting function measurements, *Geophysical Journal International*, **184**(3), 1223–1236.

[831] Rivalenti, G., Zanetti, A., Girardi, V. A., Mazzucchelli, M., Tassinari, C. C., & Bertotto, G. W., 2007. The effect of the Fernando de Noronha plume on the mantle lithosphere in north-eastern Brazil, *Lithos*, **94**(1-4), 111–131.

[832] Roden, M. K., Hart, S. R., Frey, F. A., & Melson, W. G., 1984. Sr, Nd and Pb isotopic and REE geochemistry of St. Paul's Rocks: the metamorphic and metasomatic development of an alkali basalt mantle source, *Contributions to Mineralogy and Petrology*, **85**, 376–390.

[833] Roeder, P. L., Thornber, C., Poustovetov, A., & Grant, A., 2003. Morphology and composition of spinel in Pu'u 'O'o lava (1996-1998), Kilauea volcano, Hawaii, *Journal of Volcanology and Geothermal Research*, **123**(3-4), 245–265.

[834] Roex, A. P., Späth, A., & Zartman, R. E., 2001. Lithospheric thickness beneath the southern Kenya Rift: implications from basalt geochemistry, *Contributions to Mineralogy and Petrology*, **142**(1), 89–106.

[835] Rogers, N., Macdonald, R., Fitton, J. G., George, R., Smith, M., & Barreiro, B., 2000. Two mantle plumes beneath the East African rift system: Sr, Nd and Pb isotope evidence from Kenya Rift basalts, *Earth and Planetary Science Letters*, **176**, 387–400.

[836] Rogers, N., Thomas, L., Macdonald, R., Hawkesworth, C., & Mokadem, F., 2006. ^{238}U–^{230}Th disequilibrium in recent basalts and dynamic melting beneath the Kenya rift, *Chemical Geology*, **234**(1-2), 148–168.

[837] Rogers, N. W., De Mulder, M., & Hawkesworth, C. J., 1992. An enriched mantle source for potassic basanites: evidence from Karisimbi volcano, Virunga volcanic province, Rwanda, *Contributions to Mineralogy and Petrology*, **111**(4), 543–556.

[838] Rogers, N. W., James, D., Kelley, S. P., & De Mulder, M., 1998. The generation of potassic lavas from the eastern Virunga province, Rwanda, *Journal of Petrology*, **39**(6), 1223–1247.

[839] Ronga, F., Lustrino, M., Marzoli, A., & Melluso, L., 2010. Petrogenesis of a basalt-comendite-pantellerite rock suite: The Boseti Volcanic Complex (Main Ethiopian Rift), *Mineralogy and Petrology*, **98**(1-2), 227–243.

[840] Rooney, T., Furman, T., Bastow, I., Ayalew, D., & Yirgu, G., 2007. Lithospheric modification during crustal extension in the Main Ethiopian Rift, *Journal of Geophysical Research: Solid Earth*, **112**(10).

[841] Rooney, T. O., 2010. Geochemical evidence of lithospheric thinning in the southern Main Ethiopian Rift, *Lithos*, **117**(1-4), 33–48.

[842] Rooney, T. O., 2017. The Cenozoic magmatism of East-Africa: Part I — Flood basalts and pulsed magmatism, *Lithos*, **286-287**, 264–301.

[843] Rooney, T. O., Furman, T., Yirgu, G., & Ayalew, D., 2005. Structure of the Ethiopian lithosphere: Xenolith evidence in the Main Ethiopian Rift, *Geochimica et Cosmochimica Acta*, **69**(15), 3889–3910.

[844] Rooney, T. O., Hanan, B. B., Graham, D. W., Furman, T., Blichert-Toft, J., & Schilling, J.-G., 2011. Upper mantle pollution during Afar plume-continental rift interaction, *Journal of Petrology*, **53**(2), 365–389.

[845] Rooney, T. O., Mohr, P., Dosso, L., & Hall, C., 2013. Geochemical evidence of mantle reservoir evolution during progressive rifting along the western Afar margin, *Geochimica et Cosmochimica Acta*, **102**, 65–88.

[846] Rooney, T. O., Nelson, W. R., Dosso, L., Furman, T., & Hanan, B., 2014. The role of continental lithosphere metasomes in the production of HIMU-like magmatism on the northeast African and Arabian plates, *Geology*, **42**(5), 419–422.

[847] Rosenthal, A., Foley, S., Pearson, D. G., Nowell, G. M., & Tappe, S., 2009. Petrogenesis of strongly alkaline primitive volcanic rocks at the propagating tip of the western branch of the East African Rift, *Earth and Planetary Science Letters*, **284**(1-2), 236–248.

[848] Rossi, P., Tranne, C., Calanchi, N., & Lanti, E., 1996. Geology, stratigraphy and volcanological evolution of the island of Linosa (Sicily Channel), *Acta Vulcanologica*, **8**, 73–90.

[849] Rotolo, S., Castorina, F., Cellura, D., & Pompilio, M., 2006. Petrology and geochemistry of submarine volcanism in the Sicily Channel Rift, *The Journal of geology*, **114**(3), 355–365.

[850] Russell, J. K. & Hauksdóttir, S., 2001. Estimates of crustal assimilation in Quaternary lavas from the Northern Cordillera, British Columbia, *Canadian Mineralogist*, **39**(2), 275–297.

[851] Rutter, M. J., 1987. The nature of the lithosphere beneath the Sardinian continental block: mantle and deep crustal inclusions in mafic alkaline lavas, *Lithos*, **20**, 225–234.

[852] Saadat, S. & Stern, C. R., 2011. Petrochemistry and genesis of olivine basalts from small monogenetic parasitic cones of Bazman stratovolcano, Makran arc, southeastern Iran, *Lithos*, **125**(1-2), 607–619.

[853] Saadat, S., Stern, C. R., & Moradian, A., 2014. Petrochemistry of ultrapotassic tephrites and associated cognate plutonic xenoliths with carbonatite affinities from the late Quaternary Qa'le Hasan Ali maars, central Iran, *Journal of Asian Earth Sciences*, **89**, 108–122.

[854] Saal, A. E., Kurz, M. D., Hart, S. R., Blusztajn, J. S., Blichert-Toft, J., Liang, Y., & Geist, D. J., 2007. The role of lithospheric gabbros on the composition of Galapagos lavas, *Earth and Planetary Science Letters*, **257**(3-4), 391–406.

[855] Sakuyama, T., Nagaoka, S., Miyazaki, T., Chang, Q., Takahashi, T., Hirahara, Y., Senda, R., Itaya, T., Kimura, J., & Ozawa, K., 2014. Melting of the uppermost metasomatized asthenosphere triggered by fluid fluxing from ancient subducted sediment: Constraints from the quaternary basalt lavas at Chugaryeong Volcano, Korea, *Journal of Petrology*, **55**(3), 499–528.

[856] Salaün, A., Villemant, B., Semet, M., & Staudacher, T., 2010. Cannibalism of olivine-rich cumulate xenoliths during the 1998 eruption of Piton de la Fournaise (La Réunion hotspot): Implications for the generation of magma diversity, *Journal of Volcanology and Geothermal Research*, **198**(1-2), 187–204.

[857] Santo, A. P., Capaccioni, B., Tedesco, D., & Vaselli, O., 2003. Petrographic and geochemical features of the 2002 Nyiragongo lava flows, *Acta Vulcanologica*, **14**(1/2), 63.

[858] Savatenkov, V. M., Yarmolyuk, V. V., Kudryashova, E. A., & Kozlovskii, A. M., 2010. Sources and geodynamics of the Late Cenozoic volcanism of Central Mongolia: Evidence from isotope-geochemical studies, *Petrology*, **18**(3), 278–307.

[859] Schaeffer, A. J. & Lebedev, S., 2013. Global shear speed structure of the upper mantle and transition zone, *Geophysical Journal International*, **194**(1), 417–449.

[860] Schiano, P., David, K., Vlastélic, I., Gannoun, A., Klein, M., Nauret, F., & Bonnand, P., 2012. Osmium isotope systematics of historical lavas from Piton de la Fournaise (Réunion Island, Indian Ocean), *Contributions to Mineralogy and Petrology*, **164**(5), 805–820.

[861] Schiellerup, H., 1995. Generation and equilibration of olivine tholeiites in the northern rift zone of Iceland. A petrogenetic study of the Bláfjall table mountain, *Journal of Volcanology and Geothermal Research*, **65**(3-4), 161–179.

[862] Schneider, B., Kuiper, K. F., Mai, K., Foeken, J. P., Stuart, F. M., & Wijbrans, J. R., 2014. Fuerteventura - Assessment of a calibration site for cosmogenic ^{3}He exposure dating with the ^{40}Ar/^{39}Ar incremental heating method, *Quaternary Geochronology*, **21**(1), 58–69.

[863] Schubert, S., Jung, S., Pfänder, J. A., Hauff, F., & Garbe-Schönberg, D., 2015. Petrogenesis of Tertiary continental intra-plate lavas between Siebengebirge and Westerwald, Germany: Constraints from trace element systematics and Nd, Sr and Pb isotopes, *Journal of Volcanology and Geothermal Research*, **305**, 84–99.

[864] Schwarz, S., Klügel, A., van den Bogaard, P., & Geldmacher, J., 2005. Internal structure and evolution of a volcanic rift system in the eastern North Atlantic: The Desertas rift zone, Madeira archipelago, *Journal of Volcanology and Geothermal Research*, **141**(1-2), 123–155.

[865] Scott, J. M., Turnbull, I. M., Auer, A., & Palin, J. M., 2013. The sub-Antarctic Antipodes Volcano: A <0.5 Ma HIMU-like Surtseyan volcanic outpost on the edge of the Campbell Plateau, New Zealand, *New Zealand Journal of Geology and Geophysics*, **56**(3), 134–153.

[866] Searle, M. P., Chung, S.-L., & Lo, C.-H., 2010. Geological offsets and age constraints along the northern Dead Sea fault, Syria, *Journal of the Geological Society*, **167**(5), 1001–1008.

[867] Sebai, A., Zumbo, V., Féraud, G., Bertrand, H., Hussain, A. G., Giannérini, G., & Campredon, R., 1991. ^{40}Ar/^{39}Ar dating of alkaline and tholeiitic magmatism of Saudi Arabia related to the early Red Sea Rifting, *Earth and Planetary Science Letters*, **104**(2-4), 473–487.

[868] Seghedi, I., Downes, H., Vaselli, O., Szakács, A., Balogh, K., & Pécskay, Z., 2004. Post-collisional Tertiary-Quaternary mafic alkalic magmatism in the Carpathian-Pannonian region: A review, *Tectonophysics*, **393**(1-4 SPEC.ISS.), 43–62.

[869] Sérgio de Souza, Z., Leite do Nascimento, M. A., Nunes Barbosa, R. V., & Gustavo da Silveira Dias, L., 2005. Geology and tectonics of the Boa Vista Basin (Paraíba, northeastern Brazil) and geochemistry of associated Cenozoic tholeiitic magmatism, *Journal of South American Earth Sciences*, **18**(3-4 SPEC. ISS.), 391–405.

[870] Shaanan, U., Porat, N., Navon, O., Weinberger, R., Calvert, A., & Weinstein, Y., 2011. OSL dating of a Pleistocene maar: Birket Ram, the Golan heights, *Journal of Volcanology and Geothermal Research*, **201**(1-4), 397–403.

[871] Sharkov, E. V., Chernyshev, I. V., Devyatkin, E. V., Dodonov, A. E., Ivanenko, V. V., Karpenko, M. I., Leonov, Y. G., Novikov, V. M., Hanna, S., & Khatib, K., 1994. Geochronology of Late Cenozoic Basalts in Western Syria, *Petrology*, **2**(4), 385–394.

[872] Sharkov, E. V., Chernyshev, I. V., Devyatkin, E. V., Dodonov, A. E., Ivanenko, V. V., Karpenko, M. I., Lebedev, V. A., Novikov, V. M., Hanna, S., & Khatib, K., 1998. New Data on the Geochronology of Upper Cenozoic Plateau Basalts from the Northeastern Periphery of the Red Sea Rift Area (Northern Syria) New Data on the Geochronology of Upper Cenozoic Plateau Basalts from the Northeastern Periphery of the Red Sea Rift, *Geology*, **358**, 19–22.

[873] Shaw, J. E., Baker, J. A., Menzies, M. A., Thirlwall, M. F., & Ibrahim, K. M., 2003. Petrogenesis of the Largest Intraplate Volcanic Field on the Arabian Plate (Jordan): a Mixed Lithosphere-Asthenosphere Source Activated by Lithospheric Extension, *Journal of Petrology*, **44**(9), 1657–1679.

[874] Sheth, H. C., Mahoney, J. J., & Baxter, A. N., 2003. Geochemistry of Lavas from Mauritius, Indian Ocean: Mantle Sources and Petrogenesis, *International Geology Review*, **45**(9), 780–797.

[875] Shinjo, R., Chekol, T., Meshesha, D., Itaya, T., & Tatsumi, Y., 2011. Geochemistry and geochronology of the mafic lavas from the southeastern Ethiopian rift (the East African Rift System): Assessment of models on magma sources, plume-lithosphere interaction and plume evolution, *Contributions to Mineralogy and Petrology*, **162**(1), 209–230.

[876] Shorttle, O., Maclennan, J., & Piotrowski, A. M., 2013. Geochemical provincialism in the Iceland plume, *Geochimica et Cosmochimica Acta*, **122**, 363–397.

[877] Shrbenỳ, O., 1980. Chemical composition of the alkaline neovolcanics of the Krušnéhory Mountains, *Bohemia. Věstník Ústředního Ústavu geologického*, **55**, 1–10.

[878] Siebel, W., Becchio, R., Volker, F., Hansen, M. A. F., Viramonte, J., Trumbull, R. B., Haase, G., & Zimmer, M., 2000. Trindade and Martin Vaz Islands, South Atlantic: Isotopic (Sr, Nd, Pb) and trace element constraints on plume related magmatism, *Journal of South American Earth Sciences*, **13**(1-2), 79–103.

[879] Siebert, L. & Carrasco-Núñez, G., 2002. Late-Pleistocene to precolumbian behind-the-arc mafic volcanism in the eastern Mexican Volcanic Belt; implications for future hazards, *Journal of Volcanology and Geothermal Research*, **115**(1-2), 179–205.

[880] Sigmarsson, O., Condomines, M., & Fourcade, S., 1992. A detailed Th, Sr and O isotope study of Hekla: differentiation processes in an Icelandic Volcano, *Contributions to Mineralogy and Petrology*, **112**(1), 20–34.

[881] Sigurdsson, I. A., Steinthorsson, S., & Grönvold, K., 2000. Calcium-rich melt inclusions in Cr-spinels from Borgarhraun, northern Iceland, *Earth and Planetary Science Letters*, **183**(1-2), 15–26.

[882] Silveira, F. V., 2006. *Magmatismo cenozóico da porção central do Rio Grande do Norte , NE do Brasil*, Ph.D. thesis, Universidade Federal Do Rio Grande Do Norte.

[883] Simonetti, A. & Bell, K., 1994. Nd, Pb and Sr isotopic data from the Napak carbonatite-nephelinite centre, eastern Uganda: an example of open-system crystal fractionation, *Contributions to Mineralogy and Petrology*, **115**(3), 356–366.

[884] Simonetti, A. & Bell, K., 1995. Nd, Pb and Sr isotopic data from the Mount Elgon volcano, eastern Uganda-western Kenya: Implications for the origin and evolution of nephelinite lavas, *Lithos*, **36**(2), 141–153.

[885] Simonov, V. A., Kudryashova, E. A., Yarmolyuk, V. V., Kovyazin, S. V., & Kotlyarov, A. V., 2013. Petrogenesis of late cenozoic basaltic complexes in the southern Baikal and southern Khangai volcanic areas in central Asia: Evidence from melt inclusions, *Petrology*, **21**(5), 489–506.

[886] Sims, K. W., Maclennan, J., Blichert-Toft, J., Mervine, E. M., Blusztajn, J., & Grönvold, K., 2013. Short length scale mantle heterogeneity beneath Iceland probed by glacial modulation of melting, *Earth and Planetary Science Letters*, **379**, 146–157.

[887] Sinton, C. W., Hauff, F., Hoernle, K., & Werner, R., 2018. Age progressive volcanism opposite Nazca plate motion: Insights from seamounts on the northeastern margin of the Galapagos Platform, *Lithos*, **310**, 342–354.

[888] Skovgaard, A. C., Storey, M., Baker, J., Blusztajn, J., & Hart, S. R., 2001. Osmium-oxygen isotopic evidence for a recycled and strongly depleted component in the Iceland mantle plume, *Earth and Planetary Science Letters*, **194**(1-2), 259–275.

[889] Slater, L., Jull, M., McKenzie, D., & Gronvöld, K., 1998. Deglaciation effects on mantle melting under Iceland: Results from the northern volcanic zone, *Earth and Planetary Science Letters*, **164**(1-2), 151–164.

[890] Slater, L., McKenzie, D., Grönvold, K., & Shimizu, N., 2001. Melt Generation and Movement beneath Theistareykir, NE Iceland, *Journal of Petrology*, **42**(2), 321–354.

[891] Sobolev, A. V. & Nikogosian, I. K., 1994. Petrology of long-lived mantle plume magmatism: Hawaii, Pacific, and Reunion Island, Indian Ocean, *Petrology*, **v. 2**(no. 2), p. 111–144.

[892] Sohn, Y. K., Cronin, S. J., Brenna, M., Smith, I. E., Németh, K., White, J. D., Murtagh, R. M., Jeon, Y. M., & Kwon, C. W., 2012. Ilchulbong tuff cone, Jeju Island, Korea, revisited: A compound monogenetic volcano involving multiple magma pulses, shifting vents, and discrete eruptive phases, *Bulletin of the Geological Society of America*, **124**(3-4), 259–274.

[893] Späth, A., Le Roex, A. P., & Opiyo-Akech, N., 2000. The petrology of the Chyulu Hills Volcanic Province, southern Kenya, *Journal of African Earth Sciences*, **31**(2), 337–358.

[894] Späth, A., Le Roex, A. P., & Opiyo-akech, N., 2001. Plume-Lithosphere Interaction and the Origin of Continental Rift-related Alkaline Volcanism – the Chyulu Hills Volcanic Province, Southern Kenya, *Journal of Petrology*, **42**(4), 765–787.

[895] Späth, A., Le Roex, A. P., & Duncan, R. A., 2007. The Geochemistry of Lavas from the Gomores Archipelago, Western Indian Ocean: Petrogenesis and Mantle Source Region Characteristics, *Journal of Petrology*, **37**(4), 961–991.

[896] Spengler, S. R. & Garcia, M. O., 1988. Geochemistry of the Hawi lavas, Kohala Volcano, Hawaii, *Contributions to Mineralogy and Petrology*, **99**(1), 90–104.

[897] Standish, J., Geist, D., Harpp, K., & Kurz, M. D., 1998. The emergence of a Galapagos shield volcano, Roca Redonda, *Contributions to Mineralogy and Petrology*, **133**(1-2), 136–148.

[898] Stein, M. & Hofmann, A. W., 1992. Fossil plume head beneath the Arabian lithosphere?, *Earth and Planetary Science Letters*, **114**(1), 193–209.

[899] Steinitz, G., Bartov, Y., & Hunziker, J. C., 1978. K-Ar age determinations of some Miocene–Pliocene basalts in Israel: Their significance to the tectonics of the Rift Valley, *Geological Magazine*, **115**(5), 329–340.

[900] Stephenson, P., Burch-Johnston, A., Stanton, D., & Whitehead, P., 1998. Three long lava flows in north Queensland, *Journal of Geophysical Research: solid earth*, **103**(B11), 27359–27370.

[901] Stephenson, P., Zhang, M., & Spry, M., 2000. Fractionation modelling of segregations in the Toomba Basalt, north Queensland, *Australian Journal of Earth Sciences*, **47**(2), 291–300.

[902] Stephenson, S. N., 2019. *Dynamic Topography of Madagascar and its Surroundings*, Ph.D. thesis, University of Cambridge.

[903] Stern, C. R., Frey, F. A., Futa, K., Zartman, R. E., Peng, Z., & Kurtis Kyser, T., 1990. Trace-element and Sr, Nd, Pb, and O isotopic composition of Pliocene and Quaternary alkali basalts of the Patagonian Plateau lavas of southernmost South America, *Contributions to Mineralogy and Petrology*, **104**(3), 294–308.

[904] Stewart, K. & Rogers, N., 1996. Mantle plume and lithosphere contributions to basalts from southern Ethiopia, *Earth and Planetary Science Letters*, **139**(1-2), 195–211.

[905] Stoffers, P., Botz, R., Cheminée, J.-L., Devey, C. W., Froger, V., Glasby, G., Hartmann, M., Hekinian, R., Kögler, F., Laschek, D.,*et al.*, 1989. Geology of MacDonald seamount region, Austral Islands: recent hotspot volcanism in the South Pacific, *Marine Geophysical Researches*, **11**(2), 101–112.

[906] Stolper, E., Sherman, S., Garcia, M., Baker, M., & Seaman, C., 2004. Glass in the submarine section of the HSDP2 drill core, Hilo, Hawaii, *Geochemistry, Geophysics, Geosystems*, **5**(7).

[907] Stolz, A., 1985. The role of fractional crystallization in the evolution of the Nandewar Volcano, north-eastern New South Wales, Australia, *Journal of Petrology*, **26**(4), 1002–1026.

[908] Stone, W. E. & Fleet, M. E., 1991. Nickel-copper sulphides from the 1959 eruption of Kilauea volcano, Hawaii; contrasting compositions and phase relations in eruption pumice and Kilauea Iki lava lake, *American Mineralogist*, **76**(7-8), 1363–1372.

[909] Stoppa, F. & Schiazza, M., 2013. An overview of monogenetic carbonatitic magmatism from Uganda, Italy, China and Spain: Volcanologic and geochemical features, *Journal of South American Earth Sciences*, **41**, 140–159.

[910] Storey, M., Saunders, A., Tarney, J., Leat, P., Thirlwall, M., Thompson, R., Menzies, M., & Marriner, G., 1988. Geochemical evidence for plume-mantle interactions beneath Kerguelen and Heard Islands, Indian Ocean, *Nature*, **336**(6197), 371.

[911] Stracke, A., Zindler, A., Salters, V. J., McKenzie, D., Janne, B. T., Albarède, F., & Grönvold, K., 2003. Theistareykir revisited, *Geochemistry, Geophysics, Geosystems*, **4**(2).

[912] Straub, S. M., Gómez-tuena, A., Zellmer, G. F., Espinasa-perena, R., Stuart, F. M., Cai, Y., Langmuir, C. H., Martin-del pozzo, A. L., & Mesko, G. T., 2013. The processes of melt differentiation in arc volcanic rocks: Insights from OIB-type arc magmas in the central mexican volcanic belt, *Journal of Petrology*, **54**(4), 665–701.

[913] Suh, C., Luhr, J. F., & Njome, M., 2008. Olivine-hosted glass inclusions from Scoriae erupted in 1954–2000 at Mount Cameroon volcano, West Africa, *Journal of Volcanology and Geothermal Research*, **169**(1-2), 1–33.

[914] Suh, C. E., Sparks, R. S., Fitton, J. G., Ayonghe, S. N., Annen, C., Nana, R., & Luckman, A., 2003. The 1999 and 2000 eruptions of Mount Cameroon: Eruption behaviour and petrochemistry of lava, *Bulletin of Volcanology*, **65**(4), 267–281.

[915] Sun, P., Niu, Y., Guo, P., Ye, L., Liu, J., & Feng, Y., 2017. Elemental and Sr–Nd–Pb isotope geochemistry of the Cenozoic basalts in Southeast China: Insights into their mantle sources and melting processes, *Lithos*, **272-273**, 16–30.

[916] Sutherland, F. L., 2003. Eastern Australian rift margins and the Indian-Pacific mantle boundary, *Evolution and dynamics of the Australian Plate*, **372**, 203.

[917] Sutherland, F. L., Stubbs, D., & Green, D. C., 1977. K-Ar ages of Cainozoic volcanic suites, Bowen-St Lawrence Hinterland, North Queensland (with some implications for petrologic models), *Journal of the Geological Society of Australia*, **24**(7-8), 447–460.

[918] Sutherland, F. L., Graham, I. T., Pogson, R. E., Schwarz, D., Webb, G. B., Coenraads, R. R., Fanning, C. M., Hollis, J. D., Allen, T. C.,*et al.*, 2002. The Tumbarumba basaltic gem field, New South Wales: In relation to sapphire-ruby deposits of eastern Australia, *Records – Australian Museum*, **54**(2), 215–248.

[919] Tagami, T., Nishimitsu, Y., & Sherrod, D. R., 2003. West Maui volcano , Hawaii : new evidence from K–Ar ages and chemistry of Lahaina Volcanics, *Journal of Volcanology and Geothermal Research*, **120**.

[920] Takamasa, A., Nakai, S., Sahoo, Y. V., Hanyu, T., & Tatsumi, Y., 2009. W isotope compositions of oceanic islands basalts from French Polynesia and their meaning for core-mantle interaction, *Chemical Geology*, **260**(1-2), 37–46.

[921] Taneja, R., Rushmer, T., Blichert-Toft, J., Turner, S., & O'Neill, C., 2016. Mantle heterogeneities beneath the Northeast Indian Ocean as sampled by intra-plate volcanism at Christmas Island, *Lithos*, **262**(March 2018), 561–575.

[922] Tang, Y. J., Zhang, H. F., & Ying, J. F., 2006. Asthenosphere-lithospheric mantle interaction in an extensional regime: Implication from the geochemistry of Cenozoic basalts from Taihang Mountains, North China Craton, *Chemical Geology*, **233**(3-4), 309–327.

[923] Tanguy, J. C., Condomines, M., & Kieffer, G., 1997. Evolution of the Mount Etna magma: Constraints on the present feeding system and eruptive mechanism, *Journal of Volcanology and Geothermal Research*, **75**(3-4), 221–250.

[924] Tappe, S., Foley, S., & Pearson, D., 2003. The kamafugites of Uganda: a mineralogical and geochemical comparison with their Italian and Brazilian analogues, *Periodico di Mineralogia*, **72**, 51–77.

[925] Tarawneh, K., 2000. *Dating of the Harrat Ash Shaam basalts northeast Jordan: phase 1*, Geological Survey of Israel and the Natural Resources Authority of Jordan.

[926] Tatsumi, Y., Kimura, N., Itaya, T., Koyaguchi, T., & Suwa, K., 1991. Intermittent upwelling of asthenosphere beneath Gregory Rift, Kenya, *Geophysical Research Letters*, **18**(6), 1111–1114.

[927] Tatsumi, Y., Oguri, K., Shimoda, G., Kogiso, T., & Barsczus, H. G., 2000. Contrasting behavior of noble-metal elements during magmatic differentiation in basalts from the Cook Islands, Polynesia, *Geology*, **28**(2), 131–134.

[928] Tatsumi, Y., Shukuno, H., Yoshikawa, M., Chang, Q., Sato, K., & Lee, M. W., 2005. The petrology and geochemistry of volcanic rocks on Jeju Island: Plume magmatism along the Asian continental margin, *Journal of Petrology*, **46**(3), 523–553.

[929] Teasdale, R., Geist, D., Kurz, M., & Harpp, K., 2005. 1998 Eruption at Volcán Cerro Azul, Galápagos Islands: I. syn-eruptive petrogenesis, *Bulletin of Volcanology*, **67**(2), 170–185.

[930] Teklay, M., Asmerom, Y., & Toulkeridis, T., 2005. Geochemical and Sr-Nd isotope ratios in Cenozoic basalts from Eritrea: evidence for temporal evolution from low-Ti tholeiitic to high-Ti alkaline basalts in Afro-Arabian Continental Flood Basalt Province, *Periodico di Mineralogia*, **74**, 167–182.

[931] Teklay, M., Scherer, E. E., Mezger, K., & Danyushevsky, L., 2010. Geochemical characteristics and Sr-Nd-Hf isotope compositions of mantle xenoliths and host basalts from Assab, Eritrea: Implications for the composition and thermal structure of the lithosphere beneath the Afar Depression, *Contributions to Mineralogy and Petrology*, **159**(5), 731–751.

[932] Teklemariam, M., Battaglia, S., Gianelli, G., & Ruggieri, G., 1996. Hydrothermal alteration in the Aluto-Langano geothermal field, Ethiopia, *Geothermics*, **25**(6), 679–702.

[933] Thirlwall, M. F., Jenkins, C., Vroon, P. Z., & Mattey, D. P., 1997. Crustal interaction during construction of ocean islands: Pb-Sr-Nd-O isotope geochemistry of the shield basalts of Gran Canaria, Canary Islands, *Chemical Geology*, **135**(3-4), 233–262.

[934] Thomas, L. E., 1999. *Uranium series, major and trace element geochemistry of lavas from Tenerife and Lanzarote, Canary Islands*, Ph.D. thesis, The Open University.

[935] Thompson, G., Smith, I., & Malpas, J., 2001. Origin of oceanic phonolites by crystal fractionation and the problem of the Daly gap: An example from Rarotonga, *Contributions to Mineralogy and Petrology*, **142**(3), 336–346.

[936] Thompson, R. N., Ottley, C. J., Smith, P. M., Pearson, D., G., Pickin, A. P., Morrison, M. A., Leat, P. T., & Gibson, S. A., 2005. Source of Quaternary alkalic basalts, picrites and basanites of the Potrillo Volcanic Field, New Mexico, USA: lithosphere or convecting mantle?, *Journal of Petrology*, **46**(8), 1603–1643.

[937] Thornber, C. R., 2003. Magma-reservoir processes revealed by geochemistry of the Pu'u'O'o-Kupaianaha eruption, *US Geological Survey, Professional Paper*, **1676**, 121–136.

[938] Thornber, C. R., Sherrod, D. R., Siems, D. F., Heliker, C. C., Meeker, G. P., Oscarson, R. L., & Kauahikaua, J. P., 2002. Whole-rock and glass major-element geochemistry of Kilauea Volcano, Hawaii, near-vent eruptive products: September 1994 through September 2001, Tech. rep., US Geological Survey.

[939] Tian, H.-C., Yang, W., Li, S.-G., Ke, S., & Duan, X.-Z., 2018. Low δ^{26}Mg volcanic rocks of Tengchong in Southwestern China: A deep carbon cycle induced by supercritical liquids, *Geochimica et Cosmochimica Acta*, **240**, 191–219.

[940] Tian, S., Hou, Z., Mo, X., Tian, Y., Zhao, Y., Hou, K., Yang, Z., Hu, W., Li, X., & Zhang, Y., 2020. Lithium isotopic evidence for subduction of the Indian lower crust beneath southern Tibet, *Gondwana Research*, **77**, 168–183.

[941] Tian, S.-H., Yang, Z.-S., Hou, Z.-Q., Mo, X.-X., Hu, W.-J., Zhao, Y., & Zhao, X.-Y., 2017. Subduction of the Indian lower crust beneath southern Tibet revealed by the post-collisional potassic and ultrapotassic rocks in SW Tibet, *Gondwana Research*, **41**, 29–50.

[942] Timm, C., Hoernle, K., van den Bogaard, P., Bindeman, I., & Weaver, S., 2009. Geochemical evolution of intraplate volcanism at Banks Peninsula, New Zealand: Interaction between asthenospheric and lithospheric melts, *Journal of Petrology*, **50**(6), 989–1023.

[943] Tonarini, S., Armienti, P., D'Orazio, M., Innocenti, F., Pompilio, M., & Petrini, R., 1995. Geochemical and isotopic monitoring of Mt. Etna 1989-1993 eruptive activity: bearing on the shallow feeding system, *Journal of Volcanology and Geothermal Research*, **64**(1-2), 95–115.

[944] Tonarini, S., D'Orazio, M., Armienti, P., Innocenti, F., & Scribano, V., 1996. Geochemical features of eastern Sicily lithosphere as probed by Hyblean xenoliths and lavas, *European Journal of Mineralogy*, **8**, 1153–1174.

[945] Trifonov, V. G., Dodonov, A. E., Sharkov, E. V., Golovin, D. I., Chernyshev, I. V., Lebedev, V. A., Ivanova, T. P., Bachmanov, D. M., Rukieh, M., Ammar, O., Minini, H., Al Kafri, A. M., & Ali, O., 2011. New data on the Late Cenozoic basaltic volcanism in Syria, applied to its origin, *Journal of Volcanology and Geothermal Research*, **199**(3-4), 177–192.

[946] Trønnes, R. G., Planke, S., Sundvoll, B., & Imsland, P., 1999. Recent volcanic rocks from Jan Mayen: low-degree melt fractions of enriched northeast Atlantic mantle, *Journal of Geophysical Research*, **104**(B4), 7153–7168.

[947] Trua, T., Esperança, S., & Mazzuoli, R., 1998. The evolution of the lithospheric mantle along the N. African Plate: Geochemical and isotopic evidence from the tholeiitic and alkaline volcanic rocks of the Hyblean plateau, Italy, *Contributions to Mineralogy and Petrology*, **131**(4), 307–322.

[948] Trua, T., Deniel, C., & Mazzuoli, R., 1999. Crustal control in the genesis of Plio-Quaternary bimodal magmatism of the Main Ethiopian Rift: geochemical and isotopic (Sr, Nd, Pb) evidence, *Chemical Geology*, **155**, 201–231.

[949] Trua, T., Serri, G., & Marani, M. P., 2003. Lateral flow of African mantle below the nearby Tyrrhenian plate: Geochemical evidence, *Terra Nova*, **15**(6), 433–440.

[950] Tsypukova, S. S., Perepelov, A. B., Demonterova, E. I., Pavlova, L. A., Travin, A. V., & Puzankov, M. Y., 2014. Origin and evolution of Neogene alkali-basaltic magmas in the southwestern flank of the Baikal rift system (Heven lava plateau, northern Mongolia), *Russian Geology and Geophysics*, **55**(2), 190–215.

[951] Turner, S., Arnaud, N., LIU, J., Rogers, N., Hawkesworth, C., Harris, N., Kelley, S., Van Calsteren, P., & Deng, W., 1996. Post-collision, shoshonitic volcanism on the Tibetan Plateau: implications for convective thinning of the lithosphere and the source of ocean island basalts, *Journal of petrology*, **37**(1), 45–71.

[952] Turner, S., Hawkesworth, C., Rogers, N., & King, P., 1997. U–Th isotope disequilibria and ocean island basalt generation in the Azores, *Chemical Geology*, **139**(1-4), 145–164.

[953] Turner, S., Hoernle, K., Hauff, F., Johansen, T. S., Klügel, A., Kokfelt, T., & Lundstrom, C., 2015. ^{238}U-^{230}Th-^{226}Ra disequilibria constraints on the magmatic evolution of the Cumbre Vieja volcanics on La Palma, Canary Islands, *Journal of Petrology*, **56**(10), 1999–2024.

[954] Turner, S. P., Platt, J. P., George, R. M. M., Kelley, S. P., Pearson, D. G., & Nowell, G. M., 1999. Magmatism associated with orogenic collapse of the Betic–Alboran domain, SE Spain, *Journal of Petrology*, **40**(6), 1011–1036.

[955] Ulrych, J., Pivec, E., Lang, M., & Lloyd, F. E., 2000. Ijolitic segregations in melilite nephelinite of Podhorni vrch volcano, Western Bohemia, *Neues Jahrbuch fur Mineralogie, Abhandlungen*, **175**(September 2000), 317–348.

[956] Ulrych, J., Lloyd, F. E., & Balogh, K., 2003. Age Relations and Geochemical Constraints of Cenozoic Alkaline Volcanic Series in W Bohemia : A Review, *Geolines*, **15**, 168–180.

[957] Ulrych, J., Ackerman, L., Balogh, K., Hegner, E., Jelínek, E., Pécskay, Z., Přichystal, A., Upton, B. G., Zimák, J., & Foltýnová, R., 2013. Plio-Pleistocene basanitic and melilititic series of the Bohemian Massif: K-Ar ages, major/trace element and Sr-Nd isotopic data, *Chemie der Erde*, **73**(4), 429–450.

[958] Ulrych, J., Krmíček, L., Tomek, Č., Lloyd, F. E., Ladenberger, A., Ackerman, L., & Balogh, K., 2016. Petrogenesis of Miocene alkaline volcanic suites from western Bohemia: Whole rock geochemistry and Sr-Nd-Pb isotopic signatures, *Chemie der Erde*, **76**(1), 77–93.

[959] Van Otterloo, J., Raveggi, M., Cas, R. A., & Maas, R., 2014. Polymagmatic activity at the monogenetic Mt Gambier volcanic complex in the Newer Volcanics Province, SE Australia: New insights into the occurrence of intraplate volcanic activity in Australia, *Journal of Petrology*, **55**(7), 1317–1351.

[960] Vannucci, R., Bottazzi, P., Wulff-Pedersen, E., & Neumann, E.-R., 1998. Partitioning of REE, Y, Sr, Zr and Ti between clinopyroxene and silicate melts in the mantle under La Palma (Canary Islands): implications for the nature of the metasomatic agents, *Earth and Planetary Science Letters*, **158**(1-2), 39–51.

[961] Velasco-Tapia, F. & Verma, S. P., 2001. First partial melting inversion model for a rift-related origin of the sierra de chichinautzin volcanic field, central mexican volcanic belt, *International Geology Review*, **43**(9), 788–817.

[962] Verma, S. P., 2000. Geochemistry of the subducting cocos plate and the origin of subduction-unrelated mafic volcanism at the volcanic front of the central mexican volcanic belt, *Cenozoic tectonics and volcanism of Mexico*, **334**, 195.

[963] Verma, S. P., 2000. Geochemical evidence for a lithospheric source for magmas from Los Humeros caldera, Puebla, Mexico, *Chemical Geology*, **164**(1-2), 35–60.

[964] Verma, S. P., 2001. Geochemical and Sr-Nd-Pb isotopic evidence for a combined assimilation and fractional crystallisation process for volcanic rocks from the Huichapan caldera, Hidalgo, Mexico, *Lithos*, **56**(2-3), 141–164.

[965] Verma, S. P., 2001. Geochemical evidence for a lithospheric source for magmas from Acoculco caldera, Eastern Mexican Volcanic Belt, *International Geology Review*, **43**(1), 31–51.

[966] Verma, S. P., 2001. Geochemical evidence for a rift-related origin of bimodal volcanism at meseta rio San Juan, north-central mexican volcanic belt, *International Geology Review*, **43**(6), 475–493.

[967] Verma, S. P., 2002. Absence of Cocos plate subduction-related basic volcanism in southern Mexico: a unique case on Earth?, *Geology*, **30**(12), 1095–1098.

[968] Verma, S. P., 2003. Geochemical and Sr-Nd isotopic evidence for a rift-related origin of magmas in Tizayuca volcanic field, Central Mexican Volcanic Belt, *Journal of the Geological Society of India*, **61**(March), 257–276.

[969] Viccaro, M., Nicotra, E., & Urso, S., 2015. Production of mildly alkaline basalts at complex ocean ridge settings: Perspectives from basalts emitted during the 2010 eruption at the Eyjafjallajökull volcano, Iceland, *Journal of Geodynamics*, **91**, 51–64.

[970] Vickery, N. M., Dawson, M. W., Sivell, W. J., Malloch, K. R., & Dunlap, W. J., 2007. Cainozoic igneous rocks in the Bingara to Inverell area northeastern New South Wales, *Quarterly Notes of the Geological Society of New South Wales*, **123**, 1–27.

[971] Villemant, B., Salaün, A., & Staudacher, T., 2009. Evidence for a homogeneous primary magma at Piton de la Fournaise (La Réunion): A geochemical study of matrix glass, melt inclusions and Pélé's hairs of the 1998-2008 eruptive activity, *Journal of Volcanology and Geothermal Research*, **184**(1-2), 79–92.

[972] Vlastélic, I., Staudacher, T., & Semet, M., 2005. Rapid change of lava composition from 1998 to 2002 at Piton de la Fournaise (Réunion) inferred from Pb isotopes and trace elements: Evidence for variable crustal contamination, *Journal of Petrology*, **46**(1), 79–107.

[973] Vlastélic, I., Peltier, A., & Staudacher, T., 2007. Short-term (1998-2006) fluctuations of Pb isotopes at Piton de la Fournaise volcano (Reunion Island): Origins and constraints on the size and shape of the magma reservoir, *Chemical Geology*, **244**(1-2), 202–220.

[974] Vlastélic, I., Staudacher, T., Deniel, C., Devidal, J. L., Devouard, B., Finizola, A., & Télouk, P., 2013. Lead isotopes behavior in the fumarolic environment of the Piton de la Fournaise volcano (Réunion Island), *Geochimica et Cosmochimica Acta*, **100**, 297–314.

[975] Vlastélic, I., Di Muro, A., Bachèlery, P., Gurioli, L., Auclair, D., & Gannoun, A., 2018. Control of source fertility on the eruptive activity of Piton de la Fournaise volcano, La Réunion, *Scientific Reports*, **8**(1), 1–7.

[976] Vogel, D. C. & Keays, R. R., 1997. The petrogenesis and platinum-group element geochemistry of the Newer Volcanic Province, Victoria, Australia, *Chemical Geology*, **136**(3-4), 181–204.

[977] Volker, F., Altherr, R., Jochum, K. P., & McCulloch, M. T., 1997. Quaternary volcanic activity of the southern Red Sea: New data and assessment of models on magma sources and Afar plume-lithosphere interaction, *Tectonophysics*, **278**(1-4), 15–29.

[978] Wahab, A. A., Maaty, M. A. A., Stuart, F. M., Awad, H., & Kafafy, A., 2014. The geology and geochronology of Al Wahbah maar crater, Harrat Kishb, Saudi Arabia, *Quaternary Geochronology*, **21**, 70–76.

[979] Wahab, A. A., Ghoneim, M. F., Stuart, F. M., & Kafafy, A., 2015. On Cenozoic Volcanic Lava of Al Waabah Crater (Maklaa Tameya), Kishb Area , Saudi Arabia : Geochemical Assessment and Tectonic Implication, *Earth Science*, **1**, 57–82.

[980] Walker, R. T., Gans, P., Allen, M. B., Jackson, J., Khatib, M., Marsh, N., & Zarrinkoub, M., 2009. Late Cenozoic volcanism and rates of active faulting in eastern Iran, *Geophysical Journal International*, **177**(2), 783–805.

[981] Wallace, P. J., 1998. Water and partial melting in mantle plumes: Inferences from the dissolved H_2O concentrations of Hawaiian basaltic magmas, *Geophysical Research Letters*, **25**(19), 3639–3642.

[982] Wallace, P. J. & Carmichael, I. S., 1999. Quaternary volcanism near the Valley of Mexico: Implications for subduction zone magmatism and the effects of crustal thickness variations on primitive magma compositions, *Contributions to Mineralogy and Petrology*, **135**(4), 291–314.

[983] Walters, R. L., Jones, S. M., & Maclennan, J., 2013. Renewed melting at the abandoned Húnafloí Rift, northern Iceland, caused by plume pulsing, *Earth and Planetary Science Letters*, **377-378**, 227–238.

[984] Wandji, P., Tsafack, J., Bardintzeff, J., Nkouathio, D., Dongmo, A. K., Bellon, H., & Guillou, H., 2009. Xenoliths of dunites, wehrlites and clinopyroxenites in the basanites from Batoke volcanic cone (Mount Cameroon, Central Africa): petrogenetic implications, *Mineralogy and Petrology*, **96**(1-2), 81–98.

[985] Wang, B., Chen, J., Xu, J., & Wang, L., 2014. Geochemical and Sr–Nd–Pb–Os isotopic compositions of Miocene ultrapotassic rocks in southern Tibet: Petrogenesis and implications for the regional tectonic history, *Lithos*, **208**, 237–250.

[986] Wang, K. L., Chung, S. L., Lo, Y. M., Lo, C. H., Yang, H. J., Shinjo, R., Lee, T. Y., Wu, J. C., & Huang, S. T., 2012. Age and geochemical characteristics of Paleogene basalts drilled from western Taiwan: Records of initial rifting at the southeastern Eurasian continental margin, *Lithos*, **155**, 426–441.

[987] Wang, Q., McDermott, F., Xu, J.-f., Bellon, H., & Zhu, Y.-t., 2005. Cenozoic K-rich adakitic volcanic rocks in the Hohxil area, northern Tibet: lower-crustal melting in an intracontinental setting, *Geology*, **33**(6), 465–468.

[988] Wang, Y., Zhao, Z. F., Zheng, Y. F., & Zhang, J. J., 2011. Geochemical constraints on the nature of mantle source for Cenozoic continental basalts in east-central China, *Lithos*, **125**(3-4), 940–955.

[989] Wanming, D., 1991. Cenozoic volcanism and intraplate subduction at the northern margin of the Tibetan Plateau, *Chinese Journal of Geochemistry*, **10**(2), 140–152.

[990] Weaver, B. L., 1990. Geochemistry of highly-undersaturated ocean island basalt suites from the South Atlantic Ocean: Fernando de Noronha and Trindade islands, *Contributions to Mineralogy and Petrology*, **105**(5), 502–515.

[991] Wedepohl, K. H., Gohn, E., & Hartmann, G., 1994. Cenozoic alkali basaltic magmas of western Germany and their products of differentiation, *Contributions to Mineralogy and Petrology*, **115**(3), 253–278.

[992] Wei, F., Prytulak, J., Xu, J., Wei, W., Hammond, J. O., & Zhao, B., 2017. The cause and source of melting for the most recent volcanism in Tibet: A combined geochemical and geophysical perspective, *Lithos*, **288**, 175–190.

[993] Weidendorfer, D., Schmidt, M. W., & Mattsson, H. B., 2016. Fractional crystallization of Si-undersaturated alkaline magmas leading to unmixing of carbonatites on Brava Island (Cape Verde) and a general model of carbonatite genesis in alkaline magma suites, *Contributions to Mineralogy and Petrology*, **171**(5), 1–29.

[994] Weinstein, J. P., Fodor, R. V., & Bauer, G. R., 2004. Koolau shield basalt as xenoliths entrained during rejuvenated-stage eruptions: Perspectives on magma mixing, *Bulletin of Volcanology*, **66**(2), 182–199.

[995] Weinstein, Y., 2000. Spatial and temporal geochemical variability in basin-related volcanism, northern Israel, *Journal of African Earth Sciences*, **30**(4), 865–886.

[996] Weinstein, Y., 2007. A transition from strombolian to phreatomagmatic activity induced by a lava flow damming water in a valley, *Journal of volcanology and geothermal research*, **159**(1-3), 267–284.

[997] Weinstein, Y., 2012. Transform faults as lithospheric boundaries, an example from the Dead Sea Transform, *Journal of Geodynamics*, **54**, 21–28.

[998] Weinstein, Y., Navon, O., & Lang, B., 1994. Fractionation of Pleistocene alkali-basalts from the northern Golan Heights, Israel, *Isr J Earth Sci*, **43**, 63–79.

[999] Weinstein, Y., Navon, O., Altherr, R., & Stein, M., 2006. The role of lithospheric mantle heterogeneity in the generation of Plio-Pleistocene alkali basaltic suites from NW Harrat Ash Shaam (Israel), *Journal of Petrology*, **47**(5), 1017–1050.

[1000] Weis, D., Frey, F. A., Schlich, R., Schaming, M., Montigny, R., Damasceno, D., Mattielli, N., Nicolaysen, K. E., & Scoates, J. S., 2002. Trace of the Kerguelen mantle plume: Evidence from seamounts between the Kerguelen Archipelago and Heard Island, Indian Ocean, *Geochemistry, Geophysics, Geosystems*, **3**(6), 1–27.

[1001] Wendt, I., Kreuzer, H., Müller, P., Von Rad, U., & Raschka, H., 1976. K/Ar age of basalts from Great Meteor and Josephine seamounts (eastern North Atlantic), in *Deep Sea Research and Oceanographic Abstracts*, vol. 23, pp. 849–862, Elsevier.

[1002] West, H. B. & Leeman, W. P., 1994. The open-system geochemical evolution of alkalic cap lavas from Haleakala Crater, Hawaii, USA, *Geochimica et Cosmochimica Acta*, **58**(2), 773–796.

[1003] West, H. B., Garcia, M. O., Gerlach, D. C., & Romano, J., 1992. Geochemistry of tholeiites from Lanai, Hawaii, *Contributions to Mineralogy and Petrology*, **112**(4), 520–542.

[1004] White, C. M., Hart, W. K., Bonnichsen, B., & Matthews, D., 2002. Geochemical and Sr-isotopic variations in western snake river plain basalts, Idoho, *Geological Survey Bulletin*, **30**, 329–342.

[1005] White, W. M. & Duncan, R. A., 1993. Portrait of a pathological mantle plume, *Journal of Geophysical Research,*, **98**, 533–563.

[1006] Whitehead, P., 1991. The geology and geochemistry of Mt. Napier and Mt. Rouse, western Victoria, *The Cainozoic in Australia: A Re-Appraisal of the Evidence. Geological Society of Australia, Special Publications*, **18**, 309–320.

[1007] Whitehead, P. & Stephenson, P., 1998. Lava rise ridges of the Toomba basalt flow, north Queensland, Australia, *Journal of Geophysical Research: solid earth*, **103**(B11), 27371–27382.

[1008] Widom, E., Carlson, R., Gill, J., & Schmincke, H.-U., 1997. Th–Sr–Nd–Pb isotope and trace element evidence for the origin of the Sao Miguel, Azores, enriched mantle source, *Chemical Geology*, **140**(1-2), 49–68.

[1009] Wilkinson, J. F. & Hensel, H. D., 1988. The petrology of some picrites from Mauna Loa and Kilauea volcanoes, Hawaii, *Contributions to Mineralogy and Petrology*, **98**(3), 326–345.

[1010] Wilkinson, J. F. & Stolz, A. J., 1983. Low-pressure fractionation of strongly undersaturated alkaline ultrabasic magma: the olivine-melilite-nephelinite at Moiliili, Oahu, Hawaii, *Contributions to Mineralogy and Petrology*, **83**(3-4), 363–374.

[1011] Willbold, M. & Stracke, A., 2006. Trace element composition of mantle end-members: Implications for recycling of oceanic and upper and lower continental crust, *Geochemistry, Geophysics, Geosystems*, **7**(4), 1–30.

[1012] Williams, H. M., Turner, S. P., Pearce, J. A., Kelley, S., & Harris, N., 2004. Nature of the source regions for post-collisional, potassic magmatism in southern and northern Tibet from geochemical variations and inverse trace element modelling, *Journal of Petrology*, **45**(3), 555–607.

[1013] Wilson, M. & Downes, H., 1991. Tertiary - Quaternary extension-related alkaline magmatism in Western and Central Europe, *Journal of Petrology*, **32**(4), 811–849.

[1014] Wilson, M. & Patterson, R., 2001. Intraplate magmatism related to short-wavelength convective instabilities in the upper mantle: Evidence from the Tertiary-Quaternary volcanic province of western and central Europe, *Special Papers-Geological Society of America*, pp. 37–58.

[1015] Wilson, M., Downes, H., & Cebriá, J. M., 1995. Contrasting Fractionation Trends in Coexisting Continental Alkaline Magma Series; Cantal, Massif Central, France, *Journal of Petrology*, **36**(6), 1729–1753.

[1016] Wilson, M., Tankut, A., & Guleç, N., 1997. Tertiary volcanism of the Galatia province, north-west Central Anatolia, Turkey, *Lithos*, **42**(1–2), 105–121.

[1017] Wolde, B. & Widenfalk, L., 1994. Petrochemical and geochemical constraints on Cenozoic volcanism in Ethiopia, *African Geosciences Reviews*, **1**, 475–494.

[1018] WoldeGabriel, G., Yemane, T., Suwa, G., White, T., & Asfaw, B., 1991. Age of volcanism and rifting in the Burji-Soyoma area, Amaro Horst, southern Main Ethiopian Rift: geo- and biochronologic data, *Journal of African Earth Sciences*, **13**(3-4), 437–447.

[1019] Wolela, A., 2014. Volcanism, sedimentation, K/Ar and palynology studies, Yayu and Delbi-Moye Basins, Southwestern Plateau of Ethiopia, *Journal of African Earth Sciences*, **93**, 1–13.

[1020] Wolfe, E., Neal, C., Banks, N., Duggan, T.,*et al.*, 1988. Geologic observations and chronology of eruptive events, chap. 1 of, *The Puu Oo eruption of Kilauea Volcano, Hawaii*, pp. 1–97.

[1021] Wolfe, E. W., Garcia, M., Jackson, D., Koyanagi, R., Neal, C., & Okamura, A., 1987. The Puu Oo eruption of Kilauea volcano, episodes 1–20, January 3, 1983, to June 8, 1984, in *Volcanism in Hawaii: US Geological Survey Professional Paper*, vol. 1350, pp. 471–508, United States Government Printing Office, Washington, U.S.A.

[1022] Wood, B. J. & Blundy, J. D., 1997. A predictive model for rare earth element partitioning between clinopyroxene and anhydrous silicate melt, *Contributions to Mineralogy and Petrology*, **129**(2-3), 166–181.

[1023] Woodhead, J. D., 1996. Extreme HIMU in an oceanic setting: The geochemistry of Mangaia Island (Polynesia), and temporal evolution of the Cook-Austral hotspot, *Journal of Volcanology and Geothermal Research*, **72**(1-2), 1–19.

[1024] Woodhead, J. D. & Devey, C. W., 1993. Geochemistry of the Pitcairn seamounts, I: source character and temporal trends, *Earth and Planetary Science Letters*, **116**(1-4), 81–99.

[1025] Workman, R. K., Hart, S. R., Jackson, M., Regelous, M., Farley, K. A., Blusztajn, J., Kurz, M., & Staudigel, H., 2004. Recycled metasomatized lithosphere as the origin of the Enriched Mantle II (EM2) end-member: Evidence from the Samoan Volcanic Chain, *Geochemistry, Geophysics, Geosystems*, **5**(4), 1–44.

[1026] Wright, T. L., 1984. Origin of Hawaiian Tholeiite: a Metasomatic Model., *Journal of Geophysical Research*, **89**(B5), 3233–3252.

[1027] Xiachen, Z., 1991. Trace element geochemistry of tertiary continental alkali basalts from the Liuhe-Yizheng area, Jiangsu Province, China, *Chinese Journal of Geochemistry*, **10**(3), 204–216.

[1028] Xu, Y., Chung, S., Ma, J., & Shi, L., 2004. Contrasting Cenozoic Lithospheric Evolution and Architecture in the Western and Eastern Sino-Korean Craton: Constraints from Geochemistry of Basalts and Mantle Xenoliths, *The Journal of Geology*, **112**(5), 593–605.

[1029] Xu, Y. G., Ma, J. L., Frey, F. A., Feigenson, M. D., & Liu, J. F., 2005. Role of lithosphere-asthenosphere interaction in the genesis of Quaternary alkali and tholeiitic basalts from Datong, western North China Craton, *Chemical Geology*, **224**(4), 247–271.

[1030] Xu, Z., Zhao, Z. F., & Zheng, Y. F., 2012. Slab-mantle interaction for thinning of cratonic lithospheric mantle in North China: Geochemical evidence from Cenozoic continental basalts in central Shandong, *Lithos*, **146-147**, 202–217.

[1031] Yahiaoui, R., Dautria, J. M., Alard, O., Bosch, D., Azzouni-Sekkal, A., & Bodinier, J. L., 2014. A volcanic district between the Hoggar uplift and the Tenere Rifts: Volcanology, geochemistry and age of the In-Ezzane lavas (Algerian Sahara), *Journal of African Earth Sciences*, **92**, 14–20.

[1032] Yan, Q. S., Shi, X. F., Wang, K. S., Bu, W. R., & Xiao, L., 2008. Major element, trace element, and Sr, Nd and Pb isotope studies of Cenozoic basalts from the South China Sea, *Science in China, Series D: Earth Sciences*, **51**(4), 550–566.

[1033] Yang, A. Y., Zhao, T. P., Qi, L., Yang, S. H., & Zhou, M. F., 2011. Chalcophile elemental constraints on sulfide-saturated fractionation of Cenozoic basalts and andesites in SE China, *Lithos*, **127**(1-2), 323–335.

[1034] Yang, H.-J., Frey, F. A., Garcia, M. O., & Clague, D. A., 1994. Submarine lavas from Mauna Kea Volcano, Hawaii: Implications for Hawaiian shield stage processes, *Journal of Geophysical Research*, **99**(B8), 15577.

[1035] Yarmolyuk, V., Kovalenko, V., Ivanov, V., & Pokrovskii, B., 2003. Magmatism and geodynamics of the Southern Baikal volcanic region (mantle hot spot): results of geochronological, geochemical, and isotopic (Sr, Nd, and O) investigations, *Petrology*, **11**(1), 1–30.

[1036] Yaseen, I. A. A. B., 2014. Contribution to the Petrography, Geochemistry, and Petrogenesis of Zarqa-Ma'in Pleistocene Alkali Olivine Basalt Flow of Central Jordan, *International Journal of Geosciences*, **05**(06), 657–672.

[1037] Yaseen, I. A. A. B., 2016. Petrography, Geochemistry and Petrogensis of Basal Flow from Ar-Rabba Area, Central Jordan, *International Journal of Geosciences*, **07**(03), 378–396.

[1038] Yemane, T., WoldeGabriel, G., Tesfaye, S., Berhe, S., Durary, S., Ebinger, C., & Kelley, S., 1999. Temporal and geochemical characteristics of Tertiary volcanic rocks and tectonic history in the southern Main Ethiopian Rift and the adjacent volcanic fields, *Acta Vulcanologica*, **11**, 99–120.

[1039] Yokoyama, T., Aka, F. T., Kusakabe, M., & Nakamura, E., 2007. Plume–lithosphere interaction beneath Mt. Cameroon volcano, West Africa: constraints from ^{238}U–^{230}Th–^{226}Ra and Sr–Nd–Pb isotope systematics, *Geochimica et Cosmochimica Acta*, **71**(7), 1835–1854.

[1040] Zanettin, B., Bellieni, G., & Visentin, E. J., 2006. New radiometric age of volcanic rocks in the central Eritrean plateau (from Asmara to Adi Quala): considerations on stratigraphy and correlations, *Journal of African Earth Sciences*, **45**(2), 156–161.

[1041] Zanon, V. & Frezzotti, M. L., 2013. Magma storage and ascent conditions beneath Pico and Faial islands (Azores archipelago): A study on fluid inclusions, *Geochemistry, Geophysics, Geosystems*, **14**(9), 3494–3514.

[1042] Zanon, V. & Pimentel, A., 2015. Spatio-temporal constraints on magma storage and ascent conditions in a transtensional tectonic setting: the case of the Terceira Island (Azores), *American Mineralogist*, **100**(4), 795–805.

[1043] Zellmer, G., Rubin, K., Gronvold, K., & Juradochichay, Z., 2008. On the recent bimodal magmatic processes and their rates in the Torfajökull–Veidivötn area, Iceland, *Earth and Planetary Science Letters*, **269**(3-4), 388–398.

[1044] Zeng, G., Chen, L. H., Xu, X. S., Jiang, S. Y., & Hofmann, A. W., 2010. Carbonated mantle sources for Cenozoic intra-plate alkaline basalts in Shandong, North China, *Chemical Geology*, **273**(1-2), 35–45.

[1045] Zeng, G., Chen, L. H., Hofmann, A. W., Jiang, S. Y., & Xu, X. S., 2011. Crust recycling in the sources of two parallel volcanic chains in Shandong, North China, *Earth and Planetary Science Letters*, **302**(3-4), 359–368.

[1046] Zhang, H. H., Xu, Y. G., Ge, W. C., & Ma, J. L., 2006. Geochemistry of late Mesozoic-Cenozoic basalts in Yitong-Datun area, Jilin Province and its implication, *Acta Geologica Sinica*, **22**(6), 1576–1596.

[1047] Zhang, J. J., Zheng, Y. F., & Zhao, Z. F., 2009. Geochemical evidence for interaction between oceanic crust and lithospheric mantle in the origin of Cenozoic continental basalts in east-central China, *Lithos*, **110**(1-4), 305–326.

[1048] Zhang, M. & Guo, Z., 2016. Origin of Late Cenozoic Abaga–Dalinuoer basalts, eastern China: Implications for a mixed pyroxenite–peridotite source related with deep subduction of the Pacific slab, *Gondwana Research*, **37**(19), 130–151.

[1049] Zhang, M. & O'Reilly, S. Y., 1997. Multiple sources for basaltic rocks from Dubbo, eastern Australia: Geochemical evidence for plume-lithospheric mantle interaction, *Chemical Geology*, **136**(1-2), 33–54.

[1050] Zhang, M., Menzies, M. A., Suddaby, P., & Thirlwall, M. F., 1991. EM1 signature from within the post-Archean subcontinental lithospheric mantle: evidence from the potassic volcanic rocks in NE China, *Geochemical Journal*, **25**, 387–398.

[1051] Zhang, M., Suddaby, P., Thompson, R. N., Thirlwall, M. F., & Menzies, M. A., 1995. Potassic volcanic rocks in NE China: Geochemical constraints on mantle source and magma genesis, *Journal of Petrology*, **36**(5), 1275–1303.

[1052] Zhang, M., Stephenson, P., O'Reilly, S. Y., McCulloch, M. T., & Norman, M., 2001. Petrogenesis and geodynamic implications of late Cenozoic basalts in North Queensland, Australia: trace-element and Sr–Nd–Pb isotope evidence, *Journal of Petrology*, **42**(4), 685–719.

[1053] Zhang, W. H., Zhang, H. F., Fan, W. M., Han, B. F., & Zhou, M. F., 2012. The genesis of Cenozoic basalts from the Jining area, northern China: Sr-Nd-Pb-Hf isotope evidence, *Journal of Asian Earth Sciences*, **61**, 128–142.

[1054] Zhang, Y., Liu, J., & Meng, F., 2012. Geochemistry of Cenozoic volcanic rocks in Tengchong, SW China: relationship with the uplift of the Tibetan Plateau, *Island Arc*, **21**(4), 255–269.

[1055] Zhang, Z., Feng, C., Li, Z., Li, S., Xin, Y., Li, Z., & Wang, X., 2002. Petrochemical study of the Jingpohu Holocene alkali basaltic rocks, northeastern China, *Geochemical Journal*, **36**(2), 133–153.

[1056] Zhang, Z., Xiao, X., Wang, J., Wang, Y., & Kusky, T. M., 2008. Post-collisional Plio-Pleistocene shoshonitic volcanism in the western Kunlun Mountains, NW China: Geochemical constraints on mantle source characteristics and petrogenesis, *Journal of Asian Earth Sciences*, **31**(4-6), 379–403.

[1057] Zhao, Y. W., Fan, Q. C., Zou, H., & Li, N., 2014. Geochemistry of Quaternary basaltic lavas from the Nuomin volcanic field, Inner Mongolia: Implications for the origin of potassic volcanic rocks in Northeastern China, *Lithos*, **196-197**, 169–180.

[1058] Zhao, Z., Mo, X., Dilek, Y., Niu, Y., DePaolo, D. J., Robinson, P., Zhu, D., Sun, C., Dong, G., Zhou, S.,*et al.*, 2009. Geochemical and Sr–Nd–Pb–O isotopic compositions of the post-collisional ultrapotassic magmatism in SW Tibet: petrogenesis and implications for India intra-continental subduction beneath southern Tibet, *Lithos*, **113**(1-2), 190–212.

[1059] Zhi, X., Song, Y., Frey, F. A., Feng, J., & Zhai, M., 1990. Geochemistry of Hannuoba basalts, eastern China: Constraints on the origin of continental alkalic and tholeiitic basalt, *Chemical Geology*, **88**(1-2), 1–33.

[1060] Zhou, M.-F., Robinson, P. T., Wang, C. Y., Zhao, J.-H., Yan, D.-P., Gao, J.-F., & Malpas, J., 2012. Heterogeneous mantle source and magma differentiation of quaternary arc-like volcanic rocks from Tengchong, SE margin of the Tibetan Plateau, *Contributions to Mineralogy and Petrology*, **163**(5), 841–860.

[1061] Zhou, P. & Mukasa, S. B., 1997. Nd-Sr-Pb isotopic, and major-and trace-element geochemistry of Cenozoic lavas from the Khorat Plateau, Thailand: sources and petrogenesis, *Chemical Geology*, **137**(3-4), 175–193.

[1062] Zindler, A. & Hart, S., 1986. Chemical geodynamics, *Annual Review of Earth and Planetary Sciences*, **14**(1), 493–571.

[1063] Zou, H., Zindler, A., Xu, X., & Qi, Q., 2000. Major, trace element, and Nd, Sr and Pb isotope studies of Cenozoic basalts in SE China: Mantle sources, regional variations, and tectonic significance, *Chemical Geology*, **171**(1-2), 33–47.

[1064] Zou, H., Reid, M. R., Liu, Y., Yao, Y., Xu, X., & Fan, Q., 2003. Constraints on the origin of historic potassic basalts from northeast China by U-Th disequilibrium data, *Chemical Geology*, **200**(1-2), 189–201.

[1065] Zou, H., Fan, Q., & Yao, Y., 2008. U–Th systematics of dispersed young volcanoes in NE China: asthenosphere upwelling caused by piling up and upward thickening of stagnant Pacific slab, *Chemical Geology*, **255**(1-2), 134–142.
