# Supplementary material for: Global influence of mantle temperature and plate thickness on intraplate volcanism
Source: Nat Commun. 2021 Apr 6;12:2045. doi: 10.1038/s41467-021-22323-9 (PMC8024351; doi:10.1038/s41467-021-22323-9)
Supplement: Supplementary file 3 — Description of Additional Supplementary Files [file 41467_2021_22323_MOESM3_ESM.pdf]

## Description of Additional Supplementary Files

Supplementary Data 1

Database of locations; stratigraphic and radiometric dates; major and trace element compositions; and stable isotopic ratios for Quaternary-Neogene intraplate volcanic rocks.
